# Altitudinal Variation of Metabolites, Mineral Elements and Antioxidant Activities of *Rhodiola crenulata* (Hook.f. & Thomson) H.Ohba

**DOI:** 10.3390/molecules26237383

**Published:** 2021-12-05

**Authors:** Tingting Dong, Yueqi Sha, Hairong Liu, Liwei Sun

**Affiliations:** National Engineering Laboratory for Tree Breeding, College of Biological Sciences and Technology, Beijing Forestry University, Beijing 100083, China; dongtingting187@163.com (T.D.); sha1285325561@163.com (Y.S.); Liuhairong03@163.com (H.L.)

**Keywords:** rhodiola, high altitude, metabolite, element, change

## Abstract

*Rhodiolacrenulata* (Hook.f. & Thomson) H.Ohba is an alpine medicinal plant that can survive in extreme high altitude environments. However, its changes to extreme high altitude are not yet clear. In this study, the response of *Rhodiola crenulata* to differences in altitude gradients was investigated through chemical, ICP-MS and metabolomic methods. A targeted study of *Rhodiola crenulata* growing at three vertical altitudes revealed that the contents of seven elements Ca, Sr, B, Mn, Ni, Cu, and Cd, the phenolic components, the ascorbic acid, the ascorbic acid/dehydroascorbate ratio, and the antioxidant capacity were positively correlated with altitude, while the opposite was true for total ascorbic acid content. Furthermore, 1165 metabolites were identified: flavonoids (200), gallic acids (30), phenylpropanoids (237), amino acids (100), free fatty acids and glycerides (56), nucleotides (60), as well as other metabolites (482). The differential metabolite and biomarker analyses suggested that, with an increasing altitude: (1) the shikimic acid-phenylalanine-phenylpropanoids-flavonoids pathway was enhanced, with phenylpropanoids upregulating biomarkers much more than flavonoids; phenylpropanes and phenylmethanes upregulated, and phenylethanes downregulated; the upregulation of quercetin was especially significant in flavonoids; upregulation of condensed tannins and downregulation of hydrolyzed tannins; upregulation of shikimic acids and amino acids including phenylalanine. (2) significant upregulation of free fatty acids and downregulation of glycerides; and (3) upregulation of adenosine phosphates. Our findings provide new insights on the responses of *Rhodiola crenulata* to extreme high altitude adversity.

## 1. Introduction

*Rhodiola crenulata* (Hook.f. & Thomson) H.Ohba is an alpine medicinal species of the *Rhodiola* L., which is widely distributed in Asia, Europe, and the Americas. Several rhodiola species have been widely used in traditional medicine in Asia and Europe to improve human health and enhance physical fitness [1,2,3,4]. In China, *Rhodiola crenulata* has been recognized as a representative medicinal species, and its medicinal part rhizome is listed in the Chinese Pharmacopoeia [5,6]. Modern pharmacological studies have shown that *Rhodiola crenulata* is effective in enhancing immunity [7], delaying aging [8], preventing and treating cancer [9], protecting the heart [10], resisting plateau reactions [11]. In particular, it has been shown that *Rhodiola crenulata* has a variety of health benefits, including lung function and resistance to respiratory viral infections [12,13]. *Rhodiola crenulata* contains a variety of phytochemicals, including phytophenols (i.e., flavonoids + phenylpropanoids + tannins), gallic acids, amino acids, free fatty acids and glycerides, and nucleotides, and its health benefits are directly related to its phytochemicals [4,14]. *Rhodiola crenulata* grows mainly in Tibet, northwestern Yunnan, and western Sichuan in China, at altitudes of 2800–5600 m [15]. Therefore, it is able to adapt to the extreme high altitude environment, including low temperature, lack of oxygen and strong UV rays.

Multiple types of components in plants are significantly linked to environmental stresses. Flavonoids, phenylpropanoids and tannins, collectively known as phytophenols (also known as plant phenolics), are important antioxidants in plant defense mechanisms [16,17,18]. Ascorbic acid is also an important antioxidant in plants against oxidative stress induced by adversity [19,20]. Flavonoids and phenylpropanoids possess the ability to resist a variety of adversity stresses such as UV radiation, low temperature, drought, or pathogen [21,22,23,24,25,26]. It has been shown that the amount and type of plant phenolics and ascorbic acid in the plant body would respond to a variety of adversities and play an important role in helping plants to withstand adversity stress [27,28,29]. Amino acid classes play an important physiological role in promoting plant growth and enhancing plant stress resistance in several ways [30,31,32]. Aminobutyric acid is associated with enhancing plant energy metabolism [33,34]. Phenylalanine, generated by the shikimic acid pathway, is then a key upstream intermediate in the synthesis of phenylpropanoids-flavonoids in plants, and is an important indicator of altered phenylpropanoids-flavonoids biosynthesis [35,36,37].

A wide range of mineral elements are closely related to improving plant stress resistance, i.e., Ca, Sr, B, Mn, Ni, Cu, and Cd. Some studies have shown that they are closely related to the flavonoid composition and its content, the activities of antioxidant-related enzymes, and the cell wall synthesis in plants [38,39,40,41,42,43,44,45,46]. It has been reported that the roles of the above seven mineral elements in plants facilitate plants against adversities [38,47,48,49,50,51,52].

Other studies have shown that plant changes in response to adversity are also closely related to energy metabolism [53,54]. Fatty acids offer an essential source of energy for life activities in plants [55,56], while adenosine phosphates, which are involved in ATP energy metabolism, supply a more straightforward energy utilization for plants [57]. It has also been suggested that fatty acid content and adenosine phosphates content change in a variety of plants under salt, low temperature or drought stress [58,59,60,61,62]. This indicates that adversity stress may cause changes in energy metabolism in different aspects.

However, these studies mostly focus on a single adversity and lack the investigation of integrated adversity, which is precisely in line with the actual situation of ecological environment. Therefore, such research is highly representative and urgent for application.

Alpine habitats exhibit specific properties of integrated adversities such as strong radiation, oxygen deprivation, alpine cold, freezing and thawing, as well as regional-specific constraints on green vegetation cover [63,64,65]. Therefore, investigating plant changes in alpine habitats will provide a direct and effective way to understand plant change mechanisms from a holistic perspective and systemic cognitive level. Furthermore, it will provide strong support to improve green vegetation cover, crop resistance and crop quality in alpine and plateau areas, so as to adapt and improve the ecological environment for the practical development needs of human beings.

Our previous studies investigated the material change patterns of *Rhodiola crenulata* grown in the Sejila Mountains and Shannan in Tibet [14]. In this study, we investigated three vertical elevations (3933, 4249 and 4531 m) of *Rhodiola crenulata* from Dakazi Mountain (Ganzi Tibetan Autonomous Region, Sichuan, China), which is important for characterizing the effects of different elevations on *Rhodiola crenulata* in the same region. This is a rigorous investigation of the effects of different altitudes on the metabolites of *Rhodiola crenulata* in the same region and is highly relevant and representative. Furthermore, this study is more systematic and comprehensive in analyzing the changes of substances, which not only deepens the analysis of the changes of flavonoids, phenylpropanoids and amino acids, but also extends to seven new mineral elements, free fatty acids and glycerides and nucleotides related to the resistance.

## 2. Results and Discussion

### 2.1. Effect of Altitude Gradient on Oxidative Stress Levels in Rhodiola crenulata

**Figure 1:**
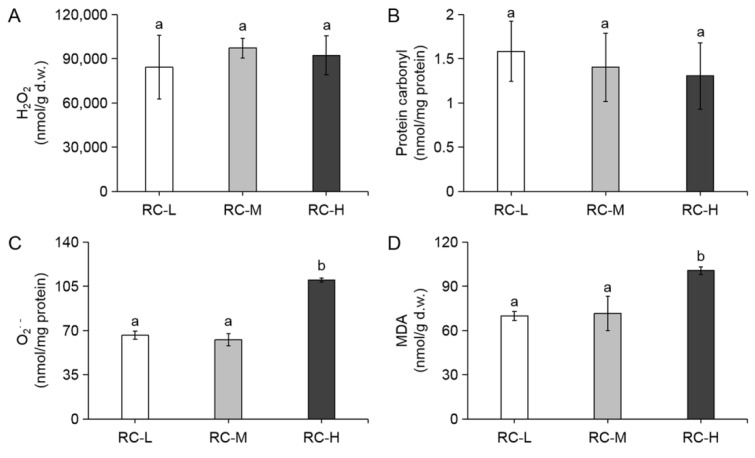
Oxidative states in rhizomes of *Rhodiola crenulata* (RC-L, RC-M, and RC-H) from different altitudes (3933, 4249 and 4531 m), respectively. (**A**): H_2_O_2_; (**B**): Protein carbonyl; (**C**): O_2_^·−^; (**D**): Malondialdehyde (MDA). Results are presented as the mean ± SD of six independent experiments (*n* = 6). In each column, different letters (a, b) mean significant differences between two groups (*p* < 0.05) found via a *t*-test. Note: *Rhodiola crenulata* samples are presented according to the ascending order of altitude from left to right.

Increased altitude leads to an intensification of integrated adversity stress consisting of multiple adversities such as UV radiation, low temperature, and hypoxia [63,64,65]. Excessive production of reactive oxygen species (ROS), such as H_2_O_2_ and O_2_^·−^, leads to oxidative stress [66], which induces the emergence of protein carbonylation and lipid peroxidation as indicated by Malondialdehyde (MDA) [67,68,69], which together indicate the level of oxidative stress. The levels of oxidative stress and its adaptability in *Rhodiola crenulata* at different altitudes were investigated by measuring H_2_O_2_, protein carbonylation, O_2_^·^^−^ and MDA levels. As shown in Figure 1A,B, H_2_O_2_ and protein carbonylation were stable at certain levels in all three elevation samples and did not increase significantly with elevation. As for O_2_^·−^ and MDA, both of them did not show any significant change between RC-L (3933 m) and RC-M (4249 m), while they showed a significant increase in RC-H (Figure 1C,D). The above results reveal that elevated altitude leads to increased oxidative stress and its level from the perspectives of H_2_O_2_, protein carbonylation, O_2_^·^^−^ and MDA, to which *Rhodiola crenulata* exhibits an excellent resistance.

### 2.2. Effect of Altitude Gradient on the Accumulation of Seven Mineral Elements in Rhodiola crenulata

**Figure 2:**
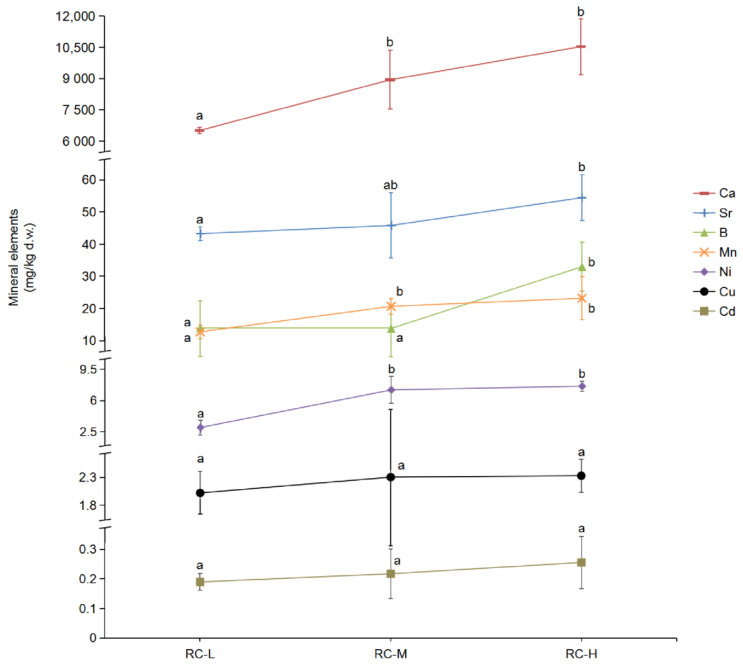
Accumulations of seven mineral elements in rhizomes of *Rhodiola crenulata* (RC-L, RC-M, and RC-H) from different altitudes (3933, 4249 and 4531 m), respectively. Calcium (Ca); Strontium (Sr); Boron (B); Manganese (Mn); Nickel (Ni); Copper (Cu); Cadmium (Cd). Results are presented as the mean ± SD of four independent experiments (*n* = 4). Different letters (a, b) mean significant differences between two groups (*p* < 0.05) found via a *t*-test. Note: *Rhodiola crenulata* samples are presented according to the ascending order of altitude from left to right.

To characterize the response of *Rhodiola crenulata* to the altitude gradient in terms of the resistance-related mineral element accumulation, we measured the changes of accumulation of seven plant resistance-related mineral elements in *Rhodiola crenulata* rhizomes grown at different altitudes (3933, 4249 and 4531 m), including Ca, Sr, B, Mn, Ni, Cu and Cd (Figure 2). The results showed that the accumulation of these seven mineral elements in *Rhodiola crenulata* rhizome at 3933–4531 m showed an overall increase with altitude, i.e., RC-L < RC-M < RC-H. The accumulation of five elements, i.e., Ca, Sr, B, Mn, and Ni, changed significantly with altitude in some altitude gradient zones. The accumulation of the remaining two elements, i.e., Cu and Cd, exhibited a rising trend with increasing altitude.

A thorough analysis of the variation characteristics of the seven mineral elements with altitude revealed that the accumulation of Ca, Sr, B, Mn and Ni increased significantly with altitude in some altitude gradient zones. Specifically, the accumulation of Ca, Mn, and Ni in the rhodiola rhizomes at both middle and high altitudes significantly increased compared to the lower altitude samples. This indicates that the altitude range from 3933–4249 m may contribute to the significant accumulation of Ca, Mn and Ni in *Rhodiola crenulata*. The accumulation of Sr in *Rhodiola crenulata* rhizomes at high altitude (i.e., 4531 m) was significantly higher than that in the low altitude (i.e., 3933 m) sample, which indicates that the accumulation of Sr in *Rhodiola crenulata* rhizomes may be closely related to the wider altitude span. The accumulation of B in rhodiola rhizomes from high elevation significantly exceeded those from low and middle elevations, denoting that elevations from 4249–4531 m might favor accumulation of B in *Rhodiola crenulata*. The accumulation of Cu and Cd in *Rhodiola crenulata* rhizomes showed a general increasing trend with elevation from 3933–4531 m, suggesting that elevation may promote the accumulation of these two elements in *Rhodiola crenulata* rhizomes.

The above results suggest that elevation in the altitude of 3933–4531 m contributes to the accumulation of seven mineral elements, Ca, Sr, B, Mn, Ni, Cu and Cd, in *Rhodiola crenulata* rhizomes. Ca contributes to plant resistance by enhancing pectin methyl esterase activity and inhibiting pectin hydrolase (i.e., polygalacturonase and galactosidase) activity, which plays a role in the formation of plant cell walls [39,40]. Acuña et al. [38] showed a positive correlation between the concentration of antioxidant active compounds such as catechin and epicatechin in grapes and the content of elements such as Sr and Mn. Hanaka et al. [43] found that the concentration of Sr in *Glycine max* was positively correlated with its isoflavone content. B attenuated oxidative stress induced by reactive oxygen species (e.g., H_2_O_2_ and O_2_) by enhancing the activity of antioxidant enzymes in the plant above ground [42]. Mn and Cu are metal auxiliary groups of superoxide dismutase (SOD), which have important effects on its activity of scavenging reactive oxygen species, such as H_2_O_2_ and O_2_ [45,46,52]. Kovacik et al. [41] found that Ni accumulation in *Matricaria chamomilla* increased its total phenol and chlorogenic acid content. Han et al. [44] reported that the accumulation of Cd in pumpkin roots increased its superoxide dismutase, peroxidase, and catalase activities to remove reactive oxygen species.

Furthermore, Singh et al. [70] found that the concentrations of Ca in the roots of wild *Rheum Emodi* Wallr. that collected in October was 3684.0 mg/kg of d.w. for 3400 m, which increased to 12,235.0 mg/kg of d.w. for 3600 m. This report was consistent with our results for Ca (6506.8 mg/kg of d.w. for 3933 m; 10,540.5 mg/kg of d.w. for 4531 m), indicating that altitude increase leaded to the increased accumulation of Ca in *Rhodiola crenulata* rhizomes. Negi et al. [71] reported the concentrations of Mn and Cu in the roots of wild *Swertia speciosa* (G. Don) that collected in September–October were 3.0 and 18.0 mg/kg of d.w. for 3400–3500 m, respectively, which increased to 16.0 and 19.0 mg/kg of d.w. for 4000 m, respectively. Their report coincided with our results (12.8 and 2.0 mg/kg of d.w. of Mn and Cu for 3933 m, respectively; 23.2 and 2.3 mg/kg of d.w. of Mn and Cu for 4531 m, respectively), implying that accumulations of Mn and Cu in *Rhodiola crenulata* rhizomes were augmented with elevations.

Regarding to the remaining four elements (namely Sr, B, Ni and Cd), to our best knowledge, we reported here for the first time their concentrations were increased with elevation rise in plants.

Combined with the above studies, we inferred that the increased concentrations of these seven mineral elements in *Rhodiola crenulata* rhizomes might strengthen the adaptation of *Rhodiola crenulata* to high altitude adversity in multiple fashions.

### 2.3. Effects of Altitude Gradient on Phenolic Components Content, Ascorbic Acid Content and Antioxidant Capacity in Rhodiola crenulata

**Figure 3:**
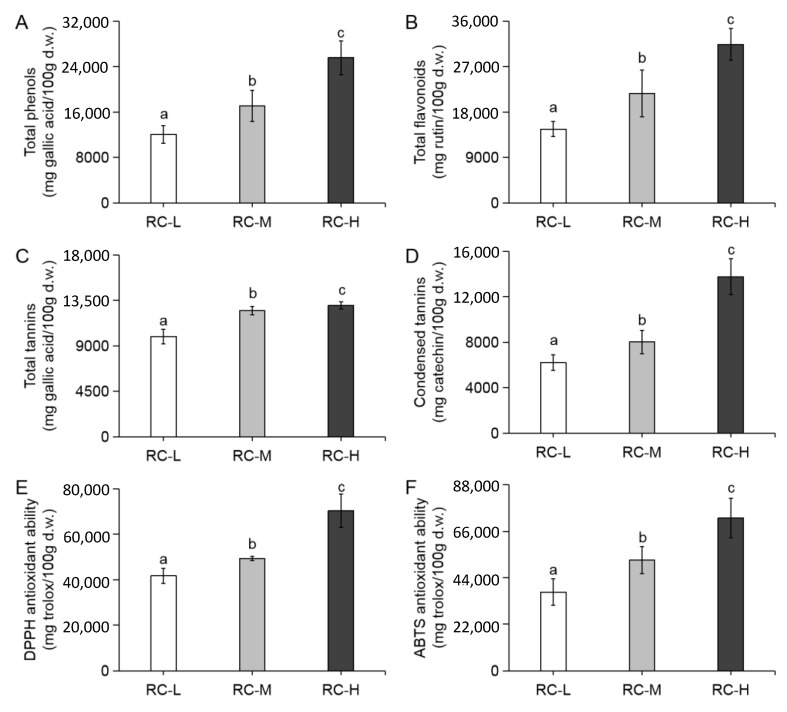
Characteristics of phenolic components and antioxidant capacity for rhizomes of *Rhodiola crenulata* (RC-L, RC-M, and RC-H) from different altitudes (3933, 4249 and 4531 m), respectively. (**A**): Total phenols; (**B**): Total flavonoids; (**C**): Total tannins; (**D**): Condensed tannins; (**E**): DPPH antioxidant ability (DPPH is a abbreviation of 2,2-diphenyl-1-picrylhydrazyl); (**F**): ABTS antioxidant ability (ABTS is a abbreviation of (2,2′-azino-bis(3-ethylbenzothiazoline-6-sulphonic acid). Gallic acid was used as a standard for Total phenols and Total tannins. Rutin and Catechin were used as standards for Total flavonoids and Condensed tannins, respectively. Trolox was used as a standard for DPPH and ABTS antioxidant ability. Results are presented as the mean ± SD of six independent experiments (*n* = 6) and are expressed as mg standard per 100 g d.w. of plant materials. In each column, different letters (a, b, c) mean significant differences between two groups (*p* < 0.05) found via a *t*-test. Note: *Rhodiola crenulata* samples are presented according to the ascending order of altitude from left to right.

To examine the response of *Rhodiola crenulata* to altitude gradient from the perspective of phenolic components and antioxidant capacity, we determined the changes of phenolic components content and antioxidant capacity in the rhizomes of *Rhodiola crenulata* grown at three altitudes (3933, 4249 and 4531 m). As shown in Figure 3 and Table 1, the total phenols, total flavonoids, total tannins and condensed tannins contents, and the antioxidant capacity of DPPH and ABTS showed a significant increase with altitude, i.e., RC-L < RC-M < RC-H, and there was a significant difference between the three altitude samples compared with each other. Our results suggest that elevated altitude facilitates the accumulation and enhancement of phenolic components and antioxidant capacity in *Rhodiola crenulata* rhizomes. In addition, the positive correlation between DPPH and ABTS free radical removal ability and phenolic content was also suggested, and the positive correlation with total phenols, total flavonoids and condensed tannins content was particularly noticeable when the increasing trend between the three altitude samples was further examined.

**Figure 4:**
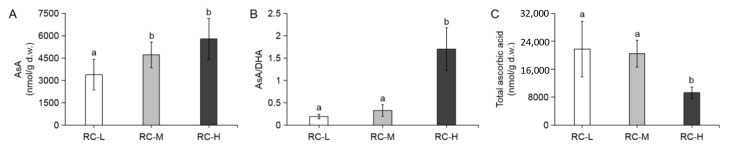
Contents of ascorbic acid in rhizomes of *Rhodiola crenulata* (RC-L, RC-M, and RC-H) from different altitudes (3933, 4249 and 4531 m), respectively. (**A**): AsA (ascorbic acid); (**B**): AsA/DHA (ascorbic acid/dehydroascorbate); (**C**): Total ascorbic acid. Results are presented as the mean ± SD of six independent experiments (*n* = 6). In each column, different letters (a, b) mean significant difference between two groups (*p* < 0.05) found via a *t*-test. Note: *Rhodiola crenulata* samples are presented according to the ascending order of altitude from left to right.

To probe the pattern of ascorbic acid response to the altitude gradient, we then determined the ascorbic acid content, ascorbic acid/dehydroascorbate ratio and total ascorbic acid content in the rhizomes of *Rhodiola crenulata* grown at three altitudes (3933, 4249 and 4531 m). As can be seen from Figure 4A,B, the ascorbic acid content and ascorbic acid/dehydroascorbate ratio (i.e., AsA/DHA) showed a significant increase with altitude, while the total ascorbic acid content showed a sharp decrease with elevation (Figure 4C). These results suggested a possible contribution of ascorbic acid (AsA) in the resistance of *Rhodiola crenulata* to stresses exacerbated by altitude, but the dramatic decrease in total ascorbic acid content suggested that the role of ascorbic acid might be limited.

The intensified UV-B radiation and low temperature stress at high altitude caused severe damage to the plant by introducing oxidative stress, which could be effectively counteracted by accumulating phenolic components to improve adaptive capacity so as to mitigate the adversity and survive better [72,73]. It has been shown that flavonoids, phenylpropanoids and tannins increase significantly in plants with elevation, which is consistent with our results [74,75,76,77].

Taken together, total ascorbic acid content in *Rhodiola crenulata* rhizomes at three altitudes (3933, 4249 and 4531 m) significantly decreased with altitude gradient, while total phenols, total flavonoids, total tannins, and condensed tannins contents, and antioxidant capacity showed significant increase. This suggests that phenolic components may serve a more significant role in the response of *Rhodiola crenulata* to altitude gradient.

The results of Section 2.3 for phenolic components and their closely related antioxidant capacity with increasing altitude favorably support the finding that increasing altitude leads to an increase in oxidative stress and its level in Section 2.1. This fully demonstrates the significance of understanding how phenolic components respond to altitude gradients.

### 2.4. Characteristics of Chemical Constituents of Rhodiola crenulata in Response to Altitude Gradient

To systematically characterize and gain insight into the chemical changes influenced by altitude, a UPLC-QqQ-MS-based metabolomics approach was used to analyze metabolites in *Rhodiola crenulata* at altitude gradient (RC-L, altitude of 3933 m; RC-M, altitude of 4249 m; RC-H, altitude of 4531 m).

A total of 1165 metabolites were identified, which were listed in Table S1, including flavonoids (200), gallic acid derivatives (30), phenylpropanoids (237), amino acids (100), free fatty acids and glycerides (56), nucleotides (60), and others (482), and then visualized as a heat map (Figure S1). It shows significant differences between three samples (RC-L, RC-M, and RC-H).

**Figure 5:**
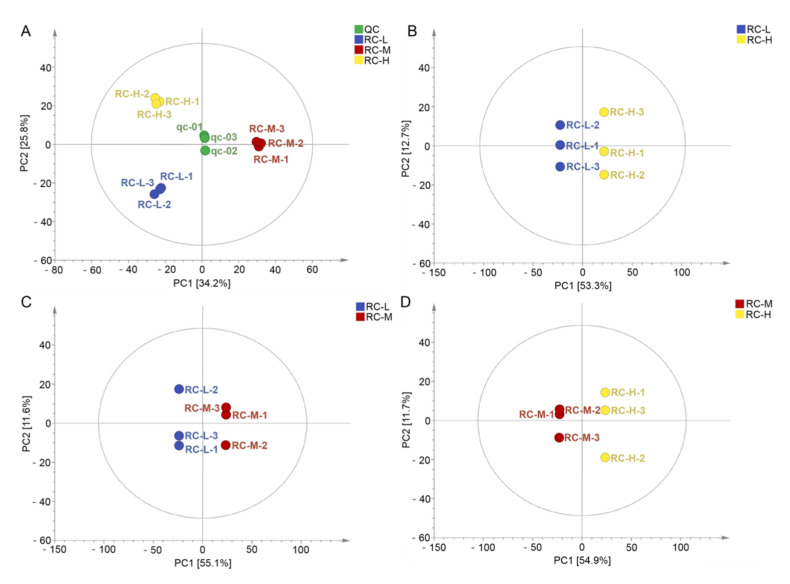
Principal component analysis (PCA) plots and orthogonal projections to latent structures discriminant analysis (OPLS-DA) of *Rhodiola crenulata* (RC-L, altitude of 3933 m; RC-M, altitude of 4249 m; RC-H, altitude of 4531 m) with three repeats for respective sample. (**A**): Two-dimensional scatter plot of the PCA for RC-L, RC-M, RC-H, and the quality control (QC). The QC was a mixture of RC-L, RC-M, and RC-H; (**B**–**D**): Score scatter plots of the OPLS-DA for RC-H vs. RC-L, RC-M vs. RC-L and RC-H vs. RC-M.

Unsupervised modeling using principal component analysis (PCA) was employed to assess the differences in the identified metabolites between the samples. The PCA plots for RC-L, RC-M, RC-H, and quality control (QC) samples in Figure 5A show that three samples were clustered into different regions, highlighting significant variations between RC-L, RC-M, and RC-H. Two principal components, PC1 and PC2, explained 60.0% of the total variance, manifesting a clear effect of altitude gradient on metabolites of *Rhodiola crenulata*.

An orthogonal projection to latent structures discriminant analysis (OPLS-DA) model was subsequently performed to compare metabolic characteristics of RC-L, RC-M, and RC-H. The OPLS-DA scatter scores of the three pairwise comparison groups are shown in Figure 5B–D, which show that RC-H and RC-L (Figure 5B), RC-M and RC-L (Figure 5C), and RC-H and RC-M (Figure 5D) were significantly distinguished in their pairwise comparisons.

Furthermore, the values of R2Y and Q2 by permutation tests of the OPLS-DA model confirmed the good credibility of individual models (Figure S2).

**Figure 6:**
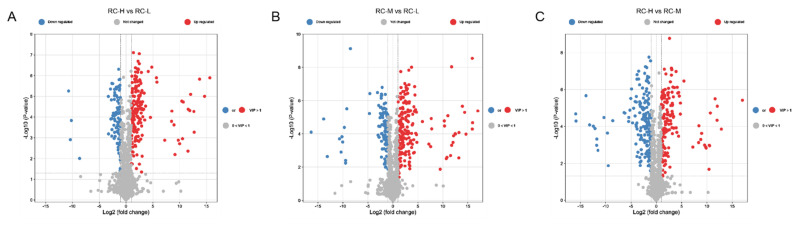
Differential metabolite analyses for RC-H vs. RC-L, RC-M vs. RC-L, and RC-H vs. RC-M with the criteria setting by simultaneously meeting variable importance in project (VIP) value > 1, |log^2^(fold change)| ≥ 1, and *p*-value < 0.05, plotted by volcano maps. Red and blue dots denoted upregulated and downregulated differential metabolites, respectively; gray dots represented non-differential metabolites. Upregulation denotes that the content of a metabolite in RC increased with elevation between two altitudes, and the opposite for downregulation. (**A**): RC-H vs. RC-L; (**B**): RC-M vs. RC-L; (**C**): RC-H vs. RC-M.

Next, differential metabolites were recognized and visualized as volcano maps based on the criteria of simultaneously meeting VIP > 1, |log^2^(fold change)| ≥ 1, and *p*-value < 0.05 (Figure 6). For both pairwise comparisons, 267 (173 up- and 94 downregulated), 314 (207 up- and 107 downregulated), and 335 (157 up- and 178 downregulated) differential metabolites were valid in distinguishing RC-H vs. RC-L, RC-M vs. RC-L, and RC-H vs. RC-M, respectively (also see Tables S2–S4). A heat map of changes of these differential metabolites in three rhodiola samples is shown in Figure S3.

Based on the changes in up- and downregulation of these differential metabolites, the specific alterations of *Rhodiola crenulata* metabolites in response to the elevation gradient from 3933–4531 m will be subsequently analyzed to explore the specific pattern of *Rhodiola crenulata* response to the elevation gradient.

#### 2.4.1. Biomarkers

For the differential metabolites, 22, 35 and 28 that contributed significantly to the differentiation between RC-H and RC-L, RC-M and RC-L, and RC-H and RC-M, were first investigated (Table 2). Among these 22, 35 and 28 metabolites, 19, 23 and 17 of them were found exclusively in *Rhodiola crenulata* at relatively higher altitudes, suggesting that the production of these 19, 23 and 17 were accompanied by elevations increasing from 3933–4531 m (RC-H vs. RC-L), 3933–4249 m (RC-M vs. RC-L), and 4249–4531 m (RC-H vs. RC-M). However, 3, 12 and 11 were only present at lower elevations in *Rhodiola crenulata* (correponding to RC-H vs. RC-L, RC-M vs. RC-L, and RC-H vs. RC-M), demonstrating that these 3, 12 and 11 were closely related to the elevation decreases in their comparisons. Therefore, these 22, 35 and 28 metabolites could be used as biomarker to differentiate between *Rhodiola crenulata* grown at elevations ranging from 3933–4531 in three pairwise comparisons mentioned here.

Among these 19, 23 and 17 found exclusively in RC with higher altitude, 9, 7 and 8 belong to phenylpropanoids while 2, 2 and 1 are flavonoids, accounting for 47.37 vs. 10.53% of these 19 (RC-H vs. RC-L), 30.44 vs. 8.70% of these 23 (RC-M vs. RC-L), and 47.06 vs. 5.88% of these 17 (RC-H vs. RC-M), indicating that upregulation of phenylpropanoids biomarker dominated over those flavonoids counterparts. There is evidence that intensive UV radiation leads to the accumulation of phenylpropanoids in plants, which function as potent protectants against UV radiation, as well as low temperatures [23,72,78,79]. Based on the above findings and the common knowledge that an elevated altitude has severe effects on the ambient UV radiation and temperature, it is speculated that these 9, 7 and 8 phenylpropanoids may be greatly responsible for the resistance of *Rhodiola crenulata* to increased altitude-related stress, particularly for increased UV radiation, as well as a decrease in temperature, and they might be used as indicators to evaluate the resistance of *Rhodiola crenulata* to cope with altitude stresses.

#### 2.4.2. Six Categories of Differential Metabolites

1. Flavonoids

Among the qualitative analysis of 200 flavonoids, 37 (31 upregulated vs. 6 downregulated, 83.78% upregulation rate, for RC-H vs. RC-L), 56 (43 upregulated vs. 13 downregulated, 76.79% upregulation rate, for RC-M vs. RC-L) and 37 (15 upregulated vs. 22 downregulated, 40.54% upregulation rate, for RC-H vs. RC-M) flavonoids were identified as differential metabolites, containing both biomarker and common differential metabolites (CDM). From the perspective of biomarker, the ratio of upregulation to downregulation in RC-H vs. RC-L is 2:0, and the upregulation rate was 100%; the ratio is 2:1 for RC-M vs. RC-L, with an upregulation rate of 66.7%; and the ratio is 1:0 for RC-H vs. RC-M, with an upregulation rate of 100%. These results suggest that elevation in the range of 3933–4531 m drives an overall enhancement of flavonoid biosynthesis.

Based on their corresponding aglucones, 25 (RC-H vs. RC-L), 38 (RC-M vs. RC-L) and 26 (RC-H vs. RC-M) were further grouped into four subclasses: quercetin and derivatives (12, 2 biomarkers + 10 CDMs, for RC-H vs. RC-L; 20, 3 biomarkers + 17 CDMs, for RC-M vs. RC-L; 9, 1 biomarker + 8 CDMs, for RC-H vs. RC-M), kaempferol and derivatives (6 CDMs for RC-H vs. RC-L, 8 CDMs for RC-M vs. RC-L, and 7 CDMs for RC-H vs. RC-M), catechins and derivatives (7 CDMs for RC-H vs. RC-L, 6 CDMs for RC-M vs. RC-L, and 6 CDMs for RC-H vs. RC-M), and Luteolin and derivatives (0 CDM for RC-H vs. RC-L, 4 CDMs for RC-M vs. RC-L, and 4 CDMs for RC-H vs. RC-M). They accounted for 67.57% of 37, 67.86% of 56, and 70.27% of 37 of the corresponding flavonoid differential metabolites, respectively.

Some studies have shown that these four flavonoids are important representative flavonoid classes in *Rhodiola crenulate* [4]. Therefore, the changes of these four flavonoids with altitude will be further discussed in detail to elucidate the specific alteration characteristics of the major representative flavonoids in *Rhodiola crenulata* in response to altitude changes in the range of 3933–4531 m.

**Figure 7:**
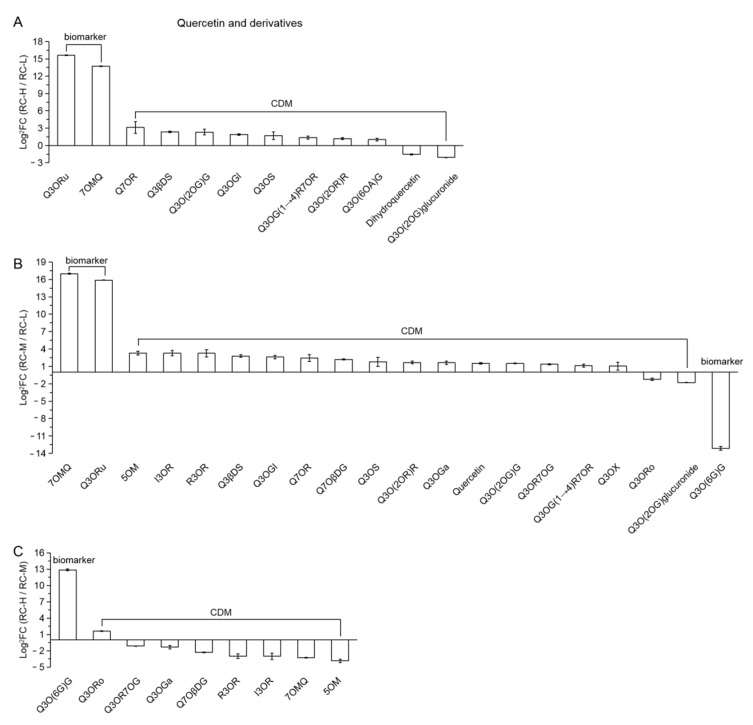
Comparison of differential metabolites of quercetin and its derivatives between RC-L, RC-M and RC-H. These were 2 biomarkers and 10 common differential metabolites (CDMs) for RC-H vs. RC-L (**A**), 3 biomarkers and 17 CDMs for RC-M vs. RC-L (**B**), and 1 biomarker and 8 CDMs for RC-H vs. RC-M (**C**), respectively. Upregulation denotes that the content of a metabolite in RC increased with elevation between two altitudes, and the opposite for downregulation. The Y-axis is shown as Log^2^FC values for the convenience of visual presentation (Note: The up-amplitude in the text refers to the FC value). The X-axis denotes metabolite names.

In RC-H vs. RC-L, 2 biomarkers and 10 CDMs were classified into the class of quercetin and its derivatives. According to the FC value size, these 12 substances are presented in Figure 7A in a descending order from left to right. We observed that 2 biomarkers and 8 CDMs were upregulated and 2 CDMs were downregulated for a total of 12 substances, suggesting that the elevation from 3933–4531 m overall enhanced the biosynthesis of quercetin and its derivatives class. Further analysis revealed that of these 12, 10 were quercetin glycosides (all showed upregulation with the exception of quercetin-3-O-(2′’-O-glucosyl) glucuronide(Q3O(2OG) glucuronide)), and only two were aglucones, namely Dihydroquercetin (downregulated) and 7-O-Methxyl Quercetin (7OMQ, upregulated). This suggests that the increase in altitude from 3933–4531 m contributed to the enhanced biosynthesis of various quercetin glycosides, making the overall change more active, while the biosynthetic species of quercetin aglucones were less affected.

In RC-M vs. RC-L, 3 biomarkers and 17 CDMs were classified into the class of quercetin and its derivatives. These 20 substances are presented in Figure 7B in a descending order from left to right according to the FC value size. As seen in the Figure, 2 biomarkers and 15 CDMs were upregulated, and 2 CDMs and 1 biomarker were downregulated, which indicates that the elevation from 3933–4249 m generally enhanced the biosynthesis of quercetin and its derivatives class. Further analysis revealed that 17 were quercetin glycosides, except for quercetin-3-O-robinobioside (Q3ORo), quercetin-3-O-(2′’-O-glucosyl) glucuronide (Q3O(2OG) glucuronide) and quercetin-3-O-(6′’-galloyl) galactoside (Q3O(6G)G), which were downregulated, while the other 14 showed upregulation; only three were aglucones, all of which were upregulated, namely two methoxy aglucones (7-O-Methxyl Quercetin (7OMQ), 5-O-Methylquercetin (5OM)) and quercetin. This suggests that the increase in altitude from 3933–4249 m enhanced the biosynthesis of multiple quercetin glycosides, while the aglucone species were relatively less affected, which is similar to RC-H vs. RC-L.

In RC-H vs. RC-M, one biomarker and eight CDMs were classified into the class of quercetin and its derivatives. According to the size of FC value, these 9 substances are presented in Figure 7C in descending order from left to right. We can see that one biomarker (quercetin-3-O-(6′’-galloyl) galactoside (Q3O(6G)G), 7396.52-fold change) and one CDM (quercetin-3-O-robinobioside (Q3ORo), 3.03-fold change) were upregulated for 2 substances and downregulated for 7 CDMs (for comparison, their FC values were normalized so that their range is 2.22–14.37), indicating a weakening of the enhanced effect of elevation from 4249–4531 m on the biosynthesis of quercetin and its derivatives.

Based on the results of pairwise comparisons of *Rhodiola crenulata* at the above three altitudes (3933, 4249 and 4531 m), we speculate that there may be a weak point affecting the biosynthesis of quercetin and its derivatives at an altitude of about 4200 m. The increase from below this weak point to above it has a weak effect on the enhancement of the biosynthesis of quercetin and its derivatives in *Rhodiola crenulata*.

In summary, the above results clearly demonstrate that elevation increases contribute to more active biosynthesis of quercetin and its derivative classes between the altitude of 3933–4531 m, mainly through the upregulation of multiple glycosides. Meanwhile, the number of differential metabolites of quercetin and its derivative classes was 12, 20 and 9 in the comparison of RC-H vs. RC-L, RC-M vs. RC-L, and RC-H vs. RC-M, which reached 32.43% of 37, 35.71% of 56 and 24.32% of 37 of the total number of flavonoid differential metabolites, respectively. This indicates that when the elevation change from 3933–4531 m, quercetin and its derivative classes accounted for the top of the significant variation in flavonoid metabolites in *Rhodiola crenulata*, amounting to about 1/3 of the total flavonoid differential metabolites.

It was found that exposure of *Vicia faba* [80], *Brassica napus* [26] and apple fruits [81] to UV-B radiation resulted in a significant increase in quercetin biosynthesis. Quercetin-3-O-rutinoside (Q3ORu, Rutin) and 7-O-Methxyl Quercetin (7OMQ, Rhamnetin) are both important antioxidants in plants [82,83], and we found that these substances were significantly upregulated as biomarkers. Based on the above findings, we suggest that the upregulation of quercetin and its derivatives at middle and high altitudes may be closely related to enhancing *Rhodiola crenulata*’s resistance to increased high-altitude UV stress.

**Figure 8:**
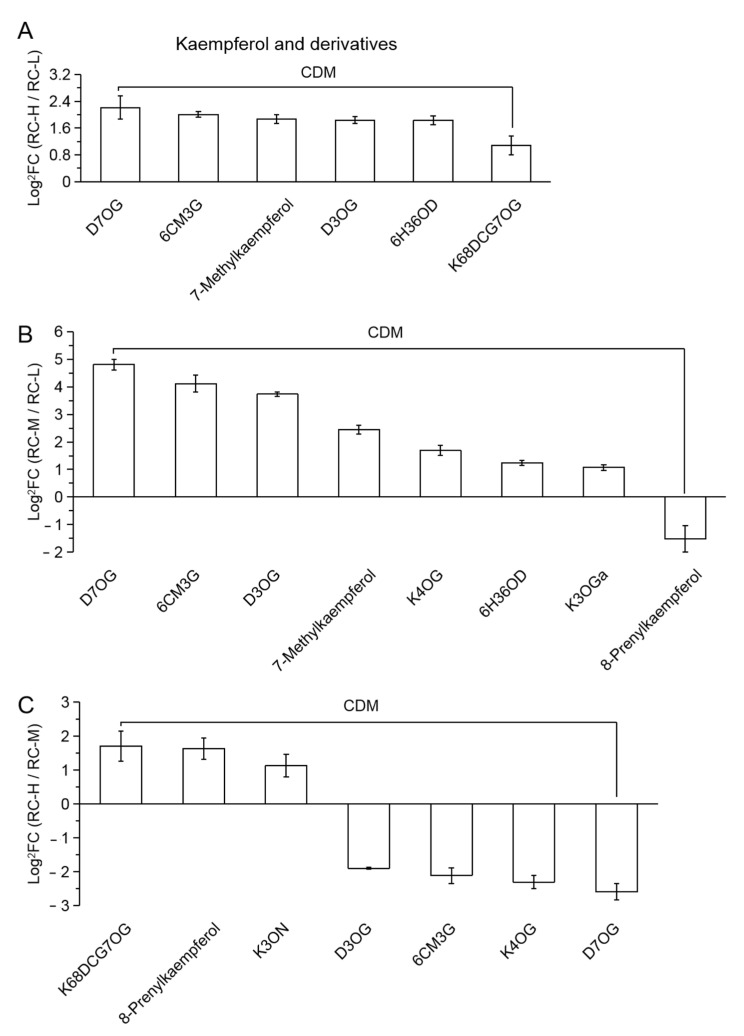
Comparison of differential metabolites of kaempferol and its derivatives between RC-L, RC-M and RC-H. These were 6 CDMs for RC-H vs. RC-L (**A**), 8 CDMs for RC-M vs. RC-L (**B**), and 7 CDMs for RC-H vs. RC-M (**C**), respectively. Upregulation denotes that the content of a metabolite in RC increased with elevation between two altitudes, and the opposite for downregulation. The Y-axis is shown as Log^2^FC values for the convenience of visual presentation (Note: The up-amplitude in the text refers to the FC value). The X-axis denotes metabolite names.

Among RC-H vs. RC-L and RC-M vs. RC-L, 6 and 8 CDMs were classified into the class of kaempferol and its derivatives, which are presented in Figure 8A,B in descending order according to the FC value, respectively. We found that all CDMs were upregulated except for 8-Prenylkaempferol which showed downregulation in RC-M vs. RC-L on the right-most side. Incorporating the results in Figure 8C, the 7 CDMs with the 3 upregulated and 4 downregulated in RC-H vs. RC-M, the biosynthetic patterns of kaempferol and its derivatives in the altitude range from 3933–4531 m were similar to those of quercetin and its derivatives, but the difference was that kaempferol and its derivatives did not show biomarker for the differential metabolites in the altitude range from 3933–4531 m. This indicates that the response of kaempferol and its derivatives to the changes in this altitude range was slightly smaller than that of the former.

Meanwhile, the number of differential metabolites of kaempferol and its derivatives was 6, 8 and 7 in the comparisons of RC-H vs. RC-L, RC-M vs. RC-L, and RC-H vs. RC-M. Within the range of variation, kaempferol and its derivatives accounted for the second highest significant variation in flavonoid metabolites in *Rhodiola crenulata* *with* 16.22% of 37, 14.29% of 56 and 18.92% of 37 in the total number of flavonoid differential metabolites, respectively, slightly lower than quercetin and its derivatives.

**Figure 9:**
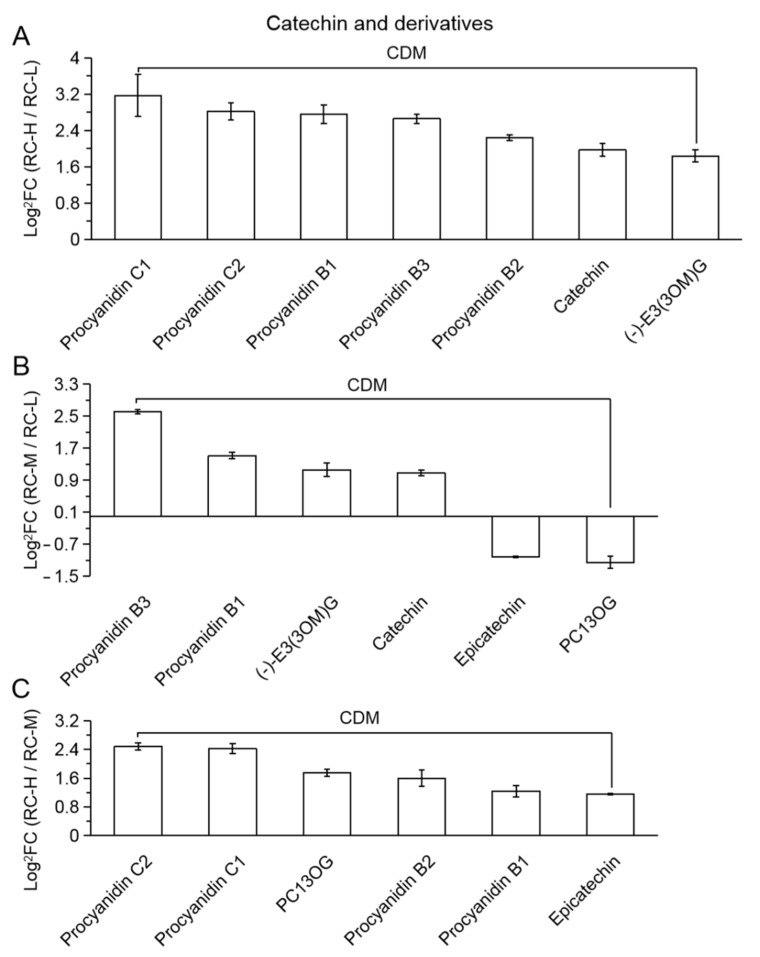
Comparison of differential metabolites of catechin and derivatives between RC-L, RC-M and RC-H. These were 7 CDMs for RC-H vs. RC-L (**A**), 6 CDMs for RC-M vs. RC-L (**B**), and 6 CDMs for RC-H vs. RC-M (**C**), respectively. Upregulation denotes that the content of a metabolite in RC increased with elevation between two altitudes, and the opposite for downregulation. The Y-axis is shown as Log^2^FC values for the convenience of visual presentation (Note: The up-amplitude in the text refers to the FC value). The X-axis denotes metabolite names.

In RC-H vs. RC-L, seven CDMs were classified into the class of catechin and derivatives. These 7 substances are presented in Figure 9A in descending order from left to right according to the size of FC values. As seen in the figure, all seven CDMs were upregulated, indicating that the elevation from 3933–4531 m enhanced the biosynthesis of catechin and derivatives. Further analysis revealed that the leftmost five were condensed tannin (most significantly upregulated, with a range of FC values from 9.02–4.73), and then the catechin (3.92-fold change) and (−)−Epicatechin-3-(3′’-O-methyl) gallate ((−)−E3(3OM)G, 3.58-fold change). This suggests that the increase in elevation from 3933–4531 m contributed to the overall enhancement of catechin and its derivative-like biosynthesis, specifically in the form of enhanced condensed tannin biosynthesis.

In RC-H vs. RC-M, six CDMs were classified into catechin and derivatives. These six substances are presented in Figure 9C in a descending order from left to right according to the FC value. We can see that all six CDMs were upregulated (FC values ranged from 5.62–2.22), indicating that the elevation from 4249–4531 m enhanced the biosynthesis of catechin and derivatives, which was similar to RC-H vs. RC-L, i.e., the enhanced condensed tannin biosynthesis.

In RC-M vs. RC-L, six CDMs were classified into the catechin and derivatives. These six substances are presented in Figure 9B in descending order from left to right according to the FC value. As seen in Figure 9B, four CDMs were upregulated (FC values ranging from 6.12–2.11, containing two condensed tannin) and two CDMs were downregulated (FC values ranging from 0.495–0.45, containing one condensed tannin). This indicates that the elevation from 3933–4249 m generally enhanced the biosynthesis of catechin and derivatives represented by condensed tannin. However, compared to all upregulation in RC-H vs. RC-L and RC-H vs. RC-M, some catechin and derivative metabolites showed downregulation in RC-M vs. RC-L. This suggests that there may be influence on the altered biosynthesis of catechin and derivatives around 4000 m.

Based on the above results of pairwise comparisons of catechin and derivatives at three altitudes (3933, 4249 and 4531 m), we hypothesized that there might be an intensification point affecting the alteration of biosynthesis of catechin and derivatives in *Rhodiola* *crenulata* around 4000 m, i.e., ascending from below 4000 m to above 4000 m, the enhancement of catechins and their derivatives-like biosynthesis in *Rhodiola* *crenulata* starts to occur. This pattern was significantly strengthened from 4249–4531 m (i.e., RC-H vs. RC-M), all CDMs were upregulated, 6 upregulated vs. 0 downregulated. From 3933–4531 m (i.e., RC-H vs. RC-L), the enhancement is most pronounced (all CDMs are upregulated and more numerous, 7 upregulated vs. 0 downregulated), i.e., it focuses on the enhanced development of condensed tannin biosynthesis.

Meanwhile, the number of catechin and its derivative-like differential metabolites was 7, 6 and 6 in the comparisons of RC-H vs. RC-L, RC-M vs. RC-L, and RC-H vs. RC-M, which account for 18.92% of 37, 10.71% of 56, and 16.22% of 37 in the total number of flavonoid differential metabolites, respectively. The catechin and derivatives accounted for the third most significant variation in flavonoid metabolites in *Rhodiola* *crenulata* within the elevation range variation.

**Figure 10:**
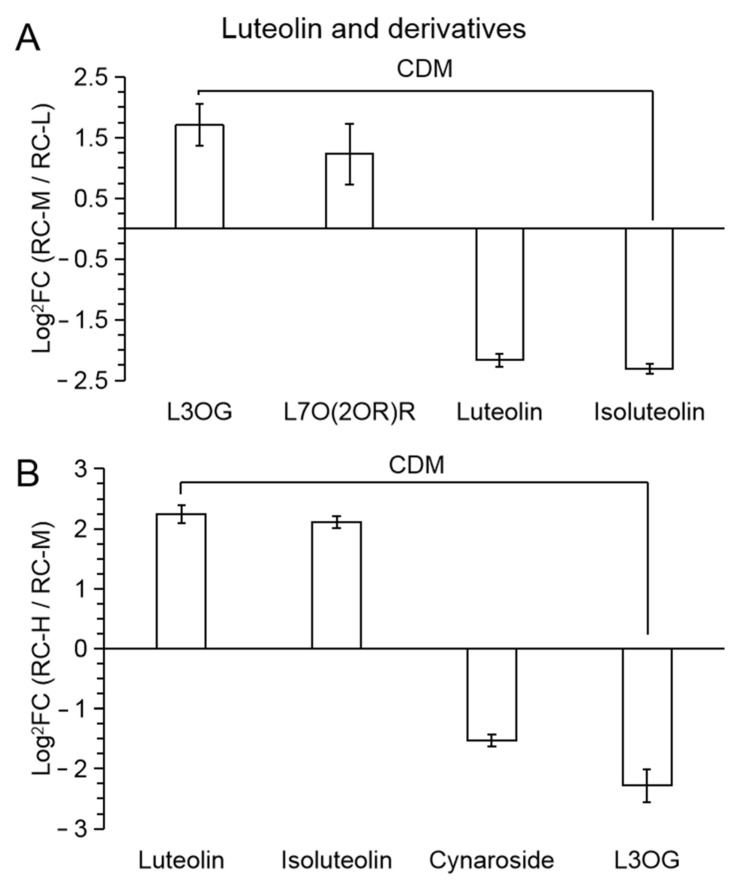
Comparison of differential metabolites of luteolin and its derivatives between RC-L, RC-M and RC-H. These were 0 CDMs for RC-H vs. RC-L, 4 CDMs for RC-M vs. RC-L (**A**), and 4 CDMs for RC-H vs. RC-M (**B**), respectively. Upregulation denotes that the content of a metabolite in RC increased with elevation between two altitudes, and the opposite for downregulation. The Y-axis is shown as Log^2^FC values for the convenience of visual presentation (Note: The up-amplitude in the text refers to the FC value). The X-axis denotes metabolite names.

The pattern of variation of Luteolin and their derivative classes was less clear than the three aforementioned flavonoid classes, with only four CDMs presented in RC-M vs. RC-L and RC-H vs. RC-M, respectively, while no differential metabolites were presented in RC-H vs. RC-L. From Figure 10A,B, although there were 2 upregulated and 2 downregulated CDMs in both RC-M vs. RC-L and RC-H vs. RC-M, however, in RC-M vs. RC-L, it exhibited 2 upregulated as glycosides (luteolin-3′-O-glucoside (L3OG); luteolin-7-O-(2′’-O-rhamnosyl)-O-rhamnosyl) rutinoside (L7O(2OR)R)), 2 downregulated to aglucones (Luteolin and Isoluteolin), and the pattern reversed in RC-H vs. RC-M, i.e., 2 upregulated to aglucones (Luteolin and Isoluteolin), 2 downregulated to glycosides (Luteolin-7-O-glucoside (Cynaroside) and luteolin-3′-O-glucoside (L3OG)). Thus, it indicates that the biosynthesis of Luteolin and its derivatives showed little response to elevation changes in the range of 3933–4531 m and did not show significant patterns.

The number of differential metabolites of Luteolin and its derivative classes was 0, 4 and 4 in the comparisons of RC-H vs. RC-L, RC-M vs. RC-L and RC-H vs. RC-M. It reaches 0% of 37, 7.14% of 56 and 10.81% of 37 of the total number of differential metabolites of flavonoids, respectively, which indicates that Luteolin and its derivative classes were not significantly affected by the above elevation changes due to the share of flavonoid metabolites in *Rhodiola* *crenulata* was also small.

By analyzing the specific change patterns of the above four major representative flavonoids of *Rhodiola* *crenulata* with altitude changes, it can be seen that the altitude increase in the range of 3933–4531 m contributed to the more active biosynthesis of the flavonoids of *Rhodiola* *crenulata*, which is characterized by: (1) the enhanced synthesis was evident in quercetin, catechin and kaempferol and their derivatives, with the most significant upregulation in quercetins, followed by kaempferols and then catechins, while the changes in luteolins were less clear; (2) there may be an intensification point affecting the biosynthesis of catechin and derivatives at around 4000 m, and there may be a weakening point affecting the biosynthesis of flavonoids represented by quercetin and its derivatives and kaempferol and its derivatives at around 4200 m; (3) the enhancement of biosynthesis of quercetin and kaempferol and their derivatives by the effect of altitude was mainly in the form of their glycosides, while the biosynthesis of catechin and derivatives was mainly characterized by the synthesis of condensed tannins.

This enhanced biosynthesis of flavonoids, mainly in the form of flavonoid glycosides, with increasing altitude generally agrees with our previous studies [14].

The new findings of this study include (1) the flavonoid catechin and derivatives exhibited mainly focused synthesis of condensed tannins with elevation in the range of 3933–4531 m, and (2) the enhanced biosynthesis of flavonoid quercetin and its derivatives was particularly significant in response to elevation in the range of 3933–4531 m.

The above results support that the total flavonoids and total phenols contents increased significantly with altitude (as shown in Figure 3A,B), and further reveal that the enhanced biosynthesis of quercetin, kaempferol, and catechin and their derivatives were the main contributors, among which the contribution of quercetin and its derivatives may be particularly important.

When combined with the results of Figure 3D, which showed a significant increase in condensed tannins from 3933–4531 m, these results suggest that the enhanced biosynthesis of catechin and derivatives is the main contributor, with condensed tannins being particularly important in terms of quantity and magnitude of upregulation.

Some studies have reported an enhanced effect of UV light or low temperature on the biosynthesis of quercetins, condensed tannins and flavonoid glycosides in plants [81,84,85]. These substances have a variety of resilient biological activities against UV radiation, low temperature or drought [21,25,26], and our results are consistent with these previous findings. There are inflection points in the literature showing the effect of altitude on different flavonoids, which corroborates our results [86,87,88].

Considering the previous literatures, we suggest that flavonoid glycosides represented by quercetins, and catechins represented by condensed tannins, both contribute to the altitudinal challenge of *Rhodiola* *crenulata*, conferring a strong adaptive capacity to altitude-integrated adversities consisting of UV, low temperature, hypoxia, and oxidative stress.

Quercetin, kaempferol, and catechins play important roles in cancer prevention or treatment of COVID-19 [89,90,91]. The flavonoid glycoside contributes to the bioavailability of flavonoid substances to the body, and condensed tannins play an important role in promoting human health [92,93,94,95]. Combining the existing works, we suggest that *Rhodiola* *crenulata* grown at higher altitudes may have more advantages for the supplementation of flavonoids of quercetin, kaempferol, and catechins represented by condensed tannins and for improving the bioavailability of these flavonoids for human health.

2. Gallic Acid and derivatives

**Figure 11:**
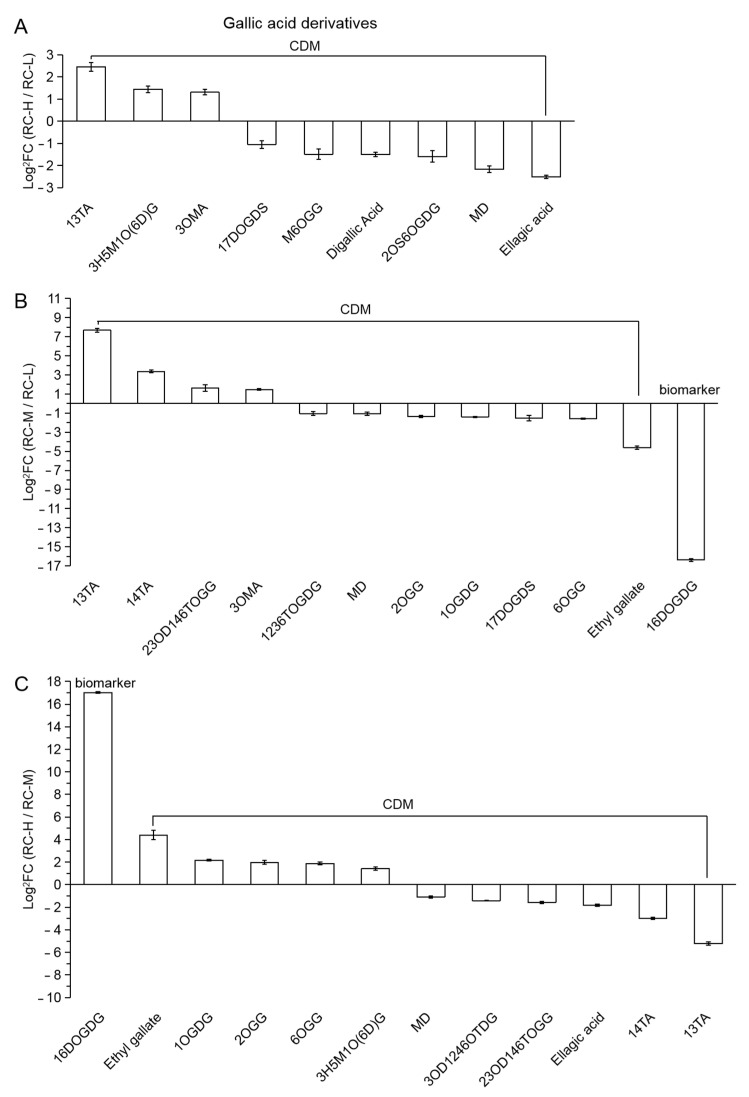
Comparison of differential metabolites of gallic acid derivatives between RC-L, RC-M and RC-H. These were 9 CDMs for RC-H vs. RC-L (**A**), 1 biomarker and 11 CDMs for RC-M vs. RC-L (**B**), and 1 biomarker and 11 CDMs for RC-H vs. RC-M (**C**). Upregulation denotes that the content of a metabolite in RC increased with elevation between two altitudes, and the opposite for downregulation. The Y-axis is shown as Log^2^FC values for the convenience of visual presentation (Note: The up-amplitude in the text refers to the FC value). The X-axis denotes metabolite names.

Among RC-H vs. RC-L and RC-M vs. RC-L, 9 CDMs and 12 (1 biomarker + 11 CDMs) differential metabolites were classified in this category, including gallic acid derivatives and hydrolyzed tannins formed from gallic acid and sugar, respectively. They are presented in a descending order from left to right in Figure 11A,B, respectively, based on the size of FC values. We found that the number of downregulations was significantly higher than the number of upregulations (6 downregulations vs. 3 upregulations; 8 downregulations vs. 4 upregulations), indicating that the elevation from 3933–4531 m and from 3933–4249 m both significantly attenuated the biosynthesis of gallic acid and its derivatives. We further analyzed the downregulated metabolites from the chemical structure perspective, and found that for the 6 downregulated CDMs in RC-H vs. RC-L, four were hydrolyzed tannins except digallic acid and ellagic acid; 8 downregulations were found in RC-M vs. RC-L (7 CDMs + 1 biomarker at the rightmost), all 7 belonged to the hydrolyzed tannins except ethyl gallate, which included 1,6-di-O-galloyl-D-glucose (16DOGDG) as a biomarker (0.00001-fold change). These results indicate that hydrolyzed tannins exhibit significant downregulation with increasing altitude, i.e., from 3933–4531 m and from 3933–4249 m.

As shown in Figure 11C, the 1biomarker and 11CDMs in RC-H vs. RC-M were classified in this category, and their variation characteristics were partially different from RC-H vs. RC-L and RC-M vs. RC-L. As seen in Figure 11C, the number of downregulated is the same as the number of upregulated (6 downregulated vs. 6 upregulated), and the number of their downregulated hydrolyzed tannins versus upregulated counterparts is 3 downregulated (Monogalloyl-diglucose; 3-O-Digalloyl-1,2,4,6-O-tetragalloyl-D-glucose; 2,3-O-Digalloyl-1,4,6-tri-O-galloyl- glucose) vs. 4 upregulated (1,6-Di-O-Galloyl-D-Glucose as biomarker; 1-O-Galloyl-D- glucose; 2-O-Galloyl-glucose; 6-O-Galloyl-glucose). This indicates that the increase in altitude from 4244–4531 m had little effect on the biosynthesis of gallic acid derivatives.

Based on the results of the pairwise comparisons of *Rhodiola crenulata* at the above three altitudes (3933, 4249 and 4531 m), we speculate that there may be an intensification point affecting the biosynthesis of gallic acid derivatives and hydrolyzed tannins in *Rhodiola crenulata* at about 4200 m, and the increase from below this intensification point to above it has an reverse effect on the weakening of the biosynthesis of gallic acid and its derivatives in *Rhodiola crenulata*.

In summary, the above results indicate that elevation in the range of 3933–4531 m generally weakens the biosynthesis of gallic acid and its derivatives mainly by promoting the downregulation of various hydrolyzed tannins. This result is generally consistent with our previous finding [14].

Combined with previous studies [96,97,98,99], we suggest that the downregulation of gallic acid and its derivatives in *Rhodiola crenulata* rhizomes with elevation may be related to changes in biotic stress and UV intensity changes with elevation.

Considering the significant upregulation of condensed tannins in catechin and derivatives with altitude, our finding of an overall downregulation of hydrolyzed tannins with altitude explains the increase in total tannins content (total tannins = condensed tannins + hydrolyzed tannins) from 3933–4531 m, which is significantly weaker than the increase in condensed tannins content (Figure 3C,D).

3. Phenylpropanoids

Among the 237 phenylpropanoid metabolites characterized, 47 (28 upregulation vs. 19 downregulation), 48 (26 upregulation vs. 22 downregulation) and 44 (26 upregulation vs. 18 downregulation) phenylpropanoids were identified as differential metabolites in RC-H vs. RC-L, RC-M vs. RC-L and RC-H vs. RC-M, containing biomarker and CDM, respectively. These results indicated a overall upregulation of phenylpropanoids in response to elevation in the range of 3933–4531 m.

Based on their basic structures, 47 (RC-H vs. RC-L), 48 (RC-M vs. RC-L) and 44 (RC-H vs. RC-M) were further grouped into four subclasses. These four subclasses, namely Cinnamic acid-Coumaroyl and derivatives, phenylmethanes, phenylethanes and shikimic acids.

Therefore, the changes of these four phenylpropanoids with altitude will be further discussed in detail to elucidate the specific patterns of changes of phenylpropanoids in *Rhodiola crenulata* with altitude.

**Figure 12:**
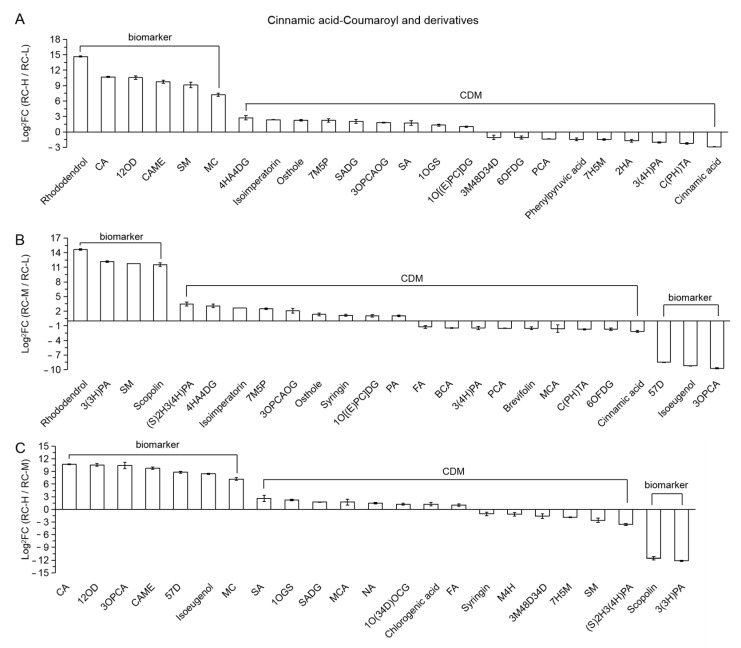
Comparison of differential metabolites of Cinnamic acid-Coumaroyl and its derivatives between RC-L, RC-M and RC-H. These were 6 biomarkers and 18 CDMs for RC-H vs. RC-L (**A**), 7 biomarkers and 18 CDMs for RC-M vs. RC-L (**B**), and 9 biomarkers and 14 CDMs for RC-H vs. RC-M (**C**). Upregulation denotes that the content of a metabolite in RC increased with elevation between two altitudes, and the opposite for downregulation. The Y-axis is shown as Log^2^FC values for the convenience of visual presentation (Note: The up-amplitude in the text refers to the FC value). The X-axis denotes metabolite names.

Twenty-four (6 biomarkers and 18 CDMs) and 23 (9 biomarkers and 14 CDMs) differential metabolites in RC-H vs. RC-L and RC-H vs. RC-M were classified as Cinnamic acid-Coumaroyl and its derivatives, respectively. They are presented in a descending order according to the FC values from left to right in Figure 12A,C, respectively. We observe that the number of upregulations is significantly higher than the number of downregulations (15 upregulations vs. 9 downregulations; 15 upregulations vs. 8 downregulations). Six biomarkers in RC-H vs. RC-L show significant upregulations, with FC values ranging from 25,852.22–147.26. Seven biomarkers out of nine in RC-H vs. RC-M display significant upregulations, with a FC from 1654.26–147.26. Two biomarkers showed a significant downregulation (0.0003- and 0.0002-fold change, respectively). These results suggest that the biosynthesis of Cinnamic acid-Coumaroyl and its derivatives was significantly enhanced by increasing altitude from 3933–4531 m and from 4249–4531 m.

Further analysis from the perspective of chemical structure revealed that 11 of the 15 upregulations in RC-H vs. RC-L were metabolites of phenylpropanes indirectly derived from Cinnamic acid-Coumaroyl via the phenylpropanoids pathway (Cinnamic acid-Coumaroyl indirect derivatives), such as Caffeoylbenzoyltartaric acid, 1,2-O-Diferuloylglycerol and Chlorogenic acid methyl ester. This includes all 6 upregulated biomarkers out of 11. The other 4 upregulated ones are direct derivatives of Cinnamic acid-Coumaroyl, which are 4-Hydroxycinnamyl alcohol 4-D-glucoside (4HA4DG), 7-methoxy-5-prenyloxycoumarin (7M5P), 3-O-p-Coumaroylquinic acid O-glucoside (3OPCAOG) and 1-O-[(E)-p-coumaroyl]-D-glucose (1O[(E)PC]DG); 13 out of the 15 upregulations in RC-H vs. RC-M are indirect Cinnamic acid-Coumaroyl derivatives, which includes five upregulated biomarkers, and the remaining two belongs to direct derivatives of Cinnamic acid-Coumaroyl, i.e., 3-O-p-Coumaroylquinic acid (3OPCA) and 5,7-dimethoxycoumarin (57D). The number of indirect derivatives vs. direct derivatives regarding the 9 downregulated CDMs in RC-H vs. RC-L was 4 vs. 5; the number of indirect derivatives vs. direct derivatives regarding the 8 downregulated (6 CDMs + 2 biomarkers at the rightmost) in RC-H vs. RC-M was 5 (including 2 biomarkers) vs. 3.

Token together, the number of indirect derivatives vs. direct derivatives was 15 vs. 9 in RC-H vs. RC-L, and 18 vs. 5 in RC-H vs. RC-M, and the number of upregulations is significantly higher than the number of downregulations for the indirect derivatives (11 upregulations vs. 4 downregulations in RC-H vs. RC-L; 13 upregulations vs. 5 downregulations), while changes of direct derivatives were less clear (also See Tables S2 and S4). These results indicate that with increasing altitude (i.e., from 3933–4531 m and from 4249–4531 m), the biosynthesis of the indirect Cinnamic acid-Coumaroyl-derived phenylpropane metabolites via the phenylpropane pathway is more affected than the direct Cinnamic acid-Coumaroyl derivatives, and being obviously upregulated.

Seven biomarkers and 18 CDMs in RC-M vs. RC-L were classified into this category, including indirect and direct derivatives. As shown in Figure 12B, the number of upregulated is slightly higher than the number of downregulated (13 upregulated vs. 12 downregulated), however, from the biomarker perspective, the upregulated biomarkers are higher than the downregulated biomarkers (4 vs. 3), with FC values of 25,319.26–2996.11 vs. 0.003–0.001. The number of indirect derivatives vs. direct derivatives was 17 vs. 8, and the number of upregulations is slightly higher than the number of downregulations for the indirect derivatives (9 upregulations vs. 8 downregulations (also See Table S3). Above findings revealed that the enhancing trend of biosynthesis of Cinnamic acid-Coumaroyl and its derivative classes, especially for the indirect derivatives (i.e., phenylpropanes) starts to appear as the altitude increased from 3933–4249 m.

Based on the results of the above-mentioned pairwise comparisons of *Rhodiola crenulata* at three altitudes (3933, 4249 and 4531 m), it was observed that the tendency of enhanced biosynthesis of Cinnamic acid-Coumaroyl and its derivatives from 3933–4249 m started to appear, and was mainly characterized by the response of indirect derivatives (i.e., phenylpropanes), which was more evident during the increase from 3933–4531 m and from 4249–4531 m.

Based on the above results, we found for the first time that the change of phenylpropanoids with elevation was characterized by the enhanced synthesis of metabolites indirectly derived from Cinnamic acid-Coumaroyl via the phenylpropanoids pathway (hereafter referred to as “phenylpropanoids variation characteristics”). Also, phenylpropanes highlighted these characteristics here.

In addition, the number of Cinnamic acid-Coumaroyl and its derivative-like differential metabolites was 24, 25 and 23 in the comparisons of RC-H vs. RC-L, RC-M vs. RC-L, and RC-H vs. RC-M, reaching 51.06% of 47, 52.08% of 48 and 52.27% of 44 of the total differential metabolites of phenylpropanoids, respectively. This indicates that the Cinnamic acid-Coumaroyl and its derivatives accounted for the most significant differential variation of phenylpropanoids in the elevation range change from 3933–4531 m.

**Figure 13:**
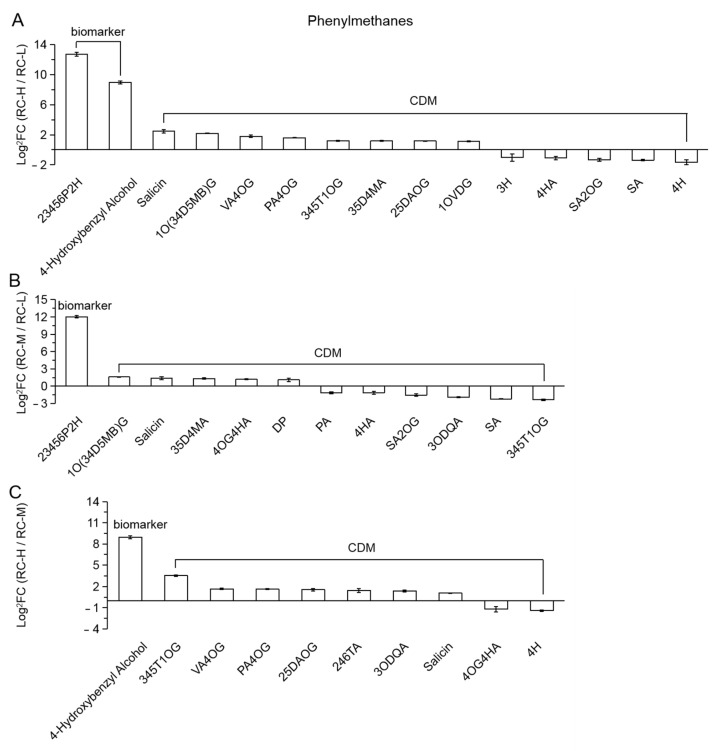
Comparison of differential metabolites of phenylmethanes between RC-L, RC-M and RC-H. These were 2 biomarkers and 13 CDMs for RC-H vs. RC-L (**A**), 1 biomarker and 11 CDMs for RC-M vs. RC-L (**B**), and 10 CDMs for RC-H vs. RC-M (**C**). Upregulation denotes that the content of a metabolite in RC increased with elevation between two altitudes, and the opposite for downregulation. The Y-axis is shown as Log^2^FC values for the convenience of visual presentation (Note: The up-amplitude in the text refers to the FC value). The X-axis denotes metabolite names.

Among RC-H vs. RC-L and RC-H vs. RC-M, 15 (2 biomarkers and 13 CDMs) and 10 CDMs differential metabolites were classified as phenylmethanes, respectively. They are presented in a descending order from left to right in Figure 13A,C according to the size of FC values. As can be seen from the figure, the number of upregulations is significantly higher than the number of downregulations (10 upregulations vs. 5 downregulations; 8 upregulations vs. 2 downregulations), and two and one upregulated biomarkers existed in RC-H vs. RC-L and RC-H vs. RC-M, respectively. These results indicate that the elevation from 3933–4531 m and from 4249–4531 m significantly enhances the biosynthesis of phenylmethanes.

One biomarker and 11 CDMs were classified in RC-M vs. RM-L. As seen in Figure 13B, the number of upregulated was equal to the number of downregulated (6 upregulated vs. 6 downregulated), however; while from the perspective of biomarker, the upregulated was significantly higher than the downregulated (1 vs. 0), i.e., 2,3,4,5,6-pentahydroxyhexyl 2-hydroxybenzoate, with an FC value of 4217.04. It can be concluded that the biosynthesis of phenylmethanes is enhanced by the increase in altitude from 3933–4249 m. Combining the results of the above three altitudes (3933, 4249 and 4531 m), we found that the response of phenylmethanes was consistent with that of Cinnamic acid-Coumaroyl and its derivatives with altitude, i.e., the trend of enhanced biosynthesis of phenylmethanes began to appear from 3933–4249 m. This tendency was more obvious during the elevation from 3933–4531 m and from 4249–4531 m. It should be noted that phenylmethanes can still be considered as a class of metabolites indirectly derived from Cinnamic acid-Coumaroyl via the phenylpropanoids pathway, thus it further enriches the above-mentioned “phenylpropanoids variation characteristics”.

In addition, the number of differential metabolites of phenylmethanes was 15, 12 and 10 in the comparisons of RC-H vs. RC-L, RC-M vs. RC-L, and RC-H vs. RC-M, which reached 31.92% of 47, 25% of 48 and 22.73% of 44 of the total differential metabolites of phenylpropanoids, respectively. This indicates that the phenylmethanes class is immediately behind Cinnamic acid-Coumaroyl and its derivatives for significant differences in the metabolites of phenylpropanoids over the elevation range of 3933–4531 m.

**Figure 14:**
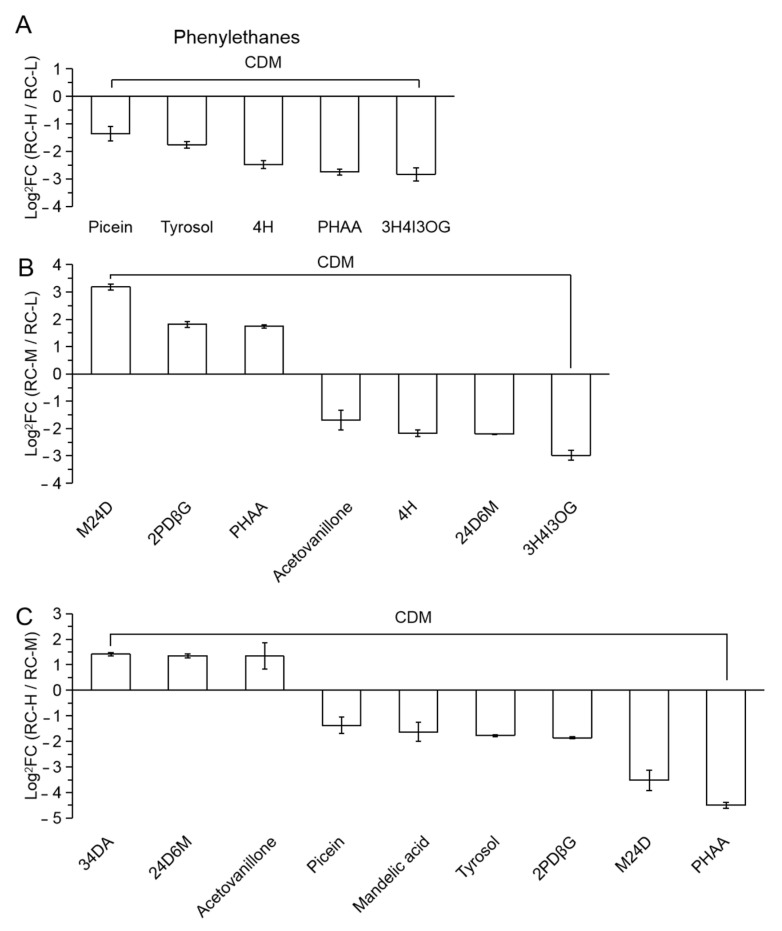
Comparison of differential metabolites of phenylethanes between RC-L, RC-M and RC-H. These were 5 CDMs for RC-H vs. RC-L (**A**), 7 CDMs for RC-M vs. RC-L (**B**), and 9 CDMs for RC-H vs. RC-M (**C**). Upregulation denotes that the content of a metabolite in RC increased with elevation between two altitudes, and the opposite for downregulation. The Y-axis is shown as Log^2^FC values for the convenience of visual presentation (Note: The up-amplitude in the text refers to the FC value). The X-axis denotes metabolite names.

The number of differential metabolites of phenylethanes in RC-H vs. RC-L, RC-M vs. RC-L and RC-H vs. RC-M were 0 vs. 5, 3 vs. 4 and 3 vs. 6, respectively, as shown in Figure 14A–C, showing an overall trend of downregulation of phenylethanes with elevation. This indicates that medium and low altitudes are more beneficial for the accumulation of phenylethanes in *Rhodiola crenulata* rhizomes compared to high altitudes. In particular, it should be noted that phenylethanes can still be considered as the category of metabolites indirectly derived from Cinnamic acid-Coumaroyl via the phenylpropanoids pathway. Therefore, from the perspective of “phenylpropanoids variation characteristics”, the elevation enhancement for the phenylpropanoids metabolites indirectly derived from Cinnamic acid-Coumaroyl via the phenylpropanoids pathway is more specific for the phenylpropanes and phenylmethanes, while the trend is reversed for phenylethanes. It should be also recalled here that the direct Cinnamic acid-Coumaroyl derivatives is less affected.

The number of phenylethanes differential metabolites was 5, 7 and 9 in RC-H vs. RC-L, RC-M vs. RC-L and RC-H vs. RC-M, reaching 10.64% of 47, 14.58% of 48 and 20.46% of 44 of the total number of phenylpropanoids differential metabolites, respectively. This indicates that in the range of 3933–4531 m, phenylethanes category was inferior to Cinnamic acid-Coumaroyl and derivatives and phenylmethanes. Therefore, it can be concluded that the effect of elevation increase in the range of 3933–4531 m on the biosynthesis of phenylpropanoids is still dominated by an enhanced tone. Combined with the quantitative perspective of biomarkers (recall that regarding upregulated biomarkers in RC-H vs. RC-L, RC-M vs. RC-L, and RC-H vs. RC-M, 9, 7 and 8 belong to phenylpropanoids while 2, 2 and 1 were flavonoids), the upregulated biomarkers of phenylpropanoids were found to be numerically more dominant in the elevation range of 3933–4531 m, suggesting that the enhancement of phenylpropanoids biosynthesis should be more dominant than flavonoids in this elevation range.

It is reported that phenylpropanoids enhance plant resistance to UV radiation, low temperature, drought, and pathogen [22,23,24], and it also has antioxidant, anti-inflammatory, and anti-diabetic effects as well as treating neuroinflammation-related diseases [100,101,102]. Combining these findings, we suggest that *Rhodiola crenulata*, grown at higher altitudes, may be of greater advantage for the beneficial effects of phenylpropanoids supplementation on human health.

**Figure 15:**
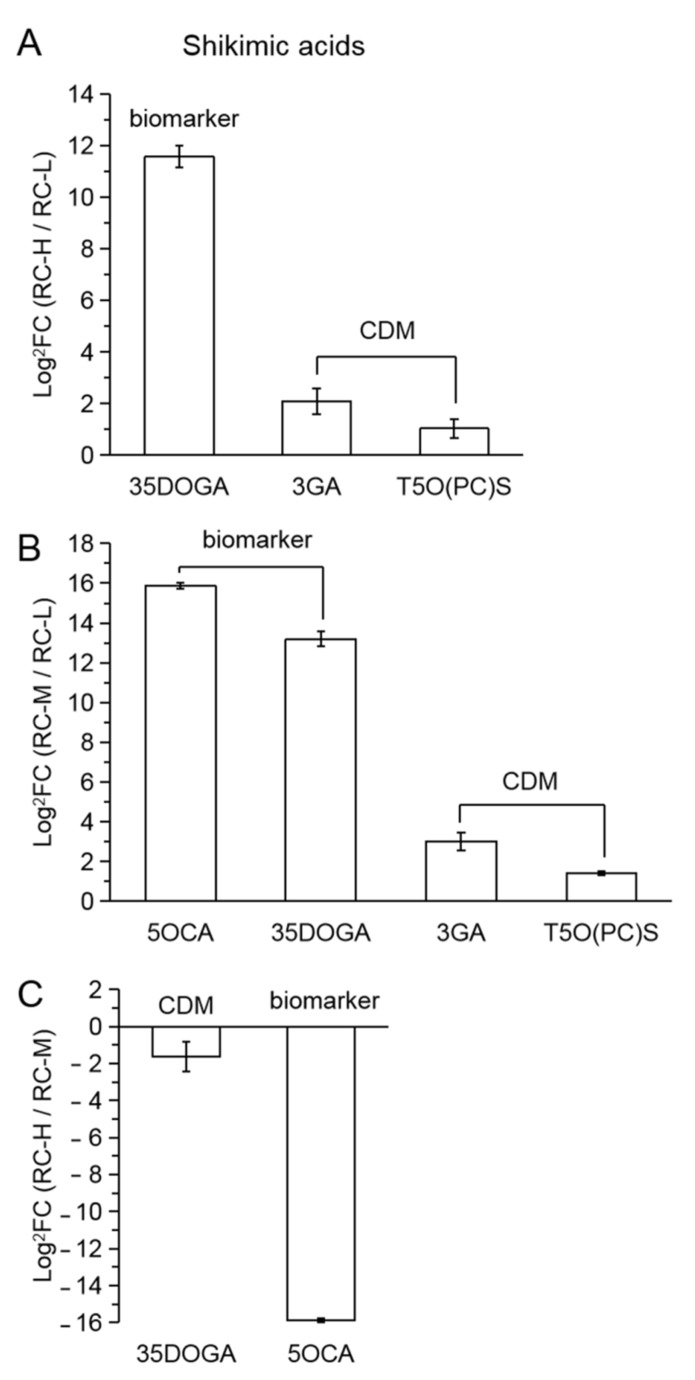
Comparison of differential metabolites of shikimic acids between RC-L, RC-M and RC-H. These were 1 biomarker and 2 CDMs for RC-H vs. RC-L (**A**), 2 biomarkers and 2 CDMs for RC-M vs. RC-L (**B**), and 1 biomarker and 1 CDM for RC-H vs. RC-M (**C**). Upregulation denotes that the content of a metabolite in RC increased with elevation between two altitudes, and the opposite for downregulation. The Y-axis is shown as Log^2^FC values for the convenience of visual presentation (Note: The up-amplitude in the text refers to the FC value). The X-axis denotes metabolite names.

In the comparisons of RC-H vs. RC-L, RC-M vs. RC-L and RC-H vs. RC-M, the number of upregulated and downregulated differential metabolites of shikimic acids were 3 vs. 0, 4 vs. 0 and 0 vs. 2, respectively (Figure 15A–C). In terms of biomarkers, there were one upregulated biomarker in RC-H vs. RC-L with FC value of 3020.26, and two upregulated biomarkers in RC-M vs. RC-L with FCs of 60,226.67 and 9381.78, respectively. These results indicate that shikimic acids show a significant upregulation with altitude, but it should be noted that RC-H vs. RC-M showed the opposite situation, which does not fully coincide with the overall upregulation.

Shikimic acid-phenylalanine is an upstream metabolic pathway of phenylpropanoids-flavonoids metabolism [35,36]. Our results for the overall upregulation of shikimic acids with increasing altitude in the altitude range of 3933–4531 m support the finding that the phenylpropanoids-flavonoids biosynthesis were enhanced with increasing altitude. Meanwhile, the results of downregulation of shikimic acids in RC-H vs. RC-M coincide with the changes of quercetin and kaempferol and their derivatives in RC-H vs. RC-M. This suggests that the weakening trend of quercetins and kaempferols biosynthesis in elevation from 4244–4531 m may be related to the downregulation of shikimic acids in RC-H vs. RC-M.

We argue that the enhanced upregulation of the shikimic acid-phenylpropanoids-flavonoids metabolic pathway is highly correlated with the overall intensification of stresses from UV radiation, low temperature, and hypoxia due to elevation.

4. Amino Acids

**Figure 16:**
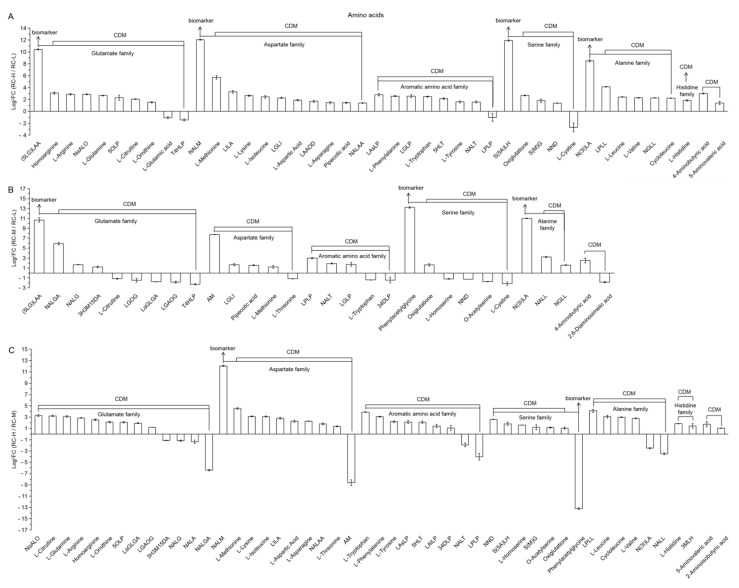
Comparison of differential metabolites of amino acids between RC-L, RC-M and RC-H. These were 4 biomarkers and 39 CDMs for RC-H vs. RC-L (**A**), 3 biomarkers and 27 CDMs for RC-M vs. RC-L (**B**), and 2 biomarkers and 47 CDMs for RC-H vs. RC-M (**C**). Upregulation denotes that the content of a metabolite in RC increased with elevation between two altitudes, and the opposite for downregulation. The Y-axis is shown as Log^2^FC values for the convenience of visual presentation (Note: The up-amplitude in the text refers to the FC value). The X-axis denotes metabolite names.

The number of upregulated and downregulated amino acid and derivative-like differential metabolites in the comparisons of RC-H vs. RC-L, RC-M vs. RC-L and RC-H vs. RC-M were 39 vs. 4; 17 vs. 13 and 39 vs. 10, respectively (Figure 16A–C). In terms of biomarkers, there are four and three upregulated biomarkers, respectively; while in RC-H vs. RC-M, there is one upregulation and one downregulation. These results suggest that amino acid and derivative classes show a significant upregulation with increase of elevation.

In terms of the extent to which the six families of amino acid were affected by elevation, the relationship between the number of differential metabolites per family (RC-H vs. RC-L, RC-M vs. RC-L, and RC-H vs. RC-M) and the total number of differential metabolites of amino acids is: glutamic acid family (23.26–30%) > aspartic acid family (16.67–25.58%) > aromatic family (16.67–18.61%) > serine family (11.63–20%) > alanine family (10–13.95%) > histidine family (0–4.08%).

For the glutamate family, glutamate and glutamine were its signature amino acids. The glutamate family shows 3 (2 upregulated (i.e., (5-L-Glutamyl)-L-amino acid as biomarker and L-Glutamine) and 1 downregulated for L-Glutamic acid), 6 (3 upregulated (i.e., (5-L-Glutamyl)-L-amino acid as biomarker, *N*-Acetyl-L-glutamic acid and *N*-Acetyl-L-Glutamine) and 3 downregulated (i.e., L-Glutamine-O-glycoside, L-α-Glutamyl-L-Glutamic Acid and L-Glutamic acid-O-glycoside)) and 5 (3 upregulated (i.e., L-Glutamine, L-α-Glutamyl-L-Glutamic Acid and L-Glutamic acid-O-glycoside) and 2 downregulated (i.e., *N*-Acetyl-L-Glutamine and *N*-Acetyl-L-glutamic acid)) in RC-H vs. RC-L, RC-M vs. RC-L and RC-H vs. RC-M, respectively, accounting for 6.98% of 43, 20.00% of 30 and 10.20% of 49 of the total number of amino acid-like differential metabolites. They are transamination pools for the synthesis of many other amino acids in plants [103,104]. Our results found a more active metabolism with altitude. This result favourably supports the finding that the amino acid biosynthetic pathway is enhanced with increase of elevation.

The phenylalanines in the aromatic family are important intermediate metabolites in the shikimic acid-phenylalanine-phenylpropanoids-flavonoids biosynthetic pathway. We found their number of differential metabolites in the comparisons of RC-H vs. RC-L, RC-M vs. RC-L and RC-H vs. RC-M to be 4 (3 upregulated vs. 1 downregulated, 75% upregulated, i.e., L-Aspartyl-L-Phenylalanine, L-Phenylalanine and L-Glycyl-L-phenylalanine to upregulation; L-Prolyl-L-Phenylalanine to downregulation), 3 (2 upregulated vs. 1 downregulated, 66.67% upregulated, i.e., L-Prolyl-L-Phenylalanine and L-Glycyl-L-phenylalanine to upregulation; 3,4-Dihydroxy-L-phenylalanine to downregulation) and 5 (4 upregulated vs. 1 downregulated, 80% upregulated, i.e., L-Phenylalanine, L-Aspartyl-L-Phenylalanine, L-Alanyl-L-Phenylalanine and 3,4-Dihydroxy-L-phenylalanine to upregulation; L-Prolyl-L-Phenylalanine to downregulation), respectively.

It accounts for 9.30% of 43, 10.00% of 30 and 10.20% of 49 of the total differential metabolites of amino acids, respectively. This indicates that it is more active in amino acid biosynthesis in response to altitude change, and its response to altitude change shows significant upregulation in terms of upregulation ratio. This finding, combined with the aforementioned results for shikimic acids, phenylpropanoids and flavonoids in response to altitude change, clearly outlines the complete metabolic pathway for the enhanced biosynthesis of shikimic acid-phenylalanine-phenylpropanoids-flavonoids biosynthesis pathway with increasing altitude, suggesting that *Rhodiola crenulata* synthesizes various phenylpropanoids and flavonoids through this pathway to systematically enhance the resistance to altitude stress.

Furthermore, among the six amino acid families with differential amino acids changing with altitude, various amino acids such as glutathione, tyrosine, arginine, cysteine, proline, ornithine, glycine, aspartate, lysine, isoleucine, and leucine enhance plant stress tolerance in multiple ways [105,106,107,108,109,110,111,112,113,114,115]; while valine and tryptophan are associated with promoting plant growth [116,117]. These differential amino acid changes suggest that *Rhodiola crenulata* may involve multiple forms of adaptive changes with altered growth patterns in response to altitude changes.

It is also important to note that 4-Aminobutyric acid was upregulated in RC-H vs. RC-L (8.04-fold change) and RC-M vs. RC-L (5.75-fold change), while 2-Aminoisobutyric acid showed upregulation in RC-H vs. RC-M (2.07-fold change). This indicates that Aminobutyric acid is upregulated with elevation. It was found that aminobutyric acid is strongly associated with enhanced plant energy metabolism [34] and multiple stresses and defense systems [118,119].

5. Free fatty acids and glycerides

**Figure 17:**
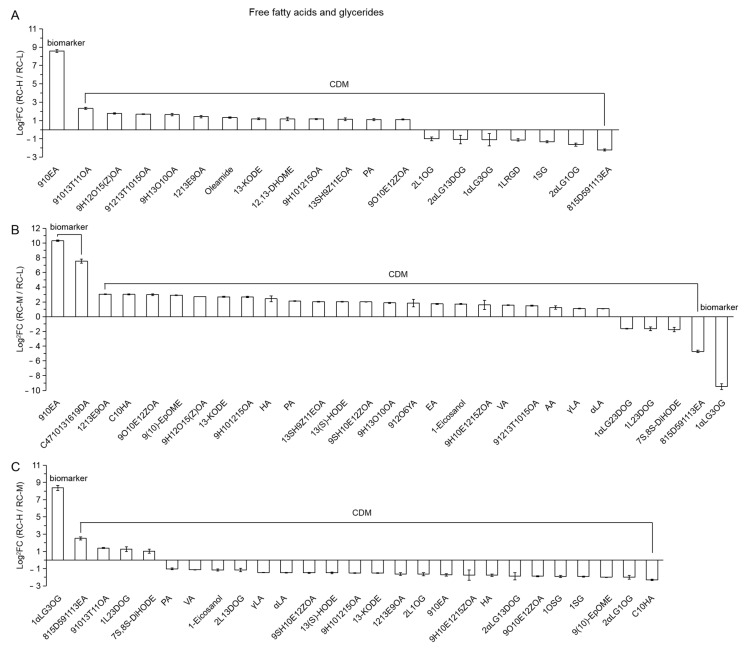
Comparison of differential metabolites of Free fatty acids and glycerides between RC-L, RC-M and RC-H. These were 1 biomarker and 19 CDMs for RC-H vs. RC-L (**A**), 3 biomarkers and 26 CDMs for RC-M vs. RC-L (**B**), and 1 biomarker and 26 CDMs for RC-H vs. RC-M (**C**). Upregulation denotes that the content of a metabolite in RC increased with elevation between two altitudes, and the opposite for downregulation. The Y-axis is shown as Log^2^FC values for the convenience of visual presentation (Note: The up-amplitude in the text refers to the FC value). The X-axis denotes metabolite names.

In RC-H vs. RC-L and RC-M vs. RC-L, 20 (1 biomarker and 19 CDMs) and 29 (3 biomarkers and 26 CDMs) differential metabolites were classified into this category, respectively. They are presented in Figure 17A,B in descending order from left to right on the basis of FC value size. As shown in the figure, the numbers of up- and downregulation were 13 vs. 7 and 24 vs. 5, respectively, indicating that the elevation increase from 3933–4531 m and from 3933–4249 m significantly enhanced the biosynthesis of free fatty acids and glycerides.

Further analysis from the chemical structure perspective revealed that all 13 upregulated CDMs in RC-H vs. RC-L were composed of free fatty acids, while the number of free fatty acids and glycerides was 1 vs. 6 in the 7 downregulated ones; among the 24 upregulated differential metabolites in RC-M vs. RC-L, the number of free fatty acids and glycerides is 24 vs. 0; while the number of free fatty acids and glycerides in the 5 downregulations was 2 vs. 3. These results suggest that, when the elevation is increased from 3933–4531 m and from 3933–4249 m, free fatty acids were significantly upregulated, while glycerides were significantly downregulated. This indicates that the changes in free fatty acids and glycerides with altitude gradient in *Rhodiola crenulata* may be based on the breakdown of glycerides and their conversion into free fatty acids. It is important to note that this pattern was not evident in RC-H vs. RC-M, i.e., elevation from 4249–4531 m.

Kuczyńska et al. found an increase in free fatty acid content and a significant decrease in triglyceride content in barley (*Hordeum vulgare* L.) treated with abiotic stresses such as salt stress [120]. This result is in agreement with our findings. Glycerides are the major forms of lipid storage in organisms, while free fatty acids serve as the main form of lipid use that provides an essential energy source for the life activities of plants [55,56,121].

In the plant body, lipases are responsible for the breakdown of triglycerides to glycerol and fatty acids [122]. Therefore, it can be concluded that *Rhodiola crenulata* provides more source of energy to resist high altitude adversity stress by breaking down glycerides and accumulating free fatty acids at higher altitudes, thus improving the resistibility against high altitude integrated adversity.

6. Nucleotides

**Figure 18:**
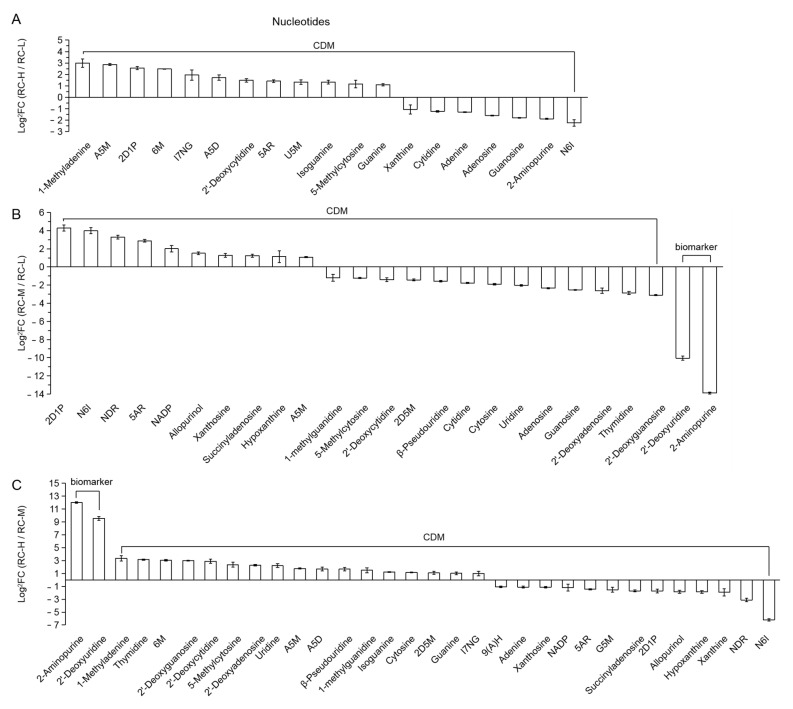
Comparison of differential metabolites of nucleotides between RC-L, RC-M and RC-H. These were 19 CDMs for RC-H vs. RC-L (**A**), 2 biomarkers and 23 CDMs for RC-M vs. RC-L (**B**), and 2 biomarkers and 30 CDMs for RC-H vs. RC-M (**C**). Upregulation denotes that the content of a metabolite in RC increased with elevation between two altitudes, and the opposite for downregulation The Y-axis is shown as Log^2^FC values for the convenience of visual presentation (Note: The up-amplitude in the text refers to the FC value). The X-axis denotes metabolite names.

Among RC-H vs. RC-L, RC-M vs. RC-L and RC-H vs. RC-M, 19 CDMs, 25 (2 biomarkers and 23 CDMs) and 32 (2 biomarkers and 30 CDMs) differential metabolites are classified in this category, respectively. They are presented in a descending order from left to right in Figure 18A–C according to the size of FC values. As can be seen from the figure, the number of upregulated and downregulated is 12 vs. 7, 10 vs. 15 and 19 vs. 13, respectively. This indicates that nucleotide biosynthesis was significantly enhanced when the altitude increased from 3933–4531 m and from 4249–4531 m, while it was attenuated when the altitude increased from 3933–4249 m. Considering the changes in altitude from 3933–4531 m, we concluded that the altitude increase in general contributed to the enhanced nucleotide synthesis in *Rhodiola crenulata* rhizome.

From a further analysis of the chemical structure, we found that the number ratio of up- and downregulated adenosine phosphates involved in ATP energy metabolism was 3 vs. 0, respectively (3 upregulated for Adenosine 5′-monophosphate, Adenosine 5′-diphosphate and Uridine 5′-monophosphate, respectively) for RC-H vs. RC-L; 2 vs. 0 (2 upregulated to NADP and Adenosine 5′-monophosphate) for RC-M vs. RC-L and 2 vs. 1 (2 upregulated to Adenosine 5′-monophosphate and Adenosine 5′-diphosphate, and 1 downregulated to NADP) for RC-H vs. RC-M. When converted to a percentage of nucleotide up- and downregulated differential metabolites, it was 25 vs. 0% for RC-H vs. RC-L; 20 vs. 0% for RC-M vs. RC-L; 10.53 vs. 7.69% for RC-H vs. RC-M (Figure 18A–C). These results indicate that adenosine phosphates exhibit upregulation as the elevation increase ranging from 3933–4531 m. In addition, adenosine triphosphate (ATP) was also upregulated in RC-H vs. RC-L (1.37-fold change) and RC-M vs. RC-L (2.60-fold change).

It was shown that Adenosine 5′-monophosphate, Adenosine 5′-diphosphate, Uridine 5′-monophosphate and ATP are directly related to energy metabolism [57,123].

Based on these findings, we suggest that elevation in the range of 3933–4531 m may enhance the adaptive capacity of *Rhodiola crenulata* against high altitude adversity by (1) upregulating nucleotide biosynthesis to promote more vigorous metabolism of genetic material and (2) supplying more abundant energy by upregulating adenosine phosphates, which is involved in ATP energy metabolism.

It should also be noted that, in conjunction with the findings of Figure 17, elevated altitude induced the upregulation of free fatty acids and downregulation of glycerides, thus providing energy to resist high altitude adversity stress. These findings provide a new insight into the resistance of *Rhodiola crenulata* to high altitude integrated adversity from an energetic perspective.

## 3. Materials and Methods

### 3.1. Plant Materials and Extracts Preparation

All *Rhodiola crenulata* samples were obtained from Dakazi Mountain, Ganzi Tibetan Autonomous Region, Sichuan Province, China, in October of 2019. According to altitudes, the samples were divided into three groups: RC-L (altitude of 3933 m; 101°53′32″ E, 28°56′4″ N), RC-M (altitude of 4249 m; 101°52′47″ E, 28°55′10″ N) and RC-H (altitude of 4531 m; 101°53′3″ E, 28°55′22″ N).

The collection sites could be characterized as a plateau type monsoon area with a alpine meadow soil. The average temperature of collection sites ranging from low to high altitude is 0.8–−4.6 °C. The temperature would decrease by 0.90 °C as elevation increased per 100 m. No absolute frost-free period exists throughout the year with a precipitation 636 mm/y and average annual sunshine of 1900–2600 h on the region. The annual light radiation is about 120–160 KCal/cm^2^. The UV radiation intensity at the collection sites at noon was 1558 (3933 m), 1857 (4249 m), 2486 (4531 m) uw/cm^2^.

For each group, three wild-grown *Rhodiola crenulata* plants were collected. All plants were authenticated as *Rhodiola* *crenulata* (Hook.f. & Thomson) H.Ohba by Dr. Shubin Dong at the Beijing Forestry University, and were freeze-dried and stored at −40 °C.

For each group, the same weights were taken from rhizomes of three plants, then ground and mixed uniformly as one rhodiola sample for subsequent experiments.

In the procedure described in Section 3.3 and Section 3.4, extract preparation was based on the methods proposed by Dong et al. and Zhang et al. [14,124]. Briefly, 1.5 g of rhodiola samples were mixed with 15 mL of 70% methanol. Then they are sonicated in a 300 W water bath (KQ-300DE ultrasonic cleaner, Kunshan Ultrasonic Instrument Co., LTD., Kunshan, China) for 30 min at room temperature with occasional stirring. The supernatant was collected after filtration by a 0.22 µm filter. This extraction process was repeated twice more with the residue. Afterwards, these three supernatants were pooled together, and then stored at −20 °C.

### 3.2. Determination of Oxidative States and Ascorbic Acids

On the basis of the protocols provided in corresponding Content Determination Kits (Solarbio Life Sciences, Beijing, China), the contents of H_2_O_2,_ Protein carbonyl, O_2_^·−^, MDA, AsA (ascorbic acid), and DHA (dehydroascorbate) were determined using the colorimetric method. The contents of total ascorbic acids were calculated by the sum of AsA and DHA.

### 3.3. Determination of Phenolic Components

#### 3.3.1. Total Phenols

Following the Folin-Ciocalteau method proposed by Sun et al. [125], the total phenols were determined. In brief, 20 μL extracts, standards (10–400 mg/L gallic acid) or blank (distilled water) were mixed with 40 μL of 25% Folin-Ciocalteu, respectively, and then 140 μL of 700 mM Na_2_CO_3_ were added and shaken at 250 rpm for 5 min. After incubation for 2 h at room temperature in the dark, absorbances were measured at 765 nm using a microplate reader (Tecan Infinite 200 Pro Full Wavelength Microplate Reader, Tecan, Männedorf, Switzerland). The results were expressed as mg gallic acid/100 g d.w. of *Rhodiola crenulata* rhizomes.

#### 3.3.2. Total Tannins

Total tannins were determined by casein-precipitation reaction based on the method proposed by Dong et al. [14]. In a nutshell, 8 g casein was added into 10 mL of the extract, and then the mixture was kept shaking for 3 h at 200 rpm. The mixture was subsequently placed in a 0.22-μm filter to obtain filtrate. The phenolic acid in filtrate was determined by the method described in Section 3.3.1. A standard curve of gallic acid (10–400 mg/L) was established. Total tannins were calculated by the difference of phenols before and after casein-precipitation reaction.

#### 3.3.3. Total Flavonoids

Total flavonoids were determined by the aluminum chloride colorimetric assay according to the approach developed by Dong et al. [14]. Briefly, 140 μL extracts, standards (10–100 mg/L rutin) or blank (distilled water) were mixed with 8 μL of 50 mg/mL NaNO_2_ for 6 min, respectively, followed by adding 8 μL of 100 mg/mL AlCl_3_. After a 5-min standing, 100 μL of 40 mg/mL NaOH were added and incubated at room temperature for 30 min. Absorbances were measured at 410 nm with the microplate reader. The results were expressed as mg rutin/100 g d.w. of *Rhodiola crenulata* rhizomes.

#### 3.3.4. Condensed Tannins

Condensed tannins were detected using a vanillin assay as described by Fan et al. [126]. In short, 20 μL extracts, standards (50–800 mg/L catechin) or blank (distilled water) were mixed with 120 μL of 4% vanillin-methanol solution, respectively, and then 60 μL of hydrochloric acid were added and incubated at room temperature for 15 min. Subsequently, absorbances were measured at 500 nm with the microplate reader. The results were expressed as mg catechin/100 g d.w. of *Rhodiola crenulata* rhizomes.

### 3.4. Determination of Antioxidant Capacity

#### 3.4.1. DPPH-Scavenging Activity

DPPH-scavenging activity was evaluated based on the method described in Dong et al. [14]. In brief, 10 μL extracts, standards (20–800 mg/L Trolox) or blank (distilled water) were mixed with 40 μL of 1mM DPPH, respectively, and then 190 μL methanol were added. After incubation for 30 min at room temperature in the dark, absorbances were measured at 517 nm using the microplate reader. Results were expressed as mg Trolox/100 g d.w. of *Rhodiola crenulata* rhizomes.

#### 3.4.2. ABTS^+^-Scavenging Activity

ABTS^+^-scavenging activity was determined according to the method proposed in Dong et al. [14]. In short, ABTS solutions were prepared by mixing an equal volume of 7 mM ABTS and 2.4 mM potassium persulfate and incubated in dark at room temperature for 12–16 h. After incubation, the ABTS solution was diluted with methanol to an absorbance of 0.7 ± 0.02 at 734 nm to obtain ABTS working solution. Subsequently, 5 μL of extracts, standards (20–800 mg/L Trolox) or blank (distilled water) were mixed with 200 μL ABTS working solution, respectively. After incubation in dark at 30 °C for 5 min, absorbances were measured at 734 nm using the microplate reader. The results were expressed as mg Trolox/100 g d.w. of *Rhodiola crenulata* rhizomes.

### 3.5. Mineral Content Analysis

The contents of seven plant mineral elements in *Rhodiola crenulata* rhizomes were detected based on the method described in National Standard [127], including Ca, Sr, B, Mn, Ni, Cu and Cd (*n* = 4). Briefly, 0.2 g of rhodiola sample was mixed with 3 mL nitric acid and 2 mL hydrochloric acid in a digestion tube, which was placed in a microwave digestion apparatus. The microwave digestion was performed according to the following gradient program: 0 min: room temperature; 0-5 min: linear increase to 130 °C; 5–13 min: 130 °C was maintained; 13–18 min: linear increase to 160 °C; 18–26 min: 160 °C was kept; 26–31 min: linear increase to 195 °C; 31–61 min: 195 °C was maintained; 61–62 min: linear decrease to 40 °C. After digestion, the volume of the mixture was fixed to 50 mL with distilled water to obtain a test solution, and a blank solution was prepared in parallel.

Ca, Sr, B, Mn, Ni, Cu and Cd were detected by inductively coupled plasma mass spectrometry (7850 ICP-MS, Agilent, Santa Clara, CA, USA). All elements were quantified by standard curves. The ICP-MS operation parameters were set as follows: RF power: 1600 W; plasma flow: 14 L/min; auxiliary flow: 0.8 L/min; atomizing gas flow: 0.97 L/min; helium flow: 3 mL/min; sampling depth: 5 mm; analog voltage: −1820 V; pulse voltage: 887 V; acquisition time: 3 s; spray chamber temperature: 2.7 °C.

### 3.6. LC-ESI-MS/MS

Rhodiola sample was prepared according to the method introduced in Section 3.1. To measure the assay’s reproducibility, a QC (quality control) sample was obtained by mixing RC-L, RC-M, and RC-H.

Metabolite extraction and LC-ESI-MS/MS analysis were based on the method described in Fan et al. [128]. In brief, 50 mg of rhodiola sample was mixd with 700 μL of extract solution (methanol:water = 3:1, precooled at −40 °C). Subsequently, the mixture was homogenized at 35 Hz for 4 min and sonicated in an ice-water bath for 5 min. The homogenization and sonication cycle were repeated twice. The mixture was then further extracted overnight at 4 °C on a shaker. Following centrifugation at 13,800× *g* for 15 min at 4 °C, the extract was filtrated through a 0.22 μm microporous membrane to obtain a filtrate.

The filtrates were analyzed by a LC-ESI-MS/MS system (HPLC, EXION LC system, Sciex, Framingham, MA, USA; MS, SCIEX QTrap 6500+, Sciex, Framingham, MA, USA). Extracts were separated through a Waters ACQUITY UPLC HSS T3 C18 (1.8 μm, 2.1 × 100 mm, Waters, Milford, MA, USA) as the column under following mobile phase conditions: mobile phase A was 0.1% formic acid in water, and mobile phase B was acetonitrile. The measurements were performed using the following gradient program: 0–0.5 min: 98% A, 2% B; 0.5–11 min: a linear gradient to 5% A, 95% B; 11–13 min: 5% A plus 95% B was kept; a combination of 98% A with 2% B was set within 0.10 min and kept running for 13.1–15 min. The column temperature was set at 40 °C. The auto-sampler temperature was set at 4 °C and the injection volume was 2 μL. The effluent was connected to an ESI-triple quadrupole-linear ion trap (QqQ-LIT)-MS.

The separated components were detected using a triple quadrupole (QqQ)-linear ion trap (LIT) mass spectrometer integrated with an IonDrive Turbo V ESI interface (SCIEX QTrap 6500+, Sciex, Framingham, MA, USA) and operated in positive and negative ion modes. The ESI source operation parameters were as follows: ion spray voltage: +5500/−4500 V; curtain gas: 35 psi; temperature: 400 °C; ion source gas 1:60 psi; ion source gas 2:60 psi; declustering potential (DP): ±100 V. Instrument tuning and mass calibration were performed in QqQ and LIT modes with polyethylene glycol solutions of 10 μmol/L and 100 μmol/L, respectively. The QqQ scan was performed by multiple reaction monitoring (MRM) with the collision gas (nitrogen) set to 5 psi. The DP and CE of single MRM transfer were achieved by further optimization of declustering potential (DP) and collision energy (CE). Based on metabolites eluted during this period, a specific set of MRM transition was monitored in each cycle.

### 3.7. Statistical Analysis

Multivariate analysis was performed using the SIMCA16.0.2 software package (Sartorius Stedim Data Analytics AB, Umea, Sweden), including principal component analysis (PCA), orthogonal partial least squares discriminant analysis (OPLS-DA), hierarchical clustering analysis (HCA) and heat map construction. PCA is an unsupervised approach that reduces the dimensionality of the data and visualizes the sample distribution and grouping. 95% confidence interval in the PCA score plot was used as a threshold to identify potential outliers in dataset. The data were processed by HCA to create a heatmap. Based on OPLS-DA, differential metabolites were evaluated by the values of variable importance in the projection (VIP), fold change (FC) and p-value, with the criteria of simultaneously meeting VIP > 1, |log^2^(fold change)| ≥ 1, and *p*-value < 0.05. Biomarkers were identified by the high abundance of a metabolite in a *Rhodiola* sample versus zero abundance (set by 9.00 cps) of the corresponding metabolite in another *Rhodiola* sample.

## 4. Conclusions

In this paper, compared with ascorbic acid, the upregulation of phenolic components was more significant, along with the increased antioxidant capacities, in the response of *Rhodiola crenulata* to the altitude gradient, which may be the responses of *Rhodiola crenulata* to altitude-caused stresses.

It is also important to note regarding to the accumulation of six metabolites classes including phenolic components, and seven mineral elements related to stress resistance, this study proposes a novel approach to identify the elevation in the same mountain from 3933–4531 m leads to the following results: (1) overall upregulation of the top-down synthetic pathway of shikimic acid-phenylalanine-phenylpropanoids-flavonoids; (2) the number of upregulated biomarkers suggested that the upregulation of phenylpropanoids was more significant than that of flavonoids; (3) phenylpropanoids indirectly derived from Cinnamic acid-Coumaroyl via the phenylpropanoid pathway were more significantly affected, with phenylpropanes and phenylmethanes having a significant impact on the accumulation of phenylpropanoids; (4) the upregulation of quercetin and its derivatives was the most significant among the flavonoids; (5) the upregulation of condensed tannins and the downregulation of hydrolyzed tannins; (6) the significant upregulation of amino acids; (7) the overall increase in the accumulation of seven mineral elements related to resistance; (8) free fatty acids were significantly upregulated and glycerides were downregulated; and (9) nucleotides were generally upregulated, with adenosine phosphates upregulated.

The above findings uncovered how *Rhodiola crenulata* upregulates phenolic components by enhancing the shikimic acid-phenylalanine-phenylpropanoids-flavonoids pathway; changes free fatty acids and glycerides, and nucleotides; and promotes the accumulation of seven mineral elements associated with resilience under extreme high altitude adversity. These findings provide new insights and new directions for the responses of *Rhodiola crenulata* to extreme high altitude adversities, and also offer an important basis for the in-depth development of the medicinal components of *Rhodiola crenulata*. More importantly, present study provided a rich and solid scientific support for further conducting research with different subjects.

## Figures and Tables

**Table 1:** Antioxidant activities for rhizomes of *Rhodiola crenulata* from different altitudes.

Samples	IC_50_ (μg/mL) DPPH	IC_50_ (μg/mL) ABTS
RC-L	29.05	34.37
RC-M	20.06	24.16
RC-H	16.39	16.61

Note: RC-L, RC-M, and RC-H denotes rhizomes of *Rhodiola crenulata* from different altitudes (3933, 4249 and 4531 m), respectively.

**Table 2:** Biomarkers for *Rhodiola crenulata* from altitude of 3933–4531 m (RC-H vs. RC-L; RC-M vs. RC-L; RC-H vs. RC-M).

Metabolite	Category	Fold Change (RC-H vs. RC-L)	Type	Fold Change (RC-M vs. RC-L)	Type	Fold Change (RC-H vs. RC-M)	Type
Quercetin-3-O-rutinoside (Rutin)	Flavonoids	50,814.82	Up	60,233.70	Up	N/A	N/A
1-Methylpiperidine-2-carboxylic acid	Others	N/A	N/A	63,187.78	Up	1.58258E-05	Down
5-O-Caffeoylshikimic acid	Phenylpropanoids	N/A	N/A	60,226.67	Up	1.66039E-05	Down
Rhododendrol	Phenylpropanoids	25,852.22	Up	25,319.26	Up	N/A	N/A
7-O-Methxyl Quercetin (Rhamnetin)	Flavonoids	13,547.41	Up	130,288.89	Up	N/A	N/A
LysoPC 19:2	Others	N/A	N/A	15,415.56	Up	6.48695E-05	Down
Phenylacetylglycine	Amino acids	N/A	N/A	9512.74	Up	0.00011	Down
2,3,4,5,6-pentahydroxyhexyl 2-hydroxybenzoate	Phenylpropanoids	6782.52	Up	4217.04	Up	N/A	N/A
Catalposide	Others	N/A	N/A	3510.22	Up	0.00029	Down
*N*-Acetyl-D-glucosamine-1-phosphate	Others	6525.93	Up	5938.93	Up	N/A	N/A
LysoPC 20:1	Others	N/A	N/A	5650.63	Up	0.00018	Down
3-(3-Hydroxyphenyl)-propionate acid	Phenylpropanoids	N/A	N/A	4544.74	Up	0.00022	Down
N-Acetyl-L-methionine	Amino acids	4257.63	Up	N/A	N/A	4257.63	Up
S-(5′-Adenosy)-L-homocysteine	Amino acids	3839.48	Up	N/A	N/A	N/A	N/A
Tryptamine	Others	3096.93	Up	20,954.07	Up	N/A	N/A
3,5-Di-O-galloylshikimic acid	Phenylpropanoids	3020.26	Up	9381.78	Up	N/A	N/A
Caffeoylbenzoyltartaric acid	Phenylpropanoids	1654.26	Up	N/A	N/A	1654.26	Up
1,2-O-Diferuloylglycerol	Phenylpropanoids	1494.74	Up	N/A	N/A	1494.74	Up
3-Aminosalicylic acid	Others	N/A	N/A	N/A	N/A	1415.11	Up
(5-L-Glutamyl)-L-amino acid	Amino acids	1368.89	Up	1649.89	Up	N/A	N/A
LysoPC 18:4	Others	N/A	N/A	1302.52	Up	0.0008	Down
Phthalic acid	Others	1038.84	Up	1834.74	Up	N/A	N/A
Chlorogenic acid methyl ester	Phenylpropanoids	868.57	Up	N/A	N/A	868.57	Up
Sinapoyl malate	Phenylpropanoids	565.92	Up	3459.89	Up	N/A	N/A
Scopoletin-7-O-glucoside (Scopolin)	Phenylpropanoids	N/A	N/A	2996.11	Up	0.0003	Down
4-Hydroxybenzyl Alcohol	Phenylpropanoids	497.65	Up	N/A	N/A	497.65	Up
9,10-Epoxyoctadecanoic Acid	Free fatty acids and glycerides	381.88	Up	1260.63	Up	N/A	N/A
Indole 3-acetic acid (IAA)	Others	N/A	N/A	716.29	Up	0.0014	Down
Cis-4,7,10,13,16,19-Docosahexaenoic Acid	Free fatty acids and glycerides	N/A	N/A	185.51	Up	N/A	N/A
5,7-Dimethoxycoumarin	Phenylpropanoids	N/A	N/A	0.0028	Down	445.73	Up
Isoeugenol	Phenylpropanoids	N/A	N/A	0.0017	Down	345.22	Up
1-α-Linolenoyl-glycerol-3-O-glucoside	Free fatty acids and glycerides	N/A	N/A	0.0014	Down	330.92	Up
3-Indolepropionic acid	Others	N/A	N/A	0.0014	Down	3134.78	Up
3-O-p-Coumaroylquinic acid	Phenylpropanoids	N/A	N/A	0.0012	Down	1371.50	Up
2′-Deoxyuridine	Nucleotides	N/A	N/A	0.0009	Down	748.40	Up
*N*-(3-Indolylacetyl)-L-alanine	Amino acids	365.44	Up	2071.70	Up	N/A	N/A
Methyl caffeate	Phenylpropanoids	147.26	Up	N/A	N/A	147.26	Up
Clove chromone	Others	0.00085	Down	0.0009	Down	N/A	N/A
LysoPE 15:0(2n isomer)	Others	0.00075	Down	0.0008	Down	N/A	N/A
4-Pyridoxic acid-O-glucoside	Others	N/A	N/A	0.0006	Down	931.35	Up
Quercetin-3-O-(6′’-galloyl)galactoside	Flavonoids	N/A	N/A	0.0001	Down	7396.52	Up
2-Aminopurine	Nucleotides	N/A	N/A	6.617E-05	Down	4106.22	Up
1,6-Di-O-Galloyl-D-Glucose	Gallic acid derivatives	N/A	N/A	1.17381E-05	Down	131,281.48	Up
LysoPC 15:0	Others	0.00059	Down	N/A	N/A	0.00028	Down

Note: 1. Upregulation denotes that the content of a metabolite in RC exhibited a significant increase compared to that of corresponding low altitude. Conversely, downregulation indicates the content of a metabolite in RC is significantly decreased compared with the one in that of corresponding low altitude; 2. Biomarkers were identified by the high abundance of certain metabolites in a Rhodiola sample versus zero abundance (set by 9.00 cps) of the corresponding metabolite in another Rhodiola sample; 3. N/A means Not Applicable.

## Data Availability

Data are available from the authors.

## Supplementary Materials

The following are available online: Table S1: List of identified metabolites between quality control, RC-L, RC-M, and RC-H; Table S2: List of differential metabolites between RC-H vs. RC-L; Table S3: List of differential metabolites between RC-M vs. RC-L; Table S4: List of differential metabolites between RC-H vs. RC-M; Figure S1: Heat map for a total of 1165 metabolites in RC-L, RC-M, and RC-H; Figure S2: OPLS-DA permutation plots; Figure S3: Heat map for recognized differential metabolites in RC-L, RC-M, and RC-H.

## Sample Availability

Samples of the *Rhodiola* are available from the authors.

## Abbreviations

1 (Figure 7)	Q3ORu	quercetin-3-O-rutinoside (Rutin)
2	7OMQ	7-O-methxyl quercetin (Rhamnetin)
3	Q7OR	quercetin-7-O-rutinoside
4	Q3βDS	quercetin 3-β-D-sophoroside
5	Q3O(2OG)G	quercetin-3-O-(2′’-O-galactosyl)glucoside
6	Q3OGl	quercetin-3-O-glucoside (Isoquercitrin)
7	Q3OS	quercetin-3-O-sophoroside (Baimaside)
8	Q3OG(1→4)R7OR	quercetin-3-O-glucosyl(1 → 4)rhamnoside-7-O-rutinoside
9	Q3O(2OR)R	quercetin-3-O-(2′’-O-Rhamnosyl)rutinoside
10	Q3O(6OA)G	quercetin-3-O-(6′’-O-arabinosyl)glucoside
11	Q3O(2OG)glucuronide	quercetin-3-O-(2′’-O-glucosyl)glucuronide
12	5OM	5-O-methylquercetin (Azaleatin)
13	I3OR	isorhamnetin-3-O-rutinoside
14	R3OR	rhamnetin-3-O-rutinoside
15	Q7OβDG	quercetin 7-O-β-D-glucoside
16	Q3OGa	quercetin-3-O-galactoside (Hyperin)
17	Q3OR7OG	quercetin-3-O-rutinoside-7-O-glucoside
18	Q3OX	quercetin-3-O-xyloside (Reynoutrin)
19	Q3ORo	quercetin-3-O-robinobioside
20	Q3O(6G)G	quercetin-3-O-(6′’-galloyl)galactoside
21 (Figure 8)	D7OG	dihydrokaempferol-7-O-glucoside
22	6CM3G	6-C-methylkaempferol-3-glucoside
23	D3OG	dihydrokaempferol-3-O-glucoside
24	6H36OD	6-hydroxykaempferol-3,6-O-diglucoside
25	K68DCG7OG	kaempferol-6,8-di-C-glucoside-7-O-glucoside
26	K4OG	kaempferol-4′-O-glucoside
27	K3OGa	kaempferol-3-O-galactoside (Trifolin)
28	K3ON	kaempferol-3-O-neohesperidoside
29 (Figure 9)	(−)−E3(3OM)G	(−)-epicatechin-3-(3′’-O-methyl)gallate
30	PC13OG	procyanidin C1 3′-O-gallate
31 (Figure 10)	L3OG	luteolin-3′-O-glucoside
32	L7O(2OR)R	luteolin-7-O-(2′’-O-rhamnosyl)rutinoside
33 (Figure 11)	13TA	1,3-trigallic acid
34	3H5M1O(6D)G	3-hydroxy-5-methylphenol-1-O-(6′-digalloyl) glucoside
35	3OMA	3-O-methylgallic acid
36	17DOGDS	1,7-di-O-galloyl-D-sedoheptulose
37	M6OGG	methyl 6-O-galloyl-glucoside
38	2OS6OGDG	2-O-salicyl-6-O-galloyl-D-glucose
39	MD	monogalloyl-diglucose
40	14TA	1,4-trigallic acid
41	23OD146TOGG	2,3-O-digalloyl-1,4,6-tri-O-galloyl-glucose
42	1236TOGDG	1,2,3,6-tetra-O-galloyl-D-glucose
43	2OGG	2-O-galloyl-glucose
44	1OGDG	1-O-galloyl-D-glucose
45	6OGG	6-O-galloyl-glucose
46	16DOGDG	1,6-di-O-galloyl-D-glucose
47	3OD1246OTDG	3-O-digalloyl-1,2,4,6-O-tetragalloyl-D-glucose
48 (Figure 12)	CA	caffeoylbenzoyltartaric acid
49	12OD	1,2-O-diferuloylglycerol
50	CAME	chlorogenic acid methyl ester
51	SM	sinapoyl malate
52	MC	methyl caffeate
53	4HA4DG	4-hydroxycinnamyl alcohol 4-D-glucoside
54	7M5P	7-methoxy-5-prenyloxycoumarin
55	SADG	syringoylcaffeoylquinic acid-D-glucose
56	3OPCAOG	3-O-p-Coumaroylquinic acid O-glucoside
57	SA	sinapic acid
58	1OGS	1-O-glucosyl sinapate
59	1O[(E)PC]DG	1-O-[(E)-p-coumaroyl]-D-glucose
60	3M48D34D	3-methyl-4,8-dihydroxy-3,4-dihydroisocoumarin
61	6OFDG	6-O-feruloyl-D-glucose
62	PCA	p-coumaryl alcohol
63	7H5M	7-hydroxy-5-methoxycoumarin
64	2HA	2-hydroxycinnamic acid
65	3(4H)PA	3-(4-hydroxyphenyl)-propionic acid
66	C(PH)TA	caffeoyl(p-hydroxybenzoyl)tartaric acid
67	3(3H)PA	3-(3-hydroxyphenyl)-propionate acid
68	(S)2H3(4H)PA	(S)-2-hydroxy-3-(4-hydroxyphenyl)propanoic acid
69	PA	phaseolic acid
70	FA	ferulic acid
71	BCA	brevifolin carboxylic acid
72	MCA	maleoyl-caffeoylquinic acid
73	57D	5,7-dimethoxycoumarin
74	3OPCA	3-O-p-coumaroylquinic acid
75	NA	neochlorogenic acid(5-O-caffeoylquinic acid)
76	1O(34D)OCG	1′-O-(3,4-dihydroxyphenethyl)-O-caffeoyl-glucoside
77	M4H	methyl 4-hydroxycinnamate
78 (Figure 13)	23456P2H	2,3,4,5,6-pentahydroxyhexyl 2-hydroxybenzoate
79	1O(34D5MB)G	1-O-(3,4-dihydroxy-5-methoxy-benzoyl)-glucoside
80	VA4OG	vanillic acid-4-O-glucoside
81	PA4OG	protocatechuic acid-4-O-glucoside
82	345T1OG	3,4,5-trimethoxyphenyl-1-O-glucoside
83	35D4MA	3,5-dihydroxy-4-methoxybenzoic acid
84	25DAOG	2,5-dihydroxybenzoic acid O-glucoside
85	1OVDG	1-O-vanilloyl-D-glucose
86	3H	3-hydroxybenzaldehyde
87	4HA	4-hydroxybenzoic acid
88	SA2OG	salicylic acid-2-O-glucoside
89	SA	syringic acid
90	4H	4-hydroxybenzaldehyde
91	4OG4HA	4-O-glucosyl-4-hydroxybenzoic acid
92	DP	dimethyl phthalate
93	PA	protocatechuic aldehyde
94	3ODQA	3-O-digalloyl quinic acid
95	246TA	2,4,6-trihydroxybenzoic acid
96 (Figure 14)	4H	4-hydroxyacetophenone
97	PHAA	p-hydroxyphenyl acetic acid
98	3H4I3OG	3-hydroxy-4-isopropylbenzylalcohol-3-O-glucoside
99	M24D	methyl 2,4-dihydroxyphenylacetate
100	2PDβG	2-phenylethyl-D-β-glucopyranoside
101	24D6M	2′,4′-dihydroxy-6′-methoxyacetophenone
102	34DA	3,4-dihydroxybenzeneacetic acid
103 (Figure 15)	35DOGA	3,5-di-O-galloylshikimic acid
104	3GA	3-galloylshikimic acid
105	T5O(PC)S	trans-5-O-(p-coumaroyl) shikimate
106	5OCA	5-O-caffeoylshikimic acid
107 (Figure 16)	(5LG)LAA	(5-L-glutamyl)-L-amino acid
108	NαALO	N-α-acetyl-L-ornithine
109	5OLP	5-oxo-L-proline
110	T4HLP	trans-4-hydroxy-L-proline
111	NALM	N-acetyl-L-methionine
112	LILA	L-isoleucyl-L-aspartate
113	LGLI	L-glycyl-L-isoleucine
114	LAAOD	L-aspartic acid-O-diglucoside
115	NALAA	N-acetyl-L-aspartic acid
116	LAsLP	L-aspartyl-L-phenylalanine
117	LGLP	L-glycyl-L-phenylalanine
118	5HLT	5-hydroxy-L-tryptophan
119	NALT	N-acetyl-L-tryptophan
120	LPLP	L-prolyl-L-phenylalanine
121	S(5A)LH	S-(5′-adenosy)-L-homocysteine
122	S(M)G	S-(methyl)glutathione
123	NND	N,N-dimethylglycine
124	N(3I)LA	N-(3-indolylacetyl)-L-alanine
125	LPLL	L-prolyl-L-leucine
126	NGLL	N-glycyl-L-leucine
127	NALGA	N-acetyl-L-glutamic acid
128	NALG	N-acetyl-L-glutamine
129	3H3M15DA	3-hydroxy-3-methylpentane-1,5-dioic acid
130	LGOG	L-glutamine-O-glycoside
131	LαGLGA	L-α-glutamyl-L-glutamic acid
132	LGAOG	L-glutamic acid-O-glycoside
133	AM	acetylleucine monoethanolamine
134	34DLP	3,4-dihydroxy-L-phenylalanine
135	NALL	N-acetyl-L-leucine
136	NALA	N-acetyl-L-arginine
137	LAlLP	L-alanyl-L-phenylalanine
138	3MLH	3-methyl-L-histidine
139 (Figure 17)	910EA	9,10-epoxyoctadecanoic acid
140	91013T11OA	9,10,13-trihydroxy-11-octadecenoic acid
141	9H12O15(Z)OA	9-hydroxy-12-oxo-15(Z)-octadecenoic acid
142	91213T1015OA	9,12,13-trihydroxy-10,15-octadecadienoic acid
143	9H13O10OA	9-hydroxy-13-oxo-10-octadecenoic acid
144	1213E9OA	12,13-epoxy-9-octadecenoic acid
145	13-KODE	(9Z,11E)-13-oxooctadeca-9,11-dienoic acid
146	12,13-DHOME	(9Z)-12,13-dihydroxyoctadec-9-enoic acid
147	9H101215OA	9-hydroxy-10,12,15-octadecatrienoic acid
148	13SH9Z11EOA	13S-hydroperoxy-9Z,11E-octadecadienoic acid
149	PA	punicic acid
150	9O10E12ZOA	9-oxo-10E,12Z-octadecadienoic acid
151	2L1OG	2-linoleoylglycerol-1-O-glucoside
152	2αLG13DOG	2-α-linolenoyl-glycerol-1,3-di-O-glucoside
153	1αLG3OG	1-α-linolenoyl-glycerol-3-O-glucoside
154	1LRGD	1-linolenoyl-rac-glycerol-diglucoside
155	1SG	1-stearidonoyl-glycerol
156	2αLG1OG	2-α-linolenoyl-glycerol-1-O-glucoside
157	815D591113EA	8,15-dihydroxy-5,9,11,13-eicosatetraenoic acid
158	C4710131619DA	cis-4,7,10,13,16,19-docosahexaenoic acid
159	C10HA	cis-10-heptadecenoic acid
160	9(10)-EpOME	(9R,10S)-(12Z)-9,10-epoxyoctadecenoic acid
161	HA	heptadecanoic acid
162	13(S)-HODE	13(S)-hydroxyoctadeca-9Z,11E-dienoic acid
163	9SH10E12ZOA	9S-hydroxy-10E,12Z-octadecadienoic acid
164	912O6YA	9,12-octadecadien-6-ynoic acid
165	EA	eicosadienoic acid
166	9H10E1215ZOA	9-hydroperoxy-10E,12,15Z-octadecatrienoic acid
167	VA	vaccenic acid
168	AA	arachidonic acid
169	γLA	γ-linolenic acid
170	αLA	α-linolenic acid
171	1αLG23DOG	1-α-linolenoyl-glycerol-2,3-di-O-glucoside
172	1L23DOG	1-linoleoylglycerol-2,3-di-O-glucoside
173	7S,8S-DiHODE	(9Z,12Z)-(7S,8S)-dihydroxyoctadeca-9,12-dienoic acid
174	2L13DOG	2-linoleoylglycerol-1,3-di-O-glucoside
175	1OSG	1-oleoyl-sn-glycerol
176 (Figure 18)	A5M	adenosine 5′-monophosphate
177	2D1P	2-deoxyribose-1-phosphate
178	6M	6-methylmercaptopurine
179	I7NG	isopentenyladenine-7-N-glucoside
180	A5D	adenosine 5′-diphosphate
181	5AR	5-aminoimidazole ribonucleotide
182	U5M	uridine 5′-monophosphate
183	N6I	N6-isopentenyladenine
184	NDR	nicotinate D-ribonucleoside
185	NADP	nicotinamide adenine dinucleotide phosphate
186	2D5M	2′-deoxyadenosine-5′-monophosphate
187	9(A)H	9-(arabinosyl)hypoxanthine
188	G5M	guanosine 5′-monophosphate
