# Supplementary material for: Altitudinal Variation of Metabolites, Mineral Elements and Antioxidant Activities of Rhodiola crenulata (Hook.f. & Thomson) H.Ohba
Source: Molecules. 2021 Dec 5;26(23):7383. doi: 10.3390/molecules26237383 (PMC8658832; doi:10.3390/molecules26237383)
Supplement: Supplementary file 1 [file molecules-26-07383-s001.zip › 20211103-Figure S1-3.pdf]

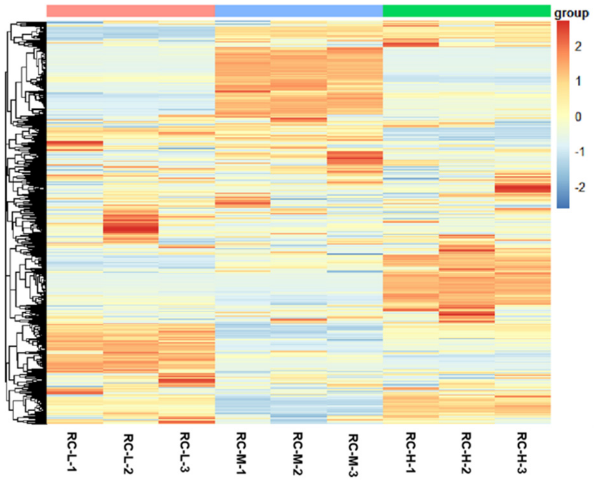

Figure S1. Heat map for a total of 1165 metabolites in RC-L, RC-M, and RC-H.

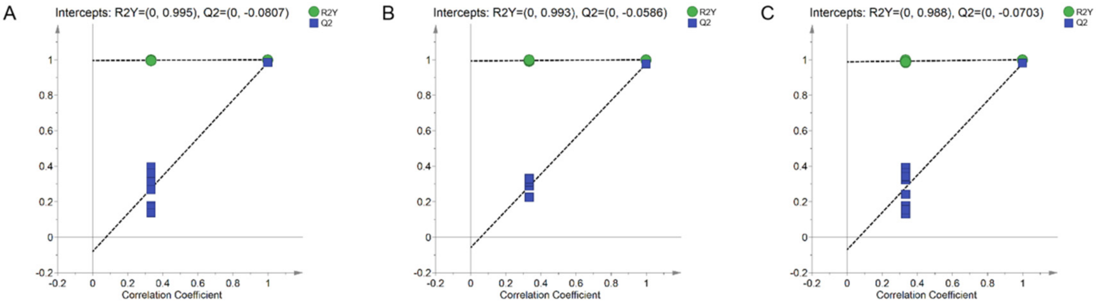

Figure S2. OPLS-DA permutation plots. A: RC-H vs RC-L; B: RC-M vs RC-L; C: RC-H vs RC-M.

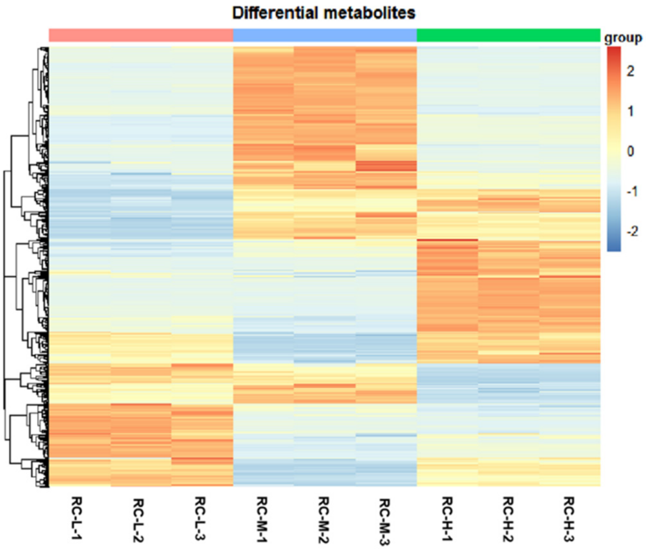

Figure S3. Heat map for recognized differential metabolites in RC-L, RC-M, and RC-H.
