# Supplementary material for: Altitudinal Variation of Metabolites, Mineral Elements and Antioxidant Activities of Rhodiola crenulata (Hook.f. & Thomson) H.Ohba
Source: Molecules. 2021 Dec 5;26(23):7383. doi: 10.3390/molecules26237383 (PMC8658832; doi:10.3390/molecules26237383)
Supplement: Supplementary file 1 [file molecules-26-07383-s001.zip › 20211124-V5-Table S1.pdf]

Table S1: List of identified metabolites between quality control, RC-L, RC-M, and RC-H

Number	Compounds	Class	qc-01	qc-02	qc-03	RC-L-1	RC-L-2	RC-L-3	RC-M-1	RC-M-2	RC-M-3	RC-H-1	RC-H-2	RC-H-3
1	Quercetin-3-O-rutinoside (Rutin)	Flavonoids	3.75E+05	3.19E+05	3.75E+05	9.00E+00	9.00E+00	9.00E+00	5.37E+05	5.46E+05	5.43E+05	4.77E+05	4.47E+05	4.48E+05
2	7-O-Methxyl Quercetin (Rhamnetin)	Flavonoids	4.85E+05	4.71E+05	4.85E+05	9.00E+00	9.00E+00	9.00E+00	1.23E+06	1.17E+06	1.11E+06	1.23E+05	1.26E+05	1.17E+05
3	Delphinidin-3-O-glucoside	Flavonoids	5.47E+05	5.51E+05	2.29E+05	9.70E+03	8.11E+04	2.42E+04	3.32E+05	2.40E+05	6.17E+04	8.93E+05	7.66E+04	1.21E+05
4	Procyanidin C1	Flavonoids	1.16E+05	8.93E+04	1.16E+05	2.14E+04	3.52E+04	2.47E+04	4.43E+04	4.58E+04	4.67E+04	2.65E+05	2.31E+05	2.36E+05
5	Quercetin-7-O-rutinoside	Flavonoids	2.89E+05	2.46E+05	2.97E+05	2.79E+04	3.55E+04	9.60E+04	2.31E+05	2.54E+05	3.70E+05	4.70E+05	4.87E+05	4.36E+05
6	Isoquercitrin	Flavonoids	4.81E+04	4.17E+04	2.48E+04	9.70E+03	3.69E+03	2.04E+03	2.31E+04	3.45E+04	3.13E+03	1.56E+04	1.06E+05	1.06E+04
7	Procyanidin C2	Flavonoids	8.18E+04	6.14E+04	8.13E+04	2.65E+04	2.33E+04	2.26E+04	3.27E+04	2.78E+04	3.09E+04	1.71E+05	1.57E+05	1.86E+05
8	Procyanidin B1	Flavonoids	6.32E+05	5.08E+05	6.33E+05	1.58E+05	1.69E+05	1.65E+05	4.52E+05	4.54E+05	5.00E+05	1.20E+06	9.67E+05	1.16E+06
9	Procyanidin B3	Flavonoids	1.26E+06	1.07E+06	1.26E+06	2.41E+05	2.58E+05	2.70E+05	1.54E+06	1.57E+06	1.59E+06	1.62E+06	1.50E+06	1.74E+06
10	Quercetin 3-beta-D-sophoroside	Flavonoids	2.19E+06	1.96E+06	2.18E+06	4.93E+05	4.47E+05	4.37E+05	2.95E+06	3.09E+06	3.49E+06	2.31E+06	2.48E+06	2.24E+06
11	Eriodictyol-7-O-glucoside	Flavonoids	8.13E+03	6.19E+03	1.41E+06	1.30E+05	1.21E+05	1.03E+05	3.23E+06	2.93E+06	3.14E+06	6.01E+05	6.20E+05	5.68E+05
12	Quercetin-3-O-(2''-O-galactosyl)glucoside	Flavonoids	8.38E+05	7.42E+05	8.51E+05	2.20E+05	2.59E+05	3.26E+05	6.62E+05	7.08E+05	9.21E+05	1.40E+06	1.45E+06	1.09E+06
13	Procyanidin B2	Flavonoids	4.62E+06	3.87E+06	4.60E+06	1.84E+06	1.79E+06	1.67E+06	2.52E+06	2.92E+06	2.85E+06	9.09E+06	8.30E+06	7.62E+06
14	Dihydrokaempferol-7-O-glucoside	Flavonoids	1.48E+06	1.42E+06	1.48E+06	9.57E+04	1.46E+05	1.15E+05	3.14E+06	3.85E+06	2.95E+06	5.65E+05	5.33E+05	5.52E+05
15	Maclurin	Flavonoids	1.00E+01	7.67E+03	1.40E+03	5.66E+02	5.62E+02	5.76E+02	1.00E+01	1.07E+03	1.12E+03	1.64E+03	1.71E+03	4.52E+03
16	6-C-MethylKaempferol-3-glucoside	Flavonoids	1.05E+06	1.00E+06	1.05E+06	1.35E+05	1.28E+05	1.18E+05	2.59E+06	1.70E+06	2.31E+06	5.47E+05	4.82E+05	5.02E+05
17	Homoeriodictyol	Flavonoids	3.30E+04	2.48E+04	3.36E+04	1.94E+04	1.44E+04	1.23E+04	1.38E+04	2.70E+04	1.82E+04	3.01E+04	1.18E+05	3.49E+04
18	Catechin	Flavonoids	6.55E+06	5.58E+06	6.54E+06	2.49E+06	2.90E+06	2.40E+06	5.28E+06	5.84E+06	5.34E+06	1.02E+07	1.02E+07	1.01E+07
19	Rhodioflavonoside	Flavonoids	3.62E+05	3.17E+05	3.64E+05	1.42E+05	1.25E+05	1.58E+05	2.98E+05	3.23E+05	3.67E+05	5.58E+05	5.65E+05	5.16E+05
20	6,7,8-Tetrahydroxy-5-methoxyflavone	Flavonoids	8.96E+05	8.11E+05	8.94E+05	2.55E+05	2.22E+05	2.36E+05	1.29E+06	1.34E+06	1.35E+06	9.28E+05	8.75E+05	9.16E+05
21	5,7,4'-Trihydroxy-6-methoxyflavone (Hispidulin)	Flavonoids	8.96E+05	8.13E+05	8.95E+05	2.56E+05	2.11E+05	2.49E+05	1.33E+06	1.35E+06	1.36E+06	8.61E+05	8.92E+05	9.10E+05
22	Quercetin-3-O-glucoside (Isoquercitrin)	Flavonoids	3.75E+05	3.40E+05	3.75E+05	9.18E+04	1.02E+05	9.25E+04	5.48E+05	5.03E+05	6.99E+05	3.39E+05	3.46E+05	3.73E+05
23	7-Methylkaempferol (Rhamnocitrin)	Flavonoids	8.92E+05	8.12E+05	8.91E+05	2.56E+05	2.24E+05	2.49E+05	1.28E+06	1.39E+06	1.31E+06	8.78E+05	9.14E+05	8.85E+05

24	Dihydrocharcone-4'-O-glucoside	Flavonoids	2.71E+06	2.40E+06	2.70E+06	9.71E+05	1.05E+06	8.75E+05	2.93E+06	3.43E+06	2.84E+06	3.48E+06	3.61E+06	3.41E+06
25	Glucoliquiritin	Flavonoids	1.87E+04	1.52E+04	3.86E+04	1.18E+04	2.13E+04	1.68E+04	3.09E+04	2.91E+04	3.07E+04	3.43E+04	1.19E+05	2.64E+04
26	(-)-Epicatechin-3-(3''-O-methyl)gallate	Flavonoids	8.36E+04	7.39E+04	8.34E+04	3.49E+04	3.53E+04	3.28E+04	7.09E+04	7.56E+04	8.36E+04	1.39E+05	1.19E+05	1.10E+05
27	Dihydrokaempferol-3-O-glucoside	Flavonoids	8.12E+03	6.19E+03	1.55E+06	2.30E+05	2.47E+05	2.31E+05	3.14E+06	3.10E+06	3.16E+06	8.59E+05	8.12E+05	8.58E+05
28	6-Hydroxykaempferol-3,6-O-Diglucoside	Flavonoids	1.75E+05	1.54E+05	1.76E+05	7.00E+04	6.87E+04	7.40E+04	1.54E+05	1.72E+05	1.77E+05	2.49E+05	2.67E+05	2.42E+05
29	Quercetin-3-O-sophoroside (Baimaside)	Flavonoids	6.60E+03	5.88E+03	6.40E+03	3.75E+03	1.29E+03	2.14E+03	8.19E+03	8.34E+03	8.05E+03	8.06E+03	7.18E+03	7.92E+03
30	Spiraeoside	Flavonoids	3.71E+05	3.47E+05	3.71E+05	1.02E+05	9.09E+04	1.06E+05	5.74E+05	6.42E+05	6.07E+05	3.71E+05	2.84E+05	2.94E+05
31	Naringenin-7-O-glucoside (Prunin)	Flavonoids	3.17E+05	2.91E+05	3.14E+05	1.23E+05	1.16E+05	1.01E+05	3.86E+05	4.62E+05	4.42E+05	3.37E+05	3.59E+05	3.11E+05
32	Xanthohumol	Flavonoids	1.28E+03	1.66E+03	1.72E+03	9.84E+02	1.32E+03	6.42E+02	8.23E+02	1.38E+03	1.99E+03	4.18E+02	6.13E+03	1.06E+03
33	Quercetin-3-O-glucosyl(1→4)rhamnoside-7-O-rutinoside	Flavonoids	4.52E+05	4.17E+05	4.59E+05	1.98E+05	2.20E+05	2.54E+05	5.07E+05	3.91E+05	5.85E+05	5.75E+05	6.07E+05	5.33E+05
34	Lancerin	Flavonoids	4.59E+03	5.62E+03	9.69E+03	5.45E+03	6.69E+02	1.11E+04	7.94E+03	1.11E+04	6.38E+03	6.18E+03	5.58E+03	2.86E+04
35	Licoisoflavone A	Flavonoids	6.39E+03	1.23E+04	5.51E+03	2.91E+03	5.91E+02	4.54E+03	1.09E+04	3.42E+03	5.85E+03	1.39E+03	1.67E+04	6.13E+02
36	3,9-Dihydroxypterocarpan	Flavonoids	3.58E+03	4.34E+03	3.07E+03	5.84E+02	2.15E+03	2.19E+03	4.76E+03	1.81E+03	2.30E+03	6.12E+03	1.61E+03	3.62E+03
37	Quercetin-3-O-(2''-O-Rhamnosyl)rutinoside	Flavonoids	3.02E+06	2.84E+06	3.04E+06	1.17E+06	1.41E+06	1.35E+06	4.07E+06	3.65E+06	4.87E+06	3.10E+06	3.06E+06	2.79E+06
38	Aromadendrin-7-O-glucoside	Flavonoids	1.28E+06	1.17E+06	1.28E+06	7.48E+05	7.64E+05	7.49E+05	1.22E+06	1.27E+06	1.16E+06	1.68E+06	1.78E+06	1.64E+06
39	Kaempferol-6,8-di-C-glucoside-7-O-glucoside	Flavonoids	2.12E+06	1.95E+06	2.17E+06	1.54E+06	1.54E+06	1.92E+06	9.46E+05	9.03E+05	1.39E+06	3.50E+06	3.84E+06	3.30E+06
40	Quercetin-3-O-(6''-O-arabinosyl)glucoside	Flavonoids	6.40E+04	5.98E+04	6.38E+04	4.92E+04	4.07E+04	4.77E+04	5.30E+04	4.86E+04	4.28E+04	1.16E+05	8.07E+04	8.09E+04
41	Hesperetin 7-O-glucoside	Flavonoids	1.41E+04	2.07E+03	6.22E+03	2.94E+03	3.88E+03	4.29E+03	6.34E+03	6.06E+03	7.08E+03	6.48E+03	6.46E+03	9.15E+03
42	Vitexin 2''-glucoside	Flavonoids	7.81E+03	6.45E+03	7.73E+03	1.20E+03	6.33E+03	7.38E+03	4.31E+03	1.20E+04	2.43E+03	3.95E+03	7.33E+03	1.81E+04
43	Norlichexanthone	Flavonoids	2.66E+03	2.62E+03	2.81E+03	1.04E+03	1.83E+03	2.21E+03	2.67E+03	2.72E+03	3.09E+03	4.85E+03	3.24E+03	1.85E+03
44	Luteolin-7-O-(2''-O-rhamnosyl)rutinoside	Flavonoids	1.79E+05	1.71E+05	1.81E+05	8.39E+04	1.08E+05	1.00E+05	2.28E+05	1.66E+05	2.91E+05	1.95E+05	1.98E+05	1.60E+05
45	Gallocatechin	Flavonoids	1.04E+03	8.11E+03	1.66E+03	1.46E+03	7.38E+02	6.51E+02	3.37E+03	1.68E+03	1.47E+03	1.91E+03	2.78E+03	6.45E+02
46	2,4,2',4'-tetrahydroxy-3'-prenylchalcone	Flavonoids	3.56E+05	3.42E+05	3.65E+05	1.83E+05	2.03E+05	2.55E+05	3.99E+05	3.94E+05	5.12E+05	3.95E+05	3.96E+05	3.69E+05
47	3',4',7-Trihydroxyflavone	Flavonoids	1.02E+05	9.66E+04	1.04E+05	7.47E+04	7.16E+04	9.04E+04	7.39E+04	7.19E+04	9.72E+04	1.50E+05	1.43E+05	1.35E+05
48	Cyanidin-3-O-sophoroside-5-O-glucoside	Flavonoids	5.84E+04	5.60E+04	5.88E+04	3.38E+04	3.47E+04	3.70E+04	6.01E+04	6.47E+04	8.58E+04	6.65E+04	6.57E+04	5.62E+04
49	Homoorientin	Flavonoids	1.74E+04	1.48E+04	1.90E+04	3.41E+03	2.51E+04	5.39E+03	1.77E+04	1.23E+04	3.16E+04	2.26E+04	1.24E+04	2.50E+04

50	Rhodiosin	Flavonoids	1.87E+06	1.77E+06	1.87E+06	1.39E+06	1.30E+06	1.43E+06	1.54E+06	1.57E+06	1.96E+06	2.45E+06	2.49E+06	2.24E+06
51	Saponarin	Flavonoids	5.18E+03	8.94E+03	8.38E+03	1.04E+03	6.33E+03	7.38E+03	1.32E+04	1.20E+04	2.43E+03	3.95E+03	1.41E+04	7.64E+03
52	1-Isomangostin hydrate	Flavonoids	7.25E+03	2.04E+03	2.49E+03	1.05E+03	1.13E+03	2.78E+03	2.07E+03	3.78E+03	1.59E+03	1.35E+03	3.58E+03	3.63E+03
53	5,7,3',4',5'-Pentahydroxyflavone (Tricetin)	Flavonoids	1.25E+05	1.20E+05	1.23E+05	8.83E+04	1.20E+05	7.43E+04	9.76E+04	9.96E+04	1.17E+05	1.86E+05	1.76E+05	1.15E+05
54	Morusin	Flavonoids	3.20E+04	3.38E+04	3.42E+04	1.67E+04	1.99E+04	3.14E+04	1.15E+04	1.84E+04	7.82E+04	5.35E+04	4.33E+04	1.75E+04
55	Rhodiolgidin	Flavonoids	1.15E+05	1.15E+05	1.21E+05	7.51E+04	8.20E+04	1.20E+05	1.28E+05	1.12E+05	6.40E+04	1.29E+05	2.08E+05	1.25E+05
56	Hesperetin-7-O-rutinoside (Hesperidin)	Flavonoids	2.62E+06	2.45E+06	2.63E+06	2.00E+06	2.06E+06	2.07E+06	2.03E+06	2.25E+06	2.42E+06	3.26E+06	3.55E+06	3.39E+06
57	Quercetin	Flavonoids	3.26E+05	3.18E+05	3.29E+05	1.57E+05	1.63E+05	1.82E+05	4.12E+05	4.69E+05	5.77E+05	2.72E+05	3.09E+05	2.50E+05
58	Kaempferol-7-O-rhamnoside	Flavonoids	2.18E+05	2.03E+05	2.21E+05	1.54E+05	1.57E+05	1.79E+05	2.13E+05	1.97E+05	2.13E+05	2.63E+05	2.51E+05	2.98E+05
59	Kaempferol-3-O-robinoside-7-O-rhamnoside (Robinin)	Flavonoids	1.79E+05	1.74E+05	1.83E+05	9.27E+04	1.10E+05	1.26E+05	2.32E+05	1.56E+05	3.14E+05	1.92E+05	1.73E+05	1.62E+05
60	6''-O-Acetylglycitin	Flavonoids	9.06E+03	6.58E+03	5.86E+03	5.19E+03	1.02E+04	1.00E+01	1.00E+01	5.66E+03	6.24E+03	5.28E+03	1.14E+04	8.05E+03
61	(+)-Afzelechin	Flavonoids	4.74E+03	9.13E+03	5.45E+03	1.49E+03	5.96E+03	4.23E+03	3.40E+03	6.20E+03	5.45E+03	5.44E+03	5.76E+03	7.18E+03
62	Pinostrobin	Flavonoids	1.13E+03	9.40E+03	3.19E+03	1.11E+03	2.79E+03	3.90E+03	1.64E+03	3.89E+03	1.13E+03	3.05E+03	6.34E+03	2.82E+03
63	Procyanidin B4	Flavonoids	3.68E+05	3.49E+05	3.64E+05	3.00E+05	3.13E+05	2.67E+05	3.64E+05	3.20E+05	2.86E+05	4.69E+05	4.76E+05	4.15E+05
64	4',7-Dihydroxyflavone	Flavonoids	3.67E+03	1.19E+03	3.27E+03	6.89E+02	3.64E+03	1.37E+03	5.81E+03	1.20E+03	5.32E+03	2.84E+03	2.72E+03	3.23E+03
65	Sciadopitysin	Flavonoids	3.94E+03	7.87E+03	8.28E+03	9.79E+03	7.65E+03	3.04E+03	1.17E+04	6.51E+03	6.15E+03	2.18E+04	6.30E+03	3.09E+03
66	Procyanidin C1 3'-O-gallate	Flavonoids	9.08E+04	8.36E+04	9.01E+04	8.83E+04	1.02E+05	8.27E+04	3.78E+04	4.29E+04	4.20E+04	1.37E+05	1.37E+05	1.40E+05
67	Homoferreirin	Flavonoids	3.33E+03	5.01E+03	5.95E+03	2.63E+03	5.64E+03	4.16E+03	7.74E+03	9.28E+03	2.30E+03	8.61E+03	7.63E+03	2.19E+03
68	Astragalin	Flavonoids	3.43E+05	3.51E+05	5.34E+05	4.35E+05	8.95E+05	2.10E+05	1.54E+05	1.44E+05	5.95E+05	6.96E+05	5.19E+05	1.06E+06
69	Garcinone D	Flavonoids	8.30E+04	4.02E+04	9.34E+03	8.97E+03	9.43E+03	4.49E+03	8.13E+03	1.06E+04	8.49E+03	1.47E+04	1.01E+04	8.76E+03
70	Isosakuranetin	Flavonoids	1.80E+04	1.97E+04	2.74E+04	2.74E+04	3.42E+04	1.45E+04	7.95E+03	5.85E+03	4.62E+04	3.89E+04	4.00E+04	3.18E+04
71	Epigallocatechin	Flavonoids	3.16E+04	2.96E+04	3.03E+04	2.84E+04	2.69E+04	1.81E+04	2.93E+04	3.20E+04	3.06E+04	3.00E+04	4.17E+04	3.40E+04
72	Eriocitrin	Flavonoids	2.18E+03	2.96E+03	2.98E+03	1.86E+03	2.68E+03	3.63E+03	2.80E+03	1.73E+03	1.42E+03	4.25E+03	4.69E+03	2.67E+03
73	Nobiletin	Flavonoids	5.21E+03	5.66E+03	1.01E+04	1.00E+01	1.09E+04	9.31E+03	1.43E+04	5.62E+03	1.27E+04	2.28E+03	1.40E+04	1.14E+04
74	Quercetin-3-O-xylosyl(1→2)glucoside	Flavonoids	1.75E+04	1.73E+04	1.75E+04	1.43E+04	1.33E+04	1.37E+04	2.12E+04	1.89E+04	1.65E+04	2.21E+04	1.86E+04	1.55E+04
75	Meloside A	Flavonoids	7.81E+03	6.45E+03	4.86E+03	1.20E+03	3.60E+03	7.38E+03	4.60E+03	3.07E+03	4.10E+03	3.95E+03	7.33E+03	4.83E+03

76	Quercetin-3-O-apiosyl(1→2)galactoside	Flavonoids	1.67E+04	1.62E+04	1.68E+04	1.43E+04	1.54E+04	1.47E+04	1.67E+04	1.52E+04	1.53E+04	2.23E+04	1.62E+04	1.86E+04
77	Quercetin-3-O-robinobioside	Flavonoids	4.54E+05	4.37E+05	4.61E+05	4.64E+05	5.47E+05	5.23E+05	2.22E+05	2.01E+05	2.25E+05	6.73E+05	6.43E+05	6.52E+05
78	Kaempferol-3-O-neohesperidoside-7-O-glucoside	Flavonoids	3.18E+05	3.15E+05	3.24E+05	2.62E+05	2.56E+05	3.11E+05	3.01E+05	3.16E+05	3.50E+05	3.75E+05	3.52E+05	3.30E+05
79	Cyanidin-3-O-rutinoside chloride	Flavonoids	1.47E+03	2.10E+03	1.95E+03	8.61E+02	2.72E+03	1.32E+03	1.04E+03	3.08E+03	1.22E+03	1.46E+03	5.99E+02	4.16E+03
80	Sachaloside IV	Flavonoids	6.57E+06	6.37E+06	6.56E+06	3.44E+06	4.55E+06	3.38E+06	9.23E+06	1.06E+07	1.03E+07	4.99E+06	4.45E+06	4.99E+06
81	Deguelin	Flavonoids	2.88E+04	2.35E+04	1.54E+04	6.17E+03	1.68E+04	1.19E+04	1.32E+04	1.65E+04	2.27E+04	6.01E+03	8.55E+03	2.79E+04
82	Quercetin-3-O-galactoside (Hyperin)	Flavonoids	5.59E+05	5.55E+05	5.62E+05	3.02E+05	2.74E+05	3.30E+05	7.32E+05	8.88E+05	1.18E+06	3.44E+05	3.90E+05	3.60E+05
83	Rhamnetin-3-O-Rutinoside	Flavonoids	6.35E+04	6.34E+04	6.40E+04	1.10E+04	1.83E+04	1.52E+04	1.48E+05	1.10E+05	1.67E+05	1.97E+04	1.79E+04	1.57E+04
84	Cyanidin	Flavonoids	1.38E+04	8.11E+03	8.79E+03	8.94E+03	1.17E+04	8.22E+03	6.49E+03	4.39E+03	5.14E+03	6.25E+03	8.72E+03	1.94E+04
85	5,7,3',4',5'-Pentahydroxydihydroflavone	Flavonoids	6.08E+05	5.84E+05	5.96E+05	7.85E+05	6.75E+05	6.85E+05	2.85E+05	3.06E+05	2.61E+05	8.67E+05	8.09E+05	8.79E+05
86	(-)-Gallocatechin gallate	Flavonoids	4.40E+03	1.69E+03	2.49E+03	1.58E+03	1.92E+03	2.56E+03	5.06E+03	1.79E+03	1.42E+03	2.09E+03	2.99E+03	2.11E+03
87	Isorhamnetin-3-O-rutinoside (Narcissin)	Flavonoids	6.46E+04	6.46E+04	6.49E+04	1.40E+04	1.51E+04	1.63E+04	1.55E+05	9.67E+04	1.83E+05	1.68E+04	1.97E+04	1.68E+04
88	Quercetin-3-O-(2''-O-rhamnosyl)galactoside	Flavonoids	3.80E+05	3.71E+05	3.81E+05	2.68E+05	3.09E+05	2.80E+05	4.33E+05	4.98E+05	5.28E+05	3.74E+05	2.77E+05	3.51E+05
89	Kaempferol-7-O-glucoside	Flavonoids	1.53E+06	1.52E+06	1.56E+06	1.11E+06	1.14E+06	1.34E+06	1.94E+06	1.32E+06	2.54E+06	1.28E+06	1.49E+06	1.42E+06
90	Fustin	Flavonoids	3.38E+03	2.45E+03	2.49E+03	2.44E+03	3.33E+03	2.37E+03	1.92E+03	1.59E+03	1.19E+03	1.31E+03	3.71E+03	4.49E+03
91	Rhodionidin	Flavonoids	8.13E+03	6.19E+03	2.05E+06	1.52E+06	1.67E+06	1.59E+06	2.69E+06	2.39E+06	2.46E+06	1.88E+06	1.80E+06	1.88E+06
92	Genistein 7-O-Glucoside (Genistin)	Flavonoids	8.34E+02	5.05E+03	4.03E+03	1.60E+03	2.48E+03	6.99E+03	3.88E+03	2.70E+03	3.32E+03	5.68E+03	5.41E+03	1.79E+03
93	Quercetin-3-O-rutinoside-7-O-glucoside	Flavonoids	8.54E+06	8.40E+06	8.59E+06	4.81E+06	5.48E+06	5.21E+06	1.19E+07	1.35E+07	1.45E+07	5.59E+06	6.12E+06	6.31E+06
94	Kaempferol-3-O-galactoside (Trifolin)	Flavonoids	1.59E+06	1.58E+06	1.62E+06	1.01E+06	9.94E+05	1.28E+06	2.00E+06	2.02E+06	2.90E+06	1.26E+06	1.15E+06	1.39E+06
95	Quercetin-3-O-rhamnoside (Quercitrin)	Flavonoids	1.90E+05	1.93E+05	1.97E+05	1.64E+05	1.69E+05	2.18E+05	1.71E+05	1.50E+05	2.28E+05	2.08E+05	2.35E+05	1.94E+05
96	Kaempferol-3-O-neohesperidoside	Flavonoids	1.36E+07	1.36E+07	1.39E+07	1.48E+07	1.48E+07	1.76E+07	8.07E+06	6.56E+06	1.01E+07	1.94E+07	1.76E+07	1.74E+07
97	Plantagoside	Flavonoids	5.22E+03	3.73E+03	4.60E+03	1.86E+03	9.59E+03	2.96E+03	2.54E+03	2.89E+03	2.30E+03	8.73E+03	4.43E+03	3.34E+03
98	Calycosin	Flavonoids	3.17E+03	6.93E+03	3.66E+03	5.77E+03	1.46E+03	3.29E+03	2.51E+03	4.86E+03	5.12E+03	4.36E+03	2.71E+03	4.95E+03
99	Kaempferol-3-O-sambubioside	Flavonoids	5.56E+04	5.59E+04	5.65E+04	4.14E+04	3.89E+04	4.91E+04	6.28E+04	6.65E+04	8.78E+04	4.93E+04	5.13E+04	4.64E+04
100	Catechin gallate	Flavonoids	1.13E+06	1.10E+06	1.12E+06	1.33E+06	1.06E+06	1.24E+06	8.91E+05	7.24E+05	9.35E+05	1.34E+06	1.31E+06	1.46E+06
101	Brazilin	Flavonoids	9.34E+03	3.55E+03	6.28E+03	6.27E+03	6.21E+03	7.24E+03	3.57E+03	8.97E+03	1.97E+03	5.42E+03	9.45E+03	7.38E+03

102	2'-Hydoxy,5-methoxyGenistein-O-rhamnosyl-glucoside	Flavonoids	6.83E+04	6.83E+04	6.85E+04	1.49E+04	1.30E+04	1.69E+04	1.52E+05	1.08E+05	2.08E+05	1.46E+04	1.85E+04	1.67E+04
103	Quercetin-3-O-xyloside (Reynoutrin)	Flavonoids	2.47E+04	2.41E+04	2.56E+04	9.96E+03	2.44E+04	1.72E+04	3.27E+04	3.22E+04	4.13E+04	1.61E+04	1.88E+04	2.23E+04
104	Baicalein	Flavonoids	1.66E+03	4.14E+03	1.32E+03	1.10E+03	1.74E+03	1.40E+03	6.05E+02	8.00E+02	1.30E+03	2.54E+03	1.75E+03	4.16E+02
105	Peonidin-3-glucoside	Flavonoids	2.27E+03	3.60E+04	3.01E+04	2.35E+04	4.62E+04	3.44E+04	1.92E+04	1.96E+04	6.61E+03	2.45E+04	6.95E+04	2.08E+04
106	Epicatechin	Flavonoids	1.28E+07	1.25E+07	1.28E+07	1.46E+07	1.54E+07	1.52E+07	7.30E+06	7.49E+06	7.57E+06	1.64E+07	1.63E+07	1.70E+07
107	Demethoxycapillarisin	Flavonoids	1.48E+04	1.29E+04	8.74E+03	7.42E+03	1.13E+04	9.65E+03	6.96E+03	6.76E+03	4.16E+03	8.80E+03	9.75E+03	1.25E+04
108	8-Prenylkaempferol	Flavonoids	9.67E+03	9.46E+03	9.73E+03	1.05E+04	1.49E+04	1.09E+04	4.04E+03	3.61E+03	5.01E+03	1.27E+04	1.40E+04	1.26E+04
109	9-Hydroxycalabaxanthone	Flavonoids	1.39E+04	6.94E+03	8.08E+03	1.31E+04	1.10E+04	1.90E+03	1.33E+04	1.00E+01	1.02E+04	1.30E+04	7.96E+03	7.26E+03
110	3,5,7,4'-Tetrahydroxyflavone (Kaempferol)	Flavonoids	1.43E+07	1.44E+07	1.47E+07	1.21E+07	1.36E+07	1.49E+07	1.44E+07	1.34E+07	1.72E+07	1.51E+07	1.47E+07	1.41E+07
111	Quercetin-3-O-neohesperidoside	Flavonoids	3.83E+05	3.73E+05	3.84E+05	2.81E+05	3.33E+05	2.87E+05	4.51E+05	4.65E+05	5.63E+05	3.26E+05	2.80E+05	3.64E+05
112	Methyl hesperidin	Flavonoids	1.00E+01	4.67E+03	2.88E+03	2.46E+03	2.11E+03	4.50E+03	2.28E+03	3.20E+03	1.22E+03	2.90E+03	1.47E+03	5.32E+03
113	5,7,3',4'-Tetrahydroxyflavone (Luteolin)	Flavonoids	1.40E+07	1.38E+07	1.40E+07	1.90E+07	1.92E+07	1.91E+07	4.43E+06	4.46E+06	3.90E+06	2.00E+07	1.99E+07	2.09E+07
114	Pedalitin	Flavonoids	2.72E+04	4.08E+04	3.61E+04	4.27E+04	2.01E+04	4.72E+04	3.76E+04	2.05E+04	4.80E+04	3.99E+04	4.17E+04	3.37E+04
115	Quercetin-3',4'-dimethyl ether	Flavonoids	2.05E+04	2.05E+04	2.13E+04	1.68E+04	1.63E+04	2.29E+04	2.11E+04	2.37E+04	2.73E+04	2.08E+04	1.50E+04	2.28E+04
116	Sakuranetin	Flavonoids	2.79E+03	7.90E+02	2.70E+03	2.17E+03	2.94E+03	2.10E+03	3.46E+03	3.18E+03	2.42E+03	2.12E+03	2.12E+03	3.26E+03
117	Sexangularetin-3-O-glucoside-7-O-rhamnoside	Flavonoids	6.54E+04	6.56E+04	6.64E+04	1.03E+04	1.66E+04	1.84E+04	1.49E+05	1.09E+05	1.91E+05	1.66E+04	1.34E+04	1.70E+04
118	Epicatechin gallate	Flavonoids	1.17E+06	1.15E+06	1.18E+06	1.22E+06	1.37E+06	1.36E+06	9.32E+05	8.45E+05	8.89E+05	1.25E+06	1.35E+06	1.48E+06
119	Myricetin	Flavonoids	9.10E+03	5.73E+03	5.61E+03	6.25E+03	4.17E+03	8.65E+03	7.23E+03	1.87E+03	3.65E+03	3.78E+03	9.94E+03	5.58E+03
120	2'-Hydroxygenistein	Flavonoids	3.39E+03	1.59E+03	3.22E+03	4.62E+03	2.24E+03	3.70E+03	2.29E+03	1.94E+03	4.85E+03	1.52E+03	5.34E+03	3.84E+03
121	Quercetin-7-O-glucoside	Flavonoids	4.69E+05	4.75E+05	4.79E+05	3.39E+05	3.94E+05	4.25E+05	5.47E+05	6.12E+05	6.87E+05	3.97E+05	3.97E+05	3.75E+05
122	Flavone	Flavonoids	1.45E+04	1.41E+04	1.34E+04	1.46E+04	1.80E+04	1.44E+04	7.85E+03	8.86E+03	1.08E+04	1.60E+04	1.38E+04	1.74E+04
123	2',3,5,7-Tetrahydroxyflavone	Flavonoids	1.03E+04	1.18E+04	1.41E+04	1.38E+04	3.06E+04	2.56E+03	6.39E+03	6.11E+03	1.97E+04	1.70E+04	1.26E+04	1.75E+04
124	Sinensetin	Flavonoids	4.67E+03	2.56E+03	2.06E+03	2.15E+03	4.04E+03	1.29E+03	1.00E+01	1.97E+03	1.70E+03	2.47E+03	3.32E+03	1.64E+03
125	Safflor Yellow A	Flavonoids	5.55E+03	1.17E+04	7.89E+03	1.09E+04	9.60E+03	1.00E+01	9.32E+03	1.43E+04	9.64E+03	9.45E+03	4.58E+03	6.19E+03
126	Phloretin-2'-O-glucoside (Phlorizin)	Flavonoids	2.89E+05	2.91E+05	2.93E+05	3.78E+05	3.84E+05	4.11E+05	1.37E+05	1.29E+05	1.38E+05	4.08E+05	3.46E+05	3.89E+05
127	7-Hydroxyflavone	Flavonoids	2.62E+03	6.76E+03	5.33E+03	2.43E+03	6.07E+03	2.32E+03	5.38E+03	6.21E+03	1.21E+04	2.22E+03	4.00E+03	4.33E+03

128	Cimifugin	Flavonoids	1.29E+04	6.50E+03	1.58E+04	1.91E+04	2.28E+04	1.18E+04	9.99E+03	1.23E+04	1.72E+04	2.27E+04	1.35E+04	1.59E+04
129	Dihydromyricetin	Flavonoids	4.45E+04	2.53E+04	3.13E+04	4.71E+04	2.78E+04	4.26E+04	2.62E+04	1.85E+04	2.58E+04	3.35E+04	4.77E+04	2.84E+04
130	Quercetin 7-O-beta-D-glucoside	Flavonoids	2.52E+05	2.54E+05	2.53E+05	1.02E+05	1.12E+05	1.14E+05	5.03E+05	4.92E+05	5.07E+05	9.81E+04	1.04E+05	9.80E+04
131	Beta-mangostin	Flavonoids	1.73E+04	8.55E+03	1.62E+04	2.06E+04	2.98E+04	8.22E+03	1.43E+04	1.82E+04	5.25E+03	1.19E+04	1.72E+04	2.46E+04
132	Ononin	Flavonoids	4.63E+04	6.83E+04	5.79E+04	5.56E+04	3.88E+04	1.06E+05	5.33E+04	3.37E+04	4.92E+04	4.06E+04	8.92E+04	5.25E+04
133	Rhodiolin	Flavonoids	3.48E+07	3.57E+07	3.51E+07	3.92E+07	3.86E+07	4.17E+07	3.06E+07	3.00E+07	3.46E+07	3.53E+07	3.59E+07	3.39E+07
134	Naringenin chalcone	Flavonoids	2.09E+06	2.14E+06	2.11E+06	2.45E+06	2.58E+06	2.57E+06	1.63E+06	1.60E+06	1.87E+06	2.19E+06	2.21E+06	2.23E+06
135	2,3-Dehydrosilybin A	Flavonoids	1.64E+05	1.36E+04	9.38E+04	2.47E+04	1.23E+05	1.44E+05	1.79E+05	1.86E+04	3.06E+04	1.07E+05	2.26E+04	1.25E+05
136	5,7,3',4'-tetrahydroxyisoflavone (Isoluteolin)	Flavonoids	1.78E+06	1.83E+06	1.77E+06	2.79E+06	2.68E+06	2.71E+06	5.27E+05	5.56E+05	5.67E+05	2.47E+06	2.33E+06	2.32E+06
137	Pinobanksin	Flavonoids	3.35E+06	3.42E+06	3.35E+06	4.07E+06	4.01E+06	4.06E+06	2.66E+06	2.54E+06	3.03E+06	3.44E+06	3.59E+06	3.46E+06
138	5,7,4'-Trihydroxyflavanone (Naringenin)	Flavonoids	3.35E+06	3.44E+06	3.36E+06	4.15E+06	3.94E+06	4.19E+06	2.68E+06	2.64E+06	2.94E+06	3.56E+06	3.38E+06	3.54E+06
139	Luteolin-7-O-rutinoside	Flavonoids	8.99E+06	9.18E+06	9.15E+06	6.93E+06	8.17E+06	8.24E+06	1.18E+07	1.00E+07	1.54E+07	6.20E+06	6.68E+06	6.75E+06
140	6-Deoxyjacareubin	Flavonoids	3.26E+03	1.00E+01	1.17E+03	8.60E+02	1.00E+01	1.78E+03	4.50E+03	8.82E+02	1.00E+01	1.28E+03	9.30E+02	1.00E+01
141	Rhodiolgin	Flavonoids	2.32E+05	2.36E+05	2.35E+05	1.08E+05	1.14E+05	1.29E+05	4.47E+05	4.08E+05	4.89E+05	1.10E+05	8.69E+04	9.68E+04
142	Isorhamnetin	Flavonoids	1.41E+05	1.48E+05	1.44E+05	1.57E+05	1.64E+05	1.84E+05	1.26E+05	1.11E+05	1.51E+05	1.46E+05	1.45E+05	1.31E+05
143	Gallocatechin 3-O-gallate	Flavonoids	4.29E+04	4.43E+04	4.35E+04	2.73E+04	2.65E+04	3.21E+04	3.23E+04	4.85E+04	1.38E+05	2.37E+04	2.64E+04	2.10E+04
144	Luteolin-7-O-neohesperidoside (Lonicerin)	Flavonoids	5.27E+06	5.41E+06	5.33E+06	4.63E+06	4.62E+06	5.11E+06	6.93E+06	5.80E+06	8.53E+06	3.81E+06	3.87E+06	3.98E+06
145	Quercetin-3-O-(6''-galloyl)galactoside	Flavonoids	4.25E+04	4.85E+04	4.68E+04	7.10E+04	6.92E+04	1.06E+05	9.00E+00	9.00E+00	9.00E+00	7.20E+04	7.05E+04	5.73E+04
146	Baicalin	Flavonoids	1.12E+04	9.57E+03	8.42E+03	1.22E+04	1.23E+04	6.94E+03	5.28E+03	5.87E+03	1.14E+04	5.58E+03	7.87E+03	1.21E+04
147	Isosalipurposide (Phlorizin Chalcone)	Flavonoids	4.50E+04	4.59E+04	4.60E+04	3.21E+04	3.58E+04	4.00E+04	6.85E+04	7.04E+04	6.73E+04	2.22E+04	3.11E+04	3.23E+04
148	Quercetin-5-O-(6''-malonyl)glucosyl-5-O-glucoside	Flavonoids	4.93E+04	5.11E+04	4.95E+04	6.32E+04	5.43E+04	6.44E+04	4.44E+04	3.59E+04	5.26E+04	4.38E+04	4.99E+04	5.06E+04
149	Pelargonidin-3-O-glucoside	Flavonoids	1.41E+03	7.92E+03	3.98E+03	2.06E+03	1.03E+04	1.86E+03	5.77E+03	1.87E+03	8.86E+02	6.85E+03	3.34E+03	9.56E+02
150	Alpha-Toxicarol	Flavonoids	1.76E+04	1.42E+04	1.49E+04	1.24E+04	1.49E+04	1.30E+04	1.46E+04	2.76E+04	1.79E+04	4.78E+03	6.29E+03	2.02E+04
151	Abyssinone V	Flavonoids	1.56E+04	7.06E+03	7.57E+03	1.14E+04	3.96E+03	7.26E+03	7.01E+03	7.04E+03	1.78E+04	2.31E+03	6.26E+03	8.88E+03
152	Kaempferol-3-O-rhamnoside (Afzelin)(Kaempferin)	Flavonoids	9.16E+05	9.61E+05	9.36E+05	1.03E+06	1.15E+06	1.19E+06	8.43E+05	6.62E+05	1.07E+06	8.97E+05	8.57E+05	8.26E+05
153	Quercetin-3-O-(2''-Galloyl)Arabinoside	Flavonoids	5.19E+04	5.27E+04	5.08E+04	6.53E+04	6.04E+04	5.63E+04	4.12E+04	5.54E+04	5.39E+04	4.72E+04	4.22E+04	4.95E+04

154	Mangostanol	Flavonoids	3.58E+03	3.55E+03	2.66E+03	2.02E+03	3.57E+03	2.64E+03	1.63E+03	4.02E+03	3.22E+03	2.53E+03	2.52E+03	1.14E+03
155	2-Benzal-4-hydroxyacetophenone	Flavonoids	1.73E+04	3.34E+03	7.41E+03	1.01E+04	8.53E+03	2.38E+03	1.56E+04	9.64E+03	8.29E+03	3.54E+03	4.21E+03	7.16E+03
156	Silychristin	Flavonoids	7.95E+04	9.52E+04	6.71E+04	4.75E+02	9.10E+04	5.55E+04	4.13E+04	1.22E+05	1.23E+05	5.47E+03	1.02E+04	8.82E+04
157	Isogentisin	Flavonoids	2.89E+03	5.86E+03	2.99E+03	2.80E+03	3.67E+03	4.21E+03	3.04E+03	2.38E+03	3.11E+03	1.58E+03	3.16E+03	2.76E+03
158	Daidzin	Flavonoids	2.01E+03	5.13E+03	3.91E+03	1.54E+03	3.47E+03	5.62E+03	3.56E+03	4.34E+03	6.81E+03	2.89E+03	1.64E+03	2.94E+03
159	Luteolin-7-O-glucoside (Cynaroside)	Flavonoids	4.18E+04	4.37E+04	4.21E+04	3.56E+04	2.79E+04	3.83E+04	6.28E+04	6.79E+04	7.01E+04	2.15E+04	2.55E+04	2.27E+04
160	6-Hydroxy-4',5,7-trimethoxyflavone	Flavonoids	1.46E+04	8.36E+03	1.24E+04	1.16E+04	1.06E+04	2.43E+04	7.68E+03	1.06E+04	1.45E+04	6.18E+03	1.33E+04	1.23E+04
161	6-Hydroxykaempferol-3-O-rutinoside-6-O-glucoside	Flavonoids	3.21E+04	3.59E+04	3.43E+04	4.28E+04	4.01E+04	6.02E+04	2.69E+04	1.98E+04	3.00E+04	3.61E+04	3.16E+04	2.95E+04
162	Luteolin-3'-O-glucoside	Flavonoids	5.34E+05	5.44E+05	5.35E+05	3.00E+05	3.20E+05	3.06E+05	7.49E+05	1.05E+06	1.23E+06	1.94E+05	2.08E+05	2.22E+05
163	5-O-Methylquercetin (Azaleatin)	Flavonoids	8.53E+04	8.63E+04	8.58E+04	1.91E+04	1.98E+04	2.33E+04	2.01E+05	1.45E+05	2.55E+05	1.41E+04	1.29E+04	1.49E+04
164	Hinokiflavone	Flavonoids	8.04E+03	1.85E+04	1.94E+04	1.46E+04	4.60E+04	1.92E+04	1.75E+04	1.62E+04	3.71E+03	6.80E+03	1.94E+04	2.64E+04
165	3,8'-Biapigenin	Flavonoids	8.04E+03	1.85E+04	1.94E+04	1.46E+04	4.60E+04	1.92E+04	1.75E+04	1.62E+04	3.71E+03	6.80E+03	1.94E+04	2.64E+04
166	isoliquiritigenin	Flavonoids	3.14E+03	9.36E+03	4.39E+03	5.96E+03	3.64E+03	7.53E+03	4.94E+03	4.46E+03	3.30E+03	2.70E+03	4.97E+03	3.60E+03
167	Kaempferol-4'-O-glucoside	Flavonoids	5.33E+05	5.48E+05	5.36E+05	3.06E+05	3.03E+05	3.33E+05	8.45E+05	1.06E+06	1.14E+06	2.02E+05	2.03E+05	2.11E+05
168	Tectochrysin	Flavonoids	7.17E+03	4.55E+03	3.90E+03	4.25E+03	6.61E+03	1.55E+03	7.37E+03	3.48E+03	4.10E+03	2.35E+03	2.39E+03	3.35E+03
169	Swertiajaponin	Flavonoids	1.63E+04	2.58E+03	4.37E+03	2.83E+03	7.92E+03	5.11E+03	3.31E+03	4.59E+03	4.20E+03	2.95E+03	4.44E+03	2.41E+03
170	Isomangiferin	Flavonoids	9.99E+03	6.44E+03	8.93E+03	6.64E+03	9.79E+03	2.21E+04	2.63E+03	9.45E+03	3.98E+03	2.84E+03	1.25E+04	8.15E+03
171	5,7,3',4'-Tetrahydroxyflavanone (Eriodictyol)	Flavonoids	1.03E+07	1.06E+07	1.02E+07	8.32E+06	8.54E+06	7.57E+06	1.84E+07	1.68E+07	1.58E+07	4.88E+06	4.64E+06	5.34E+06
172	Dihydrokaempferol	Flavonoids	3.40E+05	3.59E+05	3.35E+05	4.46E+05	4.46E+05	4.04E+05	3.38E+05	3.60E+05	3.43E+05	2.81E+05	2.54E+05	2.52E+05
173	Trimethylapigenin	Flavonoids	6.98E+03	2.99E+03	8.46E+03	7.32E+03	2.01E+04	8.05E+03	6.16E+03	6.82E+03	5.23E+03	8.79E+03	3.06E+03	9.43E+03
174	Rotenone	Flavonoids	1.54E+04	8.13E+03	1.85E+04	1.30E+04	4.90E+04	1.83E+04	7.09E+03	1.06E+04	1.57E+04	1.01E+04	2.03E+04	1.70E+04
175	Buddlenoid A	Flavonoids	8.09E+03	8.14E+03	6.33E+03	9.33E+03	1.43E+04	4.38E+03	3.84E+03	3.84E+03	7.94E+03	3.39E+03	8.79E+03	4.08E+03
176	1-Isomangostin	Flavonoids	3.25E+03	2.82E+03	3.51E+03	2.79E+03	1.16E+04	6.23E+02	1.36E+03	2.38E+03	3.50E+03	2.57E+03	1.96E+03	4.00E+03
177	Epigallate catechin gallate (EGCG)	Flavonoids	9.63E+03	1.26E+04	3.67E+03	1.07E+04	6.86E+03	2.92E+03	1.00E+01	2.90E+03	5.91E+03	9.60E+03	1.00E+01	1.18E+03
178	Isorhamnetin-3-O-arabinoside	Flavonoids	1.53E+04	1.66E+04	1.51E+04	2.05E+04	1.57E+04	1.91E+04	1.62E+04	1.89E+04	2.20E+04	1.21E+04	8.25E+03	8.41E+03
179	Neoeriocitrin	Flavonoids	1.64E+04	1.79E+04	9.61E+03	1.06E+04	1.37E+04	1.16E+04	1.31E+04	1.02E+04	9.96E+03	7.00E+03	2.71E+03	8.64E+03

180	Rhoifolin	Flavonoids	1.46E+04	4.76E+03	1.03E+04	1.11E+04	2.73E+04	1.26E+04	7.59E+03	1.15E+04	1.49E+03	3.68E+03	1.19E+04	6.55E+03
181	Troxerutin	Flavonoids	1.98E+03	8.14E+03	2.76E+03	5.32E+03	8.63E+03	8.20E+02	4.05E+02	1.56E+03	4.25E+03	1.00E+01	1.00E+01	6.40E+03
182	Liquiritin	Flavonoids	4.53E+03	5.10E+02	1.67E+03	1.00E+01	3.22E+03	5.11E+03	5.01E+02	1.00E+01	1.12E+03	2.28E+03	1.14E+03	1.00E+01
183	Apigenin	Flavonoids	9.93E+04	1.11E+05	1.01E+05	1.36E+05	1.31E+05	1.47E+05	1.24E+05	1.07E+05	1.33E+05	5.46E+04	5.30E+04	5.50E+04
184	Neohesperidin	Flavonoids	1.08E+05	8.97E+04	9.89E+04	1.27E+05	2.87E+05	1.41E+05	1.03E+04	1.19E+04	1.31E+05	6.84E+04	7.74E+04	6.43E+04
185	Kaempferol 3-O-beta-sophoroside	Flavonoids	2.29E+03	3.44E+03	2.24E+03	1.00E+01	5.44E+03	5.30E+03	7.63E+02	1.00E+01	2.52E+03	7.39E+02	2.01E+03	1.16E+03
186	Phloretin	Flavonoids	9.49E+06	1.08E+07	9.37E+06	1.75E+07	1.74E+07	1.65E+07	7.40E+06	8.10E+06	7.22E+06	6.03E+06	6.23E+06	6.13E+06
187	Dihydroquercetin(Taxifolin)	Flavonoids	1.63E+06	1.86E+06	1.67E+06	2.39E+06	2.85E+06	2.75E+06	1.66E+06	1.74E+06	1.65E+06	8.77E+05	9.28E+05	9.26E+05
188	SAFFLOMIN A	Flavonoids	5.55E+03	5.30E+03	1.36E+04	6.76E+02	3.49E+04	1.18E+04	3.37E+04	2.70E+03	9.61E+03	5.28E+03	1.00E+01	1.05E+04
189	Pelargonidin	Flavonoids	6.80E+03	1.11E+04	7.75E+03	4.77E+03	7.35E+03	3.29E+04	2.73E+03	1.00E+03	3.98E+03	3.49E+03	6.42E+03	4.11E+03
190	Kazinol A	Flavonoids	5.71E+04	6.80E+04	1.18E+05	4.70E+04	4.90E+05	3.21E+04	6.97E+04	1.10E+05	7.33E+04	6.22E+04	5.04E+04	5.91E+04
191	Fisetin	Flavonoids	5.79E+04	5.80E+04	4.47E+04	6.21E+04	1.82E+05	3.87E+04	5.75E+03	4.86E+03	4.65E+04	2.68E+04	2.81E+04	2.54E+04
192	Pinocembrin (Dihydrochrysin)	Flavonoids	1.38E+05	1.65E+05	1.35E+05	3.30E+05	3.21E+05	3.08E+05	5.85E+04	6.05E+04	6.34E+04	9.05E+04	8.99E+04	9.06E+04
193	6,7,4'-Trihydroxyisoflavone	Flavonoids	1.00E+01	3.88E+03	1.82E+03	6.07E+03	4.73E+03	2.30E+03	1.00E+01	2.85E+03	1.13E+03	1.21E+03	1.76E+03	5.89E+02
194	Wighteone	Flavonoids	1.23E+06	1.31E+06	2.54E+06	1.95E+06	1.39E+07	3.82E+05	1.24E+04	1.93E+06	1.61E+04	1.34E+06	1.26E+06	1.49E+06
195	Quercetin-3-O-(2''-O-glucosyl)glucuronide	Flavonoids	2.76E+04	3.42E+04	2.82E+04	6.40E+04	5.74E+04	6.89E+04	1.78E+04	1.67E+04	1.96E+04	1.58E+04	1.32E+04	1.59E+04
196	Tectorigenin	Flavonoids	6.57E+03	9.54E+03	9.02E+03	7.41E+03	1.73E+04	2.92E+04	2.02E+03	1.14E+04	3.18E+03	8.08E+02	2.80E+03	5.47E+03
197	Eriodictyol-7-O-glucoside	Flavonoids	2.17E+05	2.62E+05	2.11E+05	4.38E+05	4.58E+05	3.93E+05	2.26E+05	1.98E+05	2.06E+05	1.21E+05	5.61E+04	3.00E+04
198	Sulfuretin	Flavonoids	1.53E+04	1.11E+04	6.75E+03	4.77E+03	7.35E+03	3.29E+04	3.06E+03	2.51E+03	2.51E+03	1.66E+03	2.86E+03	1.15E+03
199	Cyanidin 3-rutinoside	Flavonoids	2.27E+04	1.49E+05	2.52E+04	1.55E+04	1.62E+04	1.26E+05	6.37E+03	7.19E+03	2.83E+04	6.85E+03	3.15E+03	7.51E+03
200	2'-Hydroxydaidzein	Flavonoids	6.25E+03	5.31E+03	7.41E+03	4.19E+03	1.12E+04	3.29E+04	5.47E+02	5.26E+03	3.98E+03	1.99E+03	1.35E+03	1.98E+03
201	1,3-Trigallic acid	Gallic acid derivatives	1.52E+07	1.51E+07	1.52E+07	1.92E+05	1.73E+05	2.10E+05	4.19E+07	3.98E+07	3.68E+07	9.89E+05	1.11E+06	1.05E+06
202	3-Hydroxy-5-Methylphenol-1-O-(6'-Digalloyl)Glucoside	Gallic acid derivatives	1.11E+05	9.51E+04	1.09E+05	7.50E+04	6.53E+04	6.08E+04	6.22E+04	7.05E+04	6.89E+04	1.87E+05	1.71E+05	1.86E+05
203	3-O-Methylgallic Acid	Gallic acid derivatives	7.84E+05	7.15E+05	7.77E+05	3.86E+05	3.46E+05	3.25E+05	1.01E+06	9.48E+05	9.65E+05	8.80E+05	8.61E+05	8.78E+05
204	1-O-Galloyl-D-glucose	Gallic acid derivatives	1.97E+06	1.80E+06	1.98E+06	1.92E+06	1.86E+06	1.96E+06	7.27E+05	7.38E+05	7.08E+05	3.13E+06	3.37E+06	3.34E+06
205	1-O-Galloyl-rhamnose	Gallic acid derivatives	5.23E+04	4.92E+04	5.22E+04	4.38E+04	3.71E+04	4.32E+04	5.12E+04	3.94E+04	4.74E+04	5.95E+04	7.17E+04	6.83E+04

206	2-O-Galloyl-glucose	Gallic acid derivatives	1.75E+06	1.63E+06	1.75E+06	1.87E+06	1.65E+06	1.88E+06	7.58E+05	6.84E+05	6.79E+05	2.58E+06	2.96E+06	2.79E+06
207	1,6-Di-O-Galloyl-D-Glucose	Gallic acid derivatives	6.34E+05	5.83E+05	6.45E+05	6.84E+05	8.45E+05	7.71E+05	9.00E+00	9.00E+00	9.00E+00	1.12E+06	1.24E+06	1.18E+06
208	Methyl gallate	Gallic acid derivatives	2.16E+05	2.55E+05	2.06E+05	7.33E+04	1.90E+05	2.12E+05	1.80E+05	2.50E+05	1.46E+05	2.57E+05	1.72E+05	2.38E+05
209	Galloyl Methyl gallate	Gallic acid derivatives	5.19E+06	4.88E+06	5.14E+06	4.61E+06	4.43E+06	4.18E+06	4.73E+06	4.67E+06	4.64E+06	5.96E+06	5.82E+06	6.68E+06
210	1,4-Trigallic acid	Gallic acid derivatives	9.22E+04	9.16E+04	9.23E+04	1.82E+04	2.25E+04	1.93E+04	2.07E+05	2.34E+05	1.77E+05	2.78E+04	2.76E+04	2.35E+04
211	p-Dimeric galloyl methyl ester	Gallic acid derivatives	3.17E+05	3.04E+05	3.15E+05	2.76E+05	2.83E+05	2.59E+05	3.61E+05	3.15E+05	2.60E+05	3.89E+05	2.87E+05	3.65E+05
212	6-O-Galloyl-glucose	Gallic acid derivatives	5.66E+05	5.54E+05	5.61E+05	7.11E+05	6.44E+05	6.70E+05	2.31E+05	2.25E+05	2.21E+05	8.25E+05	9.09E+05	7.62E+05
213	Hamamelitannin	Gallic acid derivatives	3.90E+04	3.09E+04	2.38E+04	1.27E+04	2.32E+04	2.07E+04	3.59E+04	2.35E+04	2.00E+04	9.28E+03	2.81E+04	2.96E+04
214	2,3-O-Digalloyl-1,4,6-tri-O-galloyl-glucose	Gallic acid derivatives	4.35E+05	4.40E+05	4.40E+05	2.49E+05	1.91E+05	2.88E+05	8.10E+05	7.59E+05	7.11E+05	2.49E+05	2.62E+05	2.49E+05
215	Gallic acid	Gallic acid derivatives	8.88E+06	8.93E+06	8.86E+06	9.74E+06	1.03E+07	9.62E+06	7.34E+06	7.67E+06	7.59E+06	9.75E+06	9.48E+06	9.18E+06
216	1,2,3,6-Tetra-O-Galloyl-D-Glucose	Gallic acid derivatives	2.14E+07	2.16E+07	2.13E+07	2.84E+07	2.63E+07	2.75E+07	1.31E+07	1.46E+07	1.18E+07	2.57E+07	2.55E+07	2.60E+07
217	Ethyl gallate	Gallic acid derivatives	5.49E+05	5.67E+05	5.44E+05	9.65E+05	9.20E+05	9.27E+05	4.22E+04	3.20E+04	3.97E+04	6.88E+05	9.18E+05	7.83E+05
218	1,2,3,4,6-Penta-O-Galloyl-D-Glucose	Gallic acid derivatives	4.87E+06	5.06E+06	4.90E+06	5.67E+06	5.34E+06	5.90E+06	4.27E+06	4.78E+06	4.71E+06	4.81E+06	5.00E+06	4.41E+06
219	2,3-Di-O-Galloyl-D-Glucose	Gallic acid derivatives	4.44E+06	4.64E+06	4.56E+06	4.96E+06	5.60E+06	5.95E+06	3.51E+06	4.22E+06	3.96E+06	4.50E+06	4.42E+06	4.35E+06
220	1,3,6-Tri-O-galloyl-D-glucose	Gallic acid derivatives	1.81E+06	1.84E+06	1.79E+06	2.37E+06	2.40E+06	2.19E+06	1.28E+06	1.43E+06	1.41E+06	1.88E+06	1.76E+06	1.93E+06
221	1,2,3-Tri-O-galloyl-D-glucose	Gallic acid derivatives	1.64E+06	1.75E+06	1.69E+06	2.16E+06	2.05E+06	2.51E+06	1.12E+06	1.40E+06	1.18E+06	1.68E+06	1.92E+06	1.62E+06
222	2,4,6-Tri-O-galloyl-D-glucose	Gallic acid derivatives	1.96E+06	1.97E+06	1.88E+06	2.71E+06	2.68E+06	2.06E+06	1.58E+06	1.57E+06	1.51E+06	1.84E+06	1.82E+06	1.96E+06
223	3-O-Digalloyl-1,2,4,6-O-tetragalloyl-D-glucose	Gallic acid derivatives	4.56E+05	4.61E+05	4.55E+05	3.30E+05	4.08E+05	3.20E+05	5.91E+05	7.61E+05	7.62E+05	2.25E+05	2.89E+05	2.81E+05
224	Octyl Gallate	Gallic acid derivatives	3.50E+04	2.36E+04	4.83E+04	5.23E+04	6.34E+04	5.88E+04	4.69E+04	4.79E+04	5.91E+04	3.54E+04	3.29E+04	4.20E+04
225	1,7-Di-O-galloyl-D-sedoheptulose	Gallic acid derivatives	1.40E+06	1.58E+06	1.40E+06	2.60E+06	2.36E+06	2.56E+06	8.17E+05	1.03E+06	7.64E+05	1.18E+06	1.31E+06	1.16E+06
226	Methyl 6-O-galloyl-glucoside	Gallic acid derivatives	1.54E+04	1.73E+04	1.51E+04	2.53E+04	3.06E+04	2.24E+04	1.38E+04	1.31E+04	1.27E+04	1.08E+04	9.63E+03	7.51E+03
227	Digallic Acid	Gallic acid derivatives	1.34E+07	1.51E+07	1.34E+07	2.14E+07	1.98E+07	2.13E+07	1.42E+07	1.54E+07	1.39E+07	7.01E+06	7.50E+06	7.64E+06
228	2-O-Salicyl-6-O-Galloyl-D-Glucose	Gallic acid derivatives	5.20E+06	5.92E+06	5.11E+06	8.87E+06	9.35E+06	8.18E+06	4.83E+06	5.24E+06	4.51E+06	3.57E+06	2.84E+06	2.36E+06
229	Monogalloyl-diglucose	Gallic acid derivatives	7.79E+05	9.22E+05	7.69E+05	1.57E+06	1.50E+06	1.49E+06	7.51E+05	6.16E+05	7.84E+05	3.70E+05	2.95E+05	3.47E+05
230	Ellagic acid	Gallic acid derivatives	8.13E+05	9.58E+05	8.08E+05	1.46E+06	1.52E+06	1.42E+06	9.60E+05	8.27E+05	9.65E+05	2.59E+05	2.51E+05	2.61E+05
231	Rhododendrol	Phenylpropanoids	1.73E+05	1.42E+05	1.73E+05	9.00E+00	9.00E+00	9.00E+00	2.36E+05	2.48E+05	1.99E+05	2.36E+05	2.17E+05	2.45E+05

232	2,3,4,5,6-pentahydroxyhexyl 2-hydroxybenzoate	Phenylpropanoids	3.71E+04	2.86E+04	3.71E+04	9.00E+00	9.00E+00	9.00E+00	4.31E+04	3.90E+04	3.18E+04	6.61E+04	4.90E+04	6.80E+04
233	3,5-Di-O-galloylshikimic acid	Phenylpropanoids	4.19E+04	3.74E+04	4.19E+04	9.00E+00	9.00E+00	9.00E+00	1.08E+05	8.18E+04	6.37E+04	2.02E+04	2.53E+04	3.60E+04
234	Caffeoylbenzoyltartaric acid	Phenylpropanoids	5.59E+03	3.66E+03	5.59E+03	9.00E+00	9.00E+00	9.00E+00	9.00E+00	9.00E+00	9.00E+00	1.36E+04	1.56E+04	1.55E+04
235	1,2-O-Diferuloylglycerol	Phenylpropanoids	5.05E+03	3.76E+03	5.05E+03	9.00E+00	9.00E+00	9.00E+00	9.00E+00	9.00E+00	9.00E+00	1.57E+04	1.43E+04	1.03E+04
236	Benzyl glucoside	Phenylpropanoids	4.07E+03	4.07E+03	4.07E+03	9.00E+00	9.00E+00	9.00E+00	9.00E+00	9.00E+00	9.00E+00	3.25E+04	9.00E+00	9.00E+00
237	Chlorogenic acid methyl ester	Phenylpropanoids	2.94E+03	2.14E+03	2.94E+03	9.00E+00	9.00E+00	9.00E+00	9.00E+00	9.00E+00	9.00E+00	7.41E+03	9.67E+03	6.38E+03
238	Sinapoyl malate	Phenylpropanoids	1.36E+04	1.32E+04	1.36E+04	9.00E+00	9.00E+00	9.00E+00	3.15E+04	3.10E+04	3.09E+04	6.48E+03	5.58E+03	3.22E+03
239	4-Hydroxybenzyl Alcohol	Phenylpropanoids	1.69E+03	1.18E+03	1.69E+03	9.00E+00	9.00E+00	9.00E+00	9.00E+00	9.00E+00	9.00E+00	5.19E+03	4.22E+03	4.03E+03
240	Methyl caffeate	Phenylpropanoids	5.03E+02	3.66E+02	5.03E+02	9.00E+00	9.00E+00	9.00E+00	9.00E+00	9.00E+00	9.00E+00	1.65E+03	1.23E+03	1.10E+03
241	Esculin	Phenylpropanoids	8.47E+04	5.94E+04	8.37E+04	2.24E+03	1.22E+04	1.13E+04	1.11E+04	1.05E+04	2.32E+03	2.18E+05	3.95E+05	9.58E+03
242	Neoglycyrol	Phenylpropanoids	1.40E+04	2.14E+04	2.78E+04	3.34E+03	5.36E+03	2.81E+03	1.81E+04	2.13E+03	2.28E+03	6.03E+03	6.71E+03	1.79E+05
243	4-Hydroxycinnamyl alcohol 4-D-glucoside	Phenylpropanoids	1.93E+04	1.68E+04	1.95E+04	2.51E+03	2.76E+03	4.55E+03	2.59E+04	2.70E+04	3.01E+04	2.36E+04	1.77E+04	2.46E+04
244	Salicin	Phenylpropanoids	1.27E+05	1.06E+05	1.28E+05	3.76E+04	3.28E+04	4.54E+04	1.02E+05	1.03E+05	1.01E+05	2.34E+05	1.96E+05	2.11E+05
245	Isoimperatorin	Phenylpropanoids	9.24E+05	8.15E+05	9.25E+05	2.02E+05	1.99E+05	2.12E+05	1.29E+06	1.26E+06	1.30E+06	1.00E+06	1.05E+06	1.08E+06
246	Osthole	Phenylpropanoids	2.63E+04	2.22E+04	2.60E+04	9.64E+03	9.26E+03	7.37E+03	2.00E+04	2.30E+04	2.28E+04	4.19E+04	4.39E+04	3.97E+04
247	7-Methoxy-5-Prenyloxycoumarin	Phenylpropanoids	3.05E+05	2.65E+05	3.03E+05	8.43E+04	7.17E+04	6.55E+04	4.23E+05	3.94E+05	4.15E+05	3.13E+05	3.56E+05	3.83E+05
248	1-O-(3,4-Dihydroxy-5-methoxy-benzoyl)-glucoside	Phenylpropanoids	1.48E+05	1.26E+05	1.48E+05	4.67E+04	4.73E+04	5.04E+04	1.38E+05	1.52E+05	1.45E+05	2.14E+05	2.16E+05	2.24E+05
249	Cinnamyl cinnamate	Phenylpropanoids	4.98E+04	4.43E+03	1.95E+04	9.10E+03	3.00E+03	9.61E+03	3.61E+04	5.61E+03	8.20E+03	6.62E+03	7.28E+04	1.40E+04
250	3-Galloylshikimic acid	Phenylpropanoids	6.79E+04	6.20E+04	6.71E+04	1.90E+04	1.02E+04	1.24E+04	1.15E+05	1.13E+05	1.08E+05	5.76E+04	6.11E+04	5.96E+04
251	3-Butylidenephthalide	Phenylpropanoids	1.28E+04	1.22E+04	1.02E+04	5.62E+03	3.55E+03	3.71E+03	3.63E+03	5.92E+03	1.06E+04	2.40E+04	2.52E+04	4.69E+03
252	Syringoylcaffeoylquinic acid-D-glucose	Phenylpropanoids	1.07E+04	8.30E+03	1.06E+04	5.40E+03	4.39E+03	4.38E+03	1.06E+04	6.93E+03	9.00E+00	2.06E+04	1.41E+04	2.37E+04
253	Vanillic acid-4-O-glucoside	Phenylpropanoids	2.37E+05	1.99E+05	2.38E+05	1.10E+05	1.31E+05	1.19E+05	1.24E+05	1.33E+05	1.40E+05	4.32E+05	4.06E+05	4.19E+05
254	3-O-p-Coumaroylquinic acid O-glucoside	Phenylpropanoids	6.75E+04	6.07E+04	6.69E+04	2.54E+04	1.87E+04	2.06E+04	7.31E+04	9.72E+04	1.01E+05	8.84E+04	6.17E+04	7.49E+04
255	Sinapic acid	Phenylpropanoids	2.01E+04	1.61E+04	1.92E+04	1.52E+04	1.13E+04	8.67E+03	3.92E+03	1.02E+04	5.10E+03	3.83E+04	3.60E+04	4.04E+04
256	3-Hydroxyphenylacetic acid	Phenylpropanoids	2.86E+05	2.25E+05	2.50E+05	5.71E+04	1.36E+05	1.62E+05	1.32E+05	1.87E+05	2.68E+05	5.74E+05	4.19E+05	1.19E+05
257	Protocatechuic acid-4-O-glucoside	Phenylpropanoids	1.30E+06	1.12E+06	1.30E+06	7.39E+05	7.61E+05	7.30E+05	6.89E+05	7.21E+05	7.50E+05	2.26E+06	2.30E+06	2.19E+06

258	3-(2,3-Dihydroxyphenyl)propanoate	Phenylpropanoids	2.99E+04	4.03E+04	4.36E+04	2.22E+04	3.69E+04	2.44E+04	2.29E+04	1.38E+04	1.44E+04	1.16E+05	3.64E+04	8.42E+04
259	1-O-Glucosyl sinapate	Phenylpropanoids	8.46E+04	7.45E+04	8.62E+04	5.64E+04	5.96E+04	7.00E+04	2.90E+04	3.88E+04	3.01E+04	1.50E+05	1.62E+05	1.51E+05
260	Veraguensin	Phenylpropanoids	6.27E+03	6.53E+03	2.77E+03	2.07E+03	1.49E+03	1.05E+03	2.03E+03	3.24E+03	3.34E+03	3.78E+03	1.28E+03	5.95E+03
261	3,4,5-Trimethoxyphenyl-1-O-Glucoside	Phenylpropanoids	1.15E+07	9.83E+06	1.15E+07	9.81E+06	9.50E+06	9.79E+06	1.71E+06	1.94E+06	2.02E+06	2.18E+07	2.21E+07	2.30E+07
262	3,5-Dihydroxy-4-methoxybenzoic acid	Phenylpropanoids	8.73E+05	8.07E+05	8.65E+05	4.50E+05	4.31E+05	3.90E+05	1.05E+06	1.06E+06	1.10E+06	1.04E+06	9.39E+05	9.15E+05
263	2,5-Dihydroxybenzoic acid O-glucoside	Phenylpropanoids	1.57E+06	1.37E+06	1.57E+06	1.11E+06	1.15E+06	1.14E+06	8.73E+05	9.48E+05	7.80E+05	2.44E+06	2.56E+06	2.67E+06
264	1-O-Vanilloyl-D-Glucose	Phenylpropanoids	1.89E+05	1.71E+05	1.89E+05	1.17E+05	1.12E+05	1.18E+05	1.67E+05	1.92E+05	1.53E+05	2.49E+05	2.58E+05	2.64E+05
265	5-Tricosyl-1,3-benzenediol	Phenylpropanoids	7.34E+03	7.47E+03	6.67E+03	1.00E+01	5.95E+03	6.37E+03	3.33E+03	7.15E+03	3.41E+03	1.62E+04	3.56E+03	7.44E+03
266	Trans-5-O-(p-Coumaroyl)shikimate	Phenylpropanoids	2.56E+05	2.45E+05	2.61E+05	1.15E+05	1.15E+05	1.55E+05	3.24E+05	2.77E+05	4.28E+05	2.67E+05	2.82E+05	2.43E+05
267	Dihydromethysticin	Phenylpropanoids	3.30E+04	5.11E+04	4.05E+04	1.80E+04	3.61E+04	2.78E+04	4.31E+04	2.44E+04	2.47E+04	3.54E+04	8.12E+04	5.11E+04
268	Psoralidin	Phenylpropanoids	1.21E+05	3.12E+05	1.49E+06	1.64E+04	3.68E+06	4.93E+04	1.40E+05	2.12E+05	2.04E+05	5.76E+04	1.62E+05	7.38E+06
269	1-O-[(E)-p-Coumaroyl]-D-glucose	Phenylpropanoids	1.55E+06	1.43E+06	1.54E+06	9.12E+05	8.09E+05	8.63E+05	1.58E+06	1.83E+06	2.02E+06	1.72E+06	1.68E+06	1.82E+06
270	Micromelin	Phenylpropanoids	6.61E+03	3.64E+03	3.88E+03	1.13E+03	3.07E+03	2.69E+03	3.55E+03	5.42E+03	2.42E+03	2.42E+03	9.01E+03	2.44E+03
271	3-(2-Hydroxyphenyl)-2-propenal	Phenylpropanoids	5.77E+03	1.71E+03	3.20E+03	8.44E+02	2.42E+03	2.58E+03	5.61E+03	2.11E+02	3.11E+03	2.90E+03	4.63E+03	4.12E+03
272	4'-Prenyloxyresveratrol	Phenylpropanoids	1.70E+04	1.18E+04	7.74E+03	5.39E+03	4.79E+03	5.49E+03	6.35E+03	9.82E+03	5.27E+03	1.22E+04	3.77E+03	1.42E+04
273	Dicaffeoylquinic acid-O-glucoside	Phenylpropanoids	1.10E+04	9.86E+03	1.07E+04	8.31E+03	5.64E+03	6.49E+03	1.11E+04	9.67E+03	1.47E+04	1.03E+04	1.26E+04	1.53E+04
274	Imperatorin	Phenylpropanoids	1.58E+04	1.06E+04	1.71E+04	2.77E+03	2.31E+04	1.23E+04	1.51E+04	8.86E+03	6.97E+03	1.93E+04	1.27E+04	3.84E+04
275	Rosmarinic acid	Phenylpropanoids	3.34E+03	1.02E+04	8.33E+03	3.34E+03	6.70E+03	7.94E+03	6.25E+03	9.53E+03	4.04E+03	7.15E+03	6.50E+03	1.85E+04
276	Matairesinol	Phenylpropanoids	5.31E+04	4.94E+04	5.30E+04	3.45E+04	3.57E+04	3.38E+04	5.58E+04	5.58E+04	5.71E+04	6.18E+04	6.03E+04	6.38E+04
277	Angelicin	Phenylpropanoids	7.69E+03	2.75E+03	3.52E+03	3.24E+03	2.49E+03	2.71E+03	1.32E+03	5.03E+03	1.75E+03	8.25E+03	5.47E+03	1.15E+03
278	Dihydrocaffeoylglucose	Phenylpropanoids	6.92E+05	6.41E+05	6.94E+05	5.05E+05	5.61E+05	5.24E+05	4.92E+05	5.82E+05	6.12E+05	8.62E+05	9.89E+05	9.30E+05
279	2-Hydroxy-3-(4-hydroxyphenyl)propenoic acid	Phenylpropanoids	2.77E+03	5.72E+03	4.81E+03	2.27E+03	3.66E+03	2.73E+03	4.76E+03	5.17E+03	7.20E+03	8.72E+03	3.45E+03	2.78E+03
280	Dimethyl Phthalate	Phenylpropanoids	1.16E+05	1.09E+05	1.15E+05	7.50E+04	6.32E+04	6.66E+04	1.38E+05	1.67E+05	1.29E+05	1.21E+05	1.14E+05	1.18E+05
281	Sphondin	Phenylpropanoids	5.00E+03	5.37E+02	3.17E+03	2.14E+03	3.31E+03	2.81E+03	1.07E+03	1.68E+03	2.36E+03	4.38E+03	9.18E+03	5.77E+02
282	Syringaldehyde-4-O-glucoside	Phenylpropanoids	7.18E+05	6.65E+05	7.08E+05	5.79E+05	6.31E+05	4.99E+05	4.91E+05	5.86E+05	6.51E+05	8.99E+05	9.87E+05	9.17E+05
283	3-O-p-Coumaroylquinic acid	Phenylpropanoids	6.61E+03	5.23E+03	6.53E+03	7.56E+03	8.27E+03	6.93E+03	9.00E+00	9.00E+00	9.00E+00	1.26E+04	6.39E+03	1.80E+04

284	3-Hydroxycoumarin	Phenylpropanoids	1.14E+04	5.11E+03	7.37E+03	8.94E+03	5.10E+03	3.59E+03	5.42E+03	7.62E+03	8.87E+03	5.23E+03	1.65E+04	6.63E+03
285	1-O-Salicyl-D-glucose	Phenylpropanoids	4.39E+06	4.11E+06	4.39E+06	3.85E+06	3.30E+06	3.90E+06	3.26E+06	3.68E+06	3.32E+06	5.49E+06	6.10E+06	6.10E+06
286	Coumarin	Phenylpropanoids	3.17E+03	3.06E+03	3.23E+03	1.98E+03	2.72E+03	2.43E+03	2.66E+03	3.03E+03	3.60E+03	3.63E+03	4.45E+03	3.33E+03
287	5-O-Caffeoylquinic acid (Neochlorogenic acid)	Phenylpropanoids	6.56E+04	6.01E+04	6.66E+04	4.79E+04	7.91E+04	5.59E+04	3.19E+04	3.63E+04	3.84E+04	1.02E+05	8.88E+04	1.00E+05
288	Notopterol	Phenylpropanoids	1.02E+04	2.34E+03	6.81E+03	9.01E+03	2.27E+03	3.85E+03	1.16E+04	3.41E+03	9.34E+03	7.58E+03	1.43E+04	2.16E+03
289	Ditartaroyl-hydroxycoumarin	Phenylpropanoids	1.42E+05	1.36E+05	1.44E+05	8.91E+04	9.44E+04	1.10E+05	1.63E+05	1.66E+05	1.57E+05	1.63E+05	1.48E+05	1.54E+05
290	Demethoxyencecalin	Phenylpropanoids	5.46E+04	4.02E+04	3.77E+04	4.04E+04	2.74E+04	3.43E+04	1.85E+04	3.70E+04	2.31E+04	8.71E+04	1.39E+04	6.03E+04
291	Scoparone	Phenylpropanoids	1.60E+04	1.16E+04	1.15E+04	6.67E+03	1.04E+04	1.05E+04	1.22E+04	6.29E+03	1.12E+04	5.11E+03	2.54E+04	1.12E+04
292	(-)-Salsoline	Phenylpropanoids	5.23E+03	8.42E+03	6.48E+03	4.81E+03	3.46E+03	2.99E+03	1.34E+04	9.80E+03	5.17E+03	6.56E+03	5.02E+03	5.42E+03
293	trans-3-Hydroxycinnamic acid	Phenylpropanoids	2.70E+04	2.22E+04	2.43E+04	1.12E+04	8.50E+03	3.14E+04	3.27E+04	1.84E+04	2.63E+04	3.59E+04	2.71E+04	1.40E+04
294	6-pentadecylsalicylic acid	Phenylpropanoids	3.42E+04	8.70E+03	2.94E+04	2.24E+04	2.28E+04	3.33E+04	1.57E+04	3.14E+04	1.50E+04	2.79E+04	5.89E+04	3.06E+04
295	Chlorogenic acid	Phenylpropanoids	7.79E+04	7.34E+04	7.99E+04	6.31E+04	8.30E+04	7.83E+04	5.56E+04	3.31E+04	5.35E+04	1.08E+05	1.13E+05	1.15E+05
296	Bergaptol	Phenylpropanoids	1.54E+03	1.85E+03	2.22E+03	1.90E+03	2.13E+03	2.16E+03	2.10E+03	6.86E+02	1.44E+03	1.94E+03	3.77E+03	3.50E+03
297	Torachrysone 8-O-glucoside	Phenylpropanoids	8.16E+03	1.41E+04	1.11E+04	6.55E+03	1.89E+04	2.92E+03	6.27E+03	1.33E+04	6.66E+03	1.09E+04	1.84E+04	1.18E+04
298	2,4,6-Trihydroxybenzoic acid	Phenylpropanoids	1.37E+06	1.31E+06	1.35E+06	1.36E+06	1.49E+06	1.25E+06	7.39E+05	6.68E+05	7.57E+05	2.12E+06	2.12E+06	1.67E+06
299	Atranorin	Phenylpropanoids	1.70E+04	1.43E+04	9.69E+03	2.21E+03	1.19E+04	1.38E+04	3.46E+03	1.70E+03	6.66E+03	1.81E+04	1.99E+04	2.03E+03
300	1-O-[(E)-Caffeoyl]-D-glucose	Phenylpropanoids	4.28E+06	4.06E+06	4.25E+06	4.03E+06	4.10E+06	3.81E+06	2.79E+06	2.84E+06	3.51E+06	6.07E+06	5.33E+06	5.60E+06
301	Coniferin	Phenylpropanoids	5.48E+06	5.17E+06	5.48E+06	5.22E+06	5.23E+06	5.22E+06	3.29E+06	3.91E+06	4.01E+06	7.44E+06	7.02E+06	7.75E+06
302	Pyroquilon	Phenylpropanoids	1.00E+01	9.91E+03	3.62E+03	1.10E+03	1.69E+03	4.48E+03	1.82E+03	6.35E+03	4.34E+03	1.13E+03	4.07E+03	5.08E+03
303	5-(2-Hydroxyethyl)-2-O-glucosylphenol	Phenylpropanoids	1.04E+06	1.01E+06	1.05E+06	7.02E+05	7.08E+05	7.30E+05	1.31E+06	1.30E+06	1.35E+06	1.04E+06	9.42E+05	1.00E+06
304	Isolariciresinol-9'-O-glucoside	Phenylpropanoids	3.19E+06	3.12E+06	3.20E+06	2.64E+06	2.57E+06	2.71E+06	3.13E+06	3.14E+06	3.10E+06	3.79E+06	3.83E+06	3.34E+06
305	Methyl benzoate	Phenylpropanoids	2.32E+03	4.71E+03	5.11E+03	3.29E+03	6.91E+03	3.62E+03	6.65E+03	3.47E+03	1.14E+03	2.28E+03	8.12E+03	8.65E+03
306	3,4-Dihydroxybenzeneacetic acid	Phenylpropanoids	9.68E+05	9.26E+05	9.78E+05	9.82E+05	1.00E+06	1.07E+06	5.34E+05	5.05E+05	5.34E+05	1.37E+06	1.42E+06	1.40E+06
307	6-O-Caffeoyl-D-glucose	Phenylpropanoids	4.35E+06	4.10E+06	4.32E+06	4.19E+06	4.18E+06	3.93E+06	2.93E+06	3.13E+06	3.55E+06	5.42E+06	5.45E+06	5.96E+06
308	Propylparaben	Phenylpropanoids	9.55E+03	3.99E+03	7.16E+03	5.94E+03	4.75E+03	4.41E+03	5.72E+03	1.12E+04	1.07E+04	7.02E+03	5.80E+03	7.67E+03
309	Mulberrofuran Q	Phenylpropanoids	8.02E+03	2.10E+04	3.98E+03	4.31E+03	2.83E+03	2.11E+03	4.50E+03	2.91E+03	7.14E+03	3.06E+03	4.31E+03	5.00E+03

310	Dalbergin	Phenylpropanoids	2.45E+03	1.65E+03	2.01E+03	2.52E+03	9.92E+02	1.33E+03	3.28E+03	1.36E+03	2.71E+03	3.65E+03	9.84E+02	1.76E+03
311	Trioxsalen	Phenylpropanoids	7.24E+03	1.96E+03	3.78E+03	1.00E+01	1.35E+03	5.77E+03	4.18E+03	5.12E+03	4.49E+03	3.25E+03	1.30E+03	4.78E+03
312	Safrole	Phenylpropanoids	4.14E+05	3.52E+05	7.88E+05	6.42E+05	4.94E+05	8.51E+05	5.35E+05	8.45E+05	9.76E+05	1.36E+06	7.20E+05	5.18E+05
313	p-Coumaric acid-4-O-glucoside	Phenylpropanoids	4.61E+05	4.42E+05	4.53E+05	3.97E+05	3.96E+05	3.31E+05	4.48E+05	5.35E+05	4.53E+05	4.62E+05	5.14E+05	4.85E+05
314	Enol-phenylpyruvate	Phenylpropanoids	2.71E+05	3.66E+05	3.02E+05	1.45E+05	3.55E+05	3.64E+05	1.96E+05	1.58E+05	2.24E+05	3.52E+05	4.01E+05	3.66E+05
315	6-O-Caffeoylarbutin	Phenylpropanoids	7.47E+04	6.95E+04	7.27E+04	6.77E+04	6.12E+04	5.11E+04	8.04E+04	7.84E+04	7.70E+04	9.03E+04	5.00E+04	9.28E+04
316	2-Hydroxy-3-methylbenzalpyruvate	Phenylpropanoids	5.16E+04	5.37E+04	6.42E+04	3.73E+04	4.34E+04	7.47E+04	5.74E+04	5.00E+04	8.68E+04	6.45E+04	6.73E+04	6.92E+04
317	Oleocanthal	Phenylpropanoids	3.75E+04	1.24E+04	2.65E+04	9.78E+03	2.14E+04	2.39E+04	2.62E+04	4.84E+04	2.19E+04	2.17E+04	2.71E+04	2.17E+04
318	Benzoin	Phenylpropanoids	3.39E+03	6.52E+03	4.45E+03	6.22E+03	4.57E+03	1.08E+03	5.21E+03	6.15E+03	3.45E+03	5.30E+03	4.33E+03	5.55E+03
319	3-O-p-Coumaroylshikimic acid	Phenylpropanoids	2.81E+04	2.74E+04	2.78E+04	1.85E+04	2.12E+04	1.62E+04	3.95E+04	2.53E+04	4.91E+04	2.55E+04	2.40E+04	2.18E+04
320	Trimethoprim	Phenylpropanoids	2.85E+06	3.23E+06	2.74E+06	1.18E+06	2.47E+06	4.22E+06	1.87E+06	1.47E+06	1.84E+06	3.16E+06	3.09E+06	3.78E+06
321	Esculetin	Phenylpropanoids	4.50E+06	4.39E+06	4.55E+06	4.24E+06	4.48E+06	4.67E+06	3.04E+06	3.23E+06	4.10E+06	5.68E+06	5.66E+06	5.53E+06
322	1'-O-(3,4-Dihydroxyphenethyl)-O-caffeoyl-glucoside	Phenylpropanoids	6.43E+04	6.09E+04	6.16E+04	7.13E+04	8.18E+04	4.96E+04	3.16E+04	4.18E+04	3.40E+04	9.00E+04	8.70E+04	7.69E+04
323	Decursin	Phenylpropanoids	4.44E+04	2.59E+04	2.26E+04	2.56E+04	1.69E+04	2.02E+04	2.82E+04	2.12E+04	1.54E+04	3.36E+04	1.75E+04	2.74E+04
324	Yangonin	Phenylpropanoids	8.12E+04	2.75E+04	3.35E+04	3.25E+04	3.17E+04	3.64E+04	2.81E+04	2.14E+04	2.48E+04	2.47E+04	6.73E+04	3.37E+04
325	3,4-Digalloylshikimic acid	Phenylpropanoids	1.44E+07	1.39E+07	1.43E+07	1.46E+07	1.49E+07	1.41E+07	9.98E+06	1.09E+07	1.05E+07	1.80E+07	1.80E+07	1.84E+07
326	5,7-Dimethoxycoumarin	Phenylpropanoids	2.32E+03	2.22E+03	2.32E+03	3.26E+03	3.24E+03	3.27E+03	9.00E+00	9.00E+00	9.00E+00	4.66E+03	3.31E+03	4.07E+03
327	1-(4-Hydroxyphenyl)propan-1-one	Phenylpropanoids	6.27E+03	1.15E+04	6.08E+03	7.64E+03	4.74E+03	2.29E+03	5.32E+03	6.86E+03	1.16E+04	9.49E+03	3.80E+03	4.56E+03
328	Coniferaldehyde	Phenylpropanoids	2.98E+05	2.97E+05	3.05E+05	2.87E+05	2.69E+05	3.48E+05	2.44E+05	2.03E+05	3.15E+05	3.53E+05	3.55E+05	3.54E+05
329	Cinnamaldehyde	Phenylpropanoids	1.06E+04	4.38E+03	6.97E+03	4.71E+03	7.83E+03	7.01E+03	5.25E+03	4.45E+03	8.32E+03	9.08E+03	7.42E+03	6.42E+03
330	Caffeic acid	Phenylpropanoids	1.31E+07	1.30E+07	1.32E+07	1.43E+07	1.34E+07	1.53E+07	8.19E+06	7.97E+06	1.09E+07	1.78E+07	1.64E+07	1.58E+07
331	Benzyl cinnamate	Phenylpropanoids	4.88E+03	3.54E+03	5.81E+03	6.58E+03	4.49E+03	5.50E+03	5.40E+03	7.69E+03	4.28E+03	5.62E+03	9.57E+03	3.92E+03
332	6-Methoxymellein	Phenylpropanoids	2.61E+03	4.67E+03	3.73E+03	3.31E+03	4.30E+03	2.42E+03	5.43E+03	2.61E+03	3.52E+03	2.17E+03	4.60E+03	4.78E+03
333	3-(4-Hydroxyphenyl)-1-propanol	Phenylpropanoids	7.32E+03	1.21E+04	1.29E+04	1.19E+04	9.15E+03	1.87E+04	1.41E+04	5.63E+03	9.87E+03	4.93E+03	2.08E+04	2.00E+04
334	1-O-Feruloylquinic acid	Phenylpropanoids	1.93E+06	1.90E+06	1.92E+06	1.45E+06	1.48E+06	1.44E+06	2.60E+06	2.60E+06	2.26E+06	1.77E+06	1.63E+06	1.63E+06
335	3-Ethyl-1,2-benzenediol	Phenylpropanoids	1.70E+04	1.33E+04	1.67E+04	1.12E+04	1.41E+04	1.40E+04	3.75E+04	1.10E+04	1.16E+04	1.36E+04	2.03E+04	1.12E+04

336	p-Hydroxymandelic acid	Phenylpropanoids	1.18E+04	8.59E+03	9.31E+03	7.39E+03	8.20E+03	7.32E+03	9.40E+03	1.34E+04	9.91E+03	9.16E+03	1.11E+04	6.02E+03
337	2,5-Dihydroxybenzaldehyde	Phenylpropanoids	3.49E+05	5.40E+05	3.98E+05	2.04E+05	3.98E+05	5.48E+05	3.10E+05	2.71E+05	3.40E+05	5.20E+05	4.39E+05	3.56E+05
338	Scopoletin-7-O-glucuronide	Phenylpropanoids	1.49E+06	1.48E+06	1.50E+06	1.11E+06	1.15E+06	1.15E+06	2.02E+06	1.94E+06	1.87E+06	1.26E+06	1.32E+06	1.30E+06
339	Euparin	Phenylpropanoids	3.89E+03	1.74E+03	9.30E+02	1.70E+03	1.17E+03	5.79E+02	1.00E+01	1.00E+01	1.75E+03	5.64E+02	5.79E+02	2.78E+03
340	Vanillic acid	Phenylpropanoids	2.46E+06	2.46E+06	2.52E+06	2.40E+06	2.35E+06	2.83E+06	2.07E+06	1.85E+06	2.51E+06	3.01E+06	2.66E+06	2.87E+06
341	N-p-Coumaroyl putrescine	Phenylpropanoids	5.64E+03	8.88E+03	4.78E+03	7.13E+03	5.34E+03	3.23E+03	4.39E+03	4.21E+03	3.44E+03	8.96E+03	5.07E+03	3.63E+03
342	3-O-Feruloylquinic acid	Phenylpropanoids	9.12E+04	9.11E+04	9.25E+04	9.91E+04	9.47E+04	1.09E+05	6.26E+04	6.24E+04	7.43E+04	1.14E+05	1.12E+05	1.10E+05
343	Phaseolic acid	Phenylpropanoids	2.51E+05	2.50E+05	2.47E+05	1.85E+05	1.80E+05	1.56E+05	3.30E+05	3.86E+05	3.49E+05	2.22E+05	1.87E+05	1.66E+05
344	Methyl rosmarinate	Phenylpropanoids	1.21E+04	9.01E+03	1.14E+04	5.42E+03	1.33E+04	1.02E+04	8.84E+03	1.44E+04	1.24E+04	3.53E+03	1.75E+04	1.07E+04
345	Maleoyl-caffeoylquinic acid	Phenylpropanoids	2.00E+04	2.00E+04	1.95E+04	2.90E+04	2.20E+04	2.53E+04	1.28E+04	8.36E+03	4.08E+03	2.94E+04	2.93E+04	2.49E+04
346	Sesamol	Phenylpropanoids	6.35E+03	5.31E+03	4.09E+03	4.92E+03	3.87E+03	4.80E+03	3.59E+03	2.32E+03	3.31E+03	4.15E+03	4.89E+03	5.77E+03
347	C-Veratroylglycol	Phenylpropanoids	3.46E+03	5.33E+03	6.02E+03	6.43E+03	4.51E+03	5.99E+03	5.03E+03	3.08E+03	1.11E+04	8.53E+03	4.29E+03	5.61E+03
348	threo-1-C-Syringylglycerol	Phenylpropanoids	5.67E+02	1.07E+04	1.23E+03	1.08E+03	1.17E+03	1.63E+03	1.11E+03	5.66E+02	1.13E+03	5.63E+02	1.16E+03	2.47E+03
349	7-(4-Hydroxyphenyl)-1-phenyl-4-hepten-3-one	Phenylpropanoids	3.04E+04	4.20E+04	5.54E+04	2.93E+04	4.30E+04	7.13E+04	4.41E+04	6.46E+04	6.50E+04	5.44E+04	4.23E+04	5.85E+04
350	p-Octopamine	Phenylpropanoids	2.97E+07	2.88E+07	2.82E+07	2.56E+07	2.56E+07	2.86E+07	2.92E+07	2.51E+07	3.21E+07	3.05E+07	2.66E+07	2.79E+07
351	Syringin	Phenylpropanoids	1.20E+05	1.18E+05	1.19E+05	9.25E+04	7.22E+04	8.18E+04	1.99E+05	1.84E+05	1.58E+05	6.97E+04	8.86E+04	9.82E+04
352	Vanillin	Phenylpropanoids	8.07E+06	8.25E+06	8.24E+06	7.95E+06	7.29E+06	9.31E+06	7.41E+06	6.96E+06	9.50E+06	9.01E+06	8.55E+06	7.90E+06
353	Chavicol	Phenylpropanoids	1.01E+05	1.00E+05	8.12E+04	4.21E+04	8.05E+04	1.24E+05	4.34E+04	6.75E+04	8.02E+04	9.91E+04	8.47E+04	7.01E+04
354	Trans-caffeic acid	Phenylpropanoids	9.94E+03	1.14E+04	1.06E+04	1.95E+04	7.19E+03	1.37E+04	5.01E+03	7.23E+03	9.84E+03	1.65E+04	1.50E+04	9.97E+03
355	Taxiphyllin	Phenylpropanoids	8.32E+03	6.78E+03	1.00E+04	1.35E+04	8.82E+03	8.21E+03	8.56E+03	1.30E+04	1.02E+04	7.74E+03	9.97E+03	1.37E+04
356	Phthalic anhydride	Phenylpropanoids	1.20E+06	1.23E+06	1.23E+06	1.16E+06	1.18E+06	1.47E+06	1.15E+06	1.13E+06	1.13E+06	1.18E+06	1.43E+06	1.21E+06
357	3-(3-Hydroxyphenyl)-propionate acid	Phenylpropanoids	1.53E+04	1.53E+04	1.53E+04	9.00E+00	9.00E+00	9.00E+00	4.64E+04	3.92E+04	3.71E+04	9.00E+00	9.00E+00	9.00E+00
358	Scopoletin-7-O-glucoside (Scopolin)	Phenylpropanoids	1.01E+04	1.01E+04	1.01E+04	9.00E+00	9.00E+00	9.00E+00	3.04E+04	3.10E+04	1.95E+04	9.00E+00	9.00E+00	9.00E+00
359	5-O-Caffeoylshikimic acid	Phenylpropanoids	2.03E+05	2.03E+05	2.03E+05	9.00E+00	9.00E+00	9.00E+00	5.26E+05	5.01E+05	5.99E+05	9.00E+00	9.00E+00	9.00E+00
360	1-Phenylethanol	Phenylpropanoids	3.92E+03	3.33E+03	4.89E+03	4.01E+03	6.74E+03	2.80E+03	9.18E+03	3.69E+03	3.14E+03	4.53E+03	4.94E+03	4.07E+03
361	Bromocriptine	Phenylpropanoids	7.54E+02	9.39E+03	6.34E+03	7.71E+03	7.54E+02	6.19E+03	1.26E+04	1.45E+04	2.19E+03	4.49E+03	2.26E+03	7.74E+03

362	Styrene-cis-2,3-dihydrodiol	Phenylpropanoids	8.78E+04	7.70E+04	7.34E+04	3.90E+04	7.79E+04	1.20E+05	4.71E+04	4.69E+04	6.17E+04	9.77E+04	5.03E+04	8.57E+04
363	2,6-Di-t-butylphenol	Phenylpropanoids	7.40E+05	7.32E+05	7.39E+05	7.41E+05	7.66E+05	7.28E+05	6.91E+05	7.14E+05	8.05E+05	6.41E+05	7.65E+05	7.98E+05
364	4-O-Glucosyl-4-hydroxybenzoic acid	Phenylpropanoids	2.74E+06	2.75E+06	2.74E+06	1.89E+06	1.78E+06	1.92E+06	4.55E+06	4.12E+06	4.05E+06	1.94E+06	1.75E+06	1.81E+06
365	2-phenylethyl- D-β- glucopyranoside	Phenylpropanoids	1.08E+06	1.09E+06	1.09E+06	5.40E+05	5.55E+05	5.85E+05	2.05E+06	1.97E+06	1.91E+06	5.46E+05	5.60E+05	5.34E+05
366	Bergamotine	Phenylpropanoids	1.85E+04	3.22E+04	4.22E+04	1.83E+04	4.60E+04	5.61E+04	4.13E+04	2.85E+04	4.85E+04	3.04E+04	4.56E+04	4.11E+04
367	Ethyl trans-p-methoxycinnamate	Phenylpropanoids	5.16E+04	3.73E+04	5.75E+04	4.11E+04	4.34E+04	7.47E+04	3.77E+04	6.31E+04	8.68E+04	2.46E+04	6.04E+04	6.92E+04
368	3-Methoxy-4,5-methylenedioxycinnamaldehyde	Phenylpropanoids	2.14E+04	4.27E+04	2.77E+04	3.24E+04	2.50E+04	3.52E+04	2.78E+04	1.96E+04	2.61E+04	3.27E+04	2.96E+04	2.58E+04
369	Daphnetin	Phenylpropanoids	5.28E+04	5.20E+04	5.26E+04	4.71E+04	6.13E+04	4.54E+04	5.12E+04	6.26E+04	5.43E+04	4.72E+04	4.73E+04	5.17E+04
370	4-O-(6'-O-Glucosylcaffeoylglucosyl)-4-hydroxybenzyl alcohol	Phenylpropanoids	4.71E+05	4.71E+05	4.70E+05	5.47E+05	6.20E+05	5.35E+05	3.17E+05	3.45E+05	3.26E+05	5.31E+05	5.49E+05	5.33E+05
371	Moracin C	Phenylpropanoids	8.98E+04	7.70E+04	1.25E+05	1.33E+05	1.52E+05	1.22E+05	1.34E+05	1.13E+05	9.86E+04	1.19E+05	1.53E+05	1.08E+05
372	10-Gingerol	Phenylpropanoids	1.49E+04	2.63E+04	2.26E+04	2.38E+04	3.89E+04	1.20E+04	1.65E+04	1.62E+04	2.81E+04	1.71E+04	4.57E+04	6.73E+03
373	3,4,5-Trimethoxycinnamyl alcohol	Phenylpropanoids	7.98E+04	6.00E+04	6.30E+04	6.51E+04	5.76E+04	6.97E+04	7.26E+04	7.07E+04	5.50E+04	7.13E+04	6.32E+04	4.42E+04
374	2,5-Dihydroxybenzoic acid	Phenylpropanoids	8.78E+06	9.04E+06	8.96E+06	9.29E+06	8.93E+06	1.07E+07	9.03E+06	7.62E+06	8.94E+06	9.31E+06	8.56E+06	8.59E+06
375	(S)-2-Hydroxy-3-(4-Hydroxyphenyl)Propanoic Acid	Phenylpropanoids	1.46E+05	1.48E+05	1.47E+05	2.88E+04	2.64E+04	3.79E+04	3.92E+05	3.28E+05	3.05E+05	3.77E+04	2.63E+04	2.08E+04
376	4-Isopropylbenzoic acid	Phenylpropanoids	8.86E+03	9.78E+03	1.12E+04	1.47E+04	1.18E+04	1.18E+04	1.03E+04	1.03E+04	1.02E+04	8.06E+03	7.12E+03	1.97E+04
377	Benzoic acid	Phenylpropanoids	9.40E+05	1.04E+06	6.61E+05	1.19E+06	1.03E+06	8.54E+04	7.70E+05	5.76E+05	7.32E+05	1.05E+06	4.27E+04	9.95E+05
378	3,4-Dihydroxybenzoic acid (Protocatechuic acid)	Phenylpropanoids	2.01E+07	2.08E+07	2.05E+07	2.08E+07	2.06E+07	2.46E+07	2.05E+07	1.85E+07	2.07E+07	2.12E+07	1.92E+07	1.90E+07
379	Terephthalic acid	Phenylpropanoids	5.23E+05	5.27E+05	5.30E+05	5.44E+05	5.39E+05	6.00E+05	5.85E+05	5.54E+05	4.49E+05	4.87E+05	4.62E+05	5.64E+05
380	2,3-Dihydroxybenzoic Acid	Phenylpropanoids	1.79E+07	1.85E+07	1.82E+07	2.01E+07	1.76E+07	2.22E+07	1.79E+07	1.64E+07	1.80E+07	1.81E+07	1.75E+07	1.77E+07
381	3,4-Dimethoxyphenyl acetic acid	Phenylpropanoids	5.34E+04	5.48E+04	5.43E+04	6.20E+04	5.95E+04	6.93E+04	4.37E+04	4.45E+04	5.13E+04	5.40E+04	5.38E+04	5.82E+04
382	Cannabinol	Phenylpropanoids	8.45E+03	1.55E+04	9.03E+03	6.08E+03	7.77E+03	1.03E+04	1.50E+04	8.59E+03	9.71E+03	4.00E+03	7.12E+03	9.79E+03
383	Coniferylaldehyde	Phenylpropanoids	5.72E+04	5.30E+04	5.63E+04	3.91E+04	7.54E+04	7.09E+04	2.89E+04	1.90E+04	9.58E+04	4.81E+04	4.76E+04	6.49E+04
384	4-Isopropylbenzaldehyde	Phenylpropanoids	1.24E+04	6.96E+03	6.47E+03	5.79E+03	9.12E+03	6.04E+03	6.17E+03	5.76E+03	6.59E+03	7.39E+03	4.09E+03	6.61E+03
385	2,4-Dimethylphenol	Phenylpropanoids	9.76E+03	1.04E+04	7.26E+03	1.75E+04	6.92E+03	6.16E+03	7.41E+03	6.37E+03	4.90E+03	1.25E+04	7.88E+03	5.96E+03
386	Epipinoresinol	Phenylpropanoids	9.91E+05	1.03E+06	1.02E+06	1.20E+06	1.12E+06	1.42E+06	7.51E+05	6.92E+05	9.39E+05	1.04E+06	1.09E+06	1.09E+06
387	Ferulic acid	Phenylpropanoids	9.69E+06	9.93E+06	9.69E+06	1.34E+07	1.30E+07	1.35E+07	4.94E+06	4.87E+06	6.93E+06	1.09E+07	1.18E+07	1.16E+07

388	4,7,9,9'-Tetrahydroxy-3,3'-dimethoxy-8-O-4'-neolignan	Phenylpropanoids	2.22E+05	2.31E+05	2.29E+05	2.12E+05	2.11E+05	2.72E+05	2.42E+05	2.46E+05	2.68E+05	1.93E+05	2.01E+05	2.01E+05
389	Metanephrine	Phenylpropanoids	1.33E+03	1.28E+03	2.06E+03	2.51E+03	2.54E+03	1.95E+03	2.38E+03	1.59E+03	2.03E+03	2.65E+03	2.02E+03	1.30E+03
390	Cardanol (C15:1)	Phenylpropanoids	8.47E+05	1.19E+05	3.19E+04	3.67E+04	3.28E+04	3.22E+04	3.83E+04	1.90E+04	4.77E+04	3.55E+04	2.22E+04	2.78E+04
391	Salidroside	Phenylpropanoids	1.42E+07	1.46E+07	1.44E+07	1.66E+07	1.73E+07	1.79E+07	1.43E+07	1.26E+07	1.06E+07	1.34E+07	1.40E+07	1.53E+07
392	Pinoresinol	Phenylpropanoids	9.62E+05	1.01E+06	9.90E+05	1.18E+06	1.12E+06	1.40E+06	7.63E+05	6.84E+05	9.01E+05	9.96E+05	1.03E+06	1.03E+06
393	trans-3,5-Dimethoxy-4-hydroxy cinnamaldehydee	Phenylpropanoids	1.01E+04	5.14E+03	4.43E+03	3.03E+03	6.49E+03	2.89E+03	3.53E+03	6.37E+03	6.03E+03	3.25E+03	3.31E+03	3.58E+03
394	5-O-p-Coumaroylshikimic acid O-glucoside	Phenylpropanoids	1.66E+04	1.67E+04	1.64E+04	2.37E+04	1.92E+04	2.17E+04	1.01E+04	1.48E+04	1.35E+04	1.47E+04	1.63E+04	2.08E+04
395	Methyl 2,4-dihydroxyphenylacetate	Phenylpropanoids	1.21E+05	1.21E+05	1.21E+05	2.92E+04	3.33E+04	2.91E+04	2.78E+05	2.78E+05	2.75E+05	1.75E+04	2.95E+04	2.56E+04
396	1-(alpha-Methyl-4-(2-methylpropyl)benzeneacetate)-beta-D-Glucopyranuronic acid	Phenylpropanoids	2.84E+04	1.09E+04	2.00E+04	2.26E+04	1.97E+04	2.09E+04	2.82E+04	2.91E+04	1.26E+04	1.59E+04	1.37E+04	1.98E+04
397	4'-Hydroxy-3'-methoxyacetophenone (Acetovanillone)	Phenylpropanoids	1.46E+04	1.54E+04	1.43E+04	2.26E+04	2.25E+04	2.04E+04	5.67E+03	6.28E+03	8.30E+03	1.85E+04	1.87E+04	1.40E+04
398	Isofraxidin	Phenylpropanoids	6.50E+03	5.44E+03	5.70E+03	2.94E+03	1.05E+04	7.09E+03	4.25E+03	3.30E+03	4.53E+03	1.71E+03	5.00E+03	9.22E+03
399	5-Hydroxyferulic acid methyl ester	Phenylpropanoids	5.69E+03	1.24E+04	1.47E+04	6.05E+03	1.57E+04	1.76E+04	1.88E+04	1.35E+04	2.21E+04	5.02E+03	5.74E+03	1.93E+04
400	Neogrifolin	Phenylpropanoids	5.45E+03	2.35E+03	2.66E+03	6.79E+03	2.35E+03	1.70E+03	2.24E+03	3.68E+03	3.06E+03	3.09E+03	3.18E+03	1.98E+03
401	alpha-Peltatin	Phenylpropanoids	1.41E+04	1.68E+04	1.34E+04	9.73E+03	2.62E+04	1.15E+04	1.24E+04	1.13E+04	1.03E+04	2.04E+04	5.52E+03	9.86E+03
402	alpha-Zearalenol	Phenylpropanoids	4.90E+04	1.80E+04	3.24E+04	3.61E+04	4.53E+04	4.26E+04	2.39E+04	1.42E+04	4.02E+04	1.43E+04	4.77E+04	3.12E+04
403	5-Heneicosylresorcinol	Phenylpropanoids	3.13E+03	1.48E+04	6.19E+03	8.80E+03	7.62E+03	8.32E+03	5.86E+03	6.11E+03	3.08E+03	7.39E+03	2.95E+03	8.18E+03
404	3,4-Dimethoxycinnamic acid	Phenylpropanoids	1.01E+05	1.09E+05	1.04E+05	1.40E+05	1.25E+05	1.64E+05	9.63E+04	8.24E+04	4.17E+04	1.15E+05	1.05E+05	1.01E+05
405	Sesartemin	Phenylpropanoids	1.45E+04	1.16E+05	9.21E+03	5.53E+03	1.33E+04	9.14E+03	4.60E+03	1.63E+04	9.62E+03	4.45E+03	6.70E+03	9.62E+03
406	4-Methylumbelliferyl acetate	Phenylpropanoids	3.59E+03	1.33E+03	3.18E+03	6.16E+03	2.92E+03	3.37E+03	4.23E+03	3.50E+03	2.26E+03	1.94E+03	4.58E+03	2.69E+03
407	Cinnamyl alcohol	Phenylpropanoids	9.03E+03	2.23E+04	1.15E+04	1.19E+04	9.42E+03	1.20E+04	2.51E+04	1.29E+04	8.34E+03	1.23E+04	6.12E+03	6.10E+03
408	Syringaldehyde	Phenylpropanoids	2.04E+04	2.10E+04	1.90E+04	3.29E+04	1.45E+04	2.17E+04	2.60E+04	2.21E+04	1.73E+04	1.91E+04	1.45E+04	1.69E+04
409	1-Caffeoylquinic acid	Phenylpropanoids	1.12E+04	3.82E+03	9.22E+03	3.86E+03	1.22E+04	1.75E+04	5.75E+03	9.14E+03	5.05E+03	1.77E+03	3.83E+03	1.85E+04
410	p-Coumaraldehyde	Phenylpropanoids	5.15E+04	5.21E+04	5.12E+04	5.73E+04	7.31E+04	5.49E+04	6.06E+04	4.16E+04	4.58E+04	4.60E+04	3.72E+04	5.01E+04
411	5'-Methoxyisolariciresinol-9'-O-glucoside	Phenylpropanoids	2.90E+05	3.07E+05	2.90E+05	4.09E+05	4.10E+05	4.11E+05	2.19E+05	1.97E+05	2.30E+05	3.17E+05	2.59E+05	2.78E+05
412	3,4-Dihydroxyphenylacetaldehyde	Phenylpropanoids	8.51E+03	8.11E+03	9.18E+03	1.80E+04	8.67E+03	9.98E+03	1.18E+04	5.79E+03	1.18E+04	5.57E+03	1.40E+04	5.80E+03

413	3-O-Digalloyl quinic acid	Phenylpropanoids	3.30E+05	3.44E+05	3.19E+05	5.90E+05	5.28E+05	4.98E+05	1.45E+05	1.39E+05	1.39E+05	3.49E+05	3.66E+05	3.86E+05
414	3,4,5-Tricaffeoylquinic acid	Phenylpropanoids	8.59E+04	9.28E+04	8.99E+04	1.09E+05	1.27E+05	1.42E+05	6.43E+04	5.64E+04	7.55E+04	8.27E+04	8.53E+04	8.63E+04
415	Schisandrol A	Phenylpropanoids	9.91E+03	3.95E+03	5.52E+03	1.37E+03	9.95E+03	3.76E+03	6.65E+03	6.76E+03	6.90E+03	1.00E+01	4.88E+03	5.22E+03
416	Salicylic acid	Phenylpropanoids	7.99E+06	8.71E+06	8.25E+06	1.01E+07	1.00E+07	1.22E+07	7.30E+06	5.57E+06	9.58E+06	7.58E+06	7.37E+06	6.40E+06
417	Venlafaxine	Phenylpropanoids	5.27E+03	4.34E+03	5.62E+03	4.08E+03	5.75E+03	9.51E+03	1.07E+04	3.66E+03	2.64E+03	3.38E+03	4.22E+03	5.16E+03
418	3-Hydroxy-4-methoxycinnamic acid	Phenylpropanoids	2.06E+04	1.15E+04	1.42E+04	1.57E+04	2.93E+04	1.42E+04	1.50E+04	1.11E+04	4.73E+03	1.32E+04	1.10E+04	1.47E+04
419	Acetic acid m-cresyl ester	Phenylpropanoids	3.46E+03	3.30E+03	4.88E+03	6.93E+03	7.49E+03	6.10E+03	2.91E+03	4.71E+03	4.59E+03	3.20E+03	6.75E+03	3.31E+03
420	Guaiacol	Phenylpropanoids	1.18E+04	1.28E+04	1.09E+04	1.79E+04	1.83E+04	1.21E+04	9.94E+03	5.02E+03	1.15E+04	1.20E+04	8.55E+03	1.00E+04
421	Feruloylmalic acid	Phenylpropanoids	1.41E+04	1.43E+04	1.41E+04	7.87E+03	1.31E+04	8.13E+03	2.02E+04	3.85E+04	1.50E+04	6.58E+03	4.85E+03	6.65E+03
422	4-Methoxycinnamic acid	Phenylpropanoids	1.56E+05	1.65E+05	1.55E+05	1.88E+05	1.66E+05	1.81E+05	2.17E+05	2.24E+05	1.24E+05	1.12E+05	1.11E+05	1.08E+05
423	(+)-Lyoniresinol 9'-O-glucoside	Phenylpropanoids	5.79E+03	7.67E+03	3.14E+03	5.41E+03	4.55E+03	4.48E+03	2.06E+03	2.97E+03	2.20E+03	3.93E+03	1.00E+01	4.93E+03
424	Methyl ferulate	Phenylpropanoids	2.05E+05	2.24E+05	2.08E+05	3.08E+05	2.79E+05	3.38E+05	2.10E+05	2.00E+05	8.56E+04	1.93E+05	1.75E+05	1.87E+05
425	4-MethoxycinnaMaldehyde	Phenylpropanoids	9.25E+04	9.95E+04	9.19E+04	1.37E+05	1.37E+05	1.32E+05	7.00E+04	7.36E+04	7.99E+04	8.24E+04	8.47E+04	7.59E+04
426	Yatein	Phenylpropanoids	1.04E+04	1.25E+04	8.88E+03	8.03E+03	1.33E+04	1.25E+04	1.37E+04	5.84E+03	5.61E+03	1.02E+04	6.67E+03	3.27E+03
427	5-Hydroxyconiferaldehyde	Phenylpropanoids	5.18E+03	5.14E+03	6.44E+03	6.05E+03	9.88E+03	8.35E+03	6.38E+03	8.70E+03	4.07E+03	3.20E+03	7.27E+03	3.69E+03
428	Methyl 4-hydroxycinnamate	Phenylpropanoids	2.22E+05	2.37E+05	2.25E+05	2.30E+05	2.23E+05	2.56E+05	3.24E+05	3.72E+05	2.13E+05	1.49E+05	1.31E+05	1.32E+05
429	Alloimperatorin	Phenylpropanoids	6.50E+03	4.67E+03	8.14E+03	8.57E+03	7.14E+03	1.23E+04	8.03E+03	1.39E+04	7.52E+03	7.48E+03	4.05E+03	4.66E+03
430	Isoeugenol	Phenylpropanoids	2.53E+03	2.82E+03	2.54E+03	5.19E+03	5.67E+03	5.30E+03	9.00E+00	9.00E+00	9.00E+00	2.85E+03	3.49E+03	2.98E+03
431	2',4'-Dihydroxy-6'-methoxyacetophenone	Phenylpropanoids	6.30E+05	6.90E+05	6.24E+05	1.19E+06	1.16E+06	1.14E+06	2.56E+05	2.51E+05	2.50E+05	6.19E+05	6.60E+05	6.60E+05
432	Brevifolin carboxylic acid	Phenylpropanoids	6.55E+06	7.22E+06	6.54E+06	1.11E+07	1.12E+07	1.11E+07	3.94E+06	4.35E+06	3.88E+06	6.38E+06	5.89E+06	5.64E+06
433	Hydrocinnamic acid	Phenylpropanoids	1.04E+04	1.30E+04	9.66E+03	5.68E+03	2.53E+04	8.34E+03	1.07E+04	7.69E+03	4.16E+03	7.40E+03	3.36E+03	1.03E+04
434	Protocatechuic aldehyde	Phenylpropanoids	2.23E+06	2.51E+06	2.31E+06	3.45E+06	3.53E+06	4.08E+06	1.81E+06	1.48E+06	1.77E+06	2.02E+06	1.96E+06	1.84E+06
435	Mandelic acid	Phenylpropanoids	9.98E+04	1.05E+05	9.92E+04	9.94E+04	9.17E+04	9.48E+04	1.66E+05	1.41E+05	1.52E+05	4.00E+04	5.45E+04	5.41E+04
436	p-Coumaric acid	Phenylpropanoids	1.42E+07	1.59E+07	1.47E+07	1.86E+07	1.85E+07	2.30E+07	1.54E+07	1.17E+07	1.83E+07	1.09E+07	1.05E+07	9.51E+06
437	Brevifolin	Phenylpropanoids	2.34E+04	2.49E+04	2.13E+04	4.80E+04	3.82E+04	3.16E+04	1.38E+04	1.36E+04	1.38E+04	2.05E+04	1.93E+04	1.98E+04
438	p-Coumaroylquinic acid-4'-O-glucuronide	Phenylpropanoids	4.90E+04	5.39E+04	4.99E+04	7.40E+04	7.39E+04	8.06E+04	4.16E+04	5.07E+04	3.72E+04	3.80E+04	3.49E+04	4.19E+04

439	3-hydroxybenzaldehyde	Phenylpropanoids	5.79E+04	6.08E+04	5.56E+04	9.76E+04	8.60E+04	7.86E+04	4.70E+04	5.31E+04	5.15E+04	3.60E+04	3.68E+04	5.56E+04
440	2-Hydroxy-3-carboxybenzalpyruvate	Phenylpropanoids	5.69E+03	1.03E+04	7.53E+03	1.39E+04	2.31E+04	4.69E+03	1.87E+03	5.93E+03	4.71E+03	4.78E+03	7.46E+03	7.73E+03
441	3-Methyl-4,8-dihydroxy-3,4-dihydroisocoumarin	Phenylpropanoids	2.23E+04	2.34E+04	2.21E+04	2.64E+04	1.83E+04	2.46E+04	3.08E+04	3.20E+04	3.76E+04	1.10E+04	6.38E+03	1.58E+04
442	4-Hydroxybenzoic acid	Phenylpropanoids	2.02E+06	2.30E+06	2.10E+06	3.21E+06	3.37E+06	3.85E+06	1.70E+06	1.30E+06	1.64E+06	1.79E+06	1.57E+06	1.58E+06
443	Schizandrin A	Phenylpropanoids	1.23E+04	1.20E+04	7.98E+03	1.80E+04	8.62E+03	1.33E+04	5.84E+03	1.13E+04	5.93E+03	4.35E+03	7.80E+03	6.66E+03
444	6-O-Feruloyl-D-glucose	Phenylpropanoids	3.22E+04	3.53E+04	3.20E+04	5.94E+04	6.11E+04	5.76E+04	2.16E+04	1.69E+04	1.59E+04	2.15E+04	2.83E+04	3.31E+04
445	Mellein	Phenylpropanoids	1.08E+04	9.13E+03	8.09E+03	6.93E+03	1.37E+04	1.81E+04	5.25E+03	7.49E+03	3.73E+03	8.97E+03	5.42E+03	2.03E+03
446	Dexmedetomidine	Phenylpropanoids	2.26E+03	4.04E+03	1.30E+03	3.94E+03	1.15E+03	2.32E+03	2.27E+03	1.69E+03	1.00E+01	5.57E+02	2.36E+03	1.00E+01
447	Salicylic acid-2-O-glucoside	Phenylpropanoids	1.42E+07	1.58E+07	1.36E+07	2.81E+07	2.78E+07	2.32E+07	8.34E+06	9.30E+06	9.13E+06	9.61E+06	1.10E+07	1.04E+07
448	4-Acetylphenyl-glucoside (Picein)	Phenylpropanoids	4.10E+04	4.66E+04	4.32E+04	4.84E+04	4.85E+04	6.66E+04	6.31E+04	6.02E+04	4.33E+04	2.03E+04	2.23E+04	2.16E+04
449	Syringic acid	Phenylpropanoids	3.31E+04	3.78E+04	3.27E+04	6.77E+04	7.30E+04	6.47E+04	1.55E+04	1.54E+04	1.35E+04	2.63E+04	2.63E+04	2.69E+04
450	p-Coumaryl alcohol	Phenylpropanoids	1.90E+06	2.17E+06	1.90E+06	3.63E+06	3.57E+06	3.62E+06	1.33E+06	1.27E+06	1.22E+06	1.33E+06	1.42E+06	1.43E+06
451	Gastrodin	Phenylpropanoids	4.79E+03	4.59E+03	3.30E+03	3.04E+03	3.84E+03	7.21E+03	3.42E+03	4.58E+03	2.20E+03	1.23E+03	1.81E+03	2.11E+03
452	Phenylpyruvic acid	Phenylpropanoids	3.11E+06	3.64E+06	3.25E+06	4.92E+06	4.52E+06	6.03E+06	3.29E+06	2.42E+06	4.08E+06	1.96E+06	1.92E+06	1.77E+06
453	7-Hydroxy-5-methoxycoumarin (Scopoletin)	Phenylpropanoids	6.16E+04	6.57E+04	5.90E+04	7.48E+04	7.71E+04	5.43E+04	9.47E+04	9.10E+04	8.19E+04	2.59E+04	2.57E+04	2.16E+04
454	Mulberrofuran A	Phenylpropanoids	4.74E+03	1.51E+03	5.65E+03	1.67E+03	2.14E+04	3.85E+03	2.69E+03	3.99E+03	3.71E+03	1.31E+03	4.78E+03	3.46E+03
455	4-Hydroxybenzaldehyde	Phenylpropanoids	7.79E+05	9.09E+05	8.25E+05	1.02E+06	1.13E+06	1.39E+06	1.05E+06	7.65E+05	1.15E+06	3.88E+05	3.78E+05	3.45E+05
456	2-Hydroxycinnamic acid	Phenylpropanoids	7.29E+06	8.61E+06	7.54E+06	1.20E+07	1.13E+07	1.40E+07	7.65E+06	5.28E+06	1.07E+07	4.15E+06	3.77E+06	3.46E+06
457	Tyrosol	Phenylpropanoids	4.57E+06	5.17E+06	4.64E+06	6.01E+06	6.20E+06	6.52E+06	6.41E+06	6.36E+06	6.09E+06	1.88E+06	1.90E+06	1.74E+06
458	Schizandrin C	Phenylpropanoids	1.00E+01	8.98E+03	1.62E+03	1.00E+01	4.28E+03	2.45E+03	2.13E+03	4.94E+02	1.63E+03	4.73E+02	9.82E+02	4.86E+02
459	8-Geranyloxypsoralen	Phenylpropanoids	1.23E+06	1.31E+06	2.91E+06	1.95E+06	1.39E+07	3.82E+05	1.37E+06	1.93E+06	1.44E+06	1.34E+06	1.44E+06	1.49E+06
460	3-(4-Hydroxyphenyl)-propionic acid	Phenylpropanoids	7.55E+05	9.44E+05	8.03E+05	1.54E+06	1.49E+06	1.93E+06	5.62E+05	4.21E+05	8.22E+05	4.05E+05	3.76E+05	4.18E+05
461	Caffeoyl(p-Hydroxybenzoyl)tartaric acid	Phenylpropanoids	1.55E+05	1.84E+05	1.49E+05	3.55E+05	3.54E+05	3.07E+05	1.19E+05	1.04E+05	8.82E+04	8.02E+04	6.75E+04	7.15E+04
462	4-Hydroxyacetophenone	Phenylpropanoids	2.36E+05	2.95E+05	2.26E+05	6.41E+05	5.49E+05	5.62E+05	1.29E+05	1.31E+05	1.27E+05	1.15E+05	1.08E+05	9.10E+04
463	p-Hydroxyphenyl acetic acid	Phenylpropanoids	7.50E+05	7.99E+05	7.46E+05	4.81E+05	5.03E+05	4.50E+05	1.69E+06	1.62E+06	1.50E+06	7.46E+04	7.74E+04	6.06E+04
464	3-Hydroxy-4-isopropylbenzylalcohol-3-O-glucoside	Phenylpropanoids	9.48E+04	1.20E+05	8.84E+04	2.93E+05	2.55E+05	2.42E+05	3.39E+04	3.06E+04	3.55E+04	3.41E+04	3.78E+04	3.85E+04

465	Cinnamic acid	Phenylpropanoids	3.83E+04	4.86E+04	3.79E+04	9.76E+04	1.03E+05	9.45E+04	2.24E+04	2.02E+04	2.42E+04	1.31E+04	1.38E+04	1.21E+04
466	Cinnamyl acetat	Phenylpropanoids	2.91E+04	4.94E+04	2.87E+04	4.68E+04	1.40E+05	3.34E+04	6.89E+03	4.81E+03	1.82E+04	8.53E+03	1.40E+04	3.89E+03
467	Acanthoside B	Phenylpropanoids	1.00E+01	6.25E+03	1.21E+02	1.00E+01	4.56E+02	4.51E+02	1.00E+01	1.00E+01	1.00E+01	1.00E+01	1.00E+01	1.00E+01
468	N-Acetyl-L-methionine	Amino acids	1.44E+04	9.52E+03	1.44E+04	9.00E+00	9.00E+00	9.00E+00	9.00E+00	9.00E+00	9.00E+00	3.59E+04	4.03E+04	3.88E+04
469	S-(5'-Adenosy)-L-homocysteine	Amino acids	1.66E+04	1.19E+04	1.66E+04	9.00E+00	9.00E+00	9.00E+00	9.00E+00	1.14E+04	1.80E+04	3.29E+04	3.25E+04	3.82E+04
470	(5-L-Glutamyl)-L-amino acid	Amino acids	1.02E+04	8.63E+03	1.02E+04	9.00E+00	9.00E+00	9.00E+00	1.65E+04	1.78E+04	1.03E+04	1.31E+04	1.14E+04	1.25E+04
471	N-(3-Indolylacetyl)-L-alanine	Amino acids	8.23E+03	7.77E+03	8.23E+03	9.00E+00	9.00E+00	9.00E+00	1.78E+04	1.83E+04	1.99E+04	2.88E+03	3.27E+03	3.71E+03
472	L-Methionine	Amino acids	1.05E+06	7.35E+05	1.06E+06	4.55E+04	4.32E+04	6.08E+04	1.20E+05	1.10E+05	1.15E+05	2.60E+06	2.79E+06	2.61E+06
473	L-Prolyl-L-Leucine	Amino acids	1.14E+06	8.13E+05	1.14E+06	1.53E+05	1.68E+05	1.53E+05	1.81E+05	1.30E+05	1.64E+05	2.70E+06	2.86E+06	2.79E+06
474	L-Isoleucyl-L-Aspartate	Amino acids	1.91E+05	1.43E+05	1.91E+05	4.53E+04	3.88E+04	4.41E+04	5.97E+04	5.70E+04	6.34E+04	3.84E+05	4.49E+05	4.30E+05
475	Homoarginine	Amino acids	1.63E+05	1.23E+05	1.62E+05	4.35E+04	4.18E+04	3.74E+04	6.25E+04	5.62E+04	6.29E+04	3.21E+05	3.62E+05	3.55E+05
476	4-Aminobutyric acid	Amino acids	1.00E+01	5.56E+04	4.08E+04	8.00E+03	1.46E+04	1.00E+01	3.88E+04	4.42E+04	4.70E+04	4.11E+04	6.82E+04	7.24E+04
477	L-Arginine	Amino acids	1.66E+07	1.25E+07	1.66E+07	4.78E+06	5.15E+06	4.87E+06	4.99E+06	4.56E+06	5.39E+06	3.55E+07	3.45E+07	3.79E+07
478	N-α-Acetyl-L-ornithine	Amino acids	2.05E+06	1.57E+06	2.05E+06	6.62E+05	6.15E+05	6.11E+05	4.82E+05	4.21E+05	5.19E+05	4.56E+06	4.70E+06	4.46E+06
479	L-Aspartyl-L-Phenylalanine	Amino acids	9.15E+04	7.22E+04	9.19E+04	2.25E+04	3.11E+04	2.61E+04	4.66E+04	4.57E+04	3.34E+04	1.79E+05	1.93E+05	1.80E+05
480	Oxiglutatione	Amino acids	5.70E+04	4.79E+04	5.67E+04	1.50E+04	1.69E+04	1.25E+04	4.61E+04	4.49E+04	4.67E+04	9.81E+04	1.03E+05	8.51E+04
481	L-Glutamine	Amino acids	1.07E+06	8.35E+05	1.07E+06	3.77E+05	3.90E+05	3.40E+05	2.68E+05	2.54E+05	2.87E+05	2.38E+06	2.38E+06	2.25E+06
482	L-Lysine	Amino acids	1.16E+06	9.10E+05	1.16E+06	4.01E+05	3.96E+05	4.21E+05	3.14E+05	2.68E+05	2.91E+05	2.70E+06	2.49E+06	2.40E+06
483	L-Phenylalanine	Amino acids	7.29E+06	5.54E+06	7.34E+06	2.40E+06	2.77E+06	2.78E+06	1.89E+06	1.81E+06	1.86E+06	1.53E+07	1.55E+07	1.68E+07
484	L-Glycyl-L-phenylalanine	Amino acids	1.75E+05	1.44E+05	1.74E+05	5.70E+04	3.82E+04	4.60E+04	1.46E+05	1.65E+05	1.58E+05	2.77E+05	2.63E+05	2.96E+05
485	L-Tryptophan	Amino acids	1.48E+07	1.13E+07	1.47E+07	6.28E+06	5.83E+06	5.71E+06	2.29E+06	2.23E+06	2.18E+06	3.25E+07	3.37E+07	3.35E+07
486	L-Isoleucine	Amino acids	5.81E+05	4.43E+05	5.77E+05	2.45E+05	2.27E+05	2.13E+05	1.41E+05	1.50E+05	1.47E+05	1.10E+06	1.33E+06	1.31E+06
487	L-Leucine	Amino acids	5.74E+05	4.47E+05	5.74E+05	2.24E+05	2.42E+05	2.25E+05	1.59E+05	1.31E+05	1.44E+05	1.15E+06	1.31E+06	1.24E+06
488	5-Oxo-L-Proline	Amino acids	4.09E+04	3.39E+04	4.20E+04	1.27E+04	1.50E+04	2.15E+04	2.10E+04	1.88E+04	1.70E+04	8.15E+04	8.40E+04	7.71E+04
489	L-Valine	Amino acids	3.60E+06	2.84E+06	3.60E+06	1.57E+06	1.53E+06	1.54E+06	1.14E+06	1.06E+06	1.08E+06	7.35E+06	7.43E+06	7.68E+06
490	L-Glycyl-L-isoleucine	Amino acids	3.57E+05	3.05E+05	3.58E+05	1.11E+05	1.06E+05	1.12E+05	3.44E+05	3.96E+05	3.07E+05	5.10E+05	5.55E+05	5.30E+05

491	N-Glycyl-L-leucine	Amino acids	4.69E+05	3.98E+05	4.71E+05	1.33E+05	1.59E+05	1.53E+05	4.21E+05	4.76E+05	4.47E+05	6.56E+05	7.38E+05	7.20E+05
492	Cycloleucine	Amino acids	8.14E+06	6.45E+06	8.12E+06	3.68E+06	3.68E+06	3.52E+06	2.25E+06	2.00E+06	2.22E+06	1.74E+07	1.68E+07	1.70E+07
493	5-Hydroxy-L-tryptophan	Amino acids	2.71E+04	2.20E+04	2.68E+04	1.28E+04	1.13E+04	1.10E+04	1.40E+04	1.08E+04	1.19E+04	5.10E+04	5.34E+04	5.13E+04
494	L-Citrulline	Amino acids	4.81E+05	3.93E+05	4.80E+05	2.50E+05	2.43E+05	2.36E+05	1.05E+05	1.09E+05	1.12E+05	1.08E+06	1.01E+06	9.44E+05
495	3-Aminoisobutanoic acid	Amino acids	1.00E+01	3.70E+03	9.49E+03	1.00E+01	6.26E+03	2.43E+03	8.63E+02	8.75E+02	3.25E+04	1.00E+01	1.00E+01	3.30E+04
496	L-Aspartic Acid	Amino acids	1.12E+06	9.18E+05	1.11E+06	6.20E+05	5.91E+05	5.45E+05	4.88E+05	3.92E+05	4.34E+05	2.09E+06	2.19E+06	2.14E+06
497	L-Histidine	Amino acids	2.23E+06	1.90E+06	2.23E+06	1.16E+06	1.05E+06	1.19E+06	1.14E+06	1.18E+06	1.10E+06	4.00E+06	4.38E+06	3.82E+06
498	S-(Methyl)glutathione	Amino acids	2.27E+05	1.97E+05	2.26E+05	1.01E+05	1.25E+05	9.39E+04	1.39E+05	1.44E+05	1.96E+05	4.26E+05	3.52E+05	3.31E+05
499	L-Aspartic acid-O-diglucoside	Amino acids	6.18E+05	5.46E+05	6.20E+05	2.91E+05	2.85E+05	3.06E+05	5.12E+05	4.81E+05	5.43E+05	1.06E+06	8.86E+05	8.85E+05
500	L-Tyrosine	Amino acids	7.73E+06	6.40E+06	7.66E+06	4.88E+06	4.80E+06	4.34E+06	3.28E+06	2.99E+06	3.10E+06	1.33E+07	1.45E+07	1.50E+07
501	N-Acetyl-L-Tryptophan	Amino acids	1.39E+05	1.23E+05	1.40E+05	5.75E+04	6.85E+04	7.19E+04	1.22E+05	1.32E+05	1.35E+05	2.01E+05	1.94E+05	1.99E+05
502	L-Ornithine	Amino acids	1.96E+05	1.68E+05	1.94E+05	1.32E+05	1.27E+05	1.13E+05	8.02E+04	7.93E+04	8.63E+04	3.83E+05	3.42E+05	3.42E+05
503	L-Asparagine	Amino acids	3.69E+06	3.16E+06	3.67E+06	2.42E+06	2.64E+06	2.27E+06	1.50E+06	1.32E+06	1.36E+06	7.53E+06	6.28E+06	6.49E+06
504	Pipecolic acid	Amino acids	5.10E+06	4.65E+06	5.06E+06	2.31E+06	2.10E+06	2.02E+06	6.28E+06	6.53E+06	6.04E+06	5.84E+06	6.06E+06	5.63E+06
505	5-Aminovaleric acid	Amino acids	2.76E+04	2.24E+04	2.77E+04	1.67E+04	1.83E+04	1.81E+04	8.83E+03	1.67E+04	1.72E+04	4.17E+04	4.16E+04	5.95E+04
506	N-Acetyl-L-Aspartic Acid	Amino acids	1.13E+05	9.98E+04	1.13E+05	7.70E+04	7.48E+04	7.03E+04	5.20E+04	6.07E+04	5.60E+04	2.04E+05	2.04E+05	1.79E+05
507	N,N-Dimethylglycine	Amino acids	9.41E+06	8.05E+06	9.37E+06	7.02E+06	6.70E+06	6.69E+06	2.98E+06	2.84E+06	2.82E+06	1.77E+07	1.76E+07	1.76E+07
508	S-(Phenylacetothiohydroximoyl)-L-cysteine	Amino acids	5.80E+03	1.11E+04	8.29E+03	4.07E+03	3.48E+03	6.94E+03	3.94E+03	1.03E+04	1.32E+04	6.00E+03	7.10E+03	1.53E+04
509	beta-Alanine	Amino acids	1.74E+05	1.16E+05	1.83E+05	8.36E+04	2.24E+05	7.23E+04	2.34E+05	1.06E+05	1.03E+05	2.40E+05	3.66E+05	1.20E+05
510	L-Alanyl-L-Phenylalanine	Amino acids	8.50E+04	7.89E+04	8.68E+04	6.77E+04	8.24E+04	8.20E+04	5.30E+04	4.16E+04	4.96E+04	1.18E+05	1.37E+05	1.31E+05
511	L-Valyl-L-Leucine	Amino acids	6.10E+05	5.84E+05	6.23E+05	3.80E+05	4.49E+05	4.83E+05	6.39E+05	6.85E+05	6.23E+05	6.99E+05	7.13E+05	6.93E+05
512	S-Adenosyl-L-methionine	Amino acids	1.21E+04	1.10E+04	1.23E+04	8.43E+03	8.40E+03	9.95E+03	1.24E+04	1.37E+04	1.30E+04	1.13E+04	1.07E+04	1.92E+04
513	D-Serine	Amino acids	6.92E+04	4.73E+04	6.29E+04	5.45E+04	5.24E+04	5.08E+04	5.87E+04	5.26E+04	5.05E+04	4.64E+04	1.00E+05	9.11E+04
514	3-Methyl-L-Histidine	Amino acids	2.50E+04	2.35E+04	2.54E+04	2.09E+04	3.00E+04	2.36E+04	1.48E+04	1.60E+04	9.28E+03	3.65E+04	3.67E+04	3.61E+04
515	N-Acetyl-L-Glutamine	Amino acids	2.13E+05	2.05E+05	2.11E+05	1.12E+05	1.08E+05	1.01E+05	3.54E+05	3.33E+05	3.35E+05	1.45E+05	1.52E+05	1.61E+05
516	N6-Acetyl-L-lysine	Amino acids	5.37E+04	5.26E+04	5.31E+04	5.04E+04	4.41E+04	4.54E+04	4.62E+04	5.43E+04	4.70E+04	7.16E+04	6.15E+04	5.43E+04

517	Nicotinurate	Amino acids	9.38E+03	1.01E+04	1.28E+04	1.73E+04	1.27E+04	7.12E+03	9.11E+03	9.30E+03	1.48E+04	1.06E+04	1.88E+04	2.01E+04
518	L-Homoserine	Amino acids	1.45E+06	1.38E+06	1.44E+06	1.57E+06	1.65E+06	1.55E+06	6.90E+05	6.91E+05	7.06E+05	2.09E+06	2.06E+06	2.11E+06
519	L-Leucyl-L-phenylalanine	Amino acids	2.12E+05	2.02E+05	2.11E+05	1.65E+05	1.65E+05	1.54E+05	2.36E+05	2.59E+05	2.52E+05	1.70E+05	2.14E+05	2.37E+05
520	Proline betaine	Amino acids	2.48E+04	1.59E+04	3.91E+04	3.91E+04	2.56E+04	4.76E+04	1.70E+04	3.83E+04	4.24E+04	5.02E+04	4.67E+04	4.51E+04
521	L-Phenylalanyl-L-phenylalanine	Amino acids	1.27E+04	1.30E+04	1.32E+04	6.97E+03	9.99E+03	1.07E+04	1.79E+04	1.57E+04	1.70E+04	1.16E+04	1.39E+04	8.90E+03
522	2-Aminoisobutyric acid	Amino acids	5.11E+06	4.92E+06	5.09E+06	5.54E+06	5.43E+06	5.36E+06	3.30E+06	3.12E+06	3.30E+06	6.63E+06	6.69E+06	6.86E+06
523	N-Acetyl-L-glycine	Amino acids	1.75E+04	1.67E+04	1.67E+04	1.91E+04	1.73E+04	1.26E+04	1.57E+04	1.35E+04	1.53E+04	2.11E+04	1.91E+04	1.89E+04
524	L-Proline	Amino acids	3.86E+05	3.78E+05	3.90E+05	3.34E+05	3.62E+05	3.64E+05	3.78E+05	3.61E+05	3.76E+05	4.08E+05	4.40E+05	4.28E+05
525	L-Homocystine	Amino acids	4.72E+04	4.73E+04	4.75E+04	5.14E+04	4.22E+04	5.34E+04	3.67E+04	4.05E+04	3.97E+04	5.95E+04	5.49E+04	5.28E+04
526	L-Threonine	Amino acids	1.21E+06	1.19E+06	1.20E+06	1.48E+06	1.44E+06	1.39E+06	6.48E+05	6.28E+05	6.20E+05	1.77E+06	1.58E+06	1.53E+06
527	L-Methionine Sulfoxide	Amino acids	5.09E+06	4.96E+06	5.08E+06	5.19E+06	5.24E+06	5.10E+06	4.41E+06	4.24E+06	4.13E+06	5.54E+06	5.83E+06	6.12E+06
528	L-α-Glutamyl-L-Glutamic Acid	Amino acids	2.97E+04	2.88E+04	3.01E+04	3.73E+04	3.76E+04	4.07E+04	1.07E+04	1.13E+04	1.17E+04	3.88E+04	4.28E+04	4.71E+04
529	L-Glutaminyl-L-valyl-L-valyl-L-cysteine	Amino acids	1.66E+04	1.63E+04	1.67E+04	1.67E+04	1.93E+04	1.79E+04	1.49E+04	1.06E+04	1.20E+04	1.96E+04	1.93E+04	2.02E+04
530	L-Homocitrulline	Amino acids	1.15E+04	1.13E+04	1.06E+04	1.59E+04	8.92E+03	8.32E+03	9.90E+03	7.77E+03	1.47E+04	1.43E+04	1.05E+04	1.04E+04
531	3-Hydroxy-3-methylpentane-1,5-dioic acid	Amino acids	6.29E+05	6.35E+05	6.33E+05	4.18E+05	3.92E+05	4.53E+05	1.01E+06	9.90E+05	9.01E+05	4.72E+05	4.38E+05	4.07E+05
532	2,3-Dimethylsuccinic acid	Amino acids	3.76E+04	3.72E+04	3.76E+04	3.77E+04	3.42E+04	3.80E+04	3.70E+04	4.25E+04	3.45E+04	3.27E+04	4.09E+04	4.09E+04
533	Phenylacetylglycine	Amino acids	3.21E+04	3.21E+04	3.21E+04	9.00E+00	9.00E+00	9.00E+00	7.93E+04	9.60E+04	8.15E+04	9.00E+00	9.00E+00	9.00E+00
534	N-Acetyl-L-tyrosine	Amino acids	2.22E+04	2.19E+04	2.19E+04	1.19E+04	1.11E+04	9.53E+03	4.30E+04	4.44E+04	3.47E+04	9.82E+03	1.04E+04	1.22E+04
535	D-alpha-Aminobutyric acid	Amino acids	1.40E+07	1.37E+07	1.28E+07	1.09E+07	1.31E+07	1.61E+07	1.21E+07	9.35E+06	1.25E+07	1.13E+07	1.37E+07	1.39E+07
536	2-Chloro-DL-Phenylalanine	Amino acids	1.31E+08	1.43E+08	1.41E+08	1.46E+08	1.28E+08	1.54E+08	1.47E+08	1.42E+08	1.41E+08	1.49E+08	1.36E+08	1.28E+08
537	L-Valyl-L-Phenylalanine	Amino acids	2.13E+05	2.15E+05	2.12E+05	2.63E+05	2.58E+05	2.59E+05	1.56E+05	1.52E+05	1.43E+05	2.47E+05	2.41E+05	2.45E+05
538	3-Nitro-L-tyrosine	Amino acids	2.41E+03	1.02E+04	3.88E+03	9.91E+03	2.58E+03	4.90E+03	1.09E+03	2.71E+03	4.07E+03	7.62E+03	5.21E+03	2.86E+03
539	4-Hydroxyphenylacetylglutamic acid	Amino acids	3.99E+06	3.79E+06	5.19E+06	5.34E+06	4.69E+06	6.56E+06	3.30E+06	5.08E+06	7.26E+06	4.02E+06	3.84E+06	6.79E+06
540	NG,NG-Dimethylarginine dihydrochloride	Amino acids	9.34E+04	8.70E+04	1.15E+05	4.94E+04	1.24E+05	1.40E+05	1.16E+05	1.31E+05	1.35E+05	5.24E+04	1.08E+05	1.16E+05
541	5-Hydroxylysine	Amino acids	1.31E+03	4.11E+03	2.84E+03	3.70E+03	2.31E+03	3.47E+03	2.69E+03	3.15E+03	2.96E+03	1.32E+03	2.60E+03	4.20E+03
542	O-Succinyl-L-homoserine	Amino acids	6.86E+02	3.94E+03	1.70E+03	1.92E+03	1.19E+03	2.07E+03	1.31E+03	3.68E+03	1.04E+03	1.44E+03	1.67E+03	1.22E+03

543	L-Leucyl-L-Leucine	Amino acids	3.40E+05	3.44E+05	3.35E+05	4.26E+05	4.32E+05	3.82E+05	3.03E+05	2.93E+05	2.53E+05	3.28E+05	3.37E+05	3.50E+05
544	N-Acetyl-L-leucine	Amino acids	1.41E+05	1.41E+05	1.41E+05	3.51E+04	3.76E+04	3.18E+04	3.30E+05	3.22E+05	3.20E+05	2.58E+04	2.79E+04	3.17E+04
545	1-Methy-L-histidine	Amino acids	4.34E+03	1.33E+04	7.82E+03	2.74E+03	8.83E+03	1.05E+04	1.20E+04	8.18E+03	5.15E+03	6.35E+03	6.31E+03	5.28E+03
546	DL-Alanine	Amino acids	7.67E+04	1.05E+05	9.56E+04	9.36E+04	1.34E+05	1.26E+05	6.31E+04	7.05E+04	8.66E+04	8.90E+04	1.00E+05	9.58E+04
547	3,4-Dihydroxy-L-phenylalanine	Amino acids	2.52E+04	2.70E+04	2.73E+04	3.01E+04	3.77E+04	4.67E+04	1.79E+04	1.21E+04	1.28E+04	2.72E+04	3.14E+04	3.28E+04
548	(2S,5S)-trans-Carboxymethylproline	Amino acids	3.16E+03	5.34E+03	5.77E+03	8.96E+03	1.08E+04	2.54E+03	6.35E+03	3.37E+03	6.53E+03	3.84E+03	6.93E+03	5.84E+03
549	p-Hydroxyphenylacetylglycine	Amino acids	3.33E+03	1.86E+03	2.16E+03	1.88E+03	1.53E+03	5.81E+03	8.26E+02	5.99E+02	1.73E+03	1.46E+03	4.17E+03	1.18E+03
550	N-Acetyl-L-threonine	Amino acids	6.14E+04	6.39E+04	6.14E+04	7.73E+04	8.72E+04	7.76E+04	4.99E+04	5.04E+04	5.01E+04	6.45E+04	5.40E+04	5.75E+04
551	N-Acetyl-L-glutamic acid	Amino acids	2.38E+06	2.38E+06	2.38E+06	1.12E+05	8.92E+04	1.10E+05	6.37E+06	6.53E+06	5.70E+06	7.17E+04	7.49E+04	7.57E+04
552	4-Acetamidobutyric acid	Amino acids	1.95E+06	2.02E+06	1.90E+06	2.49E+06	2.24E+06	2.11E+06	2.21E+06	2.13E+06	1.82E+06	1.80E+06	1.35E+06	1.53E+06
553	O-Acetylserine	Amino acids	3.95E+05	4.24E+05	3.99E+05	6.39E+05	6.53E+05	6.72E+05	1.80E+05	1.94E+05	2.06E+05	4.37E+05	4.12E+05	4.36E+05
554	L-Glutamic acid-O-glycoside	Amino acids	1.08E+05	1.14E+05	1.07E+05	1.79E+05	1.91E+05	1.72E+05	4.69E+04	4.89E+04	5.50E+04	1.07E+05	1.13E+05	1.23E+05
555	L-Serine	Amino acids	1.01E+06	1.08E+06	1.01E+06	1.38E+06	1.48E+06	1.37E+06	8.99E+05	8.50E+05	9.23E+05	9.08E+05	8.05E+05	8.36E+05
556	Acetylleucine Monoethanolamine	Amino acids	2.87E+06	2.87E+06	2.87E+06	3.64E+04	3.75E+04	3.11E+04	7.70E+06	8.26E+06	6.86E+06	2.27E+04	1.39E+04	2.16E+04
557	L-Glutamine-O-glycoside	Amino acids	9.53E+04	1.02E+05	9.39E+04	1.60E+05	1.72E+05	1.49E+05	6.22E+04	4.49E+04	6.66E+04	9.70E+04	6.11E+04	9.92E+04
558	2,6-Diaminooimelic acid	Amino acids	6.51E+05	7.24E+05	6.38E+05	1.29E+06	1.08E+06	1.19E+06	3.48E+05	3.25E+05	3.11E+05	6.52E+05	5.85E+05	6.12E+05
559	N-Acetyl-L-Arginine	Amino acids	1.78E+04	1.89E+04	1.77E+04	2.01E+04	1.82E+04	1.91E+04	2.90E+04	2.21E+04	2.41E+04	1.21E+04	6.73E+03	1.06E+04
560	L-Prolyl-L-Phenylalanine	Amino acids	1.13E+05	1.14E+05	1.12E+05	3.18E+04	3.60E+04	2.99E+04	2.72E+05	2.80E+05	2.32E+05	1.82E+04	1.04E+04	1.98E+04
561	L-Glutamic acid	Amino acids	1.12E+07	1.24E+07	1.12E+07	1.79E+07	1.85E+07	1.79E+07	9.64E+06	8.85E+06	8.90E+06	9.25E+06	8.25E+06	8.69E+06
562	N-formylmethionine	Amino acids	3.14E+03	6.07E+03	7.51E+03	6.00E+03	1.20E+04	1.15E+04	1.16E+04	5.12E+03	7.56E+03	5.54E+03	2.44E+03	4.33E+03
563	L-Theanine	Amino acids	9.33E+03	1.32E+04	9.50E+03	5.46E+03	1.72E+04	1.81E+04	7.06E+03	1.66E+04	1.55E+03	5.35E+03	7.00E+03	3.20E+03
564	Trans-4-Hydroxy-L-proline	Amino acids	2.39E+05	2.71E+05	2.27E+05	5.33E+05	5.27E+05	4.35E+05	1.18E+05	1.01E+05	8.74E+04	1.89E+05	1.75E+05	1.85E+05
565	DL-Tyrosine	Amino acids	1.59E+03	5.81E+04	3.85E+04	8.12E+04	3.70E+04	2.80E+03	1.49E+05	4.13E+03	7.84E+04	8.49E+03	3.35E+03	2.55E+04
566	L-Cystine	Amino acids	1.29E+04	1.70E+04	1.40E+04	3.11E+04	3.24E+04	4.03E+04	7.66E+03	8.74E+03	7.01E+03	6.47E+03	2.53E+03	7.09E+03
567	L-Cysteine	Amino acids	3.67E+03	1.00E+01	1.18E+03	1.26E+03	6.30E+03	3.07E+03	1.00E+01	1.00E+01	1.00E+01	1.00E+01	1.00E+01	1.00E+01
568	Cis-4,7,10,13,16,19-Docosahexaenoic Acid	Free fatty acids and glycerides	2.88E+03	2.50E+03	2.88E+03	9.00E+00	9.00E+00	9.00E+00	1.77E+03	1.89E+03	1.34E+03	1.29E+04	2.05E+03	3.08E+03

569	9,10-Epoxyoctadecanoic Acid	Free fatty acids and glycerides	5.55E+03	5.08E+03	5.55E+03	9.00E+00	9.00E+00	9.00E+00	1.08E+04	1.23E+04	1.10E+04	3.08E+03	3.52E+03	3.71E+03
570	9,10,13-Trihydroxy-11-Octadecenoic Acid	Free fatty acids and glycerides	6.12E+06	5.06E+06	6.15E+06	2.14E+06	2.04E+06	2.33E+06	4.04E+06	4.05E+06	4.30E+06	1.06E+07	1.10E+07	1.08E+07
571	9-Hydroxy-12-oxo-15(Z)-octadecenoic acid	Free fatty acids and glycerides	7.72E+05	7.13E+05	7.72E+05	1.92E+05	2.01E+05	1.88E+05	1.26E+06	1.31E+06	1.24E+06	6.71E+05	6.49E+05	6.58E+05
572	9,12,13-Trihydroxy-10,15-octadecadienoic acid	Free fatty acids and glycerides	3.05E+05	2.71E+05	3.06E+05	1.16E+05	1.20E+05	1.27E+05	3.32E+05	3.49E+05	3.39E+05	3.86E+05	3.97E+05	4.01E+05
573	9-Hydroxy-13-oxo-10-octadecenoic Acid	Free fatty acids and glycerides	2.72E+05	2.48E+05	2.74E+05	9.26E+04	9.44E+04	1.08E+05	3.47E+05	3.55E+05	3.71E+05	3.07E+05	3.12E+05	2.99E+05
574	12,13-Epoxy-9-Octadecenoic Acid	Free fatty acids and glycerides	4.15E+05	3.97E+05	4.16E+05	9.65E+04	9.09E+04	9.92E+04	7.55E+05	7.64E+05	8.46E+05	2.61E+05	2.66E+05	2.43E+05
575	9-Octadecenamide (Oleamide)	Free fatty acids and glycerides	1.79E+05	1.65E+05	1.78E+05	9.36E+04	1.05E+05	8.36E+04	2.08E+05	1.63E+05	1.61E+05	2.29E+05	2.80E+05	1.96E+05
576	13-KODE; (9Z,11E)-13-Oxooctadeca-9,11-dienoic acid	Free fatty acids and glycerides	2.83E+05	2.69E+05	2.82E+05	8.13E+04	8.40E+04	7.54E+04	5.07E+05	5.36E+05	5.11E+05	1.79E+05	1.81E+05	1.84E+05
577	12,13-DHOME; (9Z)-12,13-Dihydroxyoctadec-9-enoic acid	Free fatty acids and glycerides	1.89E+05	1.74E+05	1.91E+05	1.03E+05	9.60E+04	1.18E+05	1.86E+05	1.96E+05	2.19E+05	2.28E+05	2.47E+05	2.37E+05
578	9-Hydroxy-10,12,15-octadecatrienoic acid	Free fatty acids and glycerides	2.68E+05	2.56E+05	2.68E+05	7.88E+04	7.95E+04	7.29E+04	4.76E+05	5.05E+05	4.91E+05	1.72E+05	1.75E+05	1.71E+05
579	13S-Hydroperoxy-9Z,11E-octadecadienoic acid	Free fatty acids and glycerides	4.16E+06	3.95E+06	4.19E+06	1.52E+06	1.59E+06	1.74E+06	6.37E+06	6.22E+06	6.99E+06	3.60E+06	3.57E+06	3.42E+06
580	9Z,11E,13Z-octadecatrienoic acid (Punicic acid)	Free fatty acids and glycerides	1.27E+07	1.21E+07	1.28E+07	4.61E+06	4.64E+06	4.95E+06	2.08E+07	2.01E+07	2.12E+07	1.04E+07	1.04E+07	9.67E+06
581	9-Oxo-10E,12Z-octadecadienoic acid	Free fatty acids and glycerides	2.19E+05	2.11E+05	2.19E+05	5.38E+04	5.70E+04	5.27E+04	4.21E+05	4.18E+05	4.52E+05	1.17E+05	1.17E+05	1.17E+05
582	9(10)-EpOME;(9R,10S)-(12Z)-9,10-Epoxyoctadecenoic acid	Free fatty acids and glycerides	8.74E+05	8.47E+05	8.73E+05	2.36E+05	2.31E+05	2.26E+05	1.72E+06	1.72E+06	1.78E+06	4.38E+05	4.35E+05	4.43E+05
583	9,12-Octadecadien-6-Ynoic Acid	Free fatty acids and glycerides	4.84E+04	4.59E+04	4.73E+04	2.73E+04	1.66E+04	1.89E+04	6.68E+04	7.70E+04	8.17E+04	4.24E+04	3.65E+04	3.86E+04
584	Eicosadienoic acid	Free fatty acids and glycerides	4.65E+06	4.45E+06	4.67E+06	2.08E+06	2.19E+06	2.23E+06	7.23E+06	7.32E+06	7.06E+06	3.69E+06	3.81E+06	3.79E+06
585	13-Hydroxy-6,9,11-octadecatrienoic acid	Free fatty acids and glycerides	2.98E+04	2.84E+04	2.98E+04	1.97E+04	1.87E+04	1.92E+04	3.53E+04	3.43E+04	3.44E+04	3.27E+04	3.27E+04	3.08E+04
586	Cis-10-Heptadecenoic Acid	Free fatty acids and glycerides	2.70E+06	2.65E+06	2.71E+06	6.53E+05	6.97E+05	7.25E+05	5.38E+06	5.85E+06	5.63E+06	1.15E+06	1.14E+06	1.09E+06
587	Heptadecanoic acid	Free fatty acids and glycerides	9.25E+04	9.11E+04	9.44E+04	2.87E+04	2.64E+04	4.37E+04	1.65E+05	1.84E+05	1.81E+05	4.35E+04	5.78E+04	5.44E+04
588	3-Hydroxyoctadecanoic Acid	Free fatty acids and glycerides	5.07E+05	4.80E+05	4.99E+05	3.82E+05	4.05E+05	3.20E+05	3.93E+05	4.01E+05	7.94E+05	5.92E+05	5.51E+05	5.33E+05
589	1-Eicosanol	Free fatty acids and glycerides	1.95E+05	1.90E+05	1.95E+05	9.79E+04	8.78E+04	1.02E+05	3.02E+05	2.96E+05	3.50E+05	1.42E+05	1.40E+05	1.42E+05
590	13(S)-HODE;13(S)-Hydroxyoctadeca-9Z,11E-dienoic acid	Free fatty acids and glycerides	5.83E+06	5.72E+06	5.84E+06	2.52E+06	2.43E+06	2.65E+06	9.94E+06	1.01E+07	1.06E+07	3.71E+06	3.75E+06	3.52E+06
591	9S-Hydroxy-10E,12Z-octadecadienoic acid	Free fatty acids and glycerides	5.85E+06	5.73E+06	5.86E+06	2.56E+06	2.46E+06	2.65E+06	1.01E+07	1.02E+07	1.05E+07	3.72E+06	3.72E+06	3.59E+06
592	11-Octadecanoic acid (Vaccenic acid)	Free fatty acids and glycerides	2.30E+07	2.25E+07	2.29E+07	1.26E+07	1.22E+07	1.21E+07	3.63E+07	3.65E+07	3.63E+07	1.66E+07	1.69E+07	1.64E+07
593	Arachidonic Acid	Free fatty acids and glycerides	9.98E+03	1.00E+04	1.03E+04	4.46E+03	7.09E+03	7.05E+03	1.02E+04	1.44E+04	1.96E+04	8.71E+03	8.63E+03	6.75E+03
594	1-O-Feruloyl-3-O-caffeoylglycerol	Free fatty acids and glycerides	7.52E+04	7.46E+04	7.59E+04	4.93E+04	6.08E+04	5.50E+04	8.40E+04	9.01E+04	1.26E+05	5.83E+04	7.35E+04	5.96E+04

595	Undecylic Acid	Free fatty acids and glycerides	3.41E+05	3.40E+05	3.50E+05	3.24E+05	3.24E+05	3.91E+05	3.23E+05	2.82E+05	3.39E+05	3.22E+05	4.14E+05	4.04E+05
596	Pentadecanoic Acid	Free fatty acids and glycerides	2.91E+05	2.91E+05	2.95E+05	2.18E+05	2.26E+05	2.51E+05	3.79E+05	4.07E+05	3.67E+05	2.13E+05	2.67E+05	2.54E+05
597	Octadeca-11E,13E,15Z-trienoic acid	Free fatty acids and glycerides	1.01E+07	1.03E+07	1.03E+07	1.01E+07	9.52E+06	1.17E+07	9.43E+06	1.01E+07	9.34E+06	1.01E+07	1.26E+07	1.00E+07
598	1-Linoleoylglycerol	Free fatty acids and glycerides	1.55E+06	1.56E+06	1.56E+06	1.46E+06	1.51E+06	1.49E+06	1.74E+06	1.65E+06	1.73E+06	1.41E+06	1.47E+06	1.45E+06
599	Linoleic acid	Free fatty acids and glycerides	3.13E+07	3.13E+07	3.11E+07	2.66E+07	2.57E+07	2.48E+07	3.98E+07	4.15E+07	4.21E+07	2.49E+07	2.50E+07	2.49E+07
600	Monopalmitin	Free fatty acids and glycerides	6.26E+04	6.37E+04	6.38E+04	4.38E+04	5.31E+04	5.38E+04	8.32E+04	9.46E+04	8.27E+04	4.85E+04	5.00E+04	4.44E+04
601	Palmitoleic Acid	Free fatty acids and glycerides	1.07E+04	1.14E+04	1.22E+04	1.00E+04	1.10E+04	2.15E+04	8.74E+03	8.44E+03	8.92E+03	1.05E+04	1.21E+04	1.61E+04
602	9-Hydroperoxy-10E,12,15Z-octadecatrienoic acid	Free fatty acids and glycerides	1.10E+05	1.11E+05	1.06E+05	8.78E+04	4.74E+04	5.49E+04	1.69E+05	2.08E+05	1.96E+05	7.97E+04	4.66E+04	4.54E+04
603	γ-Linolenic Acid	Free fatty acids and glycerides	1.76E+07	1.80E+07	1.75E+07	1.37E+07	1.31E+07	1.29E+07	2.78E+07	2.81E+07	2.79E+07	1.01E+07	1.02E+07	1.03E+07
604	1-Linoleoylglycerol-2,3-di-O-glucoside	Free fatty acids and glycerides	1.30E+04	1.41E+04	1.39E+04	1.88E+04	1.79E+04	2.55E+04	6.77E+03	4.57E+03	8.49E+03	1.95E+04	1.15E+04	1.67E+04
605	2-Linoleoylglycerol	Free fatty acids and glycerides	1.59E+06	1.63E+06	1.57E+06	1.73E+06	1.66E+06	1.64E+06	1.76E+06	1.81E+06	1.89E+06	1.24E+06	1.31E+06	1.27E+06
606	α-Linolenic Acid	Free fatty acids and glycerides	1.78E+07	1.82E+07	1.78E+07	1.35E+07	1.37E+07	1.30E+07	2.82E+07	2.87E+07	2.80E+07	9.96E+06	1.02E+07	1.04E+07
607	16-Methylheptadecanoic acid	Free fatty acids and glycerides	2.39E+07	2.68E+07	2.67E+07	1.88E+07	2.00E+07	4.14E+07	4.06E+07	3.77E+07	1.55E+07	2.08E+07	1.95E+07	1.80E+07
608	1-α-Linolenoyl-glycerol	Free fatty acids and glycerides	3.10E+06	3.22E+06	3.09E+06	3.64E+06	3.72E+06	3.58E+06	3.25E+06	3.24E+06	3.37E+06	2.55E+06	2.45E+06	2.60E+06
609	Myristic Acid	Free fatty acids and glycerides	1.14E+07	1.18E+07	1.14E+07	1.17E+07	1.21E+07	1.15E+07	1.35E+07	1.35E+07	1.62E+07	7.73E+06	8.66E+06	8.05E+06
610	7S,8S-DiHODE;<br>(9Z,12Z)-(7S,8S)-Dihydroxyoctadeca-9,12-dienoic acid	Free fatty acids and glycerides	1.37E+06	1.50E+06	1.33E+06	2.78E+06	1.76E+06	2.53E+06	6.54E+05	5.99E+05	8.46E+05	1.37E+06	1.45E+06	1.48E+06
611	2-α-Linolenoyl-glycerol	Free fatty acids and glycerides	2.02E+06	2.11E+06	1.98E+06	2.51E+06	2.44E+06	2.17E+06	2.22E+06	2.31E+06	2.38E+06	1.41E+06	1.43E+06	1.44E+06
612	Eicosenoic acid	Free fatty acids and glycerides	2.33E+06	2.88E+06	2.88E+06	9.37E+05	1.02E+06	5.32E+06	5.25E+06	5.23E+06	1.95E+06	1.72E+06	1.64E+06	9.39E+05
613	1-α-Linolenoyl-glycerol-2,3-di-O-glucoside	Free fatty acids and glycerides	3.49E+03	3.75E+03	3.33E+03	5.99E+03	6.56E+03	4.64E+03	1.91E+03	2.20E+03	1.45E+03	3.96E+03	3.31E+03	2.58E+03
614	1-Oleoyl-Sn-Glycerol	Free fatty acids and glycerides	7.87E+05	8.20E+05	7.75E+05	7.18E+05	6.64E+05	6.27E+05	1.26E+06	1.39E+06	1.22E+06	3.12E+05	3.72E+05	3.59E+05
615	2-Linoleoylglycerol-1,3-di-O-glucoside	Free fatty acids and glycerides	1.28E+05	1.30E+05	1.14E+05	2.05E+05	1.26E+05	9.20E+04	1.37E+05	1.57E+05	1.86E+05	7.03E+04	6.23E+04	8.25E+04
616	2-Linoleoylglycerol-1-O-glucoside	Free fatty acids and glycerides	3.16E+04	3.28E+04	3.02E+04	3.57E+04	2.98E+04	2.53E+04	4.39E+04	4.99E+04	4.79E+04	1.59E+04	1.43E+04	1.51E+04
617	2-α-Linolenoyl-glycerol-1,3-di-O-glucoside	Free fatty acids and glycerides	3.73E+04	3.88E+04	3.70E+04	3.70E+04	3.16E+04	3.45E+04	5.75E+04	6.23E+04	6.08E+04	1.27E+04	1.41E+04	2.25E+04
618	LysoPG 16:0	Free fatty acids and glycerides	3.04E+05	3.47E+05	3.46E+05	2.82E+05	2.82E+05	6.21E+05	6.34E+05	5.54E+05	1.18E+05	1.43E+05	1.41E+05	2.75E+05
619	1-α-Linolenoyl-glycerol-3-O-glucoside	Free fatty acids and glycerides	2.45E+03	3.21E+03	2.80E+03	5.60E+03	5.02E+03	8.45E+03	9.00E+00	9.00E+00	9.00E+00	3.34E+03	3.26E+03	2.34E+03

620	1-Linolenoyl-rac-glycerol-diglucoside	Free fatty acids and glycerides	2.96E+05	3.22E+05	2.91E+05	4.75E+05	4.62E+05	4.35E+05	2.73E+05	2.71E+05	2.55E+05	2.13E+05	1.96E+05	2.23E+05
621	1-Stearidonoyl-Glycerol	Free fatty acids and glycerides	3.36E+05	3.60E+05	3.32E+05	3.65E+05	3.41E+05	3.38E+05	5.23E+05	5.09E+05	5.32E+05	1.33E+05	1.38E+05	1.45E+05
622	2-α-Linolenoyl-glycerol-1-O-glucoside	Free fatty acids and glycerides	1.91E+04	2.10E+04	1.89E+04	2.27E+04	2.27E+04	2.13E+04	2.94E+04	2.65E+04	2.99E+04	8.25E+03	7.01E+03	6.21E+03
623	8,15-Dihydroxy-5,9,11,13-eicosatetraenoic acid	Free fatty acids and glycerides	3.33E+05	4.18E+05	3.24E+05	9.65E+05	9.80E+05	8.87E+05	3.25E+04	4.05E+04	3.48E+04	2.00E+05	2.03E+05	2.12E+05
624	1-Methyladenine	Nucleotides	2.66E+04	2.02E+04	2.69E+04	5.58E+03	9.67E+03	7.50E+03	7.27E+03	4.37E+03	5.86E+03	5.89E+04	6.23E+04	5.91E+04
625	Adenosine 5'-monophosphate	Nucleotides	1.55E+06	1.25E+06	1.55E+06	4.09E+05	4.15E+05	4.06E+05	8.34E+05	9.00E+05	8.72E+05	3.03E+06	3.15E+06	2.76E+06
626	2-Deoxyribose-1-phosphate	Nucleotides	3.60E+06	3.38E+06	3.60E+06	3.90E+05	3.47E+05	3.72E+05	5.75E+06	7.14E+06	8.68E+06	2.12E+06	2.25E+06	2.16E+06
627	N1-(alpha-D-ribosyl)-5,6-dimethyl-benzimidazole	Nucleotides	3.90E+04	6.81E+04	7.83E+04	3.10E+04	2.96E+04	2.75E+04	2.42E+04	1.86E+04	2.76E+04	9.06E+04	2.78E+05	1.31E+05
628	6-Methylmercaptopurine	Nucleotides	8.16E+06	6.33E+06	8.18E+06	3.09E+06	3.16E+06	3.19E+06	2.25E+06	2.10E+06	2.02E+06	1.74E+07	1.74E+07	1.79E+07
629	Isopentenyladenine-7-N-glucoside	Nucleotides	5.48E+04	4.73E+04	5.34E+04	3.20E+04	1.48E+04	2.08E+04	3.65E+04	4.92E+04	4.43E+04	9.73E+04	8.33E+04	8.06E+04
630	Adenosine 5'-diphosphate	Nucleotides	2.02E+04	1.73E+04	2.01E+04	1.19E+04	9.50E+03	1.08E+04	8.41E+03	1.27E+04	1.19E+04	3.49E+04	3.80E+04	3.45E+04
631	5-Hydroxymethyluracil	Nucleotides	1.27E+03	4.32E+03	1.19E+03	7.97E+02	6.68E+02	3.45E+02	1.92E+03	1.00E+01	9.98E+02	3.30E+03	1.94E+03	3.07E+02
632	2'-Deoxycytidine	Nucleotides	2.27E+05	1.95E+05	2.27E+05	1.53E+05	1.69E+05	1.49E+05	4.96E+04	6.30E+04	6.47E+04	4.78E+05	4.37E+05	4.02E+05
633	5-Aminoimidazole ribonucleotide	Nucleotides	7.61E+05	7.16E+05	7.61E+05	1.85E+05	2.00E+05	1.88E+05	1.46E+06	1.30E+06	1.41E+06	4.90E+05	4.92E+05	5.44E+05
634	Uridine 5'-monophosphate	Nucleotides	1.65E+05	1.48E+05	1.66E+05	8.21E+04	1.04E+05	8.92E+04	1.54E+05	1.47E+05	1.45E+05	2.38E+05	2.27E+05	2.25E+05
635	Isoguanine	Nucleotides	2.33E+05	2.02E+05	2.33E+05	1.39E+05	1.56E+05	1.45E+05	1.54E+05	1.51E+05	1.61E+05	3.68E+05	3.42E+05	3.91E+05
636	5-Methylcytosine	Nucleotides	2.18E+04	2.00E+04	2.16E+04	1.67E+04	2.03E+04	1.48E+04	6.87E+03	8.75E+03	6.69E+03	4.78E+04	3.79E+04	2.97E+04
637	Guanine	Nucleotides	5.82E+05	5.28E+05	5.85E+05	3.97E+05	3.87E+05	4.23E+05	4.82E+05	3.97E+05	3.97E+05	8.40E+05	9.01E+05	8.56E+05
638	Nicotinamide adenine dinucleotide phosphate (NADP)	Nucleotides	1.03E+04	1.04E+04	1.06E+04	2.99E+03	4.62E+03	5.35E+03	1.58E+04	1.89E+04	1.74E+04	8.31E+03	9.80E+03	4.81E+03
639	Cytidine 5'-monophosphate (Cytidylic acid)	Nucleotides	5.20E+04	4.88E+04	5.33E+04	3.31E+04	4.42E+04	4.38E+04	4.94E+04	5.38E+04	4.07E+04	5.93E+04	6.61E+04	6.93E+04
640	β-Nicotinamide mononucleotide	Nucleotides	3.01E+05	2.98E+05	3.09E+05	2.41E+05	2.18E+05	3.00E+05	2.57E+05	2.29E+05	2.45E+05	4.72E+05	4.21E+05	3.27E+05
641	Levamisole	Nucleotides	2.68E+04	9.46E+03	2.98E+04	2.49E+04	2.85E+04	1.64E+04	2.12E+04	2.62E+04	4.48E+04	2.22E+04	4.76E+04	3.13E+04
642	7-Methylguanine	Nucleotides	1.06E+04	1.03E+04	1.02E+04	1.01E+04	8.02E+03	7.24E+03	1.11E+04	9.45E+03	1.04E+04	1.02E+04	1.57E+04	9.76E+03
643	2-Deoxyribose-5'-phosphate	Nucleotides	8.32E+05	7.78E+05	8.32E+05	7.75E+05	8.67E+05	7.80E+05	5.86E+05	5.27E+05	5.90E+05	9.65E+05	1.14E+06	1.21E+06
644	Adenosine triphosphate	Nucleotides	4.89E+03	1.67E+04	4.72E+03	1.00E+01	2.25E+03	5.35E+03	5.22E+03	1.30E+04	1.55E+03	7.42E+02	2.33E+03	7.31E+03
645	Thymidine	Nucleotides	5.10E+05	4.85E+05	5.13E+05	6.47E+05	6.91E+05	6.67E+05	8.90E+04	8.45E+04	1.03E+05	7.98E+05	8.04E+05	8.67E+05

646	1-methylguanidine	Nucleotides	1.25E+04	1.25E+04	1.26E+04	1.48E+04	1.36E+04	1.50E+04	4.87E+03	7.60E+03	6.29E+03	1.91E+04	1.88E+04	1.54E+04
647	Nicotinic acid adenine dinucleotide	Nucleotides	2.56E+04	2.45E+04	2.49E+04	2.31E+04	2.05E+04	1.77E+04	3.02E+04	3.10E+04	2.97E+04	2.08E+04	2.28E+04	2.66E+04
648	5'-Deoxy-5'-(methylthio)adenosine	Nucleotides	1.25E+06	1.23E+06	1.25E+06	1.44E+06	1.25E+06	1.43E+06	9.31E+05	8.44E+05	8.39E+05	1.59E+06	1.53E+06	1.57E+06
649	Uridine	Nucleotides	7.70E+05	7.53E+05	7.79E+05	9.64E+05	1.05E+06	1.03E+06	2.42E+05	2.40E+05	2.58E+05	1.35E+06	8.88E+05	1.17E+06
650	Nicotinate D-ribonucleoside	Nucleotides	1.32E+06	1.31E+06	1.32E+06	3.23E+05	2.71E+05	3.23E+05	3.00E+06	3.10E+06	2.84E+06	3.62E+05	2.85E+05	3.80E+05
651	5-Methyluridine	Nucleotides	5.31E+03	5.39E+03	5.54E+03	4.00E+03	6.64E+03	5.89E+03	2.88E+03	5.95E+03	4.89E+03	7.02E+03	5.83E+03	5.23E+03
652	Xanthosine	Nucleotides	1.59E+05	1.58E+05	1.60E+05	1.05E+05	9.15E+04	1.09E+05	2.46E+05	2.62E+05	2.34E+05	1.10E+05	1.10E+05	1.16E+05
653	N7-Methylguanosine	Nucleotides	1.04E+04	1.07E+04	1.14E+04	6.51E+03	7.65E+03	1.50E+04	1.49E+04	1.50E+04	7.34E+03	8.73E+03	1.06E+04	1.24E+04
654	β-Pseudouridine	Nucleotides	5.71E+04	5.75E+04	5.59E+04	7.70E+04	7.23E+04	6.81E+04	2.64E+04	2.27E+04	2.43E+04	8.09E+04	8.80E+04	6.49E+04
655	Purine	Nucleotides	1.34E+03	4.59E+03	2.73E+03	6.98E+02	6.37E+03	1.90E+03	5.21E+02	8.51E+02	2.83E+03	8.70E+02	2.52E+03	5.96E+03
656	Adenosine 3',5'-diphosphate	Nucleotides	7.63E+03	7.62E+03	5.17E+03	4.30E+03	5.69E+03	7.07E+03	4.36E+03	2.23E+03	5.43E+03	6.70E+03	4.15E+03	5.69E+03
657	Cyclic 3',5'-Adenylic acid	Nucleotides	2.30E+05	2.26E+05	2.26E+05	2.64E+05	2.48E+05	2.28E+05	2.16E+05	2.21E+05	1.88E+05	2.16E+05	2.24E+05	2.65E+05
658	Uridine 5'-diphospho-D-glucose	Nucleotides	2.11E+05	2.10E+05	2.03E+05	2.91E+05	2.62E+05	2.25E+05	1.44E+05	1.18E+05	1.55E+05	2.61E+05	2.25E+05	2.35E+05
659	2'-Deoxyguanosine	Nucleotides	1.16E+06	1.18E+06	1.16E+06	1.80E+06	1.86E+06	1.79E+06	2.02E+05	2.18E+05	2.14E+05	1.66E+06	1.70E+06	1.66E+06
660	Uracil	Nucleotides	3.37E+05	3.44E+05	3.40E+05	3.21E+05	3.83E+05	3.48E+05	3.78E+05	3.43E+05	3.43E+05	3.28E+05	3.04E+05	2.97E+05
661	9-(Arabinosyl)hypoxanthine	Nucleotides	1.13E+05	1.15E+05	1.12E+05	9.00E+04	9.38E+04	8.92E+04	1.67E+05	1.77E+05	1.41E+05	7.76E+04	8.05E+04	7.31E+04
662	Allopurinol	Nucleotides	4.24E+04	4.30E+04	4.15E+04	2.98E+04	2.58E+04	2.25E+04	7.90E+04	6.95E+04	7.31E+04	2.11E+04	2.33E+04	1.73E+04
663	2'-Deoxyadenosine-5'-monophosphate	Nucleotides	1.45E+05	1.56E+05	1.48E+05	2.09E+05	2.08E+05	2.29E+05	7.51E+04	8.32E+04	8.09E+04	1.81E+05	1.84E+05	1.43E+05
664	2'-Deoxyadenosine	Nucleotides	1.78E+06	1.82E+06	1.77E+06	2.84E+06	3.12E+06	2.75E+06	4.00E+05	4.65E+05	5.59E+05	2.04E+06	2.35E+06	2.46E+06
665	2-(Dimethylamino)guanosine	Nucleotides	4.77E+05	4.91E+05	4.79E+05	4.56E+05	4.93E+05	4.72E+05	5.68E+05	6.05E+05	5.98E+05	3.87E+05	3.53E+05	3.61E+05
666	Succinyladenosine	Nucleotides	1.86E+06	1.90E+06	1.85E+06	1.33E+06	1.30E+06	1.32E+06	3.55E+06	2.98E+06	2.82E+06	9.78E+05	9.17E+05	9.68E+05
667	Cordycepin	Nucleotides	3.18E+03	8.21E+03	4.92E+03	1.12E+04	5.85E+03	6.49E+03	3.37E+03	5.66E+03	1.08E+03	2.77E+03	6.94E+03	7.21E+03
668	2'-Deoxyuridine	Nucleotides	4.82E+03	5.42E+03	5.12E+03	7.93E+03	1.04E+04	1.04E+04	9.00E+00	9.00E+00	9.00E+00	6.36E+03	8.24E+03	5.60E+03
669	Guanosine 5'-monophosphate	Nucleotides	9.76E+04	1.01E+05	9.86E+04	7.88E+04	7.64E+04	8.69E+04	1.77E+05	1.62E+05	1.26E+05	4.91E+04	5.25E+04	5.98E+04
670	2'-Deoxyinosine-5'-monophosphate	Nucleotides	6.15E+06	6.46E+06	6.11E+06	7.06E+06	6.74E+06	6.73E+06	8.02E+06	7.19E+06	7.13E+06	4.40E+06	4.40E+06	4.25E+06
671	Hypoxanthine	Nucleotides	4.35E+04	4.69E+04	4.52E+04	3.40E+04	2.21E+04	4.76E+04	6.56E+04	8.47E+04	7.88E+04	1.60E+04	2.66E+04	2.04E+04

672	Guanosine 3',5'-cyclic monophosphate	Nucleotides	3.22E+05	3.44E+05	3.26E+05	3.94E+05	4.37E+05	4.25E+05	3.40E+05	3.43E+05	3.15E+05	2.55E+05	2.48E+05	2.44E+05
673	Cytosine	Nucleotides	5.49E+05	6.03E+05	5.58E+05	9.32E+05	9.64E+05	1.00E+06	2.65E+05	2.45E+05	2.66E+05	5.83E+05	5.69E+05	5.67E+05
674	Xanthine	Nucleotides	1.44E+05	1.54E+05	1.50E+05	1.08E+05	1.34E+05	1.55E+05	2.33E+05	2.86E+05	1.99E+05	6.77E+04	4.95E+04	7.33E+04
675	Cytidine	Nucleotides	3.02E+06	3.46E+06	3.04E+06	5.83E+06	5.72E+06	6.00E+06	1.79E+06	1.63E+06	1.72E+06	2.60E+06	2.40E+06	2.43E+06
676	Adenine	Nucleotides	5.15E+06	5.68E+06	5.18E+06	6.93E+06	6.99E+06	7.23E+06	6.52E+06	5.67E+06	6.43E+06	2.73E+06	2.94E+06	2.95E+06
677	Adenosine	Nucleotides	1.14E+06	1.34E+06	1.13E+06	2.50E+06	2.63E+06	2.40E+06	5.05E+05	4.94E+05	4.89E+05	8.25E+05	8.54E+05	8.20E+05
678	Guanosine	Nucleotides	3.76E+06	4.53E+06	3.70E+06	9.20E+06	8.64E+06	8.66E+06	1.59E+06	1.56E+06	1.48E+06	2.59E+06	2.50E+06	2.54E+06
679	AICAR	Nucleotides	5.39E+05	5.68E+05	9.35E+05	9.85E+05	4.09E+06	1.70E+05	4.32E+05	7.94E+05	5.11E+05	5.06E+05	4.25E+05	5.53E+05
680	2-Aminopurine	Nucleotides	4.69E+04	5.99E+04	4.91E+04	1.26E+05	1.38E+05	1.44E+05	9.00E+00	9.00E+00	9.00E+00	3.31E+04	3.84E+04	3.93E+04
681	N6-Isopentenyladenine	Nucleotides	7.37E+04	7.51E+04	7.41E+04	9.83E+03	1.20E+04	1.36E+04	1.95E+05	1.94E+05	1.71E+05	2.28E+03	2.90E+03	2.32E+03
682	Adenosine 3'-monophosphate	Nucleotides	1.54E+04	6.13E+03	1.09E+04	5.43E+04	3.23E+04	1.79E+04	4.64E+03	4.93E+03	8.09E+03	8.63E+03	3.71E+03	7.01E+03
683	Adenosine 2',3'-cyclic phosphate	Nucleotides	6.44E+03	2.07E+04	4.77E+03	3.08E+05	7.24E+03	1.47E+04	1.47E+03	3.62E+03	2.23E+03	8.44E+02	2.94E+03	5.17E+03
684	Harpagoside	Others	8.37E+03	1.00E+01	4.00E+03	1.00E+01	1.00E+01	1.00E+01	1.72E+03	1.33E+03	1.32E+03	1.67E+04	1.10E+04	1.00E+01
685	Artesunate	Others	1.00E+01	8.96E+04	1.07E+05	1.00E+01	1.00E+01	1.56E+03	2.97E+05	1.00E+01	3.60E+05	1.94E+05	1.00E+01	1.00E+01
686	Simvastatin	Others	1.09E+03	1.15E+04	5.77E+03	1.00E+01	2.22E+03	1.00E+01	9.13E+03	6.47E+03	1.00E+01	1.17E+04	3.11E+03	1.35E+04
687	Deoxyelephantopin	Others	4.19E+04	6.34E+04	5.37E+04	5.36E+03	4.72E+03	6.13E+03	8.16E+04	1.01E+05	1.05E+05	3.58E+04	4.59E+04	4.96E+04
688	Epifriedelanol	Others	1.66E+04	1.52E+04	3.17E+04	1.42E+04	1.50E+04	3.45E+03	1.33E+04	1.61E+04	1.72E+04	1.19E+04	4.15E+03	1.72E+05
689	Sterebin A	Others	3.38E+04	5.28E+04	4.18E+04	4.23E+03	1.76E+04	2.32E+04	2.05E+04	1.47E+04	4.36E+03	5.01E+04	1.02E+05	1.02E+05
690	Diosbulbin B	Others	5.21E+04	5.08E+04	5.37E+04	3.97E+03	3.45E+03	1.63E+04	8.76E+04	1.17E+05	1.14E+05	4.10E+04	2.31E+04	2.71E+04
691	Eucommioside	Others	2.30E+04	2.29E+04	2.27E+04	1.27E+04	9.00E+00	1.02E+04	4.22E+04	3.75E+04	3.96E+04	2.02E+04	2.07E+04	1.09E+04
692	Xanthatin	Others	4.49E+03	9.78E+03	5.16E+03	5.04E+03	3.24E+03	3.67E+03	1.06E+03	4.93E+03	1.66E+03	9.07E+03	9.48E+03	8.14E+03
693	Curcolone	Others	4.49E+03	6.99E+03	5.57E+03	5.04E+03	3.24E+03	3.67E+03	4.37E+03	4.93E+03	1.66E+03	9.07E+03	9.48E+03	8.14E+03
694	Geranylacetate	Others	1.41E+04	2.66E+04	1.01E+04	4.26E+03	8.68E+03	5.08E+03	1.21E+04	6.33E+03	8.14E+03	1.01E+04	7.61E+03	2.25E+04
695	trans-Caryophyllene	Others	1.40E+05	1.43E+05	1.44E+05	9.86E+04	8.63E+04	6.35E+04	1.74E+05	1.69E+05	1.08E+05	1.15E+05	2.04E+05	2.35E+05
696	2-(2-Hydroxy-2-propyl)-5-methyl-5-vinyltetrahydrofuran	Others	1.15E+05	6.98E+04	9.03E+04	5.47E+04	6.59E+04	4.44E+04	9.73E+04	9.96E+04	8.31E+04	8.66E+04	1.31E+05	1.15E+05
697	Abscisic acid	Others	3.59E+03	3.46E+03	3.11E+03	1.97E+03	5.18E+02	3.17E+03	2.85E+03	4.56E+03	2.78E+03	1.74E+03	5.56E+03	3.69E+03

698	Taraxerol	Others	9.89E+03	4.98E+03	6.13E+03	4.07E+03	5.98E+03	4.07E+03	6.06E+03	1.77E+03	3.72E+03	8.83E+03	7.03E+03	1.16E+04
699	Taraxasterone	Others	4.63E+03	2.12E+03	4.87E+03	1.00E+01	3.03E+03	5.76E+03	6.46E+03	2.41E+03	4.82E+03	6.65E+03	3.81E+03	6.06E+03
700	Morroniside	Others	4.90E+03	4.81E+03	5.03E+03	2.67E+03	4.39E+03	4.71E+03	1.06E+03	4.13E+03	5.09E+03	4.26E+03	8.12E+03	8.51E+03
701	Costunolide	Others	2.62E+04	3.50E+04	4.62E+04	1.68E+04	3.56E+04	2.83E+04	5.62E+04	5.52E+04	5.59E+04	3.49E+04	2.69E+04	7.67E+04
702	Oleuroside	Others	8.91E+03	5.35E+03	3.63E+03	5.73E+02	3.97E+03	2.22E+03	4.06E+03	4.05E+03	3.94E+03	2.86E+03	2.33E+03	5.57E+03
703	Germanicol	Others	8.12E+03	6.19E+03	8.35E+03	4.07E+03	8.67E+03	7.15E+03	3.94E+03	1.20E+04	3.99E+03	1.21E+04	1.01E+04	8.95E+03
704	Jangomolide	Others	5.87E+03	3.20E+03	4.44E+03	3.69E+03	4.62E+03	2.84E+03	7.13E+03	1.00E+01	3.85E+03	6.82E+03	3.55E+03	6.72E+03
705	Agnuside	Others	8.00E+03	3.07E+03	8.00E+03	1.07E+04	4.75E+03	6.02E+03	4.94E+03	5.86E+03	1.02E+04	1.64E+04	4.55E+03	1.13E+04
706	Citronellyl acetate	Others	6.38E+03	1.24E+04	1.14E+04	7.05E+03	9.99E+03	1.08E+04	8.04E+03	1.07E+04	9.99E+03	9.43E+03	7.85E+03	2.43E+04
707	Catalpalactone	Others	2.78E+04	1.86E+04	1.41E+04	1.71E+04	9.83E+03	9.61E+03	7.13E+03	2.10E+04	1.08E+04	2.05E+04	2.20E+04	1.17E+04
708	Eurycomalactone	Others	2.09E+04	2.50E+04	2.57E+04	1.83E+04	1.30E+04	2.48E+04	1.85E+04	2.76E+04	4.11E+04	1.98E+04	2.94E+04	3.11E+04
709	(-)-Borneol	Others	2.29E+03	2.91E+03	3.43E+03	6.05E+03	3.15E+03	2.33E+03	9.63E+02	2.52E+03	2.10E+03	4.03E+03	6.19E+03	6.16E+03
710	Retinoic acid	Others	7.12E+03	1.39E+03	3.78E+03	4.66E+03	2.36E+03	3.31E+03	3.60E+03	2.09E+03	4.27E+03	6.09E+03	5.92E+03	2.60E+03
711	Alpha-Santonin	Others	7.19E+03	1.16E+04	7.90E+03	9.69E+03	7.67E+03	6.08E+03	6.09E+03	4.15E+03	6.30E+03	1.13E+04	1.59E+04	5.74E+03
712	Swertiamarin	Others	1.23E+04	3.14E+03	7.01E+03	3.36E+03	1.20E+04	3.16E+03	4.38E+03	1.72E+03	9.14E+03	6.62E+03	9.18E+03	9.82E+03
713	(R)-Menthofuran	Others	1.24E+04	2.43E+04	1.85E+04	1.44E+04	2.25E+04	1.55E+04	1.19E+04	1.28E+04	1.39E+04	2.29E+04	2.27E+04	2.60E+04
714	Betulalbuside A	Others	3.29E+04	2.54E+04	2.82E+04	2.22E+04	2.83E+04	2.79E+04	2.15E+04	2.73E+04	2.31E+04	3.35E+04	2.71E+04	3.71E+04
715	Artemisinin	Others	2.77E+05	3.02E+05	3.29E+05	1.95E+05	3.66E+05	2.91E+05	2.95E+05	3.87E+05	2.44E+05	4.14E+05	2.66E+05	3.67E+05
716	Caryophyllene oxide	Others	2.29E+05	1.89E+05	1.76E+05	1.41E+05	1.23E+05	1.64E+05	1.83E+05	2.25E+05	2.03E+05	2.43E+05	1.47E+05	1.21E+05
717	(1R)-(-)-Menthyl acetate	Others	1.83E+03	6.39E+03	1.69E+03	1.67E+03	1.17E+03	1.15E+03	2.88E+03	5.56E+02	3.03E+03	8.33E+02	3.34E+03	5.62E+02
718	Epitulipinolide diepoxide	Others	6.90E+03	2.28E+03	3.33E+03	4.57E+03	2.80E+03	1.47E+03	4.51E+03	5.09E+03	2.28E+03	4.47E+03	4.56E+03	1.44E+03
719	Ailanthone	Others	1.15E+03	3.51E+03	2.75E+03	3.16E+03	1.36E+03	5.44E+03	1.16E+03	1.44E+03	8.99E+02	1.61E+03	6.98E+03	3.07E+03
720	27,28-Dicarboxyl ursolic acid	Others	1.11E+04	1.12E+04	1.11E+04	1.22E+04	1.06E+04	1.24E+04	8.17E+03	7.03E+03	9.32E+03	1.48E+04	1.50E+04	1.15E+04
721	Tripdiolide	Others	1.07E+04	1.25E+04	1.18E+04	1.87E+04	1.02E+04	1.05E+04	4.58E+03	2.41E+03	2.12E+04	9.45E+03	2.67E+04	9.78E+03
722	(+)-Glaucarubinone	Others	1.85E+04	2.26E+03	1.42E+04	1.56E+04	1.06E+04	1.64E+04	7.41E+03	1.74E+04	1.29E+04	1.80E+04	2.35E+04	7.56E+03
723	Bisabolol oxide A	Others	1.07E+04	1.65E+04	1.50E+04	1.54E+04	1.62E+04	1.10E+04	1.44E+04	1.17E+04	1.72E+04	1.07E+04	1.67E+04	2.16E+04

724	Curcumol	Others	5.91E+03	1.02E+04	8.85E+03	4.16E+03	1.19E+04	6.58E+03	5.25E+03	1.31E+04	8.06E+03	5.35E+03	5.12E+03	1.55E+04
725	Alpha-Terpineol	Others	3.14E+03	2.91E+03	3.07E+03	6.05E+03	3.15E+03	2.33E+03	1.31E+03	2.52E+03	2.10E+03	8.21E+02	6.19E+03	6.16E+03
726	Geniposide	Others	1.25E+04	3.89E+04	1.35E+04	4.79E+03	2.09E+04	1.17E+04	6.65E+03	1.80E+04	8.23E+03	1.28E+04	9.11E+03	2.04E+04
727	2,6-Dimethyl-7-octene-2,3,6-triol	Others	3.77E+03	2.93E+03	2.86E+03	1.74E+03	4.16E+03	2.34E+03	2.14E+03	3.80E+03	1.31E+03	1.87E+03	3.17E+03	4.05E+03
728	Mevastatin	Others	1.93E+04	9.60E+03	1.20E+04	1.95E+04	1.14E+04	1.03E+04	1.17E+04	1.01E+04	1.12E+04	9.72E+03	5.64E+03	2.62E+04
729	Miltirone	Others	1.71E+06	1.90E+06	2.09E+06	1.59E+06	1.82E+06	2.67E+06	1.78E+06	2.17E+06	2.17E+06	2.28E+06	1.43E+06	2.38E+06
730	Catalposide	Others	1.19E+04	1.19E+04	1.19E+04	9.00E+00	9.00E+00	9.00E+00	3.53E+04	3.39E+04	2.56E+04	9.00E+00	9.00E+00	9.00E+00
731	6,7-Dehydroferruginol	Others	1.27E+04	1.42E+04	1.72E+04	2.70E+04	2.11E+04	1.66E+04	1.58E+04	1.70E+04	1.15E+04	1.52E+04	2.06E+04	1.99E+04
732	Lathyrol	Others	9.87E+03	6.33E+03	6.32E+03	4.84E+03	9.41E+03	4.53E+03	4.60E+03	6.05E+03	9.96E+03	7.89E+03	3.99E+03	4.17E+03
733	Anisatin	Others	1.01E+04	6.68E+03	8.59E+03	8.00E+03	1.08E+04	1.57E+04	2.67E+03	1.60E+03	8.43E+03	3.26E+03	6.25E+03	2.00E+04
734	Ursolic acid	Others	7.85E+03	8.07E+03	8.07E+03	3.06E+03	3.06E+03	4.85E+03	1.77E+04	1.39E+04	1.57E+04	3.15E+03	3.09E+03	3.08E+03
735	alpha-Cyperone	Others	5.38E+04	4.41E+04	5.11E+04	7.34E+04	4.69E+04	5.40E+04	5.93E+04	4.06E+04	6.06E+04	5.87E+04	4.85E+04	4.03E+04
736	Terpinine-4-ol	Others	1.28E+04	3.08E+04	2.48E+04	2.05E+04	2.90E+04	3.02E+04	2.38E+04	2.91E+04	2.09E+04	1.96E+04	1.50E+04	3.09E+04
737	Isoalantolactone	Others	2.69E+04	3.06E+04	3.15E+04	4.37E+04	4.41E+04	3.14E+04	2.19E+04	3.03E+04	2.81E+04	3.91E+04	2.95E+04	2.78E+04
738	Lactupicrin	Others	1.06E+04	6.73E+03	6.08E+03	5.21E+03	9.72E+03	7.31E+03	4.51E+03	5.29E+03	3.96E+03	4.64E+03	4.10E+03	9.12E+03
739	Bruceine D	Others	2.14E+04	2.92E+04	1.90E+04	1.51E+04	1.96E+04	2.93E+04	1.51E+04	1.43E+04	2.65E+04	7.55E+03	2.27E+04	1.72E+04
740	Pterosin D	Others	5.17E+03	2.73E+03	2.69E+03	3.49E+03	1.65E+03	2.70E+03	3.96E+03	2.70E+03	4.76E+03	1.90E+03	1.66E+03	2.16E+03
741	Rutaevin	Others	7.85E+03	1.41E+04	8.65E+03	5.66E+03	1.36E+04	5.51E+03	1.31E+04	1.92E+04	1.00E+01	5.68E+03	2.69E+03	9.46E+03
742	Abietic acid	Others	2.00E+04	2.73E+04	2.04E+04	3.25E+04	1.30E+04	1.13E+04	1.37E+04	7.40E+04	1.09E+04	1.10E+04	1.60E+04	1.36E+04
743	Curzerene	Others	8.31E+03	1.04E+04	8.33E+03	1.61E+04	9.89E+03	1.09E+04	5.17E+03	8.09E+03	1.05E+04	8.24E+03	7.01E+03	6.82E+03
744	Alantolactone	Others	3.60E+04	1.40E+04	2.32E+04	5.31E+04	4.16E+04	1.41E+04	2.12E+04	2.00E+04	2.81E+04	1.24E+04	3.71E+04	1.07E+04
745	Linderalactone	Others	5.91E+03	5.93E+03	3.17E+03	3.39E+03	4.17E+03	3.45E+03	4.31E+03	2.65E+03	4.72E+03	2.27E+03	1.60E+03	2.20E+03
746	Tenulin	Others	1.29E+04	6.50E+03	5.97E+04	1.28E+05	1.03E+05	1.18E+04	1.08E+05	1.15E+05	1.72E+04	2.27E+04	8.47E+04	1.59E+04
747	Cornin	Others	1.53E+03	7.03E+03	5.47E+03	1.60E+04	1.07E+04	1.53E+03	3.37E+03	3.36E+03	1.14E+04	3.69E+03	1.51E+03	8.26E+03
748	Sterebin E	Others	4.53E+03	5.37E+03	7.20E+03	7.00E+03	2.28E+04	3.17E+03	4.64E+03	4.18E+03	7.31E+03	8.74E+03	2.99E+03	3.79E+03
749	Ganoderal A	Others	8.07E+03	8.44E+03	7.60E+03	9.37E+03	1.74E+04	1.06E+04	3.88E+03	5.37E+03	7.47E+03	4.24E+03	6.31E+03	5.54E+03

750	Isopulegol	Others	3.92E+03	1.15E+03	4.23E+03	2.29E+03	6.97E+03	1.05E+04	2.48E+03	2.86E+03	3.68E+03	2.51E+03	1.75E+03	3.04E+03
751	Calenduloside E	Others	6.51E+03	7.13E+03	7.10E+03	1.35E+03	2.83E+04	3.44E+03	2.98E+03	1.11E+04	1.00E+01	1.00E+01	5.36E+03	5.53E+03
752	Testosterone	Others	1.46E+04	1.74E+04	1.96E+04	3.34E+04	6.55E+04	2.67E+04	7.18E+03	1.11E+04	1.09E+04	1.39E+04	1.65E+04	5.12E+03
753	Kaurenoic acid	Others	2.23E+05	2.08E+05	1.44E+05	5.55E+05	8.61E+05	3.32E+03	1.94E+04	5.10E+03	2.07E+04	7.19E+04	7.86E+04	9.52E+04
754	Ganoderic acid F	Others	1.44E+04	8.28E+03	2.10E+04	1.96E+04	9.93E+04	6.53E+03	1.07E+04	8.26E+03	2.34E+04	4.81E+03	7.85E+03	7.42E+03
755	Geniposidic acid	Others	1.30E+05	1.92E+03	2.00E+04	7.36E+02	1.28E+03	1.48E+05	1.45E+03	2.63E+03	5.36E+02	6.99E+02	3.78E+03	1.72E+03
756	N-Acetyl-D-glucosamine-1-phosphate	Others	4.21E+04	3.41E+04	4.21E+04	9.00E+00	9.00E+00	9.00E+00	5.60E+04	5.77E+04	4.66E+04	5.29E+04	5.93E+04	6.40E+04
757	Tryptamine	Others	8.12E+04	7.80E+04	8.12E+04	9.00E+00	9.00E+00	9.00E+00	1.91E+05	1.97E+05	1.78E+05	3.05E+04	2.75E+04	2.56E+04
758	Phthalic acid	Others	9.70E+03	8.35E+03	9.70E+03	9.00E+00	9.00E+00	9.00E+00	1.48E+04	2.14E+04	1.34E+04	1.04E+04	6.78E+03	1.08E+04
759	4-Hydroxymandelonitrile	Others	1.07E+06	7.52E+05	1.07E+06	5.50E+04	5.04E+04	5.29E+04	1.30E+05	1.30E+05	1.18E+05	2.74E+06	2.74E+06	2.57E+06
760	Solatriose	Others	2.71E+05	1.89E+05	2.70E+05	2.81E+04	2.72E+04	1.91E+04	1.95E+04	1.31E+04	1.47E+04	7.09E+05	6.83E+05	6.75E+05
761	D-Fructose-1,6-biphosphate	Others	6.62E+04	4.90E+04	6.64E+04	6.44E+03	6.43E+03	7.87E+03	8.12E+03	1.30E+04	1.10E+04	1.80E+05	1.59E+05	1.45E+05
762	Ergothioneine	Others	2.71E+05	2.99E+05	3.04E+04	1.24E+03	4.90E+03	6.31E+03	2.59E+02	1.02E+03	9.20E+02	1.71E+02	2.30E+05	1.60E+02
763	Nervonic acid	Others	3.15E+03	1.02E+04	9.93E+03	1.00E+01	2.77E+03	1.71E+03	1.11E+03	1.64E+03	3.06E+03	2.50E+03	3.96E+04	2.71E+04
764	Phosphoenolpyruvate	Others	4.15E+04	2.96E+04	4.10E+04	9.44E+03	9.33E+03	5.22E+03	2.40E+03	3.59E+03	5.27E+03	9.37E+04	1.08E+05	1.00E+05
765	Indole-3-lactic acid	Others	1.14E+04	8.39E+03	1.11E+04	3.13E+03	2.38E+03	1.44E+03	2.89E+03	1.99E+03	2.36E+03	2.91E+04	2.38E+04	2.52E+04
766	2,2-Dimethylsuccinic acid	Others	1.51E+05	1.07E+05	1.50E+05	3.76E+04	3.22E+04	3.55E+04	1.59E+04	1.33E+04	1.14E+04	3.48E+05	3.62E+05	3.85E+05
767	Beta-Tocopherol	Others	9.19E+03	2.00E+04	4.36E+04	5.46E+03	2.13E+04	7.86E+03	2.83E+04	1.05E+04	1.22E+04	1.10E+04	9.67E+03	2.48E+05
768	6-Aminocaproic acid	Others	3.15E+06	2.41E+06	3.14E+06	9.46E+05	9.36E+05	8.96E+05	7.19E+05	7.89E+05	8.24E+05	7.93E+06	6.21E+06	6.84E+06
769	LysoPC 20:4	Others	1.99E+04	1.87E+04	1.99E+04	2.87E+03	2.24E+03	2.93E+03	2.90E+04	2.93E+04	3.66E+04	1.50E+04	3.16E+04	1.28E+04
770	Glucose-1-phosphate	Others	7.04E+05	5.48E+05	7.10E+05	2.07E+05	1.86E+05	2.53E+05	1.74E+05	1.37E+05	1.54E+05	1.54E+06	1.73E+06	1.50E+06
771	D-Glucose 6-phosphate	Others	7.11E+05	5.57E+05	7.15E+05	2.14E+05	1.97E+05	2.47E+05	1.33E+05	1.68E+05	1.47E+05	1.71E+06	1.64E+06	1.48E+06
772	Methoxyindoleacetic acid	Others	6.74E+05	4.99E+05	6.73E+05	2.32E+05	2.31E+05	2.22E+05	8.88E+04	7.91E+04	8.17E+04	1.54E+06	1.51E+06	1.62E+06
773	Yohimbic acid monohydrate	Others	1.03E+04	4.35E+03	1.76E+04	9.37E+03	3.95E+03	4.91E+03	5.90E+03	3.39E+03	3.81E+03	2.19E+03	7.86E+03	1.09E+05
774	Piperidine	Others	2.82E+06	2.14E+06	2.82E+06	1.00E+06	9.60E+05	1.00E+06	6.81E+05	6.86E+05	6.85E+05	5.83E+06	6.24E+06	6.45E+06
775	6-Deoxyfagomine	Others	3.17E+06	2.44E+06	3.16E+06	1.25E+06	1.06E+06	1.12E+06	7.87E+05	6.95E+05	7.92E+05	6.59E+06	7.27E+06	6.96E+06

776	N-Benzylmethylene isomethylamine	Others	3.59E+06	2.78E+06	3.60E+06	1.31E+06	1.38E+06	1.36E+06	8.83E+05	8.52E+05	9.25E+05	7.37E+06	8.13E+06	7.89E+06
777	LysoPC 19:1	Others	4.84E+04	4.64E+04	4.85E+04	3.35E+03	4.35E+03	4.28E+03	1.15E+05	1.09E+05	9.24E+04	2.54E+04	1.80E+04	2.02E+04
778	Indole	Others	3.49E+04	2.82E+04	3.47E+04	1.73E+04	1.47E+04	1.58E+04	3.23E+03	4.37E+03	6.52E+03	7.52E+04	8.84E+04	6.96E+04
779	LysoPE 17:1 (2n isomer)	Others	7.10E+04	6.63E+04	7.07E+04	1.38E+04	1.25E+04	1.17E+04	1.27E+05	1.23E+05	1.20E+05	6.77E+04	5.45E+04	4.90E+04
780	3-Indolepropionic acid	Others	1.19E+04	9.26E+03	1.22E+04	6.21E+03	4.44E+03	8.49E+03	9.00E+00	9.00E+00	9.00E+00	2.72E+04	2.77E+04	2.97E+04
781	8(R)-HPETE	Others	1.38E+04	4.56E+04	1.57E+05	1.09E+04	2.07E+05	1.04E+04	7.83E+03	1.37E+04	8.51E+03	1.14E+04	3.42E+04	9.60E+05
782	O-Phosphorylethanolamine	Others	4.70E+04	3.83E+04	4.74E+04	1.98E+04	2.23E+04	2.29E+04	1.98E+04	1.54E+04	1.77E+04	9.35E+04	9.51E+04	9.25E+04
783	Benzoic acid, 3,4,5-trihydroxy-, (1R,5R,6R)-3-carboxy-5,6-dihydroxy-3-cyclohexen-1-yl ester	Others	4.40E+05	4.04E+05	4.39E+05	8.92E+04	7.27E+04	8.80E+04	7.81E+05	7.83E+05	7.15E+05	3.72E+05	3.31E+05	3.73E+05
784	Turanose	Others	6.74E+05	5.60E+05	6.63E+05	3.05E+05	3.11E+05	2.16E+05	5.18E+05	4.39E+05	4.18E+05	1.27E+06	1.00E+06	1.13E+06
785	Pahutoxin	Others	1.00E+01	1.22E+04	4.62E+03	1.00E+01	5.95E+03	1.00E+01	1.00E+01	9.57E+02	5.84E+03	2.90E+03	4.59E+03	1.67E+04
786	Fumaric acid	Others	1.95E+06	1.86E+06	1.95E+06	2.61E+05	2.62E+05	2.75E+05	3.85E+06	4.60E+06	3.43E+06	1.13E+06	1.11E+06	9.84E+05
787	Adipic Acid	Others	2.70E+05	2.39E+05	2.71E+05	8.66E+04	7.68E+04	9.25E+04	3.24E+05	3.43E+05	3.08E+05	3.57E+05	3.24E+05	3.41E+05
788	2-Methylglutaric acid	Others	2.19E+05	1.95E+05	2.18E+05	7.30E+04	7.51E+04	6.39E+04	3.01E+05	2.96E+05	2.76E+05	2.05E+05	2.74E+05	2.56E+05
789	D-Pantothenic Acid	Others	7.17E+05	6.03E+05	7.18E+05	3.55E+05	3.76E+05	3.66E+05	3.82E+05	4.33E+05	3.98E+05	1.28E+06	1.23E+06	1.27E+06
790	Domesticoside	Others	2.51E+05	2.14E+05	2.49E+05	1.30E+05	1.24E+05	1.12E+05	1.85E+05	1.80E+05	1.78E+05	3.96E+05	4.11E+05	4.05E+05
791	Sarmentosin	Others	4.76E+04	4.23E+04	4.72E+04	1.78E+04	1.63E+04	1.44E+04	5.48E+04	6.40E+04	6.78E+04	5.07E+04	5.30E+04	5.67E+04
792	Methyl linoleate	Others	5.24E+04	5.15E+04	3.88E+04	1.17E+04	2.61E+04	3.31E+04	1.09E+04	6.11E+03	9.27E+03	4.56E+04	1.22E+05	5.72E+04
793	Alkannin	Others	1.73E+04	1.45E+04	2.00E+04	9.62E+03	4.90E+03	1.67E+04	1.25E+04	1.31E+04	1.90E+04	1.33E+04	4.20E+04	3.81E+04
794	6-Hydroxy-5-methoxyindole glucuronide	Others	5.81E+03	4.19E+03	3.22E+03	1.22E+03	1.80E+03	2.86E+03	1.78E+03	1.00E+01	1.78E+03	1.03E+04	4.93E+03	2.30E+03
795	Isodiospyrin	Others	8.57E+03	5.34E+03	8.72E+03	3.82E+03	5.77E+03	2.75E+03	1.06E+04	3.45E+03	1.06E+04	3.47E+03	9.32E+03	2.38E+04
796	Undecanolactone	Others	6.85E+03	3.54E+04	2.15E+04	8.68E+03	7.86E+03	7.28E+03	3.68E+04	3.96E+04	1.30E+04	2.67E+04	3.19E+04	8.91E+03
797	Rosiridoside B	Others	2.25E+05	2.08E+05	2.27E+05	7.25E+04	7.33E+04	8.11E+04	2.89E+05	3.69E+05	3.59E+05	2.34E+05	1.89E+05	2.18E+05
798	Strychnine	Others	5.38E+02	1.56E+03	7.34E+02	3.50E+02	8.92E+02	1.77E+02	1.00E+01	6.15E+02	3.58E+02	5.33E+02	1.30E+03	1.99E+03
799	Argininosuccinic acid	Others	1.96E+05	1.79E+05	1.96E+05	8.96E+04	9.49E+04	8.67E+04	2.17E+05	2.38E+05	2.08E+05	2.27E+05	2.68E+05	2.25E+05
800	12-OPDA	Others	3.96E+03	1.33E+04	6.02E+03	3.38E+03	6.94E+02	3.81E+03	6.85E+03	3.82E+03	1.31E+04	2.08E+03	7.64E+03	1.02E+04

801	5(S)-HPETE	Others	3.38E+03	4.80E+04	3.14E+04	1.38E+03	5.86E+04	7.17E+03	3.75E+03	1.27E+04	2.00E+03	6.19E+03	3.37E+03	1.58E+05
802	LysoPE 16:1	Others	1.95E+05	1.88E+05	1.94E+05	5.21E+04	4.94E+04	3.98E+04	3.71E+05	3.76E+05	3.64E+05	1.30E+05	1.23E+05	9.73E+04
803	Folinic acid	Others	5.72E+03	1.32E+04	1.06E+04	6.64E+03	1.00E+01	1.47E+04	4.40E+03	1.00E+01	1.31E+04	1.43E+04	2.64E+04	1.18E+04
804	Zygadenine	Others	4.22E+05	4.14E+05	4.21E+05	4.72E+04	4.24E+04	3.91E+04	1.05E+06	9.64E+05	9.72E+05	9.48E+04	1.07E+05	1.00E+05
805	6'-O-Glucosylaucubin	Others	4.13E+04	3.88E+04	4.14E+04	2.58E+04	2.40E+04	2.65E+04	3.31E+04	3.41E+04	3.52E+04	6.77E+04	6.40E+04	4.62E+04
806	Vitamin K1	Others	3.81E+03	7.05E+03	7.05E+03	2.73E+03	3.22E+03	6.02E+03	4.40E+03	3.27E+03	1.15E+04	8.60E+03	6.32E+03	1.30E+04
807	D-Ribose	Others	3.95E+05	3.52E+05	3.99E+05	2.69E+05	3.14E+05	3.00E+05	1.82E+05	1.75E+05	1.70E+05	7.41E+05	6.67E+05	6.45E+05
808	LysoPE 16:1 (2n isomer)	Others	2.01E+05	1.94E+05	2.01E+05	5.36E+04	5.12E+04	5.27E+04	3.71E+05	3.89E+05	3.79E+05	1.27E+05	1.29E+05	1.10E+05
809	2-Hydroxy-4-methylpentanoic acid	Others	1.81E+04	1.62E+04	1.93E+04	9.61E+03	1.24E+04	1.88E+04	1.40E+04	5.80E+03	8.91E+03	3.12E+04	2.88E+04	3.41E+04
810	Vitamin D2 (Ergocalciferol)	Others	1.65E+04	8.32E+03	8.16E+03	4.11E+03	6.17E+03	6.66E+03	4.91E+03	4.14E+03	5.38E+03	2.09E+03	2.77E+03	3.32E+04
811	Dimethylbenzimidazole	Others	5.45E+03	4.70E+03	4.54E+03	5.00E+03	1.14E+03	1.11E+03	2.26E+03	1.08E+04	5.09E+03	4.48E+03	6.02E+03	5.41E+03
812	Physcion 1-O-beta-D-glucoside	Others	6.39E+03	3.12E+03	2.60E+03	1.75E+03	3.46E+03	1.00E+01	1.66E+03	2.09E+03	2.19E+03	1.00E+01	6.30E+03	5.11E+03
813	D-Arabitol	Others	1.56E+05	1.43E+05	1.54E+05	8.06E+04	7.83E+04	6.66E+04	1.94E+05	2.08E+05	2.03E+05	1.64E+05	1.54E+05	1.68E+05
814	(S)-2-Acetolactate	Others	3.37E+03	5.88E+03	5.27E+03	2.72E+03	3.43E+03	3.88E+03	4.05E+03	3.85E+03	5.55E+03	4.95E+03	2.92E+03	1.36E+04
815	LysoPC 16:1 (2n isomer)	Others	8.13E+03	6.19E+03	4.61E+05	4.68E+04	5.29E+04	5.38E+04	1.09E+06	1.12E+06	1.06E+06	1.07E+05	1.07E+05	1.11E+05
816	Ribitol	Others	2.32E+05	2.16E+05	2.34E+05	1.14E+05	1.03E+05	1.32E+05	3.10E+05	3.21E+05	2.97E+05	2.32E+05	2.21E+05	2.60E+05
817	Palmitoylethanolamide	Others	1.81E+04	1.79E+04	1.16E+04	1.62E+04	1.00E+01	1.26E+04	1.00E+01	1.45E+04	8.80E+03	1.22E+04	2.84E+04	1.67E+04
818	(-)-Huperzine A	Others	6.43E+03	1.66E+03	1.51E+04	1.20E+03	3.08E+04	3.86E+03	1.00E+01	1.29E+04	1.71E+03	1.69E+03	3.40E+04	3.57E+04
819	Choline Alfoscerate	Others	5.62E+06	5.43E+06	5.67E+06	1.43E+06	1.68E+06	1.78E+06	1.06E+07	1.08E+07	1.09E+07	3.13E+06	3.11E+06	3.33E+06
820	Acetylcholine	Others	4.33E+05	3.91E+05	4.32E+05	3.46E+05	3.25E+05	3.39E+05	2.68E+05	3.05E+05	2.68E+05	6.25E+05	6.55E+05	6.70E+05
821	L-Acetylcarnitine	Others	2.69E+04	3.74E+04	1.88E+04	1.18E+04	1.24E+04	1.31E+04	1.02E+04	1.73E+04	2.51E+04	1.85E+04	3.31E+04	2.02E+04
822	Citrostadienol	Others	1.10E+04	8.33E+03	7.23E+03	8.02E+03	8.11E+03	1.49E+03	5.88E+03	3.70E+03	5.06E+03	1.52E+04	6.18E+03	1.22E+04
823	L-Tartaric acid	Others	3.32E+04	3.07E+04	3.07E+04	4.09E+04	2.23E+04	2.10E+04	1.62E+04	1.07E+04	1.54E+04	6.23E+04	5.66E+04	4.15E+04
824	1-Aminocyclopropane-1-carboxylic acid	Others	2.65E+05	2.43E+05	2.65E+05	2.32E+05	2.35E+05	2.25E+05	1.15E+05	1.09E+05	1.23E+05	4.53E+05	4.56E+05	4.01E+05
825	Acitretin	Others	2.11E+03	1.33E+03	1.80E+03	2.25E+02	7.09E+02	2.56E+03	1.38E+03	4.57E+02	2.74E+03	1.15E+03	4.71E+03	7.20E+02
826	UDP-N-acetyl-alpha-D-glucosamine	Others	4.29E+04	4.09E+04	4.28E+04	1.41E+04	1.55E+04	1.36E+04	7.78E+04	7.62E+04	7.92E+04	2.90E+04	2.15E+04	2.99E+04

827	LysoPE 15:1 (2n isomer)	Others	3.34E+04	3.15E+04	3.31E+04	2.21E+04	1.81E+04	2.03E+04	3.97E+04	4.01E+04	3.59E+04	3.83E+04	3.76E+04	3.52E+04
828	2-Hydroxy-6-oxo-6-(2-carboxyphenyl)-hexa-2,4-dienoate	Others	2.98E+03	6.41E+03	2.43E+03	1.70E+03	2.57E+03	1.11E+03	1.55E+03	3.25E+03	1.14E+03	1.12E+03	6.03E+03	2.69E+03
829	3-Hydroxyanthranilic acid	Others	5.80E+04	5.39E+04	5.81E+04	3.82E+04	4.60E+04	3.89E+04	5.19E+04	4.83E+04	5.78E+04	7.82E+04	7.20E+04	7.13E+04
830	D-Sedoheptuiose 7-phosphate	Others	1.56E+06	1.45E+06	1.57E+06	1.32E+06	1.20E+06	1.46E+06	9.83E+05	9.13E+05	9.39E+05	2.49E+06	2.29E+06	2.32E+06
831	4-Aminobenzoic acid	Others	1.26E+04	1.23E+04	1.26E+04	7.83E+03	8.86E+03	7.92E+03	1.04E+04	1.60E+04	1.37E+04	1.66E+04	1.68E+04	1.06E+04
832	Astrocasine	Others	1.69E+03	2.72E+03	1.92E+03	3.09E+03	1.67E+03	1.00E+01	1.49E+03	1.49E+03	2.15E+03	2.67E+03	4.24E+03	1.62E+03
833	DL-Dihydrozeatin	Others	6.55E+03	1.13E+04	7.24E+03	3.55E+03	6.12E+03	4.23E+03	9.22E+03	5.68E+03	7.90E+03	2.95E+03	1.56E+04	6.18E+03
834	Erythritol	Others	1.05E+04	9.07E+03	1.42E+04	1.18E+04	1.61E+04	5.92E+03	1.08E+04	8.23E+03	1.25E+04	1.31E+04	3.04E+04	1.66E+04
835	LysoPC 17:2	Others	5.40E+04	5.31E+04	5.39E+04	8.75E+03	7.78E+03	7.29E+03	1.22E+05	1.32E+05	1.19E+05	1.45E+04	1.30E+04	1.48E+04
836	Glucarate O-Phosphoric acid	Others	3.14E+06	2.93E+06	3.18E+06	2.58E+06	2.38E+06	2.96E+06	2.15E+06	2.06E+06	1.96E+06	5.00E+06	4.32E+06	4.63E+06
837	Rosiridin	Others	5.67E+04	5.28E+04	5.69E+04	4.43E+04	4.43E+04	4.59E+04	4.57E+04	4.27E+04	4.58E+04	8.28E+04	7.13E+04	7.67E+04
838	2-Hydroxyisobutyric acid	Others	2.93E+04	2.73E+04	2.83E+04	1.47E+04	1.37E+04	7.00E+03	5.05E+04	4.94E+04	4.55E+04	2.20E+04	1.59E+04	2.26E+04
839	Xylitol	Others	2.51E+05	2.44E+05	2.51E+05	6.37E+04	8.07E+04	6.49E+04	5.26E+05	5.33E+05	4.56E+05	1.15E+05	1.11E+05	1.24E+05
840	Tetracenomycin C	Others	8.01E+03	7.66E+03	7.69E+03	6.62E+03	5.64E+03	4.13E+03	7.41E+03	1.02E+04	6.81E+03	9.23E+03	1.12E+04	6.89E+03
841	DL-2-Aminoadipic acid	Others	7.35E+05	6.89E+05	7.29E+05	5.98E+05	5.53E+05	5.53E+05	6.38E+05	6.53E+05	6.16E+05	9.68E+05	9.32E+05	9.19E+05
842	Oxoglaucine	Others	4.01E+04	2.56E+04	2.77E+04	1.95E+04	1.66E+04	2.46E+04	2.34E+04	2.08E+04	3.55E+04	3.83E+04	3.38E+04	2.82E+04
843	Citric Acid	Others	3.03E+07	2.81E+07	3.03E+07	2.48E+07	2.40E+07	2.49E+07	2.51E+07	2.50E+07	2.35E+07	3.85E+07	3.86E+07	4.30E+07
844	Norbixin	Others	2.79E+03	3.36E+03	1.29E+03	7.75E+02	1.27E+03	9.43E+02	1.18E+03	1.50E+03	5.71E+02	2.29E+03	1.27E+03	1.27E+03
845	N-Methylcoclaurine	Others	1.60E+04	1.81E+04	2.66E+04	1.99E+04	1.41E+04	1.71E+04	3.89E+04	3.44E+04	2.59E+04	2.59E+04	3.47E+04	2.19E+04
846	Pantetheine	Others	1.59E+04	1.51E+04	1.58E+04	3.70E+03	4.44E+03	2.60E+03	3.42E+04	3.32E+04	3.46E+04	4.20E+03	3.83E+03	9.33E+03
847	D-(+)-Sucrose	Others	2.88E+06	2.69E+06	2.88E+06	2.70E+06	2.53E+06	2.70E+06	1.65E+06	1.54E+06	1.82E+06	4.32E+06	4.21E+06	4.23E+06
848	Piperlonguminine	Others	8.03E+03	3.91E+03	6.31E+03	5.00E+03	3.88E+03	4.23E+03	7.38E+03	4.91E+03	9.09E+03	4.21E+03	1.05E+04	6.34E+03
849	D-Proline betaine	Others	1.18E+05	1.17E+05	1.18E+05	2.17E+04	1.87E+04	2.03E+04	2.67E+05	2.99E+05	2.44E+05	3.53E+04	2.74E+04	3.45E+04
850	LysoPC 19:2 (2n isomer)	Others	7.74E+04	7.63E+04	7.80E+04	1.75E+04	2.04E+04	2.22E+04	1.78E+05	1.70E+05	1.36E+05	3.13E+04	3.42E+04	3.05E+04
851	Dictamnine	Others	2.43E+03	1.41E+03	2.67E+03	1.84E+03	2.78E+03	1.99E+03	1.46E+03	2.66E+03	1.91E+03	2.99E+03	3.88E+03	3.67E+03
852	Reticuline	Others	5.94E+02	4.50E+03	4.14E+03	2.43E+03	1.71E+03	6.61E+03	1.00E+01	2.90E+03	4.91E+03	2.94E+03	5.27E+03	8.75E+03

853	Nicotinic Acid Methyl Ester (Methyl Nicotinate)	Others	4.65E+05	4.31E+05	4.63E+05	4.70E+05	4.78E+05	4.56E+05	2.05E+05	1.96E+05	1.85E+05	7.33E+05	7.23E+05	7.27E+05
854	9-Methoxy-alpha-lapachone	Others	1.93E+04	1.97E+04	1.19E+04	3.70E+03	1.29E+04	1.06E+04	7.95E+03	1.54E+04	5.95E+03	3.99E+03	9.89E+03	2.84E+04
855	5-Carboxyvanillic acid	Others	1.87E+03	3.55E+03	2.75E+03	1.98E+03	4.17E+03	9.33E+02	7.64E+02	2.54E+03	2.73E+03	4.15E+03	2.58E+03	4.16E+03
856	Dimethylmalonic acid	Others	5.05E+05	5.01E+05	5.05E+05	5.02E+04	5.30E+04	5.26E+04	1.32E+06	1.29E+06	1.08E+06	7.35E+04	8.09E+04	8.13E+04
857	Anandamide	Others	3.50E+03	4.52E+03	3.15E+03	3.47E+03	2.74E+03	1.06E+03	3.14E+03	3.33E+03	3.98E+03	2.23E+03	2.07E+03	6.65E+03
858	Canthaxanthin	Others	2.49E+03	6.85E+03	5.12E+03	2.06E+03	3.96E+03	4.24E+03	1.14E+04	2.69E+03	3.13E+03	8.70E+03	3.67E+03	3.10E+03
859	8-Amino-7-oxononanoic acid	Others	1.77E+03	1.72E+03	1.38E+03	1.38E+03	1.29E+03	3.09E+02	1.01E+03	2.72E+03	1.20E+03	1.09E+03	2.13E+03	1.28E+03
860	β-Hydroxyisovaleric acid	Others	3.65E+06	3.54E+06	3.64E+06	1.73E+06	1.57E+06	1.66E+06	6.40E+06	6.26E+06	5.86E+06	2.42E+06	2.46E+06	2.52E+06
861	2-Hydroxyisocaproic acid	Others	8.93E+05	8.82E+05	9.01E+05	2.37E+05	2.26E+05	2.98E+05	1.98E+06	1.86E+06	1.71E+06	3.85E+05	3.53E+05	3.89E+05
862	Allocryptopine	Others	6.12E+03	3.27E+03	3.43E+03	1.70E+03	3.07E+03	3.26E+03	2.29E+03	4.55E+03	2.35E+03	4.62E+03	4.71E+03	2.55E+03
863	Trigonelline	Others	5.51E+03	2.64E+04	2.50E+04	1.26E+04	1.21E+04	1.72E+04	5.76E+04	1.89E+04	3.25E+04	2.37E+04	2.45E+04	1.37E+04
864	L-Citramalic acid	Others	8.24E+04	7.67E+04	8.37E+04	7.45E+04	7.98E+04	8.45E+04	5.62E+04	3.75E+04	6.27E+04	9.76E+04	1.21E+05	1.30E+05
865	Gibberellin A4	Others	2.61E+03	3.10E+03	2.40E+03	2.41E+03	1.88E+03	2.18E+03	1.63E+03	2.40E+03	1.68E+03	2.76E+03	4.15E+03	2.49E+03
866	Estriol 3-sulfate 16-glucuronide	Others	1.12E+04	8.37E+03	4.68E+03	4.62E+03	6.10E+03	3.07E+03	2.20E+03	4.56E+03	1.48E+03	1.50E+03	1.32E+04	5.35E+03
867	Prephenate	Others	2.35E+03	1.77E+03	3.01E+03	1.29E+03	1.52E+03	2.76E+03	6.40E+03	3.48E+03	1.82E+03	1.74E+03	2.63E+03	3.73E+03
868	LysoPC 20:3	Others	1.94E+04	1.95E+04	1.97E+04	2.06E+03	4.18E+03	4.83E+03	4.54E+04	4.90E+04	3.82E+04	4.76E+03	7.22E+03	4.07E+03
869	(-)-alpha-Narcotine	Others	1.06E+04	1.17E+04	8.64E+03	5.48E+03	5.95E+03	1.12E+04	2.97E+03	7.02E+03	9.31E+03	1.21E+04	1.02E+04	1.05E+04
870	2-Methylsuccinic acid	Others	6.74E+06	6.69E+06	6.74E+06	8.03E+05	8.08E+05	7.96E+05	1.66E+07	1.63E+07	1.59E+07	1.17E+06	1.13E+06	1.18E+06
871	Homobaldrinal	Others	2.11E+03	8.33E+02	2.06E+03	1.33E+03	2.87E+03	1.29E+03	1.15E+03	1.70E+03	1.58E+03	3.33E+03	2.26E+03	2.34E+03
872	Sinapine	Others	6.37E+03	2.42E+03	2.79E+03	6.05E+02	4.90E+03	5.97E+02	5.83E+02	2.41E+03	5.09E+03	3.33E+03	5.44E+03	1.00E+01
873	(R)-Acetoin	Others	2.75E+04	2.88E+04	4.07E+04	1.58E+04	6.91E+04	1.81E+04	5.05E+04	2.41E+04	1.70E+04	4.82E+04	7.43E+04	2.44E+04
874	Malonic acid	Others	2.46E+06	2.33E+06	2.45E+06	2.33E+06	2.40E+06	2.26E+06	1.61E+06	1.77E+06	1.75E+06	3.36E+06	3.19E+06	3.30E+06
875	N-Oleoylethanolamine	Others	6.69E+05	6.52E+05	6.73E+05	3.56E+05	3.48E+05	3.91E+05	1.05E+06	1.07E+06	9.90E+05	5.13E+05	4.98E+05	5.22E+05
876	Cevadine	Others	1.08E+04	6.89E+03	1.07E+04	9.24E+03	7.68E+03	9.11E+03	1.66E+04	8.59E+03	7.43E+03	1.32E+04	8.56E+03	1.44E+04
877	2-Hydroxybutyric Acid	Others	1.96E+04	1.92E+04	1.93E+04	9.95E+03	8.88E+03	7.50E+03	3.32E+04	3.67E+04	3.17E+04	1.24E+04	1.33E+04	1.09E+04
878	Symlandine	Others	5.19E+03	1.24E+03	3.36E+03	2.81E+03	2.88E+03	1.98E+03	3.91E+03	3.25E+03	4.18E+03	4.61E+03	2.39E+03	3.66E+03

879	Trehalose 6-phosphate	Others	4.53E+04	4.43E+04	4.58E+04	2.96E+04	3.33E+04	3.39E+04	5.45E+04	5.29E+04	5.81E+04	4.65E+04	4.59E+04	4.13E+04
880	Traumatic acid	Others	1.29E+03	3.87E+03	2.32E+03	2.09E+03	2.65E+03	1.85E+03	1.41E+03	2.92E+03	6.75E+02	2.70E+03	3.56E+03	2.81E+03
881	Ethyl caproate	Others	1.73E+05	1.65E+05	1.38E+05	9.02E+04	1.19E+05	9.75E+04	1.15E+05	2.29E+05	1.25E+05	1.09E+05	1.92E+05	1.19E+05
882	Isocitric Acid	Others	1.47E+07	1.40E+07	1.46E+07	1.39E+07	1.35E+07	1.31E+07	1.16E+07	1.18E+07	1.19E+07	1.71E+07	1.94E+07	1.88E+07
883	4-Pyridoxolactone	Others	1.17E+03	5.39E+03	4.26E+03	1.18E+03	8.24E+03	1.00E+01	4.69E+03	2.90E+03	5.39E+03	5.22E+03	6.44E+03	1.19E+03
884	Delta-Nonalactone	Others	6.44E+05	7.23E+05	3.79E+05	7.40E+04	6.55E+04	3.25E+04	1.44E+06	1.25E+06	1.39E+04	5.51E+04	1.52E+05	2.42E+04
885	3-Methyl-1-butylamine	Others	1.77E+04	1.25E+04	1.30E+04	1.30E+04	9.40E+03	1.09E+04	8.37E+03	2.14E+04	9.52E+03	6.31E+03	2.52E+04	1.32E+04
886	Raffinose	Others	7.76E+03	2.25E+04	1.01E+04	8.29E+02	2.22E+04	1.00E+01	1.00E+01	1.36E+04	1.45E+04	1.00E+01	2.18E+04	8.67E+03
887	LysoPC 18:2 (2n isomer)	Others	1.99E+06	1.98E+06	1.99E+06	2.38E+05	2.42E+05	2.25E+05	4.91E+06	5.03E+06	4.58E+06	3.13E+05	3.05E+05	3.09E+05
888	UDP-2-acetamido-3-amino-2,3-dideoxy-alpha-D-glucuronate	Others	9.97E+03	1.72E+04	7.78E+03	8.78E+03	4.69E+03	7.05E+03	8.90E+03	7.23E+03	7.42E+03	5.96E+03	1.06E+04	1.04E+04
889	Cinidilide	Others	1.45E+04	1.49E+04	1.52E+04	9.60E+03	4.67E+03	1.46E+04	1.93E+04	2.12E+04	2.37E+04	1.33E+04	1.31E+04	1.14E+04
890	Gentioflavin	Others	6.84E+03	1.07E+04	1.64E+04	1.40E+04	1.19E+04	1.71E+04	6.82E+03	2.24E+04	1.71E+04	2.43E+04	1.60E+04	1.57E+04
891	Nandrolone	Others	6.17E+03	8.66E+03	4.61E+03	1.16E+04	1.75E+03	3.39E+03	5.21E+03	4.82E+03	1.00E+01	9.24E+03	6.36E+03	6.12E+03
892	4-Sulfobenzoate	Others	8.26E+03	1.28E+04	8.08E+03	2.88E+03	1.11E+04	6.87E+03	9.76E+03	7.31E+03	2.64E+03	1.04E+04	1.09E+04	5.68E+03
893	Tetrodotoxin	Others	1.93E+04	2.11E+03	7.62E+03	4.57E+03	7.94E+03	6.63E+03	3.63E+03	3.75E+03	1.44E+04	8.61E+03	9.11E+03	6.93E+03
894	Anthranilic Acid	Others	1.15E+04	1.12E+04	1.19E+04	7.39E+03	8.47E+03	1.03E+04	1.30E+04	1.75E+04	1.21E+04	8.63E+03	1.25E+04	1.25E+04
895	D-(-)-Threose	Others	4.00E+04	3.72E+04	3.89E+04	4.44E+04	4.08E+04	3.58E+04	2.88E+04	2.70E+04	2.31E+04	5.11E+04	4.66E+04	5.80E+04
896	(S)-Actinidine	Others	6.38E+03	4.18E+03	5.44E+03	5.75E+03	6.76E+03	2.70E+03	5.20E+03	5.49E+03	3.87E+03	5.87E+03	5.88E+03	7.75E+03
897	Physalin D	Others	6.75E+03	1.40E+04	1.11E+04	1.02E+04	1.43E+04	7.49E+03	1.45E+04	5.99E+03	5.17E+03	7.50E+03	1.54E+04	1.81E+04
898	Cocamidopropyl betaine	Others	1.30E+04	1.50E+04	1.78E+04	5.22E+03	5.13E+03	4.33E+04	8.84E+03	9.14E+03	7.12E+03	2.22E+04	1.93E+04	2.72E+04
899	Lincomycin	Others	9.50E+03	1.91E+04	2.41E+04	2.70E+04	2.56E+04	1.17E+04	1.34E+04	2.84E+04	3.14E+04	3.51E+04	1.64E+04	3.06E+04
900	Quinic Acid	Others	9.82E+05	9.48E+05	9.79E+05	9.18E+05	9.32E+05	8.91E+05	8.39E+05	7.87E+05	8.91E+05	1.13E+06	1.20E+06	1.16E+06
901	N-Acetyl-D-glucosamine	Others	3.33E+05	3.23E+05	3.31E+05	3.17E+05	3.12E+05	3.01E+05	3.01E+05	2.74E+05	2.81E+05	3.77E+05	4.24E+05	3.81E+05
902	(2R,3S)-3-methylmalic acid	Others	5.05E+05	4.92E+05	5.05E+05	2.97E+05	2.73E+05	2.99E+05	8.75E+05	8.08E+05	6.93E+05	3.65E+05	3.27E+05	3.99E+05
903	2-Picolinic acid	Others	5.88E+05	5.71E+05	5.87E+05	5.35E+05	5.66E+05	5.33E+05	5.02E+05	4.96E+05	5.55E+05	7.02E+05	6.81E+05	6.63E+05
904	Pregabalin	Others	4.17E+04	3.52E+04	3.23E+04	1.77E+04	2.43E+04	3.53E+04	3.72E+04	4.11E+04	2.57E+04	3.56E+04	2.93E+04	2.99E+04

905	Methylisopelletierine	Others	6.45E+03	7.44E+03	6.01E+03	3.53E+03	9.89E+03	4.86E+03	6.08E+03	2.44E+03	2.55E+03	7.22E+03	1.04E+04	4.65E+03
906	Nicotinamide	Others	5.25E+05	5.10E+05	5.21E+05	4.98E+05	4.68E+05	4.69E+05	5.10E+05	5.20E+05	4.76E+05	5.45E+05	5.90E+05	5.91E+05
907	Deoxyvasicinone	Others	5.17E+03	2.31E+03	2.94E+03	2.25E+03	4.48E+03	1.11E+03	2.82E+03	2.85E+03	2.85E+03	3.02E+03	5.28E+03	1.11E+03
908	Choline	Others	9.29E+06	9.02E+06	9.27E+06	1.03E+07	9.86E+06	1.02E+07	6.05E+06	6.05E+06	5.87E+06	1.17E+07	1.22E+07	1.23E+07
909	Cheilanthifoline	Others	1.02E+04	1.06E+03	1.11E+04	1.77E+04	9.49E+03	5.52E+03	2.26E+04	5.91E+03	6.55E+03	6.53E+03	1.75E+04	1.48E+04
910	4-Oxopentanoic Acid	Others	7.46E+04	7.35E+04	7.47E+04	3.62E+04	3.80E+04	3.70E+04	1.29E+05	1.37E+05	1.25E+05	4.73E+04	3.86E+04	4.63E+04
911	alpha-Irone	Others	2.22E+04	1.76E+04	2.75E+04	1.60E+04	3.38E+04	3.35E+04	1.73E+04	9.98E+03	2.78E+04	3.05E+04	3.52E+04	3.19E+04
912	L-Hypoglycin A	Others	7.59E+03	7.42E+02	7.01E+03	3.65E+03	7.10E+03	7.01E+03	6.66E+03	7.64E+03	6.91E+03	7.87E+03	8.92E+03	4.00E+03
913	LysoPC 18:3 (2n isomer)	Others	8.76E+06	8.73E+06	8.75E+06	1.14E+06	1.22E+06	1.11E+06	2.21E+07	2.11E+07	2.04E+07	1.38E+06	1.37E+06	1.31E+06
914	Hypotaurine	Others	3.87E+03	3.15E+03	3.76E+03	5.31E+03	2.49E+03	2.88E+03	3.02E+03	2.78E+03	6.45E+03	3.52E+03	5.85E+03	3.10E+03
915	Beta-Tocotrienol	Others	1.80E+04	1.26E+04	1.09E+04	1.81E+04	2.99E+03	1.41E+04	7.06E+03	2.29E+03	2.01E+04	1.56E+04	1.50E+04	1.05E+04
916	Citraconic acid	Others	1.32E+05	1.30E+05	1.32E+05	1.36E+05	1.12E+05	1.32E+05	1.36E+05	1.25E+05	1.08E+05	1.51E+05	1.41E+05	1.51E+05
917	3-Aminosalicylic acid	Others	8.88E+03	7.17E+03	6.47E+03	1.93E+04	1.36E+04	9.00E+00	9.00E+00	9.00E+00	9.00E+00	1.47E+04	9.80E+03	1.37E+04
918	Cadaverine	Others	2.06E+06	2.03E+06	2.05E+06	8.18E+05	7.78E+05	7.38E+05	4.11E+06	4.05E+06	4.01E+06	8.98E+05	8.85E+05	9.29E+05
919	3-Methyl-2-Oxobutanoic acid	Others	3.72E+05	3.68E+05	3.72E+05	1.94E+05	1.92E+05	1.93E+05	6.67E+05	6.57E+05	5.97E+05	2.33E+05	2.07E+05	2.32E+05
920	N-Acetyl-2-phenylethylamine	Others	1.06E+04	7.89E+03	2.87E+03	3.05E+03	3.09E+03	2.39E+03	1.83E+03	2.01E+03	3.80E+03	1.95E+03	4.06E+03	3.84E+03
921	Rosiridoside A	Others	9.78E+04	9.39E+04	9.52E+04	9.48E+04	8.50E+04	7.39E+04	1.05E+05	7.60E+04	1.30E+05	9.26E+04	9.31E+04	1.05E+05
922	Rhombifoline	Others	1.14E+03	3.73E+03	2.53E+03	1.67E+03	2.76E+03	2.31E+03	1.13E+03	1.13E+03	5.18E+03	1.12E+03	2.86E+03	3.75E+03
923	(S)-2-Hydroxyglutaric Acid	Others	4.92E+05	4.84E+05	4.93E+05	3.07E+05	3.23E+05	3.16E+05	8.36E+05	8.37E+05	5.50E+05	3.53E+05	3.47E+05	3.81E+05
924	N-Lauryldiethanolamine	Others	1.32E+04	1.27E+04	1.35E+04	1.20E+04	1.25E+04	1.38E+04	1.41E+04	1.03E+04	1.33E+04	1.17E+04	1.39E+04	1.82E+04
925	Synephrine	Others	1.05E+04	5.51E+03	9.60E+03	1.44E+04	4.28E+03	7.47E+03	5.61E+03	1.22E+04	1.76E+04	7.71E+03	1.68E+04	5.16E+03
926	D-Xylonic acid	Others	1.04E+07	1.03E+07	1.04E+07	9.94E+06	1.00E+07	9.63E+06	1.14E+07	9.96E+06	8.54E+06	1.09E+07	1.19E+07	1.08E+07
927	Histidinol	Others	6.21E+06	6.11E+06	6.23E+06	6.34E+06	6.40E+06	6.50E+06	5.04E+06	5.08E+06	4.99E+06	7.25E+06	7.28E+06	7.29E+06
928	Beta-D-Fructose 2-phosphate	Others	1.39E+05	1.24E+05	2.08E+05	8.62E+04	2.14E+05	2.17E+05	1.69E+05	1.27E+05	3.54E+05	2.19E+05	2.19E+05	1.48E+05
929	Deoxylapachol	Others	1.01E+04	7.23E+03	7.67E+03	5.15E+03	6.87E+03	8.77E+03	1.12E+04	6.55E+03	4.49E+03	8.78E+03	5.39E+03	9.34E+03
930	Furfuryl acetate	Others	1.78E+04	8.89E+03	1.29E+04	1.08E+04	1.99E+04	8.61E+03	1.34E+04	1.01E+04	7.61E+03	1.17E+04	1.18E+04	2.03E+04

931	2-Furanoic acid	Others	5.66E+05	5.58E+05	5.66E+05	4.48E+05	4.62E+05	4.51E+05	7.16E+05	7.02E+05	6.85E+05	5.06E+05	4.94E+05	5.14E+05
932	8-Methylnonenoate	Others	2.18E+05	2.91E+05	2.17E+05	1.67E+05	2.25E+05	2.66E+05	2.46E+05	2.55E+05	1.83E+04	1.91E+05	2.88E+05	2.48E+05
933	Betaine aldehyde	Others	3.08E+04	3.80E+04	2.98E+04	4.07E+04	2.54E+04	2.99E+04	2.84E+04	2.57E+04	2.35E+04	2.73E+04	4.50E+04	3.34E+04
934	Androstanediol	Others	2.32E+03	6.16E+03	2.23E+03	3.07E+03	3.92E+03	1.00E+01	5.71E+02	1.66E+03	3.99E+03	2.50E+03	3.47E+03	1.69E+03
935	2-Aminomuconate semialdehyde	Others	3.97E+03	1.75E+03	3.05E+03	2.41E+03	3.39E+03	3.03E+03	3.36E+03	2.47E+03	2.57E+03	2.27E+03	3.34E+03	4.01E+03
936	Isoxanthopterin	Others	8.35E+03	6.56E+03	8.51E+03	3.98E+03	7.92E+03	1.04E+04	8.76E+03	1.18E+04	5.03E+03	9.24E+03	7.03E+03	7.96E+03
937	Gibberellin A7	Others	2.14E+03	1.25E+03	1.83E+03	1.34E+03	2.14E+03	2.30E+03	1.45E+03	1.19E+03	1.29E+03	1.77E+03	9.61E+02	3.52E+03
938	Caffeine	Others	8.25E+02	2.19E+03	7.75E+03	9.14E+03	6.85E+03	6.70E+03	1.91E+04	2.69E+03	2.20E+03	1.60E+04	2.95E+03	5.54E+03
939	Spermine	Others	2.67E+07	2.62E+07	2.66E+07	2.73E+07	2.83E+07	2.63E+07	2.28E+07	2.29E+07	2.39E+07	2.91E+07	2.90E+07	3.02E+07
940	LysoPC 18:1 (2n isomer)	Others	1.47E+07	1.47E+07	1.47E+07	1.74E+06	1.95E+06	1.80E+06	3.65E+07	3.75E+07	3.41E+07	1.96E+06	2.01E+06	1.89E+06
941	Dodecanedioic aicd	Others	6.80E+02	6.61E+02	6.95E+02	7.70E+02	5.74E+02	8.89E+02	5.32E+02	7.20E+02	4.60E+02	8.01E+02	5.43E+02	1.04E+03
942	O-Phosphocholine	Others	7.84E+05	7.82E+05	7.69E+05	1.03E+06	9.14E+05	9.09E+05	4.19E+05	4.45E+05	4.54E+05	9.98E+05	1.09E+06	9.25E+05
943	D-Arabinono-1,4-lactone	Others	1.63E+05	1.60E+05	1.61E+05	1.39E+05	1.43E+05	1.29E+05	2.36E+05	1.65E+05	1.88E+05	1.32E+05	1.47E+05	1.50E+05
944	Benzoylformic acid	Others	4.83E+04	4.82E+04	4.79E+04	1.30E+04	1.20E+04	9.81E+03	1.21E+05	1.06E+05	9.71E+04	1.33E+04	1.25E+04	1.06E+04
945	DL-alpha-Tocopherylacetate	Others	4.03E+03	5.77E+03	2.98E+03	2.25E+03	1.15E+03	4.45E+03	4.32E+03	3.03E+03	2.69E+03	2.26E+03	2.54E+03	3.38E+03
946	Limonexic acid	Others	6.92E+03	2.74E+04	1.65E+04	2.61E+04	2.43E+04	1.25E+04	1.55E+04	7.23E+03	7.46E+03	3.03E+04	1.68E+04	1.83E+04
947	Diethanolamine	Others	1.07E+05	1.07E+05	1.07E+05	1.27E+05	1.19E+05	1.30E+05	7.39E+04	6.79E+04	7.55E+04	1.27E+05	1.38E+05	1.24E+05
948	Platyphylline	Others	9.36E+02	3.80E+03	1.77E+03	1.22E+03	7.24E+02	2.53E+03	1.06E+03	3.76E+03	1.47E+03	2.82E+03	6.80E+02	1.14E+03
949	2,6-Dimethylaniline	Others	1.52E+04	1.32E+04	9.61E+03	6.48E+03	1.34E+04	8.59E+03	8.14E+03	9.03E+03	8.13E+03	1.24E+04	8.43E+03	8.70E+03
950	(1α,5α)-8-Azabicyclo[3.2.1]octane-3α-ol	Others	1.50E+04	3.31E+04	2.65E+04	3.22E+04	2.96E+04	2.42E+04	2.52E+04	2.14E+04	2.28E+04	1.71E+04	4.58E+04	2.59E+04
951	Eprosartan	Others	1.00E+01	1.13E+04	3.16E+03	3.10E+03	5.72E+03	1.00E+01	1.00E+01	1.53E+03	8.88E+03	7.53E+03	1.56E+03	1.00E+01
952	Methyl (indol-3-yl)acetate	Others	4.90E+03	3.57E+03	3.10E+03	2.88E+03	4.53E+03	2.00E+03	2.10E+03	4.43E+03	2.12E+03	1.43E+03	4.26E+03	3.94E+03
953	22-Dehydroclerosterol	Others	3.47E+04	4.81E+04	4.10E+04	5.45E+04	5.32E+04	3.42E+04	5.26E+04	2.53E+04	1.88E+04	3.90E+04	4.20E+04	6.28E+04
954	Droserone	Others	3.81E+03	1.56E+04	1.08E+04	1.36E+04	5.94E+03	1.06E+04	1.47E+04	1.99E+04	4.66E+03	7.71E+03	1.16E+04	1.13E+04
955	Lubiprostone	Others	4.90E+06	3.80E+06	4.46E+06	3.97E+06	5.08E+06	3.22E+06	5.65E+06	5.05E+06	4.22E+06	5.21E+06	4.03E+06	3.17E+06
956	Bakkenolide A	Others	5.79E+04	4.68E+04	4.83E+04	2.85E+04	5.73E+04	5.31E+04	6.49E+04	3.68E+04	3.40E+04	4.29E+04	4.94E+04	4.78E+04

957	Streptozotocin	Others	1.22E+04	1.28E+04	1.26E+04	3.28E+03	1.19E+04	1.37E+04	1.80E+04	2.12E+04	7.23E+03	9.08E+03	1.49E+04	5.05E+03
958	1-Methylpiperidine-2-carboxylic acid	Others	2.13E+05	2.13E+05	2.13E+05	9.00E+00	9.00E+00	9.00E+00	5.20E+05	6.00E+05	5.86E+05	9.00E+00	9.00E+00	9.00E+00
959	LysoPC 19:2	Others	5.20E+04	5.20E+04	5.20E+04	9.00E+00	9.00E+00	9.00E+00	1.40E+05	1.32E+05	1.44E+05	9.00E+00	9.00E+00	9.00E+00
960	LysoPC 20:1	Others	1.91E+04	1.91E+04	1.91E+04	9.00E+00	9.00E+00	9.00E+00	5.10E+04	5.64E+04	4.52E+04	9.00E+00	9.00E+00	9.00E+00
961	LysoPC 18:4	Others	4.40E+03	4.40E+03	4.40E+03	9.00E+00	9.00E+00	9.00E+00	1.21E+04	1.06E+04	1.25E+04	9.00E+00	9.00E+00	9.00E+00
962	Indole 3-acetic acid (IAA)	Others	2.42E+03	2.42E+03	2.42E+03	9.00E+00	9.00E+00	9.00E+00	7.46E+03	8.43E+03	3.45E+03	9.00E+00	9.00E+00	9.00E+00
963	Octadecanamide	Others	1.14E+05	7.88E+04	9.11E+04	6.98E+04	1.22E+05	8.16E+04	6.05E+04	1.05E+05	8.82E+04	9.28E+04	7.87E+04	1.00E+05
964	Nicotinic acid	Others	1.26E+04	1.51E+04	1.78E+04	1.25E+04	1.94E+04	2.04E+04	7.41E+03	1.07E+04	3.29E+04	2.05E+04	1.81E+04	1.31E+04
965	Lauric acid	Others	4.77E+03	4.84E+03	4.85E+03	4.68E+03	4.58E+03	5.31E+03	4.60E+03	5.30E+03	4.68E+03	4.48E+03	5.07E+03	4.78E+03
966	Oleic acid	Others	1.69E+06	1.21E+06	1.52E+06	1.41E+06	1.65E+06	1.41E+06	1.25E+06	1.68E+06	1.74E+06	1.28E+06	1.34E+06	1.79E+06
967	N,N-Dimethylaniline	Others	2.37E+04	2.02E+04	2.62E+04	3.67E+04	2.55E+04	2.18E+04	2.17E+04	3.22E+04	2.63E+04	3.23E+04	2.25E+04	2.77E+04
968	LysoPC 20:2	Others	6.20E+04	6.15E+04	6.25E+04	6.64E+04	7.90E+04	7.11E+04	5.02E+04	3.96E+04	4.84E+04	6.71E+04	7.01E+04	7.48E+04
969	Panaxynol	Others	1.45E+04	8.82E+03	1.27E+04	1.48E+04	1.27E+04	1.05E+04	6.57E+03	1.80E+04	1.67E+04	9.07E+03	9.03E+03	1.91E+04
970	(13E)-11a-Hydroxy-9,15-dioxoprost-13-enoic acid	Others	1.14E+04	9.13E+03	4.20E+03	1.35E+04	3.48E+03	2.05E+03	4.75E+03	4.14E+03	6.87E+02	6.25E+03	4.70E+03	7.57E+03
971	Pimelic acid	Others	1.42E+05	1.44E+05	1.49E+05	8.65E+04	1.09E+05	1.49E+05	2.02E+05	2.22E+05	1.78E+05	1.16E+05	1.20E+05	9.76E+04
972	6beta-Hydroxystigmast-4-en-3-one	Others	8.30E+04	4.02E+04	8.02E+03	8.97E+03	9.43E+03	4.49E+03	8.13E+03	1.06E+04	9.37E+03	9.86E+03	3.50E+03	8.76E+03
973	N-Acetyl-L-glutamate 5-semialdehyde	Others	4.42E+03	3.96E+03	4.31E+03	2.55E+03	1.83E+03	7.43E+03	4.32E+03	5.62E+03	3.92E+03	3.08E+03	5.37E+03	2.89E+03
974	Diethyl phosphate	Others	1.68E+05	1.69E+05	1.72E+05	1.49E+05	1.88E+05	1.78E+05	1.58E+05	1.65E+05	1.94E+05	1.71E+05	1.52E+05	1.70E+05
975	3-Hydroxypicolinic acid	Others	2.69E+03	6.43E+03	3.67E+03	2.36E+03	4.87E+03	3.44E+03	3.43E+03	5.20E+03	2.23E+03	3.94E+03	2.35E+03	3.91E+03
976	4-Nitrophenol	Others	1.67E+07	1.68E+07	1.67E+07	1.30E+07	1.30E+07	1.28E+07	2.31E+07	2.36E+07	2.37E+07	1.25E+07	1.23E+07	1.22E+07
977	Sarsasapogenin	Others	4.55E+03	6.81E+03	5.26E+03	3.72E+03	1.07E+04	2.67E+03	3.00E+03	3.01E+03	6.51E+03	6.06E+03	3.89E+03	6.22E+03
978	LysoPC 17:1	Others	2.60E+04	2.58E+04	2.52E+04	2.18E+04	2.26E+04	1.51E+04	4.55E+04	3.88E+04	2.32E+04	1.90E+04	2.00E+04	1.72E+04
979	LysoPC 19:0	Others	1.13E+04	1.17E+04	1.15E+04	8.59E+03	8.63E+03	1.01E+04	1.68E+04	1.41E+04	1.68E+04	9.63E+03	9.10E+03	7.03E+03
980	indolin-2-one	Others	9.94E+03	7.51E+03	9.88E+03	1.22E+04	9.89E+03	1.14E+04	8.59E+03	9.32E+03	8.25E+03	7.69E+03	1.02E+04	1.38E+04
981	N1,N8-Bis(sinapoyl)spermidine	Others	4.66E+04	4.78E+04	4.72E+04	3.43E+04	3.42E+04	3.94E+04	6.39E+04	7.16E+04	6.71E+04	3.31E+04	3.91E+04	2.97E+04
982	Stearic Acid	Others	2.39E+04	1.60E+04	1.77E+04	2.02E+04	1.72E+04	1.80E+04	1.21E+04	2.42E+04	1.80E+04	2.29E+04	9.37E+03	1.99E+04

983	Enoxacin	Others	1.60E+04	1.51E+04	2.16E+04	1.42E+04	2.41E+04	2.16E+04	1.86E+04	2.49E+04	2.77E+04	2.02E+04	1.47E+04	2.11E+04
984	Boldione	Others	7.15E+03	2.51E+03	2.71E+03	3.40E+03	5.24E+03	2.13E+03	7.07E+02	1.92E+03	1.69E+03	1.50E+03	5.36E+03	3.17E+03
985	Glutaric acid	Others	3.10E+06	3.11E+06	3.09E+06	1.11E+06	9.98E+05	1.07E+06	6.73E+06	6.79E+06	6.18E+06	9.90E+05	1.02E+06	9.49E+05
986	Palmitic acid	Others	1.97E+05	9.56E+04	1.62E+05	1.02E+05	1.70E+05	1.88E+05	9.84E+04	1.77E+05	2.35E+05	1.14E+05	1.06E+05	2.07E+05
987	Methylimidazole acetaldehyde	Others	8.93E+03	6.51E+03	3.81E+03	3.67E+03	7.44E+03	1.17E+03	2.19E+03	3.81E+03	4.49E+03	5.33E+03	4.43E+03	1.61E+03
988	Methylmalonic acid	Others	3.57E+07	3.59E+07	3.56E+07	2.15E+07	2.00E+07	2.10E+07	6.20E+07	6.10E+07	6.32E+07	1.94E+07	1.93E+07	1.88E+07
989	Amabiline	Others	8.57E+04	7.76E+04	8.19E+04	6.14E+04	1.13E+05	7.36E+04	6.90E+04	7.85E+04	9.23E+04	7.53E+04	5.21E+04	1.01E+05
990	Methylenesuccinic acid	Others	1.17E+05	1.18E+05	1.18E+05	1.06E+05	1.28E+05	1.13E+05	1.44E+05	1.13E+05	1.33E+05	1.03E+05	1.05E+05	1.09E+05
991	Maltol	Others	6.66E+05	6.43E+05	6.36E+05	8.03E+05	7.03E+05	5.64E+05	8.28E+05	5.42E+05	5.64E+05	5.90E+05	5.50E+05	7.50E+05
992	D-Galactaric acid	Others	9.05E+05	9.07E+05	9.04E+05	8.64E+05	9.21E+05	8.55E+05	1.08E+06	1.02E+06	9.46E+05	7.87E+05	7.85E+05	8.35E+05
993	Corticosterone	Others	2.48E+03	5.50E+03	7.93E+03	4.95E+03	4.24E+03	1.74E+04	3.80E+03	6.33E+03	7.72E+03	1.03E+04	3.59E+03	1.01E+04
994	Avermectin B2b aglycone	Others	1.58E+03	3.25E+03	2.42E+03	2.41E+03	3.12E+03	1.01E+03	3.53E+03	1.83E+03	3.94E+03	8.30E+02	3.11E+03	1.97E+03
995	Atorvastatin	Others	1.88E+03	4.28E+02	1.17E+03	2.85E+03	6.44E+02	6.37E+02	8.59E+02	1.07E+03	2.41E+03	1.00E+01	1.50E+03	2.22E+03
996	Succinic acid	Others	3.57E+07	3.60E+07	3.56E+07	2.17E+07	2.13E+07	2.11E+07	6.28E+07	6.07E+07	6.15E+07	1.94E+07	1.94E+07	1.86E+07
997	Ethylmalonic acid	Others	1.13E+05	1.13E+05	1.12E+05	4.91E+04	4.26E+04	3.89E+04	2.35E+05	2.40E+05	2.23E+05	3.80E+04	4.07E+04	3.82E+04
998	Palmitaldehyde	Others	3.26E+06	3.36E+06	3.23E+06	4.14E+06	2.65E+06	3.89E+06	3.81E+06	4.11E+06	1.86E+06	3.04E+06	3.35E+06	3.12E+06
999	Aminomalonic acid	Others	1.23E+06	1.24E+06	1.23E+06	6.99E+05	7.08E+05	7.03E+05	2.15E+06	2.22E+06	2.19E+06	6.50E+05	6.15E+05	6.11E+05
1000	LysoPC 18:0	Others	8.95E+04	9.02E+04	8.97E+04	6.78E+04	7.40E+04	6.91E+04	1.38E+05	1.44E+05	1.05E+05	6.03E+04	6.34E+04	6.34E+04
1001	O-Acetylethanolamine	Others	3.99E+06	4.23E+06	4.32E+06	4.18E+06	4.92E+06	4.98E+06	4.31E+06	4.08E+06	3.79E+06	3.66E+06	4.43E+06	4.39E+06
1002	Leucodopachrome	Others	1.14E+04	7.79E+03	1.24E+04	1.74E+04	9.59E+03	1.73E+04	7.07E+03	1.36E+04	1.22E+04	8.75E+03	1.78E+04	1.25E+04
1003	1-Naphthylacetic acid	Others	7.28E+03	4.03E+03	6.94E+03	5.10E+03	8.57E+03	4.89E+03	9.43E+03	8.75E+03	7.48E+03	3.46E+03	5.56E+03	7.35E+03
1004	Ergosterol peroxide	Others	6.57E+04	1.68E+04	8.97E+03	1.16E+04	1.06E+04	4.30E+03	7.88E+03	2.97E+03	2.26E+04	1.20E+04	4.55E+03	6.89E+03
1005	D-Saccharic acid	Others	9.29E+05	9.38E+05	9.19E+05	9.48E+05	9.19E+05	8.69E+05	1.05E+06	1.08E+06	1.03E+06	7.94E+05	8.11E+05	8.00E+05
1006	6-Hydroxyhexanoic acid	Others	3.68E+05	3.80E+05	3.72E+05	3.38E+05	3.20E+05	3.72E+05	4.81E+05	4.69E+05	4.31E+05	3.38E+05	2.89E+05	2.77E+05
1007	Phyllalbine	Others	2.44E+05	1.88E+05	1.57E+05	7.88E+04	1.95E+05	3.05E+05	1.23E+05	1.26E+05	1.00E+01	2.71E+05	1.00E+01	2.35E+05
1008	Methylecgonine	Others	3.86E+03	1.50E+03	2.65E+03	1.78E+03	3.06E+03	3.76E+03	1.76E+03	7.48E+02	4.39E+03	2.24E+03	1.59E+03	3.67E+03

1009	4-Guanidinobutyric acid	Others	1.17E+06	1.19E+06	1.16E+06	1.45E+06	1.34E+06	1.40E+06	1.01E+06	9.05E+05	9.65E+05	1.25E+06	1.21E+06	1.19E+06
1010	Riddelline	Others	6.34E+03	1.27E+04	5.23E+03	1.32E+04	4.85E+03	4.07E+03	3.24E+03	4.77E+03	5.74E+03	9.32E+03	4.13E+03	5.73E+03
1011	Riboflavin	Others	3.68E+05	3.71E+05	3.69E+05	9.41E+04	9.92E+04	1.03E+05	7.94E+05	8.11E+05	8.94E+05	8.22E+04	9.45E+04	7.88E+04
1012	L-Gulono-1,4-Lactone	Others	8.76E+04	8.85E+04	8.65E+04	5.55E+04	4.11E+04	4.64E+04	1.51E+05	1.61E+05	1.70E+05	3.79E+04	4.57E+04	3.94E+04
1013	N-Acetylputrescine	Others	4.88E+04	4.84E+04	4.76E+04	2.93E+04	2.25E+04	2.03E+04	9.79E+04	9.15E+04	8.70E+04	1.87E+04	1.97E+04	2.36E+04
1014	(S)-2-Aceto-2-hydroxybutanoate	Others	7.63E+04	5.46E+04	1.57E+05	8.54E+04	3.03E+05	1.46E+05	1.82E+05	9.81E+04	7.03E+04	3.16E+05	9.28E+04	4.85E+04
1015	Hexadecylsphingosine	Others	2.24E+06	2.26E+06	2.26E+06	2.33E+06	2.66E+06	2.49E+06	2.45E+06	2.02E+06	2.06E+06	1.86E+06	2.24E+06	2.29E+06
1016	Bilirubin	Others	1.82E+04	1.00E+01	1.97E+03	7.32E+03	1.65E+03	1.00E+01	1.00E+01	2.97E+03	3.43E+03	7.47E+02	2.25E+03	4.67E+03
1017	Oxymorphone	Others	1.01E+05	9.72E+04	7.33E+04	9.21E+04	8.76E+04	8.15E+04	9.04E+04	4.84E+04	5.59E+04	6.93E+04	6.94E+04	8.41E+04
1018	alpha-Tocopherol	Others	1.87E+06	2.77E+05	2.36E+04	2.31E+04	2.96E+04	2.42E+04	2.36E+04	2.33E+04	2.33E+04	1.73E+04	2.85E+04	1.92E+04
1019	2-Oxoadipic acid	Others	1.48E+04	1.49E+04	1.50E+04	2.94E+03	2.34E+03	4.57E+03	2.54E+04	3.87E+04	4.03E+04	3.59E+03	1.71E+03	3.01E+03
1020	Sinalbin	Others	6.87E+03	1.17E+04	6.87E+03	6.84E+03	7.20E+03	7.90E+03	6.22E+03	7.50E+03	7.72E+03	7.58E+03	7.25E+03	3.58E+03
1021	3-Dehydroquinate	Others	4.06E+03	7.52E+03	9.26E+03	4.24E+03	1.21E+04	1.38E+04	5.62E+03	9.35E+03	8.07E+03	4.57E+03	1.10E+04	9.64E+03
1022	Glyceric acid	Others	1.15E+05	6.51E+04	1.58E+05	1.02E+05	3.55E+05	1.09E+05	1.87E+05	7.85E+04	6.58E+04	2.31E+05	1.53E+05	8.53E+04
1023	4-Hydroxy-2-oxoglutaric acid	Others	2.00E+05	2.02E+05	1.97E+05	2.08E+05	1.86E+05	1.87E+05	2.11E+05	2.38E+05	2.72E+05	1.55E+05	1.62E+05	1.64E+05
1024	3-Dehydro-L-Threonic Acid	Others	1.01E+07	1.04E+07	1.02E+07	1.33E+07	1.38E+07	1.37E+07	7.02E+06	6.74E+06	6.46E+06	1.02E+07	1.18E+07	1.16E+07
1025	Pyrrole-2-carboxylic acid	Others	2.27E+03	2.92E+03	3.35E+03	2.70E+03	6.26E+03	2.03E+03	2.95E+03	4.39E+03	2.14E+03	1.48E+03	4.14E+03	3.45E+03
1026	L-(-)-Malic acid	Others	3.27E+05	3.33E+05	3.26E+05	4.52E+05	4.47E+05	4.46E+05	2.16E+05	2.17E+05	1.79E+05	3.10E+05	3.94E+05	3.97E+05
1027	Ruscogenin	Others	8.48E+03	4.80E+03	7.36E+03	6.57E+03	1.50E+04	3.70E+03	9.17E+03	8.04E+03	2.32E+03	1.59E+03	7.95E+03	1.11E+04
1028	Gluconic acid	Others	1.23E+07	1.23E+07	1.22E+07	7.05E+06	6.66E+06	6.47E+06	2.20E+07	2.40E+07	2.18E+07	5.17E+06	5.53E+06	5.81E+06
1029	Skimmianin	Others	4.36E+03	4.47E+03	2.83E+03	3.50E+03	3.29E+03	3.63E+03	3.58E+03	1.95E+03	1.77E+03	1.71E+03	4.04E+03	2.66E+03
1030	cis-2-Methylaconitate	Others	2.08E+03	2.22E+03	3.43E+03	3.64E+03	1.82E+03	6.17E+03	3.30E+03	4.63E+03	2.20E+03	2.24E+03	4.27E+03	2.79E+03
1031	Pumiliotoxin A	Others	1.21E+04	9.24E+03	1.44E+04	1.35E+04	1.88E+04	2.15E+04	1.11E+04	1.16E+04	9.25E+03	1.62E+04	1.55E+04	1.12E+04
1032	L-Nicotine	Others	8.13E+03	4.30E+03	4.70E+03	6.58E+03	4.79E+03	7.52E+03	3.97E+03	3.00E+03	3.24E+03	5.74E+03	5.69E+03	3.63E+03
1033	(2S,3R,4E)-2-Amino-4-heptadecene-1,3-diol	Others	4.74E+04	5.50E+04	3.02E+04	4.37E+04	4.29E+04	3.32E+04	5.86E+04	6.93E+03	4.94E+03	4.14E+04	4.06E+04	1.29E+04
1034	Succinic anhydride	Others	2.83E+05	2.91E+05	2.91E+05	1.25E+05	1.57E+05	1.83E+05	5.43E+05	5.32E+05	5.44E+05	1.01E+05	1.39E+05	1.27E+05

1035	17alpha-Estradiol	Others	5.57E+03	5.00E+03	5.04E+03	1.18E+04	5.38E+03	3.31E+03	6.07E+03	8.87E+03	6.13E+02	2.86E+03	8.11E+03	5.10E+03
1036	Betaine	Others	8.43E+06	8.45E+06	8.41E+06	1.47E+06	1.44E+06	1.31E+06	2.02E+07	1.90E+07	2.21E+07	1.09E+06	1.07E+06	1.15E+06
1037	Methyl jasmonate	Others	8.73E+03	1.24E+04	1.04E+04	1.93E+04	1.31E+04	9.70E+03	6.34E+03	8.79E+03	1.24E+04	4.89E+03	1.01E+04	1.75E+04
1038	5,6-Dihydroxyindole-5-O-β-glucoside	Others	3.21E+04	3.37E+04	3.37E+04	3.60E+04	5.88E+04	4.92E+04	1.81E+04	1.86E+04	1.51E+04	3.39E+04	3.96E+04	3.67E+04
1039	4-Methyl-5-thiazoleethanol	Others	3.31E+03	3.26E+03	3.03E+03	5.24E+03	4.66E+03	3.05E+03	1.71E+03	3.11E+03	1.86E+03	3.05E+03	3.43E+03	3.40E+03
1040	Norepinephrine	Others	6.49E+03	4.91E+03	5.24E+03	4.77E+03	6.23E+03	7.14E+03	4.92E+03	2.60E+03	7.26E+03	2.46E+03	6.13E+03	5.20E+03
1041	Agmatine	Others	5.08E+04	5.22E+04	5.17E+04	2.75E+04	2.53E+04	3.41E+04	1.01E+05	9.56E+04	9.13E+04	1.87E+04	2.39E+04	2.32E+04
1042	Juglone	Others	6.36E+03	1.21E+04	6.04E+03	5.63E+03	3.76E+03	8.10E+03	6.38E+03	6.22E+03	1.07E+04	3.41E+03	5.47E+03	4.30E+03
1043	1,2-Dihydroxy-6-methylcyclohexa-3,5-dienecarboxylate	Others	4.38E+03	2.33E+03	1.88E+03	1.88E+03	3.13E+03	1.34E+03	1.37E+03	3.15E+03	1.29E+03	2.19E+03	1.00E+03	1.57E+03
1044	5-Aminolevulinate	Others	7.12E+03	1.01E+04	7.79E+03	1.37E+04	2.58E+03	1.05E+04	1.13E+04	8.57E+03	9.36E+03	4.73E+03	8.32E+03	6.95E+03
1045	Pyridoxine-5'-O-glucoside	Others	5.07E+05	5.29E+05	5.13E+05	7.68E+05	7.57E+05	8.17E+05	2.25E+05	3.07E+05	2.48E+05	5.11E+05	5.97E+05	6.44E+05
1046	Anthranilate-1-O-Sophoroside	Others	2.44E+05	2.60E+05	2.51E+05	3.92E+05	4.43E+05	4.44E+05	5.41E+04	5.32E+04	6.96E+04	3.10E+05	3.16E+05	3.17E+05
1047	4-Aminoindole	Others	4.12E+05	4.19E+05	4.12E+05	2.10E+05	2.06E+05	2.09E+05	8.13E+05	8.33E+05	7.81E+05	1.52E+05	1.52E+05	1.50E+05
1048	N-Acetyl-D-mannosamine	Others	2.05E+05	2.22E+05	2.07E+05	3.02E+05	2.89E+05	3.19E+05	1.32E+05	1.24E+05	1.33E+05	2.55E+05	2.22E+05	1.82E+05
1049	3-Hydroxypropanoic acid	Others	1.16E+04	1.22E+04	1.21E+04	1.44E+04	1.24E+04	1.80E+04	1.15E+04	1.09E+04	1.21E+04	5.54E+03	1.24E+04	1.37E+04
1050	Talatisamine	Others	2.62E+04	8.77E+03	1.09E+04	2.41E+04	8.44E+03	1.37E+04	7.21E+03	1.31E+04	1.24E+04	7.15E+03	8.09E+03	1.70E+04
1051	L-Ascorbic acid	Others	4.24E+04	4.29E+04	4.21E+04	1.79E+04	1.83E+04	1.58E+04	7.23E+04	8.19E+04	1.12E+05	1.11E+04	1.39E+04	1.11E+04
1052	Physalin A	Others	7.79E+03	5.46E+03	1.33E+04	5.10E+03	2.50E+04	1.91E+04	1.18E+04	6.65E+03	1.02E+04	5.17E+03	1.87E+04	9.99E+03
1053	Prenylated FMNH2	Others	7.79E+03	1.83E+04	1.33E+04	5.10E+03	2.50E+04	1.91E+04	1.18E+04	6.65E+03	1.02E+04	5.17E+03	1.87E+04	9.99E+03
1054	Isorubrofusarin 10-gentiobioside	Others	4.14E+03	8.15E+03	2.78E+03	7.91E+03	1.04E+03	2.47E+03	3.05E+03	2.98E+03	4.91E+03	2.06E+03	1.56E+03	4.19E+03
1055	N-Acetyl-D-galactosamine	Others	1.37E+05	1.42E+05	1.35E+05	1.93E+05	1.61E+05	1.76E+05	1.34E+05	1.33E+05	1.14E+05	1.27E+05	9.75E+04	1.36E+05
1056	Mitraphylline	Others	1.16E+04	5.92E+03	9.59E+03	1.14E+04	1.35E+04	1.50E+04	2.80E+03	1.12E+04	7.07E+03	7.83E+03	9.44E+03	9.81E+03
1057	Mannitol	Others	9.57E+04	9.91E+04	9.46E+04	9.57E+04	8.66E+04	8.71E+04	1.25E+05	1.31E+05	1.48E+05	6.36E+04	5.72E+04	5.93E+04
1058	4-Hydroxybutanoic acid	Others	8.12E+03	6.85E+03	9.02E+03	6.00E+03	2.50E+04	2.66E+03	4.88E+03	7.96E+03	9.34E+03	1.34E+04	4.03E+03	4.90E+03
1059	Eucommiol	Others	4.17E+05	4.44E+05	4.17E+05	5.08E+05	3.95E+05	5.08E+05	5.10E+05	4.73E+05	5.12E+05	3.19E+05	3.27E+05	2.87E+05
1060	Sphingosine	Others	2.48E+04	1.99E+04	1.39E+04	2.45E+04	8.71E+03	1.86E+04	1.13E+04	2.79E+04	1.06E+04	9.84E+03	1.67E+04	7.52E+03

1061	Physalin L	Others	4.94E+03	7.38E+03	1.33E+04	1.31E+04	2.13E+04	1.72E+04	1.91E+04	2.98E+03	1.19E+04	1.11E+04	8.22E+03	1.47E+04
1062	Anchoic Acid	Others	4.94E+06	5.24E+06	4.90E+06	6.16E+06	4.71E+06	5.85E+06	5.90E+06	5.77E+06	6.19E+06	3.68E+06	3.64E+06	3.43E+06
1063	Ecgonine	Others	8.56E+02	2.00E+03	1.03E+03	3.69E+03	1.05E+03	1.20E+03	1.17E+03	2.93E+02	7.63E+02	1.56E+03	1.47E+03	7.57E+02
1064	Acetryptine	Others	6.64E+03	7.14E+03	6.65E+03	8.04E+03	6.38E+03	8.10E+03	7.61E+03	1.04E+04	6.45E+03	3.77E+03	6.45E+03	4.06E+03
1065	Indole-5-carboxylic acid	Others	2.31E+04	2.49E+04	2.30E+04	3.06E+04	2.52E+04	3.02E+04	2.60E+04	2.05E+04	2.80E+04	2.11E+04	1.73E+04	1.58E+04
1066	LysoPC 14:0	Others	2.99E+04	3.04E+04	2.93E+04	1.73E+04	1.27E+04	1.24E+04	6.26E+04	6.43E+04	5.58E+04	8.12E+03	1.01E+04	8.22E+03
1067	Sulforaphane	Others	3.15E+03	1.19E+04	1.40E+04	1.59E+04	1.53E+04	2.19E+04	1.60E+04	1.05E+04	1.53E+04	1.05E+04	1.51E+04	7.34E+03
1068	LysoPC 16:0	Others	4.76E+06	4.94E+06	4.71E+06	4.49E+06	4.65E+06	4.10E+06	7.06E+06	7.35E+06	6.35E+06	2.77E+06	2.76E+06	2.67E+06
1069	Solasodine	Others	1.00E+01	1.70E+04	3.62E+04	1.68E+04	3.30E+04	6.70E+04	6.29E+04	1.25E+04	4.23E+04	3.39E+04	3.83E+04	1.00E+01
1070	Histamine	Others	3.53E+05	3.79E+05	3.49E+05	6.02E+05	5.94E+05	5.69E+05	1.64E+05	1.89E+05	1.87E+05	3.35E+05	3.88E+05	3.66E+05
1071	5,6-DHET	Others	4.45E+03	5.72E+03	8.69E+03	3.15E+03	2.28E+04	1.16E+04	2.41E+03	4.99E+03	4.87E+03	3.51E+03	5.31E+03	1.41E+04
1072	1-Octacosanol	Others	6.23E+04	4.58E+04	8.06E+04	3.69E+04	2.35E+05	1.96E+04	5.40E+04	1.23E+05	3.59E+04	7.16E+04	3.82E+04	6.72E+04
1073	Rutacridone	Others	1.15E+04	2.93E+04	9.98E+03	1.61E+04	1.90E+04	5.97E+03	9.15E+03	1.13E+04	9.45E+03	9.33E+03	4.71E+03	1.09E+04
1074	LysoPE 14:0 (2n isomer)	Others	7.90E+03	8.28E+03	7.91E+03	7.51E+03	7.43E+03	7.62E+03	1.14E+04	1.39E+04	9.38E+03	3.81E+03	5.13E+03	4.60E+03
1075	Nicotianamine	Others	6.64E+04	7.14E+04	6.50E+04	1.11E+05	1.09E+05	9.96E+04	4.19E+04	3.96E+04	4.22E+04	7.40E+04	5.30E+04	6.02E+04
1076	Veratramine	Others	6.91E+03	1.55E+03	3.15E+03	5.15E+03	2.99E+03	4.47E+03	5.92E+03	1.00E+01	4.45E+03	1.49E+03	1.00E+01	5.84E+03
1077	Muconic acid	Others	1.18E+06	1.25E+06	1.17E+06	1.60E+06	1.70E+06	1.54E+06	1.09E+06	1.08E+06	1.16E+06	9.44E+05	9.02E+05	9.72E+05
1078	Indole-3-carboxylic acid	Others	8.97E+04	9.49E+04	8.82E+04	1.26E+05	1.21E+05	1.14E+05	8.63E+04	8.83E+04	8.93E+04	6.22E+04	7.30E+04	7.20E+04
1079	4-Pyridoxic acid-O-glucoside	Others	6.58E+03	7.74E+03	6.88E+03	1.45E+04	1.30E+04	1.69E+04	9.00E+00	9.00E+00	9.00E+00	7.02E+03	1.05E+04	7.62E+03
1080	g-Strophanthin	Others	5.39E+02	8.02E+03	4.29E+03	1.08E+03	6.18E+03	8.51E+03	1.63E+03	3.53E+03	5.52E+03	5.28E+02	5.07E+03	3.30E+03
1081	D-Sorbitol	Others	3.27E+05	3.45E+05	3.23E+05	3.42E+05	3.27E+05	3.12E+05	4.57E+05	4.50E+05	4.85E+05	1.93E+05	1.91E+05	1.70E+05
1082	Acetoxyacetic acid	Others	2.53E+04	2.70E+04	2.42E+04	3.69E+04	4.52E+04	2.85E+04	1.85E+04	2.05E+04	2.00E+04	2.98E+04	1.66E+04	1.46E+04
1083	LysoPC 18:0 (2n isomer)	Others	7.24E+04	7.40E+04	7.30E+04	1.70E+04	1.78E+04	2.18E+04	1.74E+05	2.01E+05	1.39E+05	1.05E+04	1.15E+04	9.03E+03
1084	Mefenamic acid	Others	1.80E+03	2.46E+03	3.43E+03	2.52E+03	7.17E+03	5.25E+03	1.31E+03	3.38E+03	2.22E+03	4.32E+03	2.37E+03	1.42E+03
1085	Homodihydrocapsaicin I	Others	1.47E+04	3.57E+04	1.98E+04	1.57E+04	4.72E+04	9.91E+03	1.79E+04	2.72E+04	1.68E+04	1.64E+04	1.39E+04	9.10E+03
1086	Androsterone	Others	7.30E+03	7.88E+03	4.18E+03	1.00E+01	4.90E+03	1.11E+04	5.36E+03	1.00E+01	3.63E+03	4.83E+03	3.64E+03	1.00E+01

1087	Etiocholanolone	Others	7.30E+03	7.88E+03	4.18E+03	1.00E+01	4.90E+03	1.11E+04	5.36E+03	1.00E+01	3.63E+03	4.83E+03	3.64E+03	1.00E+01
1088	Cyperotundone	Others	6.36E+03	1.03E+04	8.84E+03	1.63E+04	1.90E+04	6.74E+03	8.18E+03	1.22E+04	2.34E+03	7.51E+03	3.91E+03	1.08E+04
1089	Kynurenic acid	Others	8.92E+03	5.96E+03	4.26E+03	7.72E+03	3.69E+03	7.76E+03	4.66E+03	3.02E+03	4.84E+03	2.47E+03	1.86E+03	5.76E+03
1090	Chrysophanol	Others	3.30E+03	2.13E+03	2.09E+03	2.13E+03	4.59E+03	2.75E+03	1.31E+03	2.32E+02	2.91E+03	9.22E+02	1.95E+03	2.04E+03
1091	Beta-D-Glucose	Others	5.47E+03	2.71E+03	2.77E+03	3.03E+03	2.27E+03	5.33E+03	2.08E+03	2.15E+03	4.85E+03	1.63E+03	1.19E+03	2.66E+03
1092	LysoPC 17:0	Others	1.12E+04	1.18E+04	1.10E+04	1.29E+04	1.20E+04	1.12E+04	1.25E+04	1.94E+04	1.48E+04	4.63E+03	7.32E+03	6.18E+03
1093	5,6-Dihydroxyindole	Others	7.71E+03	6.62E+03	7.48E+03	5.46E+03	1.12E+04	1.46E+04	8.91E+03	6.01E+03	3.65E+03	6.05E+03	4.67E+03	4.79E+03
1094	D-(-)-Arabinose	Others	5.70E+05	6.27E+05	5.79E+05	8.07E+05	8.83E+05	8.80E+05	5.31E+05	5.24E+05	5.41E+05	4.20E+05	4.28E+05	4.25E+05
1095	LysoPE 20:2	Others	6.89E+03	7.26E+03	6.56E+03	1.18E+04	1.38E+04	9.21E+03	3.23E+03	4.21E+03	4.88E+03	5.19E+03	5.81E+03	6.19E+03
1096	1,10-Decanediol	Others	4.50E+04	4.88E+04	4.28E+04	9.33E+04	8.76E+04	7.65E+04	2.28E+04	2.89E+04	9.00E+00	3.97E+04	4.16E+04	4.56E+04
1097	2-Hydroxyhexadecanoic acid	Others	1.66E+06	1.78E+06	1.67E+06	1.75E+06	1.66E+06	1.76E+06	2.46E+06	2.54E+06	2.37E+06	8.59E+05	8.16E+05	8.58E+05
1098	Urocanic acid	Others	2.62E+04	1.08E+05	5.74E+04	2.68E+04	1.59E+05	1.73E+04	7.76E+04	1.37E+04	9.17E+04	1.12E+04	7.39E+04	1.44E+04
1099	1H-Indole-2,3-dione	Others	5.99E+03	4.97E+03	4.44E+03	2.82E+03	8.58E+03	8.68E+03	1.48E+03	2.55E+03	4.53E+03	4.00E+03	3.56E+03	2.15E+03
1100	LysoPC 15:1	Others	1.80E+04	1.93E+04	1.80E+04	2.20E+04	2.46E+04	2.24E+04	2.17E+04	2.17E+04	2.07E+04	1.11E+04	1.00E+04	1.21E+04
1101	Benzamide	Others	1.51E+05	1.55E+05	1.51E+05	5.66E+04	4.75E+04	5.37E+04	3.55E+05	3.74E+05	3.00E+05	2.42E+04	2.92E+04	2.05E+04
1102	4-Pyridoxic acid	Others	2.62E+04	2.91E+04	2.64E+04	4.01E+04	4.10E+04	4.19E+04	2.09E+04	2.67E+04	2.42E+04	1.94E+04	1.88E+04	1.85E+04
1103	D-Pinitol	Others	1.27E+05	1.35E+05	1.29E+05	1.10E+05	1.10E+05	1.23E+05	2.07E+05	2.18E+05	2.13E+05	5.05E+04	5.26E+04	5.47E+04
1104	Sodium Valproate	Others	1.26E+07	1.39E+07	1.26E+07	1.96E+07	1.91E+07	1.91E+07	1.18E+07	1.18E+07	1.25E+07	8.90E+06	8.50E+06	8.97E+06
1105	Phosphoric acid	Others	7.20E+05	7.58E+05	8.45E+05	5.25E+05	1.77E+06	1.55E+06	1.25E+05	7.74E+05	7.88E+05	5.38E+05	4.54E+05	7.57E+05
1106	3,5-Dihydroxy-2,4-dimethoxy-9H-fluoren-9-one	Others	2.54E+04	2.80E+04	2.59E+04	3.56E+04	3.92E+04	3.96E+04	2.60E+04	2.61E+04	2.52E+04	1.64E+04	1.56E+04	1.88E+04
1107	Pyridoxine	Others	1.24E+06	1.32E+06	1.24E+06	1.12E+06	1.10E+06	1.08E+06	2.06E+06	2.22E+06	1.99E+06	4.60E+05	4.98E+05	5.04E+05
1108	3-Hydroxybutyric acid	Others	5.44E+05	6.03E+05	5.33E+05	9.40E+05	9.40E+05	8.60E+05	4.34E+05	3.97E+05	4.31E+05	4.50E+05	3.70E+05	3.85E+05
1109	3-Hydroxypropyl palmitate glc-glucosamine	Others	2.46E+05	2.64E+05	2.34E+05	4.29E+05	4.22E+05	3.35E+05	1.89E+05	2.03E+05	2.06E+05	1.67E+05	1.59E+05	1.96E+05
1110	Phenethylamine	Others	5.31E+05	5.43E+05	5.31E+05	1.89E+05	1.94E+05	1.90E+05	1.16E+06	1.33E+06	1.12E+06	7.58E+04	8.24E+04	9.28E+04
1111	D-Threonic Acid	Others	9.54E+05	1.05E+06	9.57E+05	1.37E+06	1.42E+06	1.39E+06	1.05E+06	1.02E+06	1.01E+06	6.08E+05	5.57E+05	6.03E+05
1112	Phenylethanolamine	Others	2.93E+05	3.38E+05	2.91E+05	6.28E+05	6.00E+05	6.12E+05	1.27E+05	1.20E+05	1.11E+05	2.50E+05	2.53E+05	2.56E+05

1113	L-Tyramine	Others	6.56E+05	7.51E+05	6.50E+05	1.39E+06	1.49E+06	1.34E+06	2.28E+05	2.32E+05	2.32E+05	5.51E+05	5.54E+05	5.77E+05
1114	Dexamethasone	Others	5.49E+03	5.03E+03	9.20E+03	2.07E+04	1.14E+04	1.12E+04	3.60E+03	9.51E+03	2.07E+04	7.44E+03	7.56E+03	2.23E+03
1115	Securinine	Others	1.98E+03	1.34E+04	9.04E+03	2.05E+04	1.14E+04	1.16E+04	2.72E+04	2.33E+03	2.96E+03	1.04E+04	5.82E+03	6.44E+02
1116	Propyl 2-(trimethylammonio)ethyl phosphate	Others	1.35E+05	1.45E+05	1.31E+05	1.85E+05	1.89E+05	1.49E+05	1.76E+05	1.54E+05	1.77E+05	6.66E+04	6.31E+04	6.91E+04
1117	5-Hydroxymethylfurfural	Others	7.18E+05	8.28E+05	7.33E+05	1.22E+06	1.46E+06	1.33E+06	5.28E+05	4.71E+05	5.51E+05	5.43E+05	5.19E+05	4.55E+05
1118	LysoPE 16:0	Others	1.15E+06	1.27E+06	1.13E+06	1.72E+06	1.74E+06	1.52E+06	1.24E+06	1.45E+06	1.24E+06	6.12E+05	6.02E+05	6.09E+05
1119	Suberic Acid	Others	8.05E+04	9.20E+04	8.04E+04	1.38E+05	1.13E+05	1.37E+05	9.31E+04	7.75E+04	8.37E+04	4.81E+04	4.54E+04	4.49E+04
1120	Bufotaline	Others	1.43E+03	7.80E+03	4.31E+03	6.14E+03	1.02E+04	6.85E+03	1.00E+01	9.16E+03	1.00E+01	1.00E+01	4.66E+03	3.54E+03
1121	(-)-Jasmonoyl-L-Isoleucine	Others	4.27E+04	4.35E+04	4.28E+04	9.03E+03	8.54E+03	9.53E+03	1.08E+05	1.07E+05	9.95E+04	3.00E+03	3.19E+03	3.27E+03
1122	Anthraquinone	Others	7.98E+03	2.63E+04	6.30E+03	2.42E+04	1.63E+04	6.68E+03	2.30E+03	3.43E+03	5.23E+03	3.66E+03	4.53E+03	8.24E+03
1123	Alizarin 2-methyl ether	Others	2.39E+03	2.89E+03	2.01E+03	8.43E+02	1.93E+03	6.33E+03	6.63E+02	2.80E+03	1.25E+03	1.44E+03	1.68E+03	1.00E+01
1124	LysoPC 16:0 (2n isomer)	Others	2.00E+06	2.04E+06	2.00E+06	5.05E+05	5.60E+05	5.22E+05	4.75E+06	5.27E+06	4.35E+06	1.76E+05	1.71E+05	1.98E+05
1125	LysoPC 18:2	Others	2.16E+06	2.39E+06	2.11E+06	3.29E+06	3.41E+06	2.88E+06	2.53E+06	2.63E+06	2.23E+06	1.06E+06	1.04E+06	1.05E+06
1126	2-(Formylamino)benzoic acid	Others	1.95E+07	2.31E+07	2.05E+07	3.12E+07	2.91E+07	3.90E+07	2.20E+07	1.57E+07	2.55E+07	1.15E+07	1.11E+07	9.67E+06
1127	Erucic acid	Others	2.27E+06	1.99E+06	4.42E+06	2.67E+06	1.88E+07	6.45E+05	1.91E+06	4.48E+06	2.56E+06	2.18E+06	2.43E+06	2.40E+06
1128	LysoPC 18:1	Others	1.70E+06	1.90E+06	1.69E+06	2.47E+06	2.76E+06	2.45E+06	2.50E+06	1.69E+06	1.71E+06	7.74E+05	8.50E+05	8.00E+05
1129	α-Ketoglutaric acid	Others	5.20E+05	5.34E+05	5.22E+05	1.22E+05	1.35E+05	1.41E+05	1.22E+06	1.27E+06	1.30E+06	4.99E+04	4.26E+04	3.26E+04
1130	Tropine acetate	Others	6.23E+03	4.79E+03	5.36E+03	2.42E+03	8.08E+03	1.70E+04	2.30E+03	4.95E+03	2.01E+03	4.90E+02	5.72E+03	2.30E+03
1131	20-HETE	Others	3.48E+04	2.32E+04	4.84E+04	4.10E+04	2.10E+05	8.05E+03	2.75E+04	3.50E+04	2.70E+04	3.16E+04	1.95E+04	2.85E+04
1132	4-Nitrocatechol	Others	3.68E+03	3.13E+03	3.70E+03	2.35E+03	4.76E+03	9.74E+03	2.86E+03	3.38E+03	3.70E+03	2.06E+03	2.08E+03	1.01E+03
1133	2-Isopropylmalic Acid	Others	2.57E+06	2.68E+06	2.56E+06	1.34E+06	1.28E+06	1.28E+06	5.73E+06	5.60E+06	5.47E+06	4.13E+05	3.60E+05	3.90E+05
1134	Sarracine	Others	6.59E+06	7.46E+06	1.41E+07	8.63E+06	6.18E+07	2.07E+06	7.88E+06	9.58E+06	9.63E+06	7.18E+06	6.75E+06	7.68E+06
1135	3alpha,6beta-Ditigloyloxytropan-7beta-ol	Others	6.59E+06	7.46E+06	1.41E+07	8.63E+06	6.18E+07	2.07E+06	7.88E+06	9.58E+06	9.63E+06	7.18E+06	6.71E+06	7.68E+06
1136	Eicosapentaenoic acid	Others	1.17E+04	8.27E+03	1.09E+04	1.02E+04	3.41E+04	1.33E+04	8.71E+03	8.52E+03	5.49E+03	8.82E+03	3.41E+03	4.69E+03
1137	LysoPE 15:0	Others	1.86E+04	2.28E+04	1.91E+04	4.09E+04	5.03E+04	4.47E+04	7.85E+03	6.23E+03	5.93E+03	1.33E+04	1.33E+04	1.10E+04
1138	LysoPC 18:3	Others	1.30E+06	1.47E+06	1.27E+06	2.11E+06	2.16E+06	1.89E+06	1.53E+06	1.50E+06	1.45E+06	5.59E+05	5.52E+05	5.59E+05

1139	2-Aminoethanesulfonic acid	Others	1.00E+01	1.00E+01	1.33E+04	2.47E+04	2.70E+04	2.73E+04	1.32E+04	7.57E+03	1.09E+04	6.27E+03	5.99E+03	8.52E+03
1140	Estriol	Others	7.57E+03	5.99E+03	3.86E+03	1.86E+04	8.98E+02	1.03E+04	4.08E+03	1.84E+03	6.58E+03	3.77E+03	1.90E+03	1.43E+03
1141	Leukoaminochrome	Others	1.75E+04	1.22E+04	1.30E+04	9.62E+04	2.76E+04	2.00E+04	7.09E+03	7.50E+03	7.37E+03	1.18E+04	1.17E+04	1.07E+04
1142	5,7-Dihydroxychromone	Others	6.79E+04	7.50E+04	6.76E+04	7.54E+04	7.10E+04	7.30E+04	1.17E+05	1.13E+05	1.14E+05	2.00E+04	1.55E+04	1.63E+04
1143	Leukotriene A4	Others	4.63E+04	4.79E+04	3.31E+04	6.91E+04	1.19E+05	4.50E+04	9.36E+03	3.30E+03	3.33E+04	1.23E+04	1.85E+04	2.39E+04
1144	Denudatine	Others	3.76E+03	1.00E+01	4.99E+03	6.90E+03	1.76E+04	6.25E+03	3.46E+03	1.00E+01	5.59E+03	4.67E+03	1.00E+01	2.39E+03
1145	D-Glucose	Others	6.69E+06	8.10E+06	6.53E+06	1.63E+07	1.60E+07	1.50E+07	3.48E+06	3.39E+06	3.57E+06	3.52E+06	3.58E+06	3.69E+06
1146	LysoPE 14:0	Others	1.35E+04	1.68E+04	1.36E+04	3.24E+04	3.79E+04	3.33E+04	6.52E+03	5.46E+03	2.35E+03	7.70E+03	8.96E+03	6.71E+03
1147	Crotonoside	Others	1.08E+05	1.32E+05	8.63E+04	8.48E+05	2.24E+05	7.37E+04	4.28E+04	4.97E+04	4.19E+04	8.61E+04	8.59E+04	8.61E+04
1148	LysoPE 18:3	Others	1.04E+05	1.20E+05	9.87E+04	2.16E+05	2.22E+05	1.73E+05	9.96E+04	7.94E+04	7.98E+04	4.81E+04	3.87E+04	4.92E+04
1149	D-Fructose	Others	5.65E+06	6.83E+06	5.59E+06	1.30E+07	1.38E+07	1.26E+07	3.35E+06	3.29E+06	3.04E+06	2.82E+06	2.81E+06	3.09E+06
1150	(S)-10-Hydroxycamptothecin	Others	3.81E+03	5.63E+02	8.65E+02	1.33E+03	1.00E+01	2.53E+03	1.51E+03	1.51E+03	5.32E+02	1.00E+01	5.55E+02	2.68E+02
1151	Astaxanthin	Others	8.03E+03	7.23E+03	1.33E+04	2.41E+03	7.14E+04	3.83E+03	8.22E+03	3.07E+03	3.25E+03	5.91E+03	2.52E+03	8.03E+03
1152	LysoPE 18:2	Others	1.32E+06	1.42E+06	1.31E+06	1.25E+06	1.41E+06	1.15E+06	2.03E+06	2.36E+06	2.73E+06	2.31E+05	2.27E+05	3.27E+05
1153	8,9-DIHETRE	Others	1.63E+06	1.68E+06	2.92E+06	2.71E+06	1.50E+07	2.91E+05	7.43E+04	2.22E+06	2.10E+06	9.76E+04	1.67E+06	1.92E+06
1154	3,4'-Dihydroxy-3'-methoxybenzenepentanoic acid	Others	5.65E+04	6.95E+04	5.83E+04	1.13E+05	1.16E+05	1.27E+05	5.66E+04	4.64E+04	5.04E+04	1.74E+04	2.88E+04	2.35E+04
1155	LysoPE 16:0 (2n isomer)	Others	2.92E+05	3.32E+05	2.87E+05	4.31E+05	4.77E+05	3.94E+05	3.76E+05	4.21E+05	4.24E+05	6.16E+04	6.85E+04	7.77E+04
1156	D-Glucosamine	Others	5.57E+05	6.97E+05	5.47E+05	1.40E+06	1.41E+06	1.32E+06	3.42E+05	3.32E+05	3.22E+05	2.36E+05	2.14E+05	2.06E+05
1157	Methyl anthranilate	Others	2.13E+04	2.05E+04	9.30E+03	1.02E+05	6.59E+03	6.63E+03	3.03E+03	2.97E+03	4.11E+04	2.47E+03	7.66E+03	3.94E+03
1158	D-Galacturonic acid	Others	7.63E+06	7.99E+06	7.48E+06	4.44E+06	3.52E+06	3.30E+06	1.91E+07	1.68E+07	1.60E+07	3.65E+05	4.14E+05	3.79E+05
1159	D-Glucoronic acid	Others	9.18E+06	9.67E+06	9.14E+06	4.75E+06	4.94E+06	4.43E+06	2.21E+07	1.88E+07	2.14E+07	4.35E+05	5.07E+05	4.78E+05
1160	Sitostenone	Others	1.00E+06	1.25E+04	6.57E+04	5.64E+03	1.72E+04	4.14E+05	7.55E+03	2.27E+04	2.18E+04	2.67E+04	8.38E+03	7.86E+03
1161	Tetrahydroaldosterone-3-glucuronide	Others	4.36E+04	8.67E+04	6.52E+04	7.99E+04	1.65E+05	3.14E+05	2.30E+04	1.00E+01	1.00E+01	2.04E+04	1.00E+01	1.00E+01
1162	Soyacerebroside I	Others	1.00E+01	1.32E+04	1.19E+04	1.00E+01	9.41E+04	1.00E+01	1.00E+01	1.00E+01	1.00E+01	9.62E+02	1.00E+01	1.00E+01
1163	Clove chromone	Others	2.78E+03	3.96E+03	2.68E+03	1.02E+04	1.20E+04	9.46E+03	9.00E+00	9.00E+00	9.00E+00	9.00E+00	9.00E+00	9.00E+00
1164	LysoPE 15:0 (2n isomer)	Others	3.34E+03	4.48E+03	2.90E+03	1.27E+04	1.40E+04	9.12E+03	9.00E+00	9.00E+00	9.00E+00	9.00E+00	9.00E+00	9.00E+00

1165	LysoPC 15:0	Others	1.60E+04	1.80E+04	1.60E+04	1.59E+04	1.44E+04	1.58E+04	3.43E+04	3.80E+04	2.52E+04	9.00E+00	9.00E+00	9.00E+00
