# Supplementary material for: Altitudinal Variation of Metabolites, Mineral Elements and Antioxidant Activities of Rhodiola crenulata (Hook.f. & Thomson) H.Ohba
Source: Molecules. 2021 Dec 5;26(23):7383. doi: 10.3390/molecules26237383 (PMC8658832; doi:10.3390/molecules26237383)
Supplement: Supplementary file 1 [file molecules-26-07383-s001.zip › 20211124-V5-Table S2.pdf]

## Table S2: List of differential metabolites between RC-H vs. RC-L

| Number | Class | Compounds | Biomarkers | VIP | Fold change | P-value | Type |
|---|---|---|---|---|---|---|---|
| 1 | Quercetin and derivatives | Quercetin-3-O-rutinoside (Rutin) | Yes | 1.26E+00 | 5.08E+04 | 1.28E-06 | up |
| 2 | | 7-O-Methxyl Quercetin (Rhamnetin) | Yes | 1.26E+00 | 1.35E+04 | 1.49E-06 | up |
| 3 | | Quercetin-7-O-rutinoside | | 1.26E+00 | 8.73E+00 | 9.78E-05 | up |
| 4 | | Quercetin 3-beta-D-sophoroside | | 1.26E+00 | 5.11E+00 | 1.31E-05 | up |
| 5 | | Quercetin-3-O-(2''-O-galactosyl)glucoside | | 1.24E+00 | 4.89E+00 | 8.56E-04 | up |
| 6 | | Quercetin-3-O-glucoside (Isoquercitrin) | | 1.26E+00 | 3.70E+00 | 1.85E-05 | up |
| 7 | | Quercetin-3-O-sophoroside (Baimaside) | | 1.23E+00 | 3.22E+00 | 2.27E-03 | up |
| 8 | | Quercetin-3-O-glucosyl(1→4)rhamnoside-7-O-rutinoside | | 1.25E+00 | 2.55E+00 | 2.05E-04 | up |
| 9 | | Quercetin-3-O-(2''-O-Rhamnosyl)rutinoside | | 1.25E+00 | 2.28E+00 | 1.50E-04 | up |
| 10 | | Quercetin-3-O-(6''-O-arabinosyl)glucoside | | 1.14E+00 | 2.01E+00 | 1.74E-02 | up |
| 11 | | Dihydroquercetin (Taxifolin) | | 1.25E+00 | 3.42E-01 | 2.37E-04 | down |
| 12 | | Quercetin-3-O-(2''-O-glucosyl)glucuronide | | 1.26E+00 | 2.36E-01 | 1.50E-04 | down |
| 13 | Kaempferol and derivatives | Dihydrokaempferol-7-O-glucoside | | 1.26E+00 | 4.63E+00 | 1.62E-05 | up |
| 14 | | 6-C-MethylKaempferol-3-glucoside | | 1.26E+00 | 4.02E+00 | 4.40E-05 | up |
| 15 | | 7-Methylkaempferol (Rhamnocitrin) | | 1.26E+00 | 3.67E+00 | 1.53E-06 | up |
| 16 | | Dihydrokaempferol-3-O-glucoside | | 1.26E+00 | 3.57E+00 | 3.23E-06 | up |
| 17 | | 6-Hydroxykaempferol-3,6-O-Diglucoside | | 1.26E+00 | 3.57E+00 | 1.83E-05 | up |
| 18 | | Kaempferol-6,8-di-C-glucoside-7-O-glucoside | | 1.25E+00 | 2.13E+00 | 7.81E-04 | up |
| 19 | Catechin and derivatives | Procyanidin C1 | | 1.26E+00 | 9.02E+00 | 4.46E-05 | up |
| 20 | | Procyanidin C2 | | 1.26E+00 | 7.09E+00 | 6.38E-05 | up |
| 21 | | Procyanidin B1 | | 1.25E+00 | 6.75E+00 | 1.88E-04 | up |
| 22 | | Procyanidin B3 | | 1.26E+00 | 6.33E+00 | 3.57E-05 | up |
| 23 | | Procyanidin B2 | | 1.25E+00 | 4.73E+00 | 1.04E-04 | up |

| | | | | | | |
|---|---|---|---|---|---|---|
| 24 | | Catechin | 1.26E+00 | 3.92E+00 | 1.13E-06 | up |
| 25 | | (-)-Epicatechin-3-(3"-O-methyl)gallate | 1.24E+00 | 3.58E+00 | 4.67E-04 | up |
| 26 | Other flavonoids | Eriodictyol-3'-O-glucoside | 1.26E+00 | 5.05E+00 | 9.58E-06 | up |
| 27 | | Rhodioflavonoside | 1.26E+00 | 3.86E+00 | 2.35E-05 | up |
| 28 | | 6,7,8-Tetrahydroxy-5-methoxyflavone | 1.26E+00 | 3.81E+00 | 3.69E-06 | up |
| 29 | | 5,7,4'-Trihydroxy-6-methoxyflavone (Hispidulin) | 1.26E+00 | 3.71E+00 | 5.33E-06 | up |
| 30 | | Dihydrocharcone-4'-O-glucoside | 1.26E+00 | 3.63E+00 | 4.82E-06 | up |
| 31 | | Spiraeoside | 1.23E+00 | 3.18E+00 | 1.47E-03 | up |
| 32 | | Naringenin-7-O-glucoside (Prunin) | 1.26E+00 | 2.97E+00 | 1.33E-04 | up |
| 33 | | Aromadendrin-7-O-glucoside | 1.26E+00 | 2.26E+00 | 2.61E-05 | up |
| 34 | | Apigenin | 1.26E+00 | 3.92E-01 | 6.21E-05 | down |
| 35 | | Phloretin | 1.26E+00 | 3.58E-01 | 3.47E-06 | down |
| 36 | | Pinocembrin (Dihydrochrysin) | 1.26E+00 | 2.83E-01 | 3.60E-06 | down |
| 37 | | Eriodictyol-7-O-glucoside | 1.24E+00 | 1.61E-01 | 4.03E-04 | down |
| 38 | Gallic acid derivatives | 1,3-Trigallic acid | 1.26E+00 | 5.46E+00 | 1.97E-05 | up |
| 39 | | 3-Hydroxy-5-Methylphenol-1-O-(6'-Digalloyl)Glucoside | 1.26E+00 | 2.71E+00 | 6.46E-05 | up |
| 40 | | 3-O-Methylgallic Acid | 1.26E+00 | 2.48E+00 | 9.69E-06 | up |
| 41 | | 1,7-Di-O-galloyl-D-sedoheptulose | 1.26E+00 | 4.85E-01 | 1.36E-04 | down |
| 42 | | Methyl 6-O-galloyl-glucoside | 1.22E+00 | 3.57E-01 | 2.88E-03 | down |
| 43 | | Digallic Acid | 1.26E+00 | 3.54E-01 | 1.55E-05 | down |
| 44 | | 2-O-Salicyl-6-O-Galloyl-D-Glucose | 1.25E+00 | 3.32E-01 | 2.72E-04 | down |
| 45 | | Monogalloyl-diglucose | 1.26E+00 | 2.22E-01 | 4.60E-06 | down |
| 46 | | Ellagic acid | 1.26E+00 | 1.75E-01 | 2.45E-06 | down |
| 47 | Cinnamic acid-Coumaroyl and derivatives (direct) | 4-Hydroxycinnamyl alcohol 4-D-glucoside | 1.24E+00 | 6.70E+00 | 1.16E-03 | up |
| 48 | | 7-Methoxy-5-Prenyloxycoumarin | 1.25E+00 | 4.75E+00 | 2.02E-04 | up |
| 49 | | 3-O-p-Coumaroylquinic acid O-glucoside | 1.22E+00 | 3.48E+00 | 2.57E-03 | up |

| | | | | | | | |
|---|---|---|---|---|---|---|---|
| 50 | | 1-O-[(E)-p-Coumaroyl]-D-glucose | | 1.26E+00 | 2.02E+00 | 7.01E-05 | up |
| 51 | | 3-Methyl-4,8-dihydroxy-3,4-dihydroisocoumarin | | 1.15E+00 | 4.79E-01 | 3.05E-02 | down |
| 52 | | p-Coumaryl alcohol | | 1.26E+00 | 3.86E-01 | 4.96E-07 | down |
| 53 | | 7-Hydroxy-5-methoxycoumarin (Scopoletin) | | 1.21E+00 | 3.55E-01 | 3.84E-03 | down |
| 54 | | 2-Hydroxycinnamic acid | | 1.25E+00 | 3.05E-01 | 5.00E-04 | down |
| 55 | | Cinnamic acid | | 1.26E+00 | 1.32E-01 | 4.44E-06 | down |
| 56 | | Rhododendrol | Yes | 1.26E+00 | 2.59E+04 | 9.91E-06 | up |
| 57 | | Caffeoylbenzoyltartaric acid | Yes | 1.26E+00 | 1.65E+03 | 1.88E-05 | up |
| 58 | | 1,2-O-Diferuloylglycerol | Yes | 1.24E+00 | 1.49E+03 | 1.11E-03 | up |
| 59 | | Chlorogenic acid methyl ester | Yes | 1.24E+00 | 8.69E+02 | 1.31E-03 | up |
| 60 | | Sinapoyl malate | Yes | 1.20E+00 | 5.66E+02 | 6.38E-03 | up |
| 61 | | Methyl caffeate | Yes | 1.23E+00 | 1.47E+02 | 1.31E-03 | up |
| 62 | Phenylpropanes (indirect | Isoimperatorin | | 1.26E+00 | 5.11E+00 | 3.00E-06 | up |
| 63 | Cinnamic acid-Coumaroyl | Osthole | | 1.26E+00 | 4.78E+00 | 1.92E-05 | up |
| 64 | and derivatives) | Syringoylcaffeoylquinic acid-D-glucose | | 1.21E+00 | 4.12E+00 | 6.62E-03 | up |
| 65 | | Sinapic acid | | 1.25E+00 | 3.25E+00 | 3.13E-04 | up |
| 66 | | 1-O-Glucosyl sinapate | | 1.26E+00 | 2.49E+00 | 8.25E-05 | up |
| 67 | | 6-O-Feruloyl-D-glucose | | 1.24E+00 | 4.65E-01 | 8.18E-04 | down |
| 68 | | Phenylpyruvic acid | | 1.23E+00 | 3.65E-01 | 2.00E-03 | down |
| 69 | | 3-(4-Hydroxyphenyl)-propionic acid | | 1.24E+00 | 2.41E-01 | 8.57E-04 | down |
| 70 | | Caffeoyl(p-Hydroxybenzoyl)tartaric acid | | 1.26E+00 | 2.16E-01 | 8.76E-05 | down |
| 71 | | 2,3,4,5,6-pentahydroxyhexyl 2-hydroxybenzoate | Yes | 1.25E+00 | 6.78E+03 | 5.40E-04 | up |
| 72 | Phenylmethanes (indirect | 4-Hydroxybenzyl Alcohol | Yes | 1.25E+00 | 4.98E+02 | 2.39E-04 | up |
| 73 | Cinnamic acid-Coumaroyl | Salicin | | 1.26E+00 | 5.53E+00 | 1.16E-04 | up |
| 74 | and derivatives) | 1-O-(3,4-Dihydroxy-5-methoxy-benzoyl)-glucoside | | 1.26E+00 | 4.53E+00 | 6.94E-07 | up |
| 75 | | Vanillic acid-4-O-glucoside | | 1.26E+00 | 3.50E+00 | 6.08E-06 | up |

| | | | | | | | |
|---|---|---|---|---|---|---|---|
| 76 | | Protocatechuic acid-4-O-glucoside | | 1.26E+00 | 3.03E+00 | 1.48E-06 | up |
| 77 | | 3,4,5-Trimethoxyphenyl-1-O-Glucoside | | 1.26E+00 | 2.30E+00 | 5.49E-06 | up |
| 78 | | 3,5-Dihydroxy-4-methoxybenzoic acid | | 1.25E+00 | 2.28E+00 | 2.23E-04 | up |
| 79 | | 2,5-Dihydroxybenzoic acid O-glucoside | | 1.26E+00 | 2.26E+00 | 3.21E-05 | up |
| 80 | | 1-O-Vanilloyl-D-Glucose | | 1.26E+00 | 2.22E+00 | 6.64E-06 | up |
| 81 | | 3-hydroxybenzaldehyde | | 1.20E+00 | 4.89E-01 | 6.13E-03 | down |
| 82 | | 4-Hydroxybenzoic acid | | 1.24E+00 | 4.73E-01 | 8.47E-04 | down |
| 83 | | Salicylic acid-2-O-glucoside | | 1.24E+00 | 3.93E-01 | 6.18E-04 | down |
| 84 | | Syringic acid | | 1.26E+00 | 3.87E-01 | 6.67E-05 | down |
| 85 | | 4-Hydroxybenzaldehyde | | 1.22E+00 | 3.14E-01 | 1.85E-03 | down |
| 86 | Phenylethanes (indirect Cinnamic acid-Coumaroyl and derivatives) | 4-Acetylphenyl-glucoside (Picein) | | 1.20E+00 | 3.93E-01 | 5.48E-03 | down |
| 87 | | Tyrosol | | 1.26E+00 | 2.95E-01 | 9.44E-06 | down |
| 88 | | 4-Hydroxyacetophenone | | 1.26E+00 | 1.79E-01 | 8.46E-05 | down |
| 89 | | p-Hydroxyphenyl acetic acid | | 1.26E+00 | 1.48E-01 | 1.48E-05 | down |
| 90 | | 3-Hydroxy-4-isopropylbenzylalcohol-3-O-glucoside | | 1.25E+00 | 1.40E-01 | 1.25E-04 | down |
| 91 | Shikimic acids | 3,5-Di-O-galloylshikimic acid | Yes | 1.21E+00 | 3.02E+03 | 4.34E-03 | up |
| 92 | | 3-Galloylshikimic acid | | 1.25E+00 | 4.29E+00 | 9.01E-05 | up |
| 93 | | Trans-5-O-(p-Coumaroyl)shikimate | | 1.24E+00 | 2.06E+00 | 1.60E-03 | up |
| 94 | Amino acids | (5-L-Glutamyl)-L-amino acid | Yes | 1.26E+00 | 1.37E+03 | 1.70E-05 | up |
| 95 | | Homoarginine | | 1.26E+00 | 8.46E+00 | 1.82E-05 | up |
| 96 | | L-Arginine | | 1.26E+00 | 7.29E+00 | 6.62E-06 | up |
| 97 | | N-α-Acetyl-L-ornithine | | 1.26E+00 | 7.27E+00 | 7.06E-07 | up |
| 98 | | L-Glutamine | | 1.26E+00 | 6.34E+00 | 1.91E-06 | up |
| 99 | | 5-Oxo-L-Proline | | 1.26E+00 | 4.93E+00 | 4.23E-05 | up |
| 100 | | L-Citrulline | | 1.26E+00 | 4.16E+00 | 4.06E-05 | up |
| 101 | | L-Ornithine | | 1.25E+00 | 2.87E+00 | 9.42E-05 | up |

| | | | | | | |
|---|---|---|---|---|---|---|
| 102 | L-Glutamic acid | | 1.26E+00 | 4.82E-01 | 1.14E-05 | down |
| 103 | Trans-4-Hydroxy-L-proline | | 1.24E+00 | 3.67E-01 | 6.00E-04 | down |
| 104 | N-Acetyl-L-methionine | Yes | 1.26E+00 | 4.26E+03 | 8.02E-06 | up |
| 105 | L-Methionine | | 1.26E+00 | 5.35E+01 | 2.06E-06 | up |
| 106 | L-Isoleucyl-L-Aspartate | | 1.26E+00 | 9.85E+00 | 4.11E-05 | up |
| 107 | L-Lysine | | 1.26E+00 | 6.23E+00 | 1.83E-05 | up |
| 108 | L-Isoleucine | | 1.25E+00 | 5.46E+00 | 1.63E-04 | up |
| 109 | L-Glycyl-L-isoleucine | | 1.26E+00 | 4.84E+00 | 5.56E-06 | up |
| 110 | L-Aspartic Acid | | 1.26E+00 | 3.65E+00 | 1.95E-06 | up |
| 111 | L-Aspartic acid-O-diglucoside | | 1.24E+00 | 3.21E+00 | 3.98E-04 | up |
| 112 | L-Asparagine | | 1.24E+00 | 2.77E+00 | 4.16E-04 | up |
| 113 | Pipecolic acid | | 1.26E+00 | 2.73E+00 | 1.66E-05 | up |
| 114 | N-Acetyl-L-Aspartic Acid | | 1.26E+00 | 2.64E+00 | 1.30E-04 | up |
| 115 | L-Aspartyl-L-Phenylalanine | | 1.26E+00 | 6.94E+00 | 6.84E-06 | up |
| 116 | L-Phenylalanine | | 1.26E+00 | 5.98E+00 | 9.77E-06 | up |
| 117 | L-Glycyl-L-phenylalanine | | 1.26E+00 | 5.92E+00 | 2.80E-05 | up |
| 118 | L-Tryptophan | | 1.26E+00 | 5.60E+00 | 2.68E-07 | up |
| 119 | 5-Hydroxy-L-tryptophan | | 1.26E+00 | 4.44E+00 | 1.96E-06 | up |
| 120 | L-Tyrosine | | 1.26E+00 | 3.05E+00 | 5.04E-05 | up |
| 121 | N-Acetyl-L-Tryptophan | | 1.26E+00 | 3.00E+00 | 9.95E-06 | up |
| 122 | L-Prolyl-L-Phenylalanine | | 1.20E+00 | 4.96E-01 | 8.49E-03 | down |
| 123 | S-(5'-Adenosy)-L-homocysteine | Yes | 1.26E+00 | 3.84E+03 | 4.82E-05 | up |
| 124 | Oxiglutatione | | 1.26E+00 | 6.44E+00 | 1.29E-04 | up |
| 125 | S-(Methyl)glutathione | | 1.23E+00 | 3.47E+00 | 9.67E-04 | up |
| 126 | N,N-Dimethylglycine | | 1.26E+00 | 2.59E+00 | 7.74E-08 | up |
| 127 | L-Cystine | | 1.24E+00 | 1.55E-01 | 8.29E-04 | down |

| | | | | | | | |
|---|---|---|---|---|---|---|---|
| 128 | | N-(3-Indolylacetyl)-L-alanine | Yes | 1.25E+00 | 3.65E+02 | 1.60E-04 | up |
| 129 | | L-Prolyl-L-Leucine | | 1.26E+00 | 1.76E+01 | 6.34E-07 | up |
| 130 | | L-Leucine | | 1.26E+00 | 5.34E+00 | 2.96E-05 | up |
| 131 | | L-Valine | | 1.26E+00 | 4.85E+00 | 4.83E-07 | up |
| 132 | | N-Glycyl-L-leucine | | 1.26E+00 | 4.76E+00 | 2.88E-05 | up |
| 133 | | Cycloleucine | | 1.26E+00 | 4.71E+00 | 2.04E-07 | up |
| 134 | | L-Histidine | | 1.26E+00 | 3.59E+00 | 6.82E-05 | up |
| 135 | | 4-Aminobutyric acid | | 1.19E+00 | 8.04E+00 | 7.66E-03 | up |
| 136 | | 5-Aminovaleric acid | | 1.20E+00 | 2.69E+00 | 7.46E-03 | up |
| 137 | Free fatty acids and glycerides | 9,10-Epoxyoctadecanoic Acid | Yes | 1.26E+00 | 3.82E+02 | 5.14E-05 | up |
| 138 | | 9,10,13-Trihydroxy-11-Octadecenoic Acid | | 1.26E+00 | 4.99E+00 | 5.08E-07 | up |
| 139 | | 9-Hydroxy-12-oxo-15(Z)-octadecenoic acid | | 1.26E+00 | 3.40E+00 | 4.22E-07 | up |
| 140 | | 9,12,13-Trihydroxy-10,15-octadecadienoic acid | | 1.26E+00 | 3.26E+00 | 1.03E-06 | up |
| 141 | | 9-Hydroxy-13-oxo-10-octadecenoic Acid | | 1.26E+00 | 3.12E+00 | 4.12E-06 | up |
| 142 | | 12,13-Epoxy-9-Octadecenoic Acid | | 1.26E+00 | 2.69E+00 | 2.52E-05 | up |
| 143 | | 9-Octadecenamide (Oleamide) | | 1.22E+00 | 2.50E+00 | 4.86E-03 | up |
| 144 | | 13-KODE; (9Z,11E)-13-Oxooctadeca-9,11-dienoic acid | | 1.26E+00 | 2.26E+00 | 4.21E-06 | up |
| 145 | | 12,13-DHOME; (9Z)-12,13-Dihydroxyoctadec-9-enoic acid | | 1.26E+00 | 2.25E+00 | 1.11E-04 | up |
| 146 | | 9-Hydroxy-10,12,15-octadecatrienoic acid | | 1.26E+00 | 2.24E+00 | 2.16E-06 | up |
| 147 | | 13S-Hydroperoxy-9Z,11E-octadecadienoic acid | | 1.26E+00 | 2.19E+00 | 2.44E-05 | up |
| 148 | | Punicic acid (9Z,11E,13Z-octadecatrienoic acid) | | 1.26E+00 | 2.15E+00 | 3.71E-05 | up |
| 149 | | 9-Oxo-10E,12Z-octadecadienoic acid | | 1.26E+00 | 2.15E+00 | 1.07E-06 | up |
| 150 | | 2-Linoleoylglycerol-1-O-glucoside | | 1.18E+00 | 4.99E-01 | 7.68E-03 | down |
| 151 | | 2-α-Linolenoyl-glycerol-1,3-di-O-glucoside | | 1.20E+00 | 4.78E-01 | 6.31E-03 | down |
| 152 | | 1-α-Linolenoyl-glycerol-3-O-glucoside | | 1.12E+00 | 4.69E-01 | 3.79E-02 | down |
| 153 | | 1-Linolenoyl-rac-glycerol-diglucoside | | 1.26E+00 | 4.61E-01 | 6.09E-05 | down |

| | | | | | | |
|---|---|---|---|---|---|---|
| 154 | | 1-Stearidonoyl-Glycerol | 1.26E+00 | 3.98E-01 | 2.17E-05 | down |
| 155 | | 2-α-Linolenoyl-glycerol-1-O-glucoside | 1.26E+00 | 3.22E-01 | 3.56E-05 | down |
| 156 | | 8,15-Dihydroxy-5,9,11,13-eicosatetraenoic acid | 1.26E+00 | 2.17E-01 | 1.40E-05 | down |
| 157 | Nucleotides | 1-Methyladenine | 1.26E+00 | 7.92E+00 | 5.27E-06 | up |
| 158 | | Adenosine 5'-monophosphate | 1.26E+00 | 7.27E+00 | 2.43E-05 | up |
| 159 | | 2-Deoxyribose-1-phosphate | 1.26E+00 | 5.89E+00 | 1.59E-06 | up |
| 160 | | 6-Methylmercaptopurine | 1.26E+00 | 5.59E+00 | 8.67E-08 | up |
| 161 | | Isopentenyladenine-7-N-glucoside | 1.24E+00 | 3.86E+00 | 8.73E-04 | up |
| 162 | | Adenosine 5'-diphosphate | 1.26E+00 | 3.34E+00 | 4.02E-05 | up |
| 163 | | 2'-Deoxycytidine | 1.25E+00 | 2.80E+00 | 2.48E-04 | up |
| 164 | | 5-Aminoimidazole ribonucleotide | 1.26E+00 | 2.67E+00 | 6.24E-05 | up |
| 165 | | Uridine 5'-monophosphate | 1.26E+00 | 2.51E+00 | 5.02E-05 | up |
| 166 | | Isoguanine | 1.26E+00 | 2.51E+00 | 1.26E-04 | up |
| 167 | | 5-Methylcytosine | 1.13E+00 | 2.23E+00 | 1.82E-02 | up |
| 168 | | Guanine | 1.26E+00 | 2.15E+00 | 2.56E-05 | up |
| 169 | | Xanthine | 1.16E+00 | 4.80E-01 | 1.13E-02 | down |
| 170 | | Cytidine | 1.26E+00 | 4.24E-01 | 5.08E-06 | down |
| 171 | | Adenine | 1.26E+00 | 4.08E-01 | 3.54E-06 | down |
| 172 | | Adenosine | 1.26E+00 | 3.32E-01 | 1.70E-05 | down |
| 173 | | Guanosine | 1.26E+00 | 2.88E-01 | 4.42E-06 | down |
| 174 | | 2-Aminopurine | 1.26E+00 | 2.72E-01 | 5.86E-05 | down |
| 175 | | N6-Isopentenyladenine | 1.23E+00 | 2.12E-01 | 1.07E-03 | down |
| 176 | Others | Deoxyelephantopin | 1.24E+00 | 8.10E+00 | 7.56E-04 | up |
| 177 | | Sterebin A | 1.13E+00 | 5.65E+00 | 1.85E-02 | up |
| 178 | | Diosbulbin B | 1.11E+00 | 3.85E+00 | 3.07E-02 | up |
| 179 | | Xanthatin | 1.23E+00 | 2.23E+00 | 1.87E-03 | up |

| | | | | | | |
|---|---|---|---|---|---|---|
| 180 | Curcolone | | 1.23E+00 | 2.23E+00 | 1.87E-03 | up |
| 181 | 2-(2-Hydroxy-2-propyl)-5-methyl-5-vinyltetrahydrofuran | | 1.16E+00 | 2.01E+00 | 1.75E-02 | up |
| 182 | N-Acetyl-D-glucosamine-1-phosphate | Yes | 1.26E+00 | 6.53E+03 | 5.40E-05 | up |
| 183 | Tryptamine | Yes | 1.26E+00 | 3.10E+03 | 4.16E-05 | up |
| 184 | Phthalic acid | Yes | 1.23E+00 | 1.04E+03 | 1.92E-03 | up |
| 185 | 4-Hydroxymandelonitrile | | 1.26E+00 | 5.09E+01 | 1.28E-06 | up |
| 186 | Solatriose | | 1.26E+00 | 2.78E+01 | 3.94E-07 | up |
| 187 | D-Fructose-1,6-biphosphate | | 1.25E+00 | 2.34E+01 | 1.04E-04 | up |
| 188 | Phosphoenolpyruvate | | 1.26E+00 | 1.26E+01 | 2.95E-05 | up |
| 189 | Indole-3-lactic acid | | 1.25E+00 | 1.12E+01 | 1.39E-04 | up |
| 190 | 2,2-Dimethylsuccinic acid | | 1.26E+00 | 1.04E+01 | 7.01E-06 | up |
| 191 | 6-Aminocaproic acid | | 1.25E+00 | 7.55E+00 | 2.71E-04 | up |
| 192 | LysoPC 20:4 | | 1.12E+00 | 7.39E+00 | 4.47E-02 | up |
| 193 | Glucose-1-phosphate | | 1.26E+00 | 7.39E+00 | 4.23E-05 | up |
| 194 | D-Glucose 6-phosphate | | 1.26E+00 | 7.34E+00 | 3.72E-05 | up |
| 195 | Methoxyindoleacetic acid | | 1.26E+00 | 6.84E+00 | 2.36E-06 | up |
| 196 | Piperidine | | 1.26E+00 | 6.25E+00 | 9.08E-06 | up |
| 197 | 6-Deoxyfagomine | | 1.26E+00 | 6.07E+00 | 9.02E-06 | up |
| 198 | N-Benzylmethylene isomethylamine | | 1.26E+00 | 5.78E+00 | 8.98E-06 | up |
| 199 | LysoPC 19:1 | | 1.23E+00 | 5.31E+00 | 1.48E-03 | up |
| 200 | Indole | | 1.25E+00 | 4.88E+00 | 3.96E-04 | up |
| 201 | LysoPE 17:1 (2n isomer) | | 1.23E+00 | 4.50E+00 | 1.36E-03 | up |
| 202 | 3-Indolepropionic acid | | 1.26E+00 | 4.42E+00 | 9.83E-05 | up |
| 203 | O-Phosphorylethanolamine | | 1.26E+00 | 4.33E+00 | 4.81E-07 | up |
| 204 | Benzoic acid, 3,4,5-trihydroxy-, (1R,5R,6R)-3-carboxy-5,6-dihydroxy-3-cyclohexen-1-yl ester | | 1.26E+00 | 4.31E+00 | 4.98E-05 | up |

| | | | | | |
|---|---|---|---|---|---|
| 205 | Turanose | 1.24E+00 | 4.09E+00 | 5.42E-04 | up |
| 206 | Fumaric acid | 1.26E+00 | 4.04E+00 | 6.11E-05 | up |
| 207 | Adipic Acid | 1.26E+00 | 3.99E+00 | 1.84E-05 | up |
| 208 | 2-Methylglutaric acid | 1.23E+00 | 3.46E+00 | 1.17E-03 | up |
| 209 | D-Pantothenic Acid | 1.26E+00 | 3.45E+00 | 6.72E-07 | up |
| 210 | Domesticoside | 1.26E+00 | 3.31E+00 | 2.01E-06 | up |
| 211 | Sarmentosin | 1.26E+00 | 3.31E+00 | 5.00E-05 | up |
| 212 | Rosiridoside B | 1.25E+00 | 2.83E+00 | 5.11E-04 | up |
| 213 | Argininosuccinic acid | 1.25E+00 | 2.66E+00 | 4.80E-04 | up |
| 214 | LysoPE 16:1 | 1.22E+00 | 2.48E+00 | 2.75E-03 | up |
| 215 | Zygadenine | 1.25E+00 | 2.35E+00 | 1.71E-04 | up |
| 216 | 6'-O-Glucosylaucubin | 1.20E+00 | 2.33E+00 | 7.08E-03 | up |
| 217 | D-Ribose | 1.25E+00 | 2.32E+00 | 2.67E-04 | up |
| 218 | LysoPE 16:1 (2n isomer) | 1.25E+00 | 2.32E+00 | 3.29E-04 | up |
| 219 | 2-Hydroxy-4-methylpentanoic acid | 1.20E+00 | 2.31E+00 | 4.70E-03 | up |
| 220 | D-Arabitol | 1.26E+00 | 2.16E+00 | 1.32E-04 | up |
| 221 | LysoPC 16:1 (2n isomer) | 1.26E+00 | 2.12E+00 | 2.07E-05 | up |
| 222 | Ribitol | 1.24E+00 | 2.04E+00 | 1.09E-03 | up |
| 223 | D-(-)-Arabinose | 1.26E+00 | 4.95E-01 | 6.47E-05 | down |
| 224 | LysoPE 20:2 | 1.19E+00 | 4.94E-01 | 1.23E-02 | down |
| 225 | 1,10-Decanediol | 1.23E+00 | 4.93E-01 | 1.17E-03 | down |
| 226 | 2-Hydroxyhexadecanoic acid | 1.26E+00 | 4.90E-01 | 1.36E-05 | down |
| 227 | LysoPC 15:1 | 1.25E+00 | 4.81E-01 | 2.90E-04 | down |
| 228 | Benzamide | 1.23E+00 | 4.68E-01 | 1.62E-03 | down |
| 229 | 4-Pyridoxic acid | 1.26E+00 | 4.61E-01 | 2.92E-06 | down |
| 230 | D-Pinitol | 1.25E+00 | 4.60E-01 | 1.33E-04 | down |

| | | | | | |
|---|---|---|---|---|---|
| 231 | Sodium Valproate | 1.26E+00 | 4.56E-01 | 1.50E-06 | down |
| 232 | 3,5-Dihydroxy-2,4-dimethoxy-9H-fluoren-9-one | 1.25E+00 | 4.45E-01 | 1.86E-04 | down |
| 233 | Pyridoxine | 1.26E+00 | 4.43E-01 | 4.40E-06 | down |
| 234 | 3-Hydroxybutyric acid | 1.25E+00 | 4.40E-01 | 1.49E-04 | down |
| 235 | 3-Hydroxypropyl palmitate glc-glucosamine | 1.23E+00 | 4.39E-01 | 2.38E-03 | down |
| 236 | Phenethylamine | 1.26E+00 | 4.38E-01 | 3.20E-05 | down |
| 237 | D-Threonic Acid | 1.26E+00 | 4.23E-01 | 3.18E-06 | down |
| 238 | Phenylethanolamine | 1.26E+00 | 4.13E-01 | 1.59E-06 | down |
| 239 | L-Tyramine | 1.26E+00 | 3.99E-01 | 4.31E-05 | down |
| 240 | Propyl 2-(trimethylammonio)ethyl phosphate | 1.24E+00 | 3.80E-01 | 1.12E-03 | down |
| 241 | 5-Hydroxymethylfurfural | 1.25E+00 | 3.78E-01 | 4.01E-04 | down |
| 242 | LysoPE 16:0 | 1.26E+00 | 3.66E-01 | 1.13E-04 | down |
| 243 | Suberic Acid | 1.25E+00 | 3.57E-01 | 4.86E-04 | down |
| 244 | (-)-Jasmonoyl-L-Isoleucine | 1.26E+00 | 3.49E-01 | 3.83E-05 | down |
| 245 | LysoPC 16:0 (2n isomer) | 1.26E+00 | 3.44E-01 | 4.51E-05 | down |
| 246 | LysoPC 18:2 | 1.25E+00 | 3.29E-01 | 1.76E-04 | down |
| 247 | 2-(Formylamino)benzoic acid | 1.23E+00 | 3.25E-01 | 1.84E-03 | down |
| 248 | LysoPC 18:1 | 1.26E+00 | 3.16E-01 | 6.93E-05 | down |
| 249 | α-Ketoglutaric acid | 1.25E+00 | 3.15E-01 | 2.91E-04 | down |
| 250 | 2-Isopropylmalic Acid | 1.26E+00 | 2.98E-01 | 3.75E-06 | down |
| 251 | LysoPE 15:0 | 1.25E+00 | 2.77E-01 | 3.25E-04 | down |
| 252 | LysoPC 18:3 | 1.26E+00 | 2.71E-01 | 6.02E-05 | down |
| 253 | 2-Aminoethanesulfonic acid | 1.26E+00 | 2.63E-01 | 7.59E-05 | down |
| 254 | 5,7-Dihydroxychromone | 1.26E+00 | 2.36E-01 | 8.16E-06 | down |
| 255 | D-Glucose | 1.26E+00 | 2.28E-01 | 6.55E-06 | down |
| 256 | LysoPE 14:0 | 1.26E+00 | 2.26E-01 | 1.28E-04 | down |

| | | | | | | |
|---|---|---|---|---|---|---|
| 257 | LysoPE 18:3 | | 1.25E+00 | 2.23E-01 | 5.47E-04 | down |
| 258 | D-Fructose | | 1.26E+00 | 2.22E-01 | 9.30E-06 | down |
| 259 | LysoPE 18:2 | | 1.25E+00 | 2.06E-01 | 2.48E-04 | down |
| 260 | 3,4'-Dihydroxy-3'-methoxybenzenepentanoic acid | | 1.26E+00 | 1.95E-01 | 6.38E-05 | down |
| 261 | LysoPE 16:0 (2n isomer) | | 1.26E+00 | 1.60E-01 | 1.17E-04 | down |
| 262 | D-Glucosamine | | 1.26E+00 | 1.59E-01 | 2.37E-06 | down |
| 263 | D-Galacturonic acid | | 1.24E+00 | 1.03E-01 | 6.49E-04 | down |
| 264 | D-Glucoronic acid | | 1.26E+00 | 1.01E-01 | 1.00E-05 | down |
| 265 | Clove chromone | Yes | 1.26E+00 | 8.54E-04 | 1.47E-04 | down |
| 266 | LysoPE 15:0 (2n isomer) | Yes | 1.24E+00 | 7.54E-04 | 1.23E-03 | down |
| 267 | LysoPC 15:0 | Yes | 1.26E+00 | 5.85E-04 | 5.51E-06 | down |
