# Supplementary material for: Altitudinal Variation of Metabolites, Mineral Elements and Antioxidant Activities of Rhodiola crenulata (Hook.f. & Thomson) H.Ohba
Source: Molecules. 2021 Dec 5;26(23):7383. doi: 10.3390/molecules26237383 (PMC8658832; doi:10.3390/molecules26237383)
Supplement: Supplementary file 1 [file molecules-26-07383-s001.zip › 20211124-V5-Table S3.pdf]

Table S3: List of differential metabolites between RC-M vs. RC-L

Number	Class	Compounds	Biomarkers	VIP	Fold change	P-value	Type
1	Quercetin and derivatives	7-O-Methxyl Quercetin (Rhamnetin)	Yes	1.25E+00	1.30E+05	4.13E-06	up
2		Quercetin-3-O-rutinoside (Rutin)	Yes	1.25E+00	6.02E+04	2.86E-09	up
3		5-O-Methylquercetin (Azaleatin)		1.19E+00	9.68E+00	4.87E-03	up
4		Isorhamnetin-3-O-rutinoside (Narcissin)		1.19E+00	9.58E+00	6.96E-03	up
5		Rhamnetin-3-O-Rutinoside		1.22E+00	9.55E+00	1.69E-03	up
6		Quercetin 3-beta-D-sophoroside		1.24E+00	6.93E+00	7.12E-05	up
7		Quercetin-3-O-glucoside (Isoquercitrin)		1.22E+00	6.11E+00	1.19E-03	up
8		Quercetin-7-O-rutinoside		1.16E+00	5.36E+00	8.48E-03	up
9		Quercetin 7-O-beta-D-glucoside		1.25E+00	4.59E+00	2.84E-07	up
10		Quercetin-3-O-sophoroside (Baimaside)		1.22E+00	3.42E+00	1.31E-03	up
11		Quercetin-3-O-(2"-O-Rhamnosyl)rutinoside		1.22E+00	3.21E+00	1.36E-03	up
12		Quercetin-3-O-galactoside (Hyperin)		1.16E+00	3.09E+00	8.88E-03	up
13		Quercetin		1.20E+00	2.91E+00	2.83E-03	up
14		Quercetin-3-O-(2"-O-galactosyl)glucoside		1.18E+00	2.85E+00	4.41E-03	up
15		Quercetin-3-O-rutinoside-7-O-glucoside		1.23E+00	2.58E+00	4.50E-04	up
16		Quercetin-3-O-glucosyl(1→4)rhamnoside-7-O-rutinoside		1.17E+00	2.21E+00	9.99E-03	up
17		Quercetin-3-O-xyloside (Reynoutrin)		1.15E+00	2.06E+00	2.37E-02	up
18		Quercetin-3-O-robinobioside		1.24E+00	4.23E-01	3.26E-04	down
19		Quercetin-3-O-(2"-O-glucosyl)glucuronide		1.24E+00	2.85E-01	1.92E-04	down
20		Quercetin-3-O-(6"-galloyl)galactoside	Yes	1.21E+00	1.10E-04	2.28E-03	down
21	Kaempferol and derivatives	Dihydrokaempferol-7-O-glucoside		1.24E+00	2.79E+01	3.19E-04	up
22		6-C-MethylKaempferol-3-glucoside		1.23E+00	1.73E+01	1.40E-03	up
23		Dihydrokaempferol-3-O-glucoside		1.25E+00	1.33E+01	9.83E-09	up

24		7-Methylkaempferol (Rhamnocitrin)	1.25E+00	5.46E+00	4.90E-06	up
25		Kaempferol-4'-O-glucoside	1.22E+00	3.22E+00	1.32E-03	up
26		6-Hydroxykaempferol-3,6-O-Diglucoside	1.24E+00	2.37E+00	1.60E-04	up
27		Kaempferol-3-O-galactoside (Trifolin)	1.12E+00	2.11E+00	1.77E-02	up
28		8-Prenylkaempferol	1.21E+00	3.49E-01	5.79E-03	down
29		Procyanidin B3	1.25E+00	6.12E+00	1.60E-07	up
30		Procyanidin B1	1.24E+00	2.86E+00	4.38E-05	up
31	Catechin and derivatives	(-)-Epicatechin-3-(3''-O-methyl)gallate	1.23E+00	2.24E+00	3.57E-04	up
32		Catechin	1.24E+00	2.11E+00	2.54E-04	up
33		Epicatechin	1.25E+00	4.95E-01	1.06E-05	down
34		Procyanidin C1 3'-O-gallate	1.23E+00	4.50E-01	1.04E-03	down
35		Luteolin-3'-O-glucoside	1.17E+00	3.27E+00	7.52E-03	up
36	Luteolin and derivatives	Luteolin-7-O-(2''-O-rhamnosyl)rutinoside	1.13E+00	2.35E+00	2.35E-02	up
37		5,7,3',4'-Tetrahydroxyflavone (Luteolin)	1.25E+00	2.23E-01	1.59E-07	down
38		5,7,3',4'-tetrahydroxyisoflavone (Isoluteolin)	1.25E+00	2.02E-01	4.04E-07	down
39		Eriodictyol-3'-O-glucoside	1.25E+00	2.62E+01	4.96E-06	up
40		2'-Hydoxy,5-methoxyGenistein-O-rhamnosyl-glucoside	1.17E+00	1.04E+01	8.34E-03	up
41		Sexangularetin-3-O-glucoside-7-O-rhamnoside	1.19E+00	9.92E+00	4.79E-03	up
42		Spiraeoside	1.25E+00	6.10E+00	1.45E-05	up
43		5,7,4'-Trihydroxy-6-methoxyflavone (Hispidulin)	1.25E+00	5.63E+00	4.13E-07	up
44	Other flavonoids	6,7,8-Tetrahydroxy-5-methoxyflavone	1.25E+00	5.57E+00	1.06E-06	up
45		Rhodiolgin	1.24E+00	3.82E+00	1.64E-04	up
46		Naringenin-7-O-glucoside (Prunin)	1.24E+00	3.80E+00	1.81E-04	up
47		Dihydrocharcone-4'-O-glucoside	1.24E+00	3.18E+00	3.72E-04	up
48		Sachaloside IV	1.24E+00	2.65E+00	3.75E-04	up
49		Rhodioflavonoside	1.22E+00	2.33E+00	1.10E-03	up

50		5,7,3',4'-Tetrahydroxyflavanone (Eriodictyol)		1.23E+00	2.09E+00	4.32E-04	up
51		2,4,2',4'-tetrahydroxy-3'-prenylchalcone		1.17E+00	2.03E+00	7.37E-03	up
52		Eriodictyol-7-O-glucoside		1.23E+00	4.88E-01	4.58E-04	down
53		Phloretin		1.25E+00	4.42E-01	1.86E-05	down
54		5,7,3',4',5'-Pentahydroxydihydroflavone		1.24E+00	3.97E-01	3.23E-04	down
55		Phloretin-2'-O-glucoside (Phlorizin)		1.25E+00	3.44E-01	1.63E-05	down
56		Pinocembrin (Dihydrochrysin)		1.25E+00	1.90E-01	2.44E-06	down
57	Gallic acid derivatives	1,3-Trigallic acid		1.25E+00	2.06E+02	1.20E-05	up
58		1,4-Trigallic acid		1.24E+00	1.03E+01	3.55E-04	up
59		2,3-O-Digalloyl-1,4,6-tri-O-galloyl-glucose		1.24E+00	3.13E+00	2.11E-04	up
60		3-O-Methylgallic Acid		1.25E+00	2.77E+00	1.65E-05	up
61		1,2,3,6-Tetra-O-Galloyl-D-Glucose		1.24E+00	4.81E-01	1.55E-04	down
62		Monogalloyl-diglucose		1.24E+00	4.72E-01	1.59E-04	down
63		2-O-Galloyl-glucose		1.24E+00	3.92E-01	1.69E-04	down
64		1-O-Galloyl-D-glucose		1.25E+00	3.79E-01	3.19E-06	down
65		1,7-Di-O-galloyl-D-sedoheptulose		1.24E+00	3.46E-01	1.17E-04	down
66		6-O-Galloyl-glucose		1.25E+00	3.34E-01	2.27E-05	down
67		Ethyl gallate		1.25E+00	4.05E-02	3.73E-07	down
68		1,6-Di-O-Galloyl-D-Glucose	Yes	1.24E+00	1.17E-05	7.86E-05	down
69	Cinnamic acid-Coumaroyl and derivatives (direct)	4-Hydroxycinnamyl alcohol 4-D-glucoside		1.24E+00	8.45E+00	7.02E-05	up
70		7-Methoxy-5-Prenyloxycoumarin		1.25E+00	5.56E+00	5.37E-06	up
71		3-O-p-Coumaroylquinic acid O-glucoside		1.22E+00	4.19E+00	1.51E-03	up
72		1-O-[(E)-p-Coumaroyl]-D-glucose		1.21E+00	2.10E+00	1.84E-03	up
73		p-Coumaryl alcohol		1.25E+00	3.53E-01	4.21E-07	down
74		Cinnamic acid		1.25E+00	2.27E-01	9.70E-06	down
75		5,7-Dimethoxycoumarin	Yes	1.25E+00	2.76E-03	7.44E-10	down

76		3-O-p-Coumaroylquinic acid	Yes	1.25E+00	1.19E-03	4.08E-05	down
77	Phenylpropanes (indirect Cinnamic acid-Coumaroyl and derivatives)	Rhododendrol	Yes	1.24E+00	2.53E+04	1.03E-04	up
78		3-(3-Hydroxyphenyl)-propionate acid	Yes	1.24E+00	4.54E+03	1.30E-04	up
79		Sinapoyl malate	Yes	1.25E+00	3.46E+03	9.35E-09	up
80		Scopoletin-7-O-glucoside (Scopolin)	Yes	1.21E+00	3.00E+03	1.98E-03	up
81		(S)-2-Hydroxy-3-(4-Hydroxyphenyl)Propanoic Acid		1.24E+00	1.10E+01	2.84E-04	up
82		Isoimperatorin		1.25E+00	6.29E+00	1.28E-07	up
83		Osthole		1.24E+00	2.51E+00	3.74E-04	up
84		Syringin		1.22E+00	2.20E+00	1.80E-03	up
85		Phaseolic acid		1.23E+00	2.04E+00	6.66E-04	up
86		Ferulic acid		1.23E+00	4.19E-01	3.68E-04	down
87		Brevifolin carboxylic acid		1.25E+00	3.65E-01	1.37E-06	down
88		3-(4-Hydroxyphenyl)-propionic acid		1.19E+00	3.64E-01	4.45E-03	down
89		Brevifolin		1.17E+00	3.50E-01	5.79E-03	down
90		Maleoyl-caffeoylquinic acid		1.19E+00	3.31E-01	6.27E-03	down
91		Caffeoyl(p-Hydroxybenzoyl)tartaric acid		1.24E+00	3.06E-01	2.15E-04	down
92		6-O-Feruloyl-D-glucose		1.25E+00	3.05E-01	3.29E-05	down
93		Isoeugenol	Yes	1.25E+00	1.67E-03	3.06E-06	down
94	Phenylmethanes (indirect Cinnamic acid-Coumaroyl and derivatives)	2,3,4,5,6-pentahydroxyhexyl 2-hydroxybenzoate	Yes	1.23E+00	4.22E+03	3.25E-04	up
95		1-O-(3,4-Dihydroxy-5-methoxy-benzoyl)-glucoside		1.25E+00	3.01E+00	1.88E-05	up
96		Salicin		1.24E+00	2.64E+00	6.76E-05	up
97		3,5-Dihydroxy-4-methoxybenzoic acid		1.25E+00	2.52E+00	8.29E-06	up
98		4-O-Glucosyl-4-hydroxybenzoic acid		1.24E+00	2.27E+00	1.34E-04	up
99		Dimethyl Phthalate		1.21E+00	2.12E+00	3.27E-03	up
100		Protocatechuic aldehyde		1.23E+00	4.57E-01	8.23E-04	down
101		4-Hydroxybenzoic acid		1.22E+00	4.46E-01	1.07E-03	down

102		Salicylic acid-2-O-glucoside		1.23E+00	3.38E-01	4.17E-04	down
103		3-O-Digalloyl quinic acid		1.24E+00	2.62E-01	1.25E-04	down
104		Syringic acid		1.25E+00	2.16E-01	2.83E-05	down
105		3,4,5-Trimethoxyphenyl-1-O-Glucoside		1.25E+00	1.95E-01	5.27E-07	down
106	Phenylethanes (indirect Cinnamic acid-Coumaroyl and derivatives)	Methyl 2,4-dihydroxyphenylacetate		1.25E+00	9.07E+00	1.48E-08	up
107		2-phenylethyl- D-β- glucopyranoside		1.25E+00	3.53E+00	4.92E-06	up
108		p-Hydroxyphenyl acetic acid		1.24E+00	3.35E+00	3.99E-05	up
109		4'-Hydroxy-3'-methoxyacetophenone (Acetovanillone)		1.24E+00	3.09E-01	1.48E-04	down
110		4-Hydroxyacetophenone		1.24E+00	2.21E-01	9.30E-05	down
111		2',4'-Dihydroxy-6'-methoxyacetophenone		1.25E+00	2.18E-01	4.67E-07	down
112		3-Hydroxy-4-isopropylbenzylalcohol-3-O-glucoside		1.24E+00	1.27E-01	1.18E-04	down
113	Shikimic acids	5-O-Caffeoylshikimic acid	Yes	1.24E+00	6.02E+04	5.11E-05	up
114		3,5-Di-O-galloylshikimic acid	Yes	1.20E+00	9.38E+03	2.73E-03	up
115		3-Galloylshikimic acid		1.25E+00	8.08E+00	7.75E-06	up
116		Trans-5-O-(p-Coumaroyl)shikimate		1.16E+00	2.67E+00	1.01E-02	up
117	Amino acids	(5-L-Glutamyl)-L-amino acid	Yes	1.20E+00	1.65E+03	3.03E-03	up
118		N-Acetyl-L-glutamic acid		1.25E+00	5.97E+01	1.83E-05	up
119		N-Acetyl-L-Glutamine		1.25E+00	3.18E+00	6.23E-06	up
120		3-Hydroxy-3-methylpentane-1,5-dioic acid		1.24E+00	2.30E+00	1.46E-04	up
121		L-Citrulline		1.25E+00	4.47E-01	8.58E-06	down
122		L-Glutamine-O-glycoside		1.24E+00	3.61E-01	3.97E-04	down
123		L-α-Glutamyl-L-Glutamic Acid		1.25E+00	2.91E-01	1.79E-05	down
124		L-Glutamic acid-O-glycoside		1.25E+00	2.78E-01	2.83E-05	down
125		Trans-4-Hydroxy-L-proline		1.23E+00	2.05E-01	2.75E-04	down
126		Acetylleucine Monoethanolamine		1.24E+00	2.17E+02	4.97E-05	up
127		L-Glycyl-L-isoleucine		1.23E+00	3.18E+00	7.47E-04	up

128	Pipecolic acid		1.25E+00	2.93E+00	1.46E-05	up
129	L-Methionine		1.23E+00	2.31E+00	4.74E-04	up
130	L-Threonine		1.25E+00	4.40E-01	8.65E-06	down
131	L-Prolyl-L-Phenylalanine		1.24E+00	8.03E+00	1.02E-04	up
132	N-Acetyl-L-tyrosine		1.23E+00	3.76E+00	6.62E-04	up
133	L-Glycyl-L-phenylalanine		1.24E+00	3.32E+00	1.52E-04	up
134	L-Tryptophan		1.25E+00	3.75E-01	3.10E-05	down
135	3,4-Dihydroxy-L-phenylalanine		1.16E+00	3.74E-01	9.55E-03	down
136	Phenylacetylglycine	Yes	1.25E+00	9.51E+03	8.30E-05	up
137	Oxiglutatione		1.25E+00	3.10E+00	2.20E-05	up
138	L-Homoserine		1.25E+00	4.38E-01	7.41E-06	down
139	N,N-Dimethylglycine		1.25E+00	4.23E-01	5.37E-06	down
140	O-Acetylserine		1.25E+00	2.95E-01	2.80E-06	down
141	L-Cystine		1.22E+00	2.26E-01	8.08E-04	down
142	N-(3-Indolylacetyl)-L-alanine	Yes	1.25E+00	2.07E+03	8.11E-06	up
143	N-Acetyl-L-leucine		1.25E+00	9.30E+00	1.10E-07	up
144	N-Glycyl-L-leucine		1.25E+00	3.02E+00	7.10E-05	up
145	4-Aminobutyric acid		1.22E+00	5.75E+00	1.80E-03	up
146	2,6-Diaminooimelic acid		1.24E+00	2.76E-01	1.57E-04	down
147	9,10-Epoxyoctadecanoic Acid	Yes	1.25E+00	1.26E+03	1.66E-05	up
148	Cis-4,7,10,13,16,19-Docosahexaenoic Acid	Yes	1.23E+00	1.86E+02	5.81E-04	up
149	12,13-Epoxy-9-Octadecenoic Acid		1.25E+00	8.25E+00	1.82E-05	up
150	Cis-10-Heptadecenoic Acid		1.25E+00	8.12E+00	3.58E-06	up
151	9-Oxo-10E,12Z-octadecadienoic acid		1.25E+00	7.90E+00	4.57E-06	up
152	9(10)-EpOME;(9R,10S)-(12Z)-9,10-Epoxyoctadecenoic acid		1.25E+00	7.51E+00	1.92E-07	up
153	9-Hydroxy-12-oxo-15(Z)-octadecenoic acid		1.25E+00	6.55E+00	1.47E-06	up

154	Free fatty acids and glycerides	13-KODE; (9Z,11E)-13-Oxooctadeca-9,11-dienoic acid		1.25E+00	6.46E+00	1.26E-06	up
155		9-Hydroxy-10,12,15-octadecatrienoic acid		1.25E+00	6.36E+00	1.18E-06	up
156		Heptadecanoic acid		1.24E+00	5.35E+00	5.74E-05	up
157		9Z,11E,13Z-octadecatrienoic acid (Punicic acid)		1.25E+00	4.37E+00	1.32E-06	up
158		13S-Hydroperoxy-9Z,11E-octadecadienoic acid		1.25E+00	4.04E+00	3.73E-05	up
159		13(S)-HODE;13(S)-Hydroxyoctadeca-9Z,11E-dienoic acid		1.25E+00	4.04E+00	3.06E-06	up
160		9S-Hydroxy-10E,12Z-octadecadienoic acid		1.25E+00	4.01E+00	2.79E-07	up
161		9-Hydroxy-13-oxo-10-octadecenoic Acid		1.25E+00	3.64E+00	6.73E-06	up
162		9,12-Octadecadien-6-Ynoic Acid		1.23E+00	3.59E+00	5.82E-04	up
163		Eicosadienoic acid		1.25E+00	3.33E+00	5.98E-07	up
164		1-Eicosanol		1.24E+00	3.30E+00	2.30E-04	up
165		9-Hydroperoxy-10E,12,15Z-octadecatrienoic acid		1.22E+00	3.02E+00	1.64E-03	up
166		11-Octadecanoic acid(Vaccenic acid)		1.25E+00	2.96E+00	1.79E-08	up
167		9,12,13-Trihydroxy-10,15-octadecadienoic acid		1.25E+00	2.81E+00	2.87E-06	up
168		Arachidonic Acid		1.06E+00	2.38E+00	4.04E-02	up
169		γ-Linolenic Acid		1.25E+00	2.11E+00	5.76E-07	up
170		α-Linolenic Acid		1.25E+00	2.11E+00	1.17E-06	up
171		1-α-Linolenoyl-glycerol-2,3-di-O-glucoside		1.20E+00	3.23E-01	3.16E-03	down
172		1-Linoleoylglycerol-2,3-di-O-glucoside		1.18E+00	3.19E-01	5.94E-03	down
173		7S,8S-DiHODE; (9Z,12Z)-(7S,8S)-Dihydroxyoctadeca-9,12-dienoic acid		1.19E+00	2.97E-01	6.49E-03	down
174		8,15-Dihydroxy-5,9,11,13-eicosatetraenoic acid		1.25E+00	3.80E-02	6.05E-06	down
175		1-α-Linolenoyl-glycerol-3-O-glucoside	Yes	1.19E+00	1.42E-03	3.89E-03	down
176	Nucleotides	2-Deoxyribose-1-phosphate		1.22E+00	1.94E+01	1.29E-03	up
177		N6-Isopentenyladenine		1.25E+00	1.58E+01	2.42E-05	up
178		Nicotinate D-ribonucleoside		1.25E+00	9.75E+00	4.50E-06	up

179		5-Aminoimidazole ribonucleotide		1.25E+00	7.29E+00	1.63E-05	up
180		Nicotinamide adenine dinucleotide phosphate (NADP)		1.24E+00	4.02E+00	3.23E-04	up
181		Allopurinol		1.24E+00	2.84E+00	1.62E-04	up
182		Xanthosine		1.24E+00	2.42E+00	1.26E-04	up
183		Succinyladenosine		1.22E+00	2.36E+00	1.27E-03	up
184		Hypoxanthine		1.17E+00	2.21E+00	1.08E-02	up
185		Adenosine 5'-monophosphate		1.25E+00	2.12E+00	1.87E-05	up
186		1-methylguanidine		1.23E+00	4.34E-01	8.15E-04	down
187		5-Methylcytosine		1.20E+00	4.30E-01	4.83E-03	down
188		2'-Deoxycytidine		1.24E+00	3.76E-01	2.29E-04	down
189		2'-Deoxyadenosine-5'-monophosphate		1.24E+00	3.71E-01	4.60E-05	down
190		β-Pseudouridine		1.24E+00	3.38E-01	6.68E-05	down
191		Cytidine		1.25E+00	2.93E-01	1.55E-06	down
192		Cytosine		1.25E+00	2.68E-01	4.99E-06	down
193		Uridine		1.25E+00	2.43E-01	7.68E-06	down
194		Adenosine		1.25E+00	1.97E-01	7.89E-06	down
195		Guanosine		1.25E+00	1.75E-01	2.53E-06	down
196		2'-Deoxyadenosine		1.25E+00	1.63E-01	3.60E-05	down
197		Thymidine		1.25E+00	1.38E-01	1.92E-06	down
198		2'-Deoxyguanosine		1.25E+00	1.16E-01	3.24E-07	down
199		2'-Deoxyuridine	Yes	1.24E+00	9.41E-04	3.05E-04	down
200		2-Aminopurine	Yes	1.25E+00	6.62E-05	1.28E-05	down
201	Others	Catalposide	Yes	1.23E+00	3.51E+03	4.83E-04	up
202		Harpagoside		1.24E+00	1.46E+02	4.09E-04	up
203		Deoxyelephantopin		1.24E+00	1.77E+01	2.23E-04	up
204		Diosbulbin B		1.23E+00	1.34E+01	6.53E-04	up

205	Eucommioside		1.23E+00	5.20E+00	1.46E-03	up
206	Ursolic acid		1.24E+00	4.32E+00	5.92E-04	up
207	Costunolide		1.19E+00	2.07E+00	6.18E-03	up
208	1-Methylpiperidine-2-carboxylic acid	Yes	1.25E+00	6.32E+04	2.12E-05	up
209	Tryptamine	Yes	1.25E+00	2.10E+04	5.06E-06	up
210	LysoPC 19:2	Yes	1.25E+00	1.54E+04	2.16E-06	up
211	N-Acetyl-D-glucosamine-1-phosphate	Yes	1.24E+00	5.94E+03	1.01E-04	up
212	LysoPC 20:1	Yes	1.24E+00	5.65E+03	9.73E-05	up
213	Phthalic acid	Yes	1.22E+00	1.83E+03	2.56E-03	up
214	LysoPC 18:4	Yes	1.25E+00	1.30E+03	3.34E-05	up
215	Indole 3-acetic acid (IAA)	Yes	1.14E+00	7.16E+02	1.34E-02	up
216	LysoPC 19:1		1.24E+00	2.64E+01	1.11E-04	up
217	Dimethylmalonic acid		1.24E+00	2.37E+01	9.22E-05	up
218	Zygadenine		1.25E+00	2.32E+01	3.49E-06	up
219	LysoPC 16:1 (2n isomer)		1.25E+00	2.12E+01	4.55E-07	up
220	LysoPC 18:2 (2n isomer)		1.25E+00	2.06E+01	4.49E-06	up
221	2-Methylsuccinic acid		1.25E+00	2.03E+01	1.43E-07	up
222	LysoPC 18:1 (2n isomer)		1.25E+00	1.97E+01	4.42E-06	up
223	LysoPC 18:3 (2n isomer)		1.25E+00	1.83E+01	2.30E-06	up
224	LysoPC 17:2		1.25E+00	1.57E+01	6.62E-06	up
225	Fumaric acid		1.24E+00	1.49E+01	4.16E-04	up
226	Betaine		1.25E+00	1.45E+01	2.91E-05	up
227	D-Proline betaine		1.24E+00	1.33E+01	9.36E-05	up
228	LysoPC 20:3		1.24E+00	1.20E+01	2.52E-04	up
229	LysoPC 20:4		1.23E+00	1.18E+01	3.24E-04	up
230	(-)-Jasmonoyl-L-Isoleucine		1.25E+00	1.16E+01	3.96E-06	up

231	2-Oxoadipic acid	1.21E+00	1.06E+01	2.67E-03	up
232	LysoPE 17:1 (2n isomer)	1.25E+00	9.73E+00	1.15E-06	up
233	α-Ketoglutaric acid	1.25E+00	9.49E+00	1.31E-06	up
234	Pantetheine	1.25E+00	9.49E+00	1.40E-06	up
235	Benzoylformic acid	1.24E+00	9.34E+00	1.71E-04	up
236	Benzoic acid, 3,4,5-trihydroxy-, (1R,5R,6R)-3-carboxy-5,6-dihydroxy-3-cyclohexen-1-yl ester	1.25E+00	9.12E+00	8.08E-06	up
237	LysoPC 18:0 (2n isomer)	1.22E+00	9.09E+00	1.11E-03	up
238	LysoPC 16:0 (2n isomer)	1.24E+00	9.06E+00	8.88E-05	up
239	Riboflavin	1.25E+00	8.44E+00	1.89E-05	up
240	LysoPC 19:2 (2n isomer)	1.23E+00	8.07E+00	4.01E-04	up
241	LysoPE 16:1	1.25E+00	7.87E+00	3.60E-07	up
242	2-Hydroxyisocaproic acid	1.25E+00	7.30E+00	3.89E-05	up
243	Xylitol	1.24E+00	7.24E+00	6.77E-05	up
244	LysoPE 16:1 (2n isomer)	1.25E+00	7.24E+00	3.74E-07	up
245	Choline Alfoscerate	1.25E+00	6.61E+00	2.33E-07	up
246	Benzamide	1.24E+00	6.53E+00	1.99E-04	up
247	Phenethylamine	1.24E+00	6.31E+00	9.65E-05	up
248	Glutaric acid	1.25E+00	6.19E+00	9.90E-06	up
249	UDP-N-acetyl-alpha-D-glucosamine	1.25E+00	5.39E+00	4.13E-07	up
250	Ethylmalonic acid	1.25E+00	5.35E+00	5.49E-06	up
251	Cadaverine	1.25E+00	5.21E+00	9.35E-08	up
252	L-Ascorbic acid	1.19E+00	5.13E+00	4.12E-03	up
253	D-Galacturonic acid	1.24E+00	4.61E+00	1.69E-04	up

254	Rosiridoside B	1.24E+00	4.48E+00	4.71E-04	up
255	D-Glucoronic acid	1.25E+00	4.42E+00	8.67E-05	up
256	2-Isopropylmalic Acid	1.25E+00	4.31E+00	6.37E-07	up
257	LysoPC 14:0	1.24E+00	4.31E+00	1.06E-04	up
258	2-Methylglutaric acid	1.25E+00	4.12E+00	1.21E-05	up
259	2-Hydroxyisobutyric acid	1.24E+00	4.11E+00	2.13E-04	up
260	4-Aminoindole	1.25E+00	3.88E+00	2.54E-06	up
261	2-Hydroxybutyric Acid	1.24E+00	3.86E+00	1.06E-04	up
262	Sarmentosin	1.24E+00	3.85E+00	3.21E-04	up
263	N-Acetylputrescine	1.24E+00	3.83E+00	8.15E-05	up
264	Adipic Acid	1.25E+00	3.81E+00	2.70E-05	up
265	β-Hydroxyisovaleric acid	1.25E+00	3.73E+00	1.15E-05	up
266	4-Oxopentanoic Acid	1.25E+00	3.51E+00	1.09E-05	up
267	Succinic anhydride	1.25E+00	3.48E+00	2.49E-05	up
268	L-Gulono-1,4-Lactone	1.24E+00	3.37E+00	8.23E-05	up
269	Gluconic acid	1.25E+00	3.36E+00	2.60E-05	up
270	Agmatine	1.24E+00	3.32E+00	6.85E-05	up
271	3-Methyl-2-Oxobutanoic acid	1.25E+00	3.32E+00	3.28E-05	up
272	Aminomalonic acid	1.25E+00	3.11E+00	1.62E-07	up
273	Methylmalonic acid	1.25E+00	2.98E+00	7.82E-07	up
274	Succinic acid	1.25E+00	2.89E+00	3.63E-07	up
275	N-Oleoylethanolamine	1.25E+00	2.84E+00	1.85E-05	up
276	(2R,3S)-3-methylmalic acid	1.23E+00	2.73E+00	7.26E-04	up
277	D-Arabitol	1.25E+00	2.68E+00	2.62E-05	up
278	Ribitol	1.25E+00	2.66E+00	5.81E-05	up
279	Argininosuccinic acid	1.24E+00	2.45E+00	1.46E-04	up

280	4-Hydroxymandelonitrile	1.24E+00	2.39E+00	6.70E-05	up
281	(S)-2-Hydroxyglutaric Acid	1.15E+00	2.35E+00	1.12E-02	up
282	Cinidilide	1.13E+00	2.22E+00	2.02E-02	up
283	LysoPC 15:0	1.16E+00	2.11E+00	1.14E-02	up
284	3-Dehydro-L-Threonic Acid	1.25E+00	4.97E-01	6.91E-06	down
285	O-Phosphocholine	1.24E+00	4.62E-01	2.36E-04	down
286	3-Hydroxybutyric acid	1.24E+00	4.61E-01	7.30E-05	down
287	L-(-)-Malic acid	1.24E+00	4.55E-01	4.15E-05	down
288	3,4'-Dihydroxy-3'-methoxybenzenepentanoic acid	1.24E+00	4.30E-01	2.14E-04	down
289	N-Acetyl-D-mannosamine	1.25E+00	4.27E-01	4.50E-05	down
290	LysoPE 18:3	1.21E+00	4.24E-01	2.19E-03	down
291	Nicotinic Acid Methyl Ester (Methyl Nicotinate)	1.25E+00	4.18E-01	6.00E-06	down
292	2-Aminoethanesulfonic acid	1.23E+00	4.01E-01	1.02E-03	down
293	Nicotianamine	1.24E+00	3.87E-01	5.91E-05	down
294	5-Hydroxymethylfurfural	1.24E+00	3.86E-01	4.07E-04	down
295	2,2-Dimethylsuccinic acid	1.24E+00	3.86E-01	4.46E-04	down
296	Methoxyindoleacetic acid	1.25E+00	3.65E-01	4.83E-06	down
297	5,6-Dihydroxyindole-5-O-β-glucoside	1.17E+00	3.60E-01	1.02E-02	down
298	LysoPE 20:2	1.19E+00	3.54E-01	5.99E-03	down
299	Pyridoxine-5'-O-glucoside	1.24E+00	3.33E-01	6.90E-05	down
300	Histamine	1.25E+00	3.06E-01	5.55E-06	down
301	Indole	1.22E+00	2.95E-01	7.75E-04	down
302	D-Fructose	1.25E+00	2.46E-01	1.06E-05	down
303	D-Glucosamine	1.25E+00	2.41E-01	3.17E-06	down
304	D-Glucose	1.25E+00	2.21E-01	6.32E-06	down
305	1,10-Decanediol	1.20E+00	2.01E-01	2.47E-03	down

306	Phenylethanolamine		1.25E+00	1.94E-01	7.10E-07	down
307	L-Tyramine		1.25E+00	1.64E-01	1.08E-05	down
308	LysoPE 15:0		1.24E+00	1.47E-01	1.61E-04	down
309	LysoPE 14:0		1.24E+00	1.38E-01	1.50E-04	down
310	Anthranilate-1-O-Sophoroside		1.25E+00	1.38E-01	3.44E-05	down
311	3-Indolepropionic acid	Yes	1.19E+00	1.41E-03	5.57E-03	down
312	Clove chromone	Yes	1.24E+00	8.54E-04	1.47E-04	down
313	LysoPE 15:0 (2n isomer)	Yes	1.22E+00	7.54E-04	1.23E-03	down
314	4-Pyridoxic acid-O-glucoside	Yes	1.24E+00	6.09E-04	1.98E-04	down
