# Supplementary material for: Altitudinal Variation of Metabolites, Mineral Elements and Antioxidant Activities of Rhodiola crenulata (Hook.f. & Thomson) H.Ohba
Source: Molecules. 2021 Dec 5;26(23):7383. doi: 10.3390/molecules26237383 (PMC8658832; doi:10.3390/molecules26237383)
Supplement: Supplementary file 1 [file molecules-26-07383-s001.zip › 20211124-V5-Table S4.pdf]

Table S4: List of differential metabolites between RC-H vs. RC-M

Number	Class	Compounds	Biomarkers	VIP	Fold change	P-value	Type
1	Quercetin and derivatives	Quercetin-3-O-(6''-galloyl)galactoside	Yes	1.23E+00	7.40E+03	1.41E-04	up
2		Quercetin-3-O-robinobioside		1.24E+00	3.03E+00	2.91E-06	up
3		Quercetin-3-O-rutinoside-7-O-glucoside		1.22E+00	4.51E-01	7.06E-04	down
4		Quercetin-3-O-galactoside (Hyperin)		1.17E+00	3.91E-01	1.27E-02	down
5		Quercetin 7-O-beta-D-glucoside		1.24E+00	2.00E-01	1.34E-07	down
6		Rhamnetin-3-O-Rutinoside		1.21E+00	1.25E-01	1.81E-03	down
7		Isorhamnetin-3-O-rutinoside (Narcissin)		1.18E+00	1.23E-01	7.50E-03	down
8		7-O-Methxyl Quercetin (Rhamnetin)		1.24E+00	1.04E-01	6.48E-06	down
9		5-O-Methylquercetin (Azaleatin)		1.20E+00	6.96E-02	4.24E-03	down
10	Kaempferol and derivatives	Kaempferol-6,8-di-C-glucoside-7-O-glucoside		1.23E+00	3.28E+00	3.82E-04	up
11		8-Prenylkaempferol		1.24E+00	3.11E+00	1.26E-04	up
12		Kaempferol-3-O-neohesperidoside		1.22E+00	2.20E+00	1.21E-03	up
13		Dihydrokaempferol-3-O-glucoside		1.24E+00	2.69E-01	6.53E-08	down
14		6-C-MethylKaempferol-3-glucoside		1.19E+00	2.32E-01	3.04E-03	down
15		Kaempferol-4'-O-glucoside		1.22E+00	2.03E-01	7.47E-04	down
16		Dihydrokaempferol-7-O-glucoside		1.22E+00	1.66E-01	5.58E-04	down
17	Catechin and derivatives	Procyanidin C2		1.24E+00	5.62E+00	7.73E-05	up
18		Procyanidin C1		1.24E+00	5.35E+00	4.82E-05	up
19		Procyanidin C1 3'-O-gallate		1.24E+00	3.37E+00	9.96E-07	up
20		Procyanidin B2		1.23E+00	3.02E+00	2.28E-04	up
21		Procyanidin B1		1.22E+00	2.36E+00	9.33E-04	up
22		Epicatechin		1.24E+00	2.22E+00	2.12E-06	up
23	Luteolin and derivatives	5,7,3',4'-Tetrahydroxyflavone (Luteolin)		1.24E+00	4.75E+00	1.55E-06	up

24		5,7,3',4'-tetrahydroxyisoflavone (Isoluteolin)		1.24E+00	4.31E+00	3.50E-06	up
25		Luteolin-7-O-glucoside (Cynaroside)		1.24E+00	3.47E-01	5.92E-05	down
26		Luteolin-3'-O-glucoside		1.19E+00	2.06E-01	4.66E-03	down
27	Other flavonoids	5,7,3',4',5'-Pentahydroxydihydroflavone		1.24E+00	3.00E+00	2.23E-05	up
28		Phloretin-2'-O-glucoside (Phlorizin)		1.24E+00	2.83E+00	1.82E-04	up
29		Sachaloside IV		1.23E+00	4.79E-01	3.19E-04	down
30		Apigenin		1.22E+00	4.46E-01	9.62E-04	down
31		Isosalipurposide (Phlorizin Chalcone)		1.23E+00	4.15E-01	2.60E-04	down
32		Eriodictyol-7-O-glucoside		1.16E+00	3.29E-01	7.68E-03	down
33		5,7,3',4'-Tetrahydroxyflavanone (Eriodictyol)		1.24E+00	2.91E-01	1.14E-04	down
34		Rhodiolgin		1.24E+00	2.19E-01	1.34E-04	down
35		Eriodictyol-3'-O-glucoside		1.24E+00	1.93E-01	1.04E-05	down
36		2'-Hydoxy,5-methoxyGenistein-O-rhamnosyl-glucoside		1.18E+00	1.06E-01	8.69E-03	down
37		Sexangularetin-3-O-glucoside-7-O-rhamnoside		1.20E+00	1.05E-01	4.79E-03	down
38	Gallic acid derivatives	1,6-Di-O-Galloyl-D-Glucose	Yes	1.24E+00	1.31E+05	3.77E-06	up
39		Ethyl gallate		1.23E+00	2.10E+01	3.38E-04	up
40		1-O-Galloyl-D-glucose		1.24E+00	4.53E+00	4.76E-06	up
41		2-O-Galloyl-glucose		1.24E+00	3.93E+00	5.38E-05	up
42		6-O-Galloyl-glucose		1.23E+00	3.69E+00	1.41E-04	up
43		3-Hydroxy-5-Methylphenol-1-O-(6'-Digalloyl)Glucoside		1.24E+00	2.70E+00	3.66E-05	up
44		Monogalloyl-diglucose		1.21E+00	4.70E-01	2.45E-03	down
45		3-O-Digalloyl-1,2,4,6-O-tetragalloyl-D-glucose		1.20E+00	3.76E-01	1.89E-03	down
46		2,3-O-Digalloyl-1,4,6-tri-O-galloyl-glucose		1.24E+00	3.33E-01	6.23E-05	down
47		Ellagic acid		1.23E+00	2.80E-01	1.26E-04	down
48		1,4-Trigallic acid		1.23E+00	1.28E-01	4.08E-04	down
49		1,3-Trigallic acid		1.24E+00	2.65E-02	1.31E-05	down

50	Cinnamic acid-Coumaroyl and derivatives (direct)	3-O-p-Coumaroylquinic acid	Yes	1.12E+00	1.37E+03	2.12E-02	up
51		5,7-Dimethoxycoumarin	Yes	1.23E+00	4.46E+02	5.09E-04	up
52		Methyl 4-hydroxycinnamate		1.14E+00	4.52E-01	2.50E-02	down
53		3-Methyl-4,8-dihydroxy-3,4-dihydroisocoumarin		1.20E+00	3.31E-01	2.89E-03	down
54		7-Hydroxy-5-methoxycoumarin (Scopoletin)		1.24E+00	2.73E-01	8.82E-05	down
55	Phenylpropanes (indirect Cinnamic acid-Coumaroyl and derivatives)	Caffeoylbenzoyltartaric acid	Yes	1.24E+00	1.65E+03	1.88E-05	up
56		1,2-O-Diferuloylglycerol	Yes	1.21E+00	1.49E+03	1.11E-03	up
57		Chlorogenic acid methyl ester	Yes	1.22E+00	8.69E+02	1.31E-03	up
58		Isoeugenol	Yes	1.24E+00	3.45E+02	9.29E-05	up
59		Methyl caffeate	Yes	1.21E+00	1.47E+02	1.31E-03	up
60		Sinapic acid		1.23E+00	5.98E+00	1.53E-04	up
61		1-O-Glucosyl sinapate		1.24E+00	4.72E+00	1.67E-05	up
62		Syringoylcaffeoylquinic acid-D-glucose		1.12E+00	3.34E+00	3.13E-02	up
63		Maleoyl-caffeoylquinic acid		1.20E+00	3.31E+00	2.63E-03	up
64		5-O-Caffeoylquinic acid (Neochlorogenic acid)		1.23E+00	2.73E+00	1.85E-04	up
65		1'-O-(3,4-Dihydroxyphenethyl)-O-caffeoyl-glucoside		1.22E+00	2.36E+00	6.23E-04	up
66		Chlorogenic acid		1.21E+00	2.36E+00	9.94E-04	up
67		Ferulic acid		1.22E+00	2.05E+00	1.34E-03	up
68		Syringin		1.20E+00	4.74E-01	2.88E-03	down
69		Sinapoyl malate		1.24E+00	1.64E-01	1.25E-05	down
70		(S)-2-Hydroxy-3-(4-Hydroxyphenyl)Propanoic Acid		1.23E+00	8.28E-02	2.85E-04	down
71		Scopoletin-7-O-glucoside (Scopolin)	Yes	1.22E+00	3.34E-04	1.98E-03	down
72		3-(3-Hydroxyphenyl)-propionate acid	Yes	1.23E+00	2.20E-04	1.30E-04	down
73	Phenylmethanes (indirect Cinnamic acid-Coumaroyl and derivatives)	4-Hydroxybenzyl Alcohol	Yes	1.23E+00	4.98E+02	2.39E-04	up
74		3,4,5-Trimethoxyphenyl-1-O-Glucoside		1.24E+00	1.18E+01	7.84E-07	up
75		Vanillic acid-4-O-glucoside		1.24E+00	3.16E+00	4.77E-06	up

76		Protocatechuic acid-4-O-glucoside		1.24E+00	3.13E+00	2.00E-06	up
77		2,5-Dihydroxybenzoic acid O-glucoside		1.24E+00	2.95E+00	3.47E-05	up
78		2,4,6-Trihydroxybenzoic acid		1.21E+00	2.73E+00	1.23E-03	up
79		3-O-Digalloyl quinic acid		1.24E+00	2.60E+00	3.28E-05	up
80		Salicin		1.23E+00	2.10E+00	5.50E-04	up
81		4-O-Glucosyl-4-hydroxybenzoic acid		1.23E+00	4.33E-01	1.43E-04	down
82		4-Hydroxybenzaldehyde		1.18E+00	3.74E-01	6.04E-03	down
83	Phenylethanes (indirect Cinnamic acid-Coumaroyl and derivatives)	3,4-Dihydroxybenzeneacetic acid		1.24E+00	2.66E+00	1.09E-06	up
84		2',4'-Dihydroxy-6'-methoxyacetophenone		1.24E+00	2.56E+00	9.41E-06	up
85		4'-Hydroxy-3'-methoxyacetophenone (Acetovanillone)		1.19E+00	2.53E+00	3.85E-03	up
86		4-Acetylphenyl-glucoside (Picein)		1.20E+00	3.86E-01	5.32E-03	down
87		Mandelic acid		1.23E+00	3.24E-01	2.91E-04	down
88		Tyrosol		1.24E+00	2.92E-01	2.11E-06	down
89		2-phenylethyl- D-β- glucopyranoside		1.24E+00	2.77E-01	4.19E-06	down
90		Methyl 2,4-dihydroxyphenylacetate		1.24E+00	8.74E-02	2.71E-07	down
91		p-Hydroxyphenyl acetic acid		1.24E+00	4.43E-02	1.03E-05	down
92	Shikimic acids	3,5-Di-O-galloylshikimic acid		1.15E+00	3.22E-01	1.36E-02	down
93		5-O-Caffeoylshikimic acid	Yes	1.24E+00	1.66E-05	5.11E-05	down
94	Amino acids	N-α-Acetyl-L-ornithine		1.24E+00	9.65E+00	7.36E-07	up
95		L-Citrulline		1.24E+00	9.30E+00	2.11E-05	up
96		L-Glutamine		1.24E+00	8.67E+00	1.39E-06	up
97		L-Arginine		1.24E+00	7.22E+00	7.27E-06	up
98		Homoarginine		1.24E+00	5.71E+00	2.41E-05	up
99		L-Ornithine		1.24E+00	4.34E+00	3.82E-05	up
100		5-Oxo-L-Proline		1.24E+00	4.27E+00	1.24E-05	up
101		L-α-Glutamyl-L-Glutamic Acid		1.23E+00	3.82E+00	1.99E-04	up

102	L-Glutamic acid-O-glycoside		1.23E+00	2.28E+00	2.70E-04	up
103	3-Hydroxy-3-methylpentane-1,5-dioic acid		1.23E+00	4.53E-01	1.75E-04	down
104	N-Acetyl-L-Glutamine		1.24E+00	4.48E-01	2.24E-05	down
105	N-Acetyl-L-Arginine		1.19E+00	3.92E-01	4.23E-03	down
106	N-Acetyl-L-glutamic acid		1.24E+00	1.20E-02	1.79E-05	down
107	N-Acetyl-L-methionine	Yes	1.24E+00	4.26E+03	8.02E-06	up
108	L-Methionine		1.24E+00	2.32E+01	2.26E-06	up
109	L-Lysine		1.24E+00	8.70E+00	1.53E-05	up
110	L-Isoleucine		1.24E+00	8.54E+00	1.17E-04	up
111	L-Isoleucyl-L-Aspartate		1.24E+00	7.02E+00	4.93E-05	up
112	L-Aspartic Acid		1.24E+00	4.88E+00	2.02E-06	up
113	L-Asparagine		1.23E+00	4.86E+00	1.60E-04	up
114	N-Acetyl-L-Aspartic Acid		1.23E+00	3.48E+00	8.16E-05	up
115	L-Threonine		1.23E+00	2.58E+00	1.75E-04	up
116	Acetylleucine Monoethanolamine		1.24E+00	2.55E-03	4.93E-05	down
117	L-Tryptophan		1.24E+00	1.49E+01	1.06E-07	up
118	L-Phenylalanine		1.24E+00	8.55E+00	6.73E-06	up
119	L-Tyrosine		1.24E+00	4.57E+00	2.35E-05	up
120	L-Aspartyl-L-Phenylalanine		1.24E+00	4.40E+00	2.16E-05	up
121	5-Hydroxy-L-tryptophan		1.24E+00	4.24E+00	5.20E-06	up
122	L-Alanyl-L-Phenylalanine		1.23E+00	2.67E+00	2.53E-04	up
123	3,4-Dihydroxy-L-phenylalanine		1.20E+00	2.13E+00	2.76E-03	up
124	N-Acetyl-L-tyrosine		1.23E+00	2.65E-01	6.60E-04	down
125	L-Prolyl-L-Phenylalanine		1.24E+00	6.18E-02	8.12E-05	down
126	N,N-Dimethylglycine		1.24E+00	6.13E+00	1.70E-09	up
127	S-(5'-Adenosy)-L-homocysteine		1.15E+00	3.53E+00	1.12E-02	up

128		L-Homoserine		1.24E+00	3.00E+00	7.92E-08	up
129		S-(Methyl)glutathione		1.20E+00	2.31E+00	3.53E-03	up
130		O-Acetylserine		1.24E+00	2.21E+00	2.92E-05	up
131		Oxiglutatione		1.22E+00	2.08E+00	7.88E-04	up
132		Phenylacetylglycine	Yes	1.24E+00	1.05E-04	8.30E-05	down
133		L-Prolyl-L-Leucine		1.24E+00	1.75E+01	7.54E-07	up
134		L-Leucine		1.24E+00	8.51E+00	2.19E-05	up
135		Cycloleucine		1.24E+00	7.92E+00	1.66E-07	up
136		L-Valine		1.24E+00	6.86E+00	3.85E-07	up
137		N-(3-Indolylacetyl)-L-alanine		1.24E+00	1.76E-01	2.27E-05	down
138		N-Acetyl-L-leucine		1.24E+00	8.80E-02	1.02E-07	down
139		L-Histidine		1.24E+00	3.58E+00	6.28E-05	up
140		3-Methyl-L-Histidine		1.23E+00	2.73E+00	3.65E-04	up
141		5-Aminovaleric acid		1.16E+00	3.35E+00	6.94E-03	up
142		2-Aminoisobutyric acid		1.24E+00	2.07E+00	2.68E-06	up
143	Free fatty acids and glycerides	1-α-Linolenoyl-glycerol-3-O-glucoside	Yes	1.22E+00	3.31E+02	7.50E-04	up
144		8,15-Dihydroxy-5,9,11,13-eicosatetraenoic acid		1.24E+00	5.70E+00	2.92E-06	up
145		9,10,13-Trihydroxy-11-Octadecenoic Acid		1.24E+00	2.62E+00	1.49E-06	up
146		1-Linoleoylglycerol-2,3-di-O-glucoside		1.15E+00	2.40E+00	2.37E-02	up
147		7S,8S-DiHODE; (9Z,12Z)-(7S,8S)-Dihydroxyoctadeca-9,12-dienoic acid		1.22E+00	2.05E+00	8.80E-04	up
148		9Z,11E,13Z-octadecatrienoic acid (Punicic acid)		1.24E+00	4.91E-01	1.40E-05	down
149		11-Octadecanoic acid(Vaccenic acid)		1.24E+00	4.58E-01	2.84E-08	down
150		1-Eicosanol		1.23E+00	4.48E-01	5.10E-04	down
151		2-Linoleoylglycerol-1,3-di-O-glucoside		1.19E+00	4.47E-01	4.70E-03	down
152		γ-Linolenic Acid		1.24E+00	3.66E-01	1.75E-08	down

153		α-Linolenic Acid		1.24E+00	3.60E-01	2.19E-07	down
154		9S-Hydroxy-10E,12Z-octadecadienoic acid		1.24E+00	3.58E-01	4.35E-07	down
155		13(S)-HODE;13(S)-Hydroxyoctadeca-9Z,11E-dienoic acid		1.24E+00	3.58E-01	6.04E-06	down
156		9-Hydroxy-10,12,15-octadecatrienoic acid		1.24E+00	3.51E-01	3.08E-06	down
157		13-KODE; (9Z,11E)-13-Oxooctadeca-9,11-dienoic acid		1.24E+00	3.50E-01	3.23E-06	down
158		12,13-Epoxy-9-Octadecenoic Acid		1.24E+00	3.26E-01	5.73E-05	down
159		2-Linoleoylglycerol-1-O-glucoside		1.24E+00	3.20E-01	5.78E-05	down
160		9,10-Epoxyoctadecanoic Acid		1.23E+00	3.03E-01	9.33E-05	down
161		9-Hydroperoxy-10E,12,15Z-octadecatrienoic acid		1.21E+00	3.00E-01	1.13E-03	down
162		Heptadecanoic acid		1.24E+00	2.94E-01	6.99E-05	down
163		2-α-Linolenoyl-glycerol-1,3-di-O-glucoside		1.23E+00	2.73E-01	2.00E-04	down
164		9-Oxo-10E,12Z-octadecadienoic acid		1.24E+00	2.72E-01	9.19E-06	down
165		1-Oleoyl-Sn-Glycerol		1.24E+00	2.69E-01	6.05E-05	down
166		1-Stearidonoyl-Glycerol		1.24E+00	2.66E-01	9.03E-07	down
167		9(10)-EpOME;(9R,10S)-(12Z)-9,10-Epoxyoctadecenoic acid		1.24E+00	2.53E-01	3.41E-07	down
168		2-α-Linolenoyl-glycerol-1-O-glucoside		1.24E+00	2.50E-01	5.61E-05	down
169		Cis-10-Heptadecenoic Acid		1.24E+00	2.01E-01	5.15E-06	down
170		2-Aminopurine	Yes	1.24E+00	4.11E+03	4.65E-05	up
171		2'-Deoxyuridine	Yes	1.22E+00	7.48E+02	1.02E-03	up
172		1-Methyladenine		1.24E+00	1.03E+01	2.48E-06	up
173		Thymidine		1.24E+00	8.94E+00	5.54E-06	up
174	Nucleotides	6-Methylmercaptopurine		1.24E+00	8.26E+00	8.48E-08	up
175		2'-Deoxyguanosine		1.24E+00	7.91E+00	7.05E-08	up
176		2'-Deoxycytidine		1.24E+00	7.43E+00	7.25E-05	up
177		5-Methylcytosine		1.18E+00	5.18E+00	4.22E-03	up
178		2'-Deoxyadenosine		1.23E+00	4.81E+00	1.67E-04	up

179		Uridine	1.21E+00	4.61E+00	2.75E-03	up
180		Adenosine 5'-monophosphate	1.24E+00	3.43E+00	5.59E-05	up
181		Adenosine 5'-diphosphate	1.23E+00	3.25E+00	1.32E-04	up
182		β-Pseudouridine	1.21E+00	3.18E+00	1.51E-03	up
183		1-methylguanidine	1.21E+00	2.84E+00	1.30E-03	up
184		Isoguanine	1.23E+00	2.36E+00	1.29E-04	up
185		Cytosine	1.24E+00	2.21E+00	3.17E-06	up
186		2'-Deoxyadenosine-5'-monophosphate	1.19E+00	2.12E+00	2.56E-03	up
187		Guanine	1.23E+00	2.03E+00	1.99E-04	up
188		Isopentenyladenine-7-N-glucoside	1.20E+00	2.01E+00	2.36E-03	up
189		9-(Arabinosyl)hypoxanthine	1.22E+00	4.76E-01	1.44E-03	down
190		Adenine	1.23E+00	4.63E-01	2.85E-04	down
191		Xanthosine	1.24E+00	4.51E-01	9.29E-05	down
192		Nicotinamide adenine dinucleotide phosphate (NADP)	1.18E+00	4.40E-01	4.88E-03	down
193		5-Aminoimidazole ribonucleotide	1.24E+00	3.66E-01	6.93E-05	down
194		Guanosine 5'-monophosphate	1.21E+00	3.48E-01	2.79E-03	down
195		Succinyladenosine	1.22E+00	3.06E-01	6.31E-04	down
196		2-Deoxyribose-1-phosphate	1.20E+00	3.03E-01	4.10E-03	down
197		Allopurinol	1.23E+00	2.79E-01	8.49E-05	down
198		Hypoxanthine	1.21E+00	2.75E-01	9.99E-04	down
199		Xanthine	1.21E+00	2.65E-01	2.59E-03	down
200		Nicotinate D-ribonucleoside	1.24E+00	1.15E-01	5.64E-06	down
201		N6-Isopentenyladenine	1.24E+00	1.34E-02	1.90E-05	down
202		Sterebin A	1.15E+00	6.43E+00	1.62E-02	up
203	Others	Kaurenoic acid	1.21E+00	5.44E+00	1.44E-03	up
204		Xanthatin	1.16E+00	3.49E+00	7.47E-03	up

205	(-)-Borneol		1.14E+00	2.94E+00	1.34E-02	up
206	Curcolone		1.18E+00	2.43E+00	8.48E-03	up
207	Taraxerol		1.04E+00	2.38E+00	4.31E-02	up
208	Deoxyelephantopin		1.19E+00	4.58E-01	3.22E-03	down
209	Eucommioside		1.19E+00	4.34E-01	2.89E-03	down
210	Diosbulbin B		1.20E+00	2.86E-01	2.17E-03	down
211	Ursolic acid		1.23E+00	1.97E-01	2.96E-04	down
212	Catalposide	Yes	1.23E+00	2.85E-04	4.83E-04	down
213	3-Indolepropionic acid	Yes	1.24E+00	3.13E+03	3.17E-06	up
214	3-Aminosalicylic acid	Yes	1.22E+00	1.42E+03	1.04E-03	up
215	4-Pyridoxic acid-O-glucoside	Yes	1.22E+00	9.31E+02	1.49E-03	up
216	Solatriose		1.24E+00	4.37E+01	3.45E-07	up
217	2,2-Dimethylsuccinic acid		1.24E+00	2.70E+01	5.38E-06	up
218	Phosphoenolpyruvate		1.24E+00	2.68E+01	2.17E-05	up
219	4-Hydroxymandelonitrile		1.24E+00	2.13E+01	1.44E-06	up
220	Methoxyindoleacetic acid		1.24E+00	1.87E+01	1.56E-06	up
221	Indole		1.23E+00	1.65E+01	2.11E-04	up
222	D-Fructose-1,6-biphosphate		1.23E+00	1.51E+01	1.19E-04	up
223	Indole-3-lactic acid		1.24E+00	1.08E+01	1.25E-04	up
224	D-Glucose 6-phosphate		1.24E+00	1.08E+01	2.92E-05	up
225	Glucose-1-phosphate		1.24E+00	1.03E+01	3.20E-05	up
226	6-Deoxyfagomine		1.24E+00	9.15E+00	6.36E-06	up
227	Piperidine		1.24E+00	9.03E+00	7.15E-06	up
228	6-Aminocaproic acid		1.23E+00	8.99E+00	2.48E-04	up
229	N-Benzylmethylene isomethylamine		1.24E+00	8.79E+00	6.83E-06	up
230	Anthranilate-1-O-Sophoroside		1.24E+00	5.33E+00	1.45E-06	up

231	O-Phosphorylethanolamine	1.24E+00	5.32E+00	8.62E-07	up
232	D-Ribose	1.24E+00	3.89E+00	6.59E-05	up
233	L-Tartaric acid	1.18E+00	3.80E+00	3.64E-03	up
234	1-Aminocyclopropane-1-carboxylic acid	1.24E+00	3.78E+00	6.03E-05	up
235	Nicotinic Acid Methyl Ester(Methyl Nicotinate)	1.24E+00	3.72E+00	1.44E-07	up
236	2-Hydroxy-4-methylpentanoic acid	1.21E+00	3.28E+00	1.52E-03	up
237	D-Pantothenic Acid	1.24E+00	3.12E+00	2.34E-06	up
238	Cocamidopropyl betaine	1.19E+00	2.74E+00	3.69E-03	up
239	D-(+)-Sucrose	1.24E+00	2.54E+00	7.29E-06	up
240	D-Sedoheptuiose 7-phosphate	1.24E+00	2.51E+00	2.81E-05	up
241	Turanose	1.22E+00	2.47E+00	1.33E-03	up
242	1,10-Decanediol	1.11E+00	2.45E+00	4.93E-02	up
243	L-Tyramine	1.24E+00	2.43E+00	2.38E-06	up
244	Acetylcholine	1.24E+00	2.32E+00	3.40E-05	up
245	O-Phosphocholine	1.23E+00	2.28E+00	3.07E-04	up
246	Glucarate O-Phosphoric acid	1.23E+00	2.26E+00	2.21E-04	up
247	Pyridoxine-5'-O-glucoside	1.20E+00	2.25E+00	2.15E-03	up
248	Domesticoside	1.24E+00	2.23E+00	1.36E-06	up
249	L-Citramalic acid	1.17E+00	2.23E+00	6.38E-03	up
250	5,6-Dihydroxyindole-5-O-β-glucoside	1.23E+00	2.13E+00	5.69E-04	up
251	Phenylethanolamine	1.24E+00	2.12E+00	1.05E-05	up
252	Histamine	1.23E+00	2.02E+00	4.74E-04	up
253	Choline	1.24E+00	2.01E+00	6.32E-06	up
254	N-Oleoylethanolamine	1.24E+00	4.92E-01	3.51E-05	down
255	(S)-2-Hydroxyglutaric Acid	1.17E+00	4.86E-01	1.66E-02	down
256	LysoPC 18:0	1.20E+00	4.83E-01	5.24E-03	down

257	Benzoic acid, 3,4,5-trihydroxy-, (1R,5R,6R)-3-carboxy-5,6-dihydroxy-3-cyclohexen-1-yl ester	1.24E+00	4.72E-01	1.10E-04	down
258	LysoPE 16:0	1.22E+00	4.64E-01	6.15E-04	down
259	LysoPE 17:1 (2n isomer)	1.23E+00	4.62E-01	3.77E-04	down
260	(2R,3S)-3-methylmalic acid	1.22E+00	4.59E-01	1.67E-03	down
261	3,4'-Dihydroxy-3'-methoxybenzenepentanoic acid	1.20E+00	4.54E-01	3.30E-03	down
262	Mannitol	1.23E+00	4.47E-01	4.72E-04	down
263	LysoPC 18:2	1.23E+00	4.26E-01	2.93E-04	down
264	2-Hydroxyisobutyric acid	1.23E+00	4.17E-01	4.23E-04	down
265	LysoPC 18:1	1.14E+00	4.10E-01	1.21E-02	down
266	β-Hydroxyisovaleric acid	1.24E+00	3.99E-01	2.34E-05	down
267	D-Sorbitol	1.24E+00	3.98E-01	2.75E-05	down
268	LysoPC 16:0	1.24E+00	3.95E-01	1.50E-04	down
269	Propyl 2-(trimethylammonio)ethyl phosphate	1.23E+00	3.92E-01	1.78E-04	down
270	LysoPE 14:0 (2n isomer)	1.19E+00	3.90E-01	6.82E-03	down
271	LysoPC 17:0	1.14E+00	3.88E-01	1.21E-02	down
272	LysoPC 18:3	1.24E+00	3.73E-01	1.53E-06	down
273	2-Hydroxybutyric Acid	1.23E+00	3.61E-01	1.86E-04	down
274	3-Methyl-2-Oxobutanoic acid	1.24E+00	3.50E-01	5.74E-05	down
275	UDP-N-acetyl-alpha-D-glucosamine	1.24E+00	3.45E-01	5.52E-05	down
276	2-Hydroxyhexadecanoic acid	1.24E+00	3.44E-01	6.36E-06	down
277	4-Oxopentanoic Acid	1.24E+00	3.38E-01	3.86E-05	down
278	LysoPE 16:1 (2n isomer)	1.24E+00	3.21E-01	5.25E-06	down
279	LysoPE 16:1	1.24E+00	3.15E-01	1.73E-05	down
280	Succinic acid	1.24E+00	3.10E-01	3.63E-07	down
281	Methylmalonic acid	1.24E+00	3.09E-01	3.69E-07	down

282	Choline Alfoscerate	1.24E+00	2.96E-01	1.95E-07	down
283	Aminomalonic acid	1.24E+00	2.86E-01	2.58E-07	down
284	Fumaric acid	1.22E+00	2.72E-01	1.12E-03	down
285	L-Gulono-1,4-Lactone	1.24E+00	2.56E-01	3.68E-05	down
286	D-Pinitol	1.24E+00	2.48E-01	1.25E-06	down
287	Gluconic acid	1.24E+00	2.43E-01	1.98E-05	down
288	Pyridoxine	1.24E+00	2.33E-01	2.08E-05	down
289	Xylitol	1.24E+00	2.31E-01	1.01E-04	down
290	Agmatine	1.24E+00	2.28E-01	2.40E-05	down
291	Succinic anhydride	1.24E+00	2.26E-01	4.01E-06	down
292	N-Acetylputrescine	1.24E+00	2.24E-01	3.36E-05	down
293	Cadaverine	1.24E+00	2.23E-01	5.86E-08	down
294	2-Hydroxyisocaproic acid	1.24E+00	2.03E-01	4.76E-05	down
295	LysoPC 19:1	1.23E+00	2.01E-01	2.81E-04	down
296	LysoPC 19:2 (2n isomer)	1.23E+00	1.98E-01	5.61E-04	down
297	4-Aminoindole	1.24E+00	1.87E-01	1.76E-06	down
298	Pantetheine	1.23E+00	1.70E-01	1.01E-04	down
299	LysoPE 16:0 (2n isomer)	1.24E+00	1.70E-01	3.11E-05	down
300	Ethylmalonic acid	1.24E+00	1.67E-01	2.92E-06	down
301	Glutaric acid	1.24E+00	1.50E-01	9.03E-06	down
302	5,7-Dihydroxychromone	1.24E+00	1.50E-01	8.27E-07	down
303	Tryptamine	1.24E+00	1.48E-01	1.08E-05	down
304	LysoPC 14:0	1.24E+00	1.45E-01	4.14E-05	down
305	L-Ascorbic acid	1.21E+00	1.35E-01	3.18E-03	down
306	LysoPC 20:3	1.23E+00	1.21E-01	3.08E-04	down
307	D-Proline betaine	1.24E+00	1.20E-01	1.19E-04	down

308	LysoPC 17:2		1.24E+00	1.13E-01	8.37E-06	down
309	Benzoylformic acid		1.23E+00	1.12E-01	1.73E-04	down
310	LysoPE 18:2		1.23E+00	1.10E-01	5.11E-04	down
311	Riboflavin		1.24E+00	1.02E-01	1.82E-05	down
312	Zygadenine		1.24E+00	1.01E-01	4.57E-06	down
313	LysoPC 16:1 (2n isomer)		1.24E+00	9.99E-02	5.57E-07	down
314	2-Oxoadipic acid		1.20E+00	7.95E-02	2.49E-03	down
315	Benzamide		1.24E+00	7.18E-02	1.38E-04	down
316	2-Methylsuccinic acid		1.24E+00	7.14E-02	1.59E-07	down
317	Phenethylamine		1.24E+00	6.95E-02	6.56E-05	down
318	2-Isopropylmalic Acid		1.24E+00	6.92E-02	2.76E-07	down
319	LysoPC 18:3 (2n isomer)		1.24E+00	6.39E-02	2.38E-06	down
320	LysoPC 18:2 (2n isomer)		1.24E+00	6.38E-02	4.78E-06	down
321	Dimethylmalonic acid		1.24E+00	6.37E-02	1.01E-04	down
322	LysoPC 18:0 (2n isomer)		1.22E+00	6.05E-02	8.94E-04	down
323	LysoPC 18:1 (2n isomer)		1.24E+00	5.43E-02	4.46E-06	down
324	Betaine		1.24E+00	5.40E-02	2.72E-05	down
325	LysoPC 16:0 (2n isomer)		1.24E+00	3.80E-02	6.48E-05	down
326	α-Ketoglutaric acid		1.24E+00	3.31E-02	9.35E-07	down
327	(-)-Jasmonoyl-L-Isoleucine		1.24E+00	3.00E-02	3.06E-06	down
328	D-Glucoronic acid		1.24E+00	2.28E-02	3.29E-05	down
329	D-Galacturonic acid		1.24E+00	2.23E-02	5.43E-05	down
330	Indole 3-acetic acid (IAA)	Yes	1.17E+00	1.40E-03	1.34E-02	down
331	LysoPC 18:4	Yes	1.24E+00	7.68E-04	3.34E-05	down
332	LysoPC 15:0	Yes	1.22E+00	2.77E-04	1.06E-03	down
333	LysoPC 20:1	Yes	1.24E+00	1.77E-04	9.73E-05	down

334	LysoPC 19:2	Yes	1.24E+00	6.49E-05	2.16E-06	down
335	1-Methylpiperidine-2-carboxylic acid	Yes	1.24E+00	1.58E-05	2.12E-05	down
